# Brain atlas of the Mongolian gerbil (*Meriones unguiculatus*) in CT/MRI-aided stereotaxic coordinates

**DOI:** 10.1007/s00429-016-1259-0

**Published:** 2016-08-10

**Authors:** Susanne Radtke-Schuller, Gerd Schuller, Frank Angenstein, Oliver S. Grosser, Jürgen Goldschmidt, Eike Budinger

**Affiliations:** 1 Division of Neurobiology, Department Biology II, Ludwig-Maximilians-University Munich, Grosshadernerstrasse 2, Planegg-Martinsried, 82152 Munich, Germany; 2 German Center for Neurodegenerative Diseases with the Helmholtz Association, Functional Neuroimaging Group, Magdeburg, Germany; 3 Clinic for Radiology and Nuclear Medicine, University Hospital Magdeburg, Magdeburg, Germany; 4 Department Systems Physiology of Learning, Leibniz Institute for Neurobiology Magdeburg, Magdeburg, Germany; 5 Clinic of Neurology, University Hospital Magdeburg, Magdeburg, Germany; 6 Center for Behavioral Brain Sciences, Magdeburg, Germany

**Keywords:** Neuroanatomy, Rodent, Cytoarchitecture, Fiber architecture

## Abstract

A new stereotaxic brain atlas of the Mongolian gerbil (*Meriones unguiculatus*), an important animal model in neurosciences, is presented. It combines high-quality histological material for identification of brain structures with reliable stereotaxic coordinates. The atlas consists of high-resolution images of frontal sections alternately stained for cell bodies (Nissl) and myelinated fibers (Gallyas) of 62 rostro-caudal levels at intervals of 350 μm. Brain structures were named according to the Paxinos nomenclature for rodents. The accuracy of the stereotaxic coordinate system was improved substantially by comparing and matching the series of histological sections to in vivo brain images of the gerbil obtained by magnetic resonance imaging (MRI). The skull outlines corresponding to the MR images were acquired using X-ray computerized tomography (CT) and were used to establish the relationship between coordinates of brain structures and skull. Landmarks such as lambda, bregma, ear canals and occipital crest can be used to line up skull and brain in standard atlas coordinates. An easily reproducible protocol allows sectioning of experimental brains in the standard frontal plane of the atlas.

## Introduction

During the last decades, the Mongolian gerbil (*Meriones unguiculatus*, Thomas 1908) has emerged as an important animal model in neuroscience. It is a versatile and advantageous laboratory animal because of its robustness, its ease of handling and its reliable breeding under laboratory conditions.

Virtually all sensory systems, especially the auditory system, are being intensively studied in gerbils, involving a wide range of neuroanatomical and neurophysiological approaches. Topics include development and plasticity as well as effects of aging. Research in the motor system and investigations of behavioral mechanisms, learning and memory and of transmitter systems use gerbils as model organism as well. Due to a peculiarity of the cerebral arteries (circle of Willis) in Mongolian gerbils, cerebral infarction can be induced in a controllable way and has made it a widely used model for cerebral ischemia. It is also a model animal for inherited epilepsy, hippocampal seizure and pathogenesis of CNS infections.

Despite a large body of literature related to the investigation of the gerbil brain, the availability of brain atlases published for this animal species is limited. To date, there are two stereotaxic atlases of the gerbil’s brain. The ‘Stereotaxic Atlas of the Mongolian Gerbil Brain’ (Loskota et al. 1974) includes photographic montages of corresponding hemispheres of adjacent sections stained for myelinated fibers (Weil) and cell bodies (Nissl). Brain structures are outlined and labeled separately, while the neocortex is represented without subdivisions. The heavy shrinkage of the brain caused by the celloidin embedding technique was not corrected in the stereotaxic coordinates.

The brains used for the ‘Stereotaxic Atlas of the Gerbil Brain’ by Thiessen and Yahr (1977) were frozen and cut in a cryostat, which causes only little shrinkage and thus more reliably reproduces stereotaxic coordinates. This atlas incorporates the earlier ‘Stereotaxic Atlas of the Hypothalamus’ by Thiessen and Goar (1970). The atlas presents only schematic outlines of structures and does not provide illustrative material of the underlying Nissl-stained histological sections. In addition, the sectioning plane deviates from the conventional frontal plane in rodents perpendicular to the axis of the brain stem in both atlases.

Thus, the need for a new stereotaxic atlas of the gerbil brain that combines high-quality histological material to identify brain structures with reliable stereotaxic coordinates is evident. Brain sections are inevitably subject to distortions during tissue fixation and subsequent histological procedures (embedding, sectioning, staining and section mounting). Here, we improved the accuracy of the stereotaxic coordinate system substantially by comparing and matching the series of histological sections to in vivo brain images of the gerbil obtained by magnetic resonance imaging (MRI). Moreover, X-ray computerized tomography (CT) yielded the outlines of the skull corresponding to the MR images, which helped to establish the relationship between coordinates of brain structures and skull coordinates. This is essential for any stereotaxic procedure using landmarks on the skull to reliably target brain structures for recording, imaging, tracer or virus applications. The atlas can also be used effectively as a common reference base to collect and compare positional data from any kind of research in the gerbil brain.

## Methods

### Animals

Twenty-one young adult male Mongolian gerbils (*Meriones unguiculatus*) at the age of 4 months and weighing between 80 and 100 g were used for this study. Out of them, brains of seven animals were processed for cyto- and myeloarchitectonic features. Six other brains were additionally processed for chemo- and immunoarchitecture to support identification of anatomical structures. This material is not included in the atlas and will be published separately. Overall 10 CT scans of skulls and a total of 13 MR brain scans were performed in various combinations.

All experiments were in agreement with the NIH Guide for the Care and Use of Laboratory Animals (2011) and the guidelines of the European Communities Council Directive (86/609/EEC) and approved by the animal care committee of Sachsen-Anhalt, Germany.

### CT imaging

Animals were scanned under isoflurane anesthesia (1.0–1.5 % in 2:1 O_2_:N_2_O volume ratio) with the CT functionality of a NanoSPECT/CT scanner (Mediso Ltd., Budapest, Hungary). CT scans were made at 45 kVp, 1.77 μA, with 180 projections, 500 ms per projection and 96 μm pixel size. Images were reconstructed with the InVivoScope (vs.1.43) at isotropic voxel sizes of 100 μm and analyzed with the DICOM viewers Osirix (Pixmeo SARL, Bernex, Switzerland, v.5.1.7 64-bit) and the open source program AMIDE: A Medical Imaging Data Examiner (amide.exe 1.0.4, ^©^Andreas Loening, http://amide.sourceforge.net/; GNU GPL).

### MR imaging

Animals were anesthetized with isoflurane (1.0–1.5 % in 1:1 O_2_:N_2_ volume ratio) and fixed with bite bars in a head-holder to reduce motion artifacts. MR scans were performed on a Bruker Biospec 47/20 scanner (Bruker Biospin GmbH, Rheinstetten, Germany) at 4.7 T (free bore of 20 cm) equipped with a BGA 09 (400 mT/m) gradient system. A 35 mm Litzcage small animal imaging system (DotyScientific Inc., Colombus, SC, USA) was used for radio frequency (RF) excitation and signal reception. Two days before MRI measurements, animals were injected subcutaneously with an aqueous solution containing 1 μmol/g MnCl_2_ (manganese enhanced MRI: ME-MRI). A data set of T1-weighted images was obtained using a 3D MDEFT (modified driven equilibrium Fourier transform) pulse sequence with the following parameters: repetition time 13.6 ms; echo time 4.3 ms; flip angle 20°; field of view 30 × 30 mm^2^; matrix 256 × 256 (yielding a nominal in plane resolution of 117 × 117 µm^2^); standard frontal orientation; slice thickness 350 µm; 20 averages. Images were reconstructed using Bruker ParaVision 4.0 (Bruker Biospin GmbH, Rheinstetten, Germany) and exported as raw images as well as in DICOM format. The open source program AMIDE (amide.exe 1.0.4, ^©^Andreas Loening, http://amide.sourceforge.net/) was used to align CT and MR scans and to extract images shown in the atlas.

### Histology

Animals were anesthetized with a lethal dose of ketamine (40 mg/100 g body weight, i.p.) and xylazine (2 mg/100 g body weight, i.p.). When a deep anesthetic state marked by a complete loss of the flexor reflex at all limbs was reached, animals were perfused transcardially with 20 mL of phosphate buffered saline (0.1 M PBS, pH 7.4) supplemented with 0.1 % heparin followed by 200 mL of 4 % PFA (in 0.05 M PBS, pH 7.4). The brains were postfixed in the skull with 4 % PFA (in 0.05 M PBS, pH 7.4) at 4 °C for at least 7 days before removal to best preserve the brain shape.

Brains were cryo-protected in 22.5 % sucrose in PBS (0.05 M, pH 7.4) overnight and cut in a cryostat (LEICA CM 3050S) into four series of 40 µm thick frontal sections. The sections were directly mounted on gelatine-coated slides and dried overnight. Alternating section series were stained on-slide either for cells (Nissl) or for myelin (Gallyas 1979). The brains additionally processed for chemo- and immunoarchitecture were stained for cytochrome oxidase, acetylcholine-esterase (AChE), NADPH-diaphorase, calcium-binding proteins (parvalbumin, calbindin and calretinin) and neurofilament protein (SMI-32) in various combinations. Sections were imaged with a virtual slide microscope (VS120 S1, Olympus BX61VST, Olympus-Deutschland, Hamburg, Germany) at 10× magnification using the proprietary software dotSlide^®^ (Olympus).

### Atlas coordinate system

**Fig. 1:**
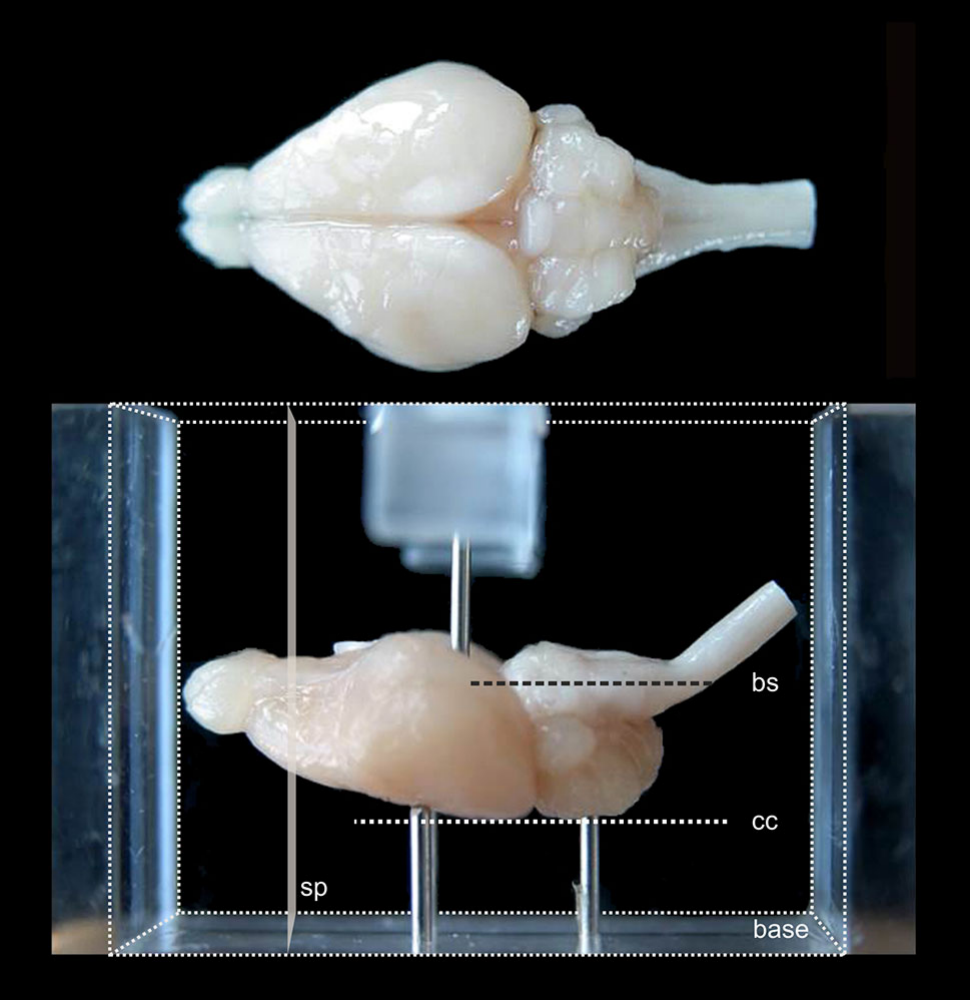
View of fixed gerbil brain positioned for embedding. In the *lower* *part* of the figure, the brain is shown in the acrylic glass box used for embedding (rectangular block volume indicated by *fine* *dotted lines*). The brain is positioned on three pins (one is hidden by the left front pin) protruding from the base so that the plane defined by the most dorsal elevation of cerebrum and cerebellum (*cc*) as well as the brainstem axis (*bs*) are aligned parallel to the base. The anterior and posterior surfaces of the embedding block define the frontal sectioning plane (*sp*) perpendicular to *cc* and *bs*. A pin protruding from a bracket over the side walls of the box (only partly shown) prevents the brain from being washed off when the embedding medium is poured into the box

The coordinate system of the brain atlas is based on the conventional definition of anatomical sectioning planes in rodents. Frontal sections are perpendicular to the brainstem axis, which in the Mongolian gerbil is also parallel to the plane defined by the most dorsal points of cerebrum and cerebellum (Fig. 1). This plane is therefore chosen as origin for the dorsoventral dimension with negative values in ventral direction. The lateral dimension is zeroed to the midsagittal plane with negative values towards the right and positive values towards the left side. The anterior to posterior coordinates of the atlas are given for different origins (bregma, lambda, interaural line and occipital crest as skull landmarks) and are valid for the skull in standard atlas orientation.

The frontal sectioning plane was implemented by a standardized embedding procedure using an acrylic glass box (Fig. 1). Each brain was oriented within the box so that the brainstem axis (Fig. 1*bs*) was parallel to the base of the box and the midsagittal plane lined up with the long axis of the box. Note that in this orientation the plane through the highest point of cerebellum and cerebrum (Fig. 1*cc*) is parallel to the base of the box and can therefore also be used to align the brain. The brain was stabilized in this orientation by adjustable supporting needles protruding from the bottom and from a bracket on top of the box. The volume around the brain was filled with embedding medium, namely a freshly prepared mixture of gelatin–albumin–glutaraldehyde. After 2–3 min, this mixture had hardened and the block was taken out of the box. Subsequently, the block was shock frozen in dry ice and mounted with its hind surface on the cutting platform of the cryostat. Due to the prior orientation within the box, the sectioning plane was now perpendicular to the long axis of the block and therefore also perpendicular to the brainstem axis and the horizontal plane through the highest cerebellar and cerebral points.

### Stereotaxic reference system

**Fig. 2:**
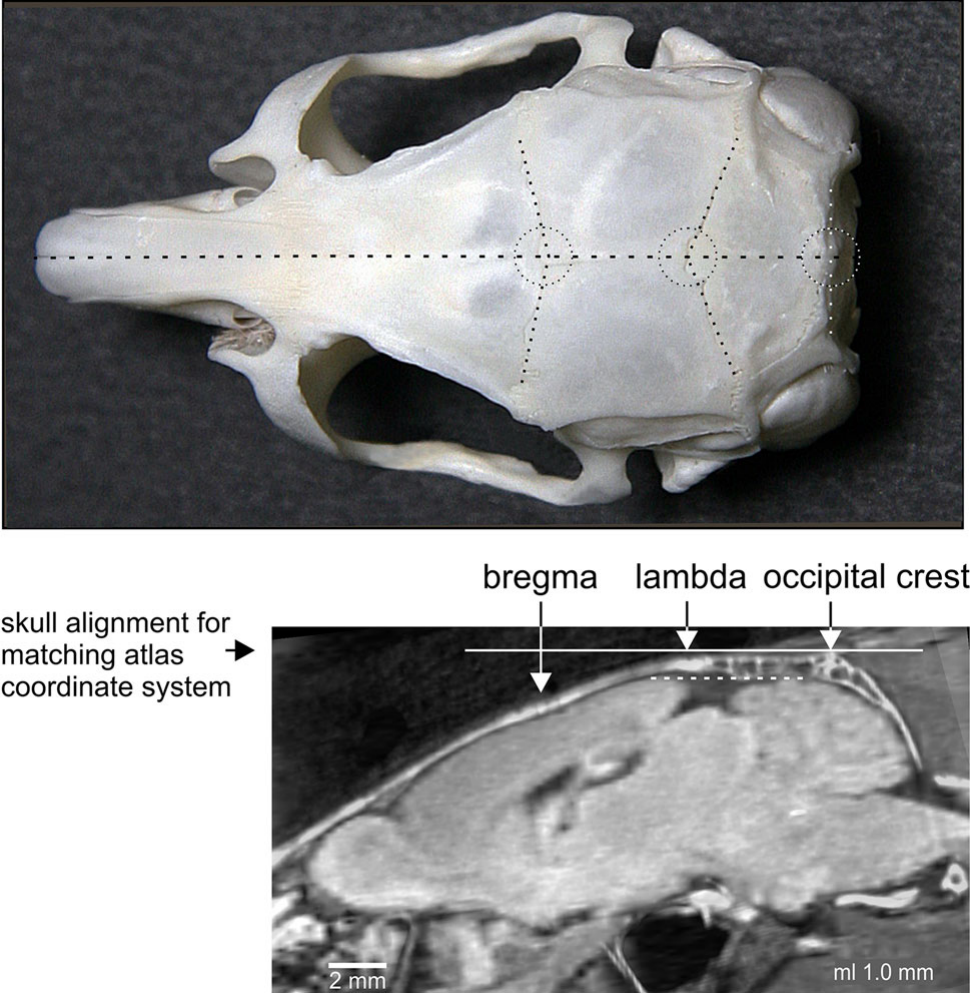
Atlas coordinate system and stereotaxic reference system. *Upper panel* landmarks bregma, lambda and occipital crest (*encircled*) on the skull of a Mongolian gerbil in a *top view*. They are defined by the intersection of lines (*dotted lines*) approximating the course of the bone sutures. *Lower panel* montage of CT skull image and MR brain image at a parasagittal distance of 1 mm in standard orientation of the atlas. The *solid line* corresponds to the horizontal plane through lambda and occipital crest, which is parallel to the plane through the highest points of cerebrum and cerebellum (*dotted line*)

In rats and mice, the connecting line through lambda and bregma coincides with that through lambda and occipital crest and is used as a horizontal guideline to align the in vivo brain in the classical planes (Paxinos and Watson 2007; Paxinos and Franklin 2001). In the Mongolian gerbil, the line linking lambda and bregma deviates from that linking lambda and occipital crest (Fig. 2, lower panel) and should, therefore, not be used as horizontal guideline to position the gerbil skull and brain in the atlas coordinate system. A horizontal adjustment of the skull along the line between lambda and occipital crest (Fig. 2, horizontal solid line) results in the best approximation to the atlas orientation (Fig. 2, dotted line) and is recommended as standard orientation.

### Selection of atlas series

The atlas series of histological sections was selected according to the following criteria:

- the entire series, alternately stained for cell bodies (Nissl) and myelin (Gallyas), had to show good staining quality and tissue preservation
- the atlas series had to match the MR scan of an average-sized brain, and relative distances of indicative structures of the brain had to show congruency with the distances in the available MR scans.

**Fig. 3:**
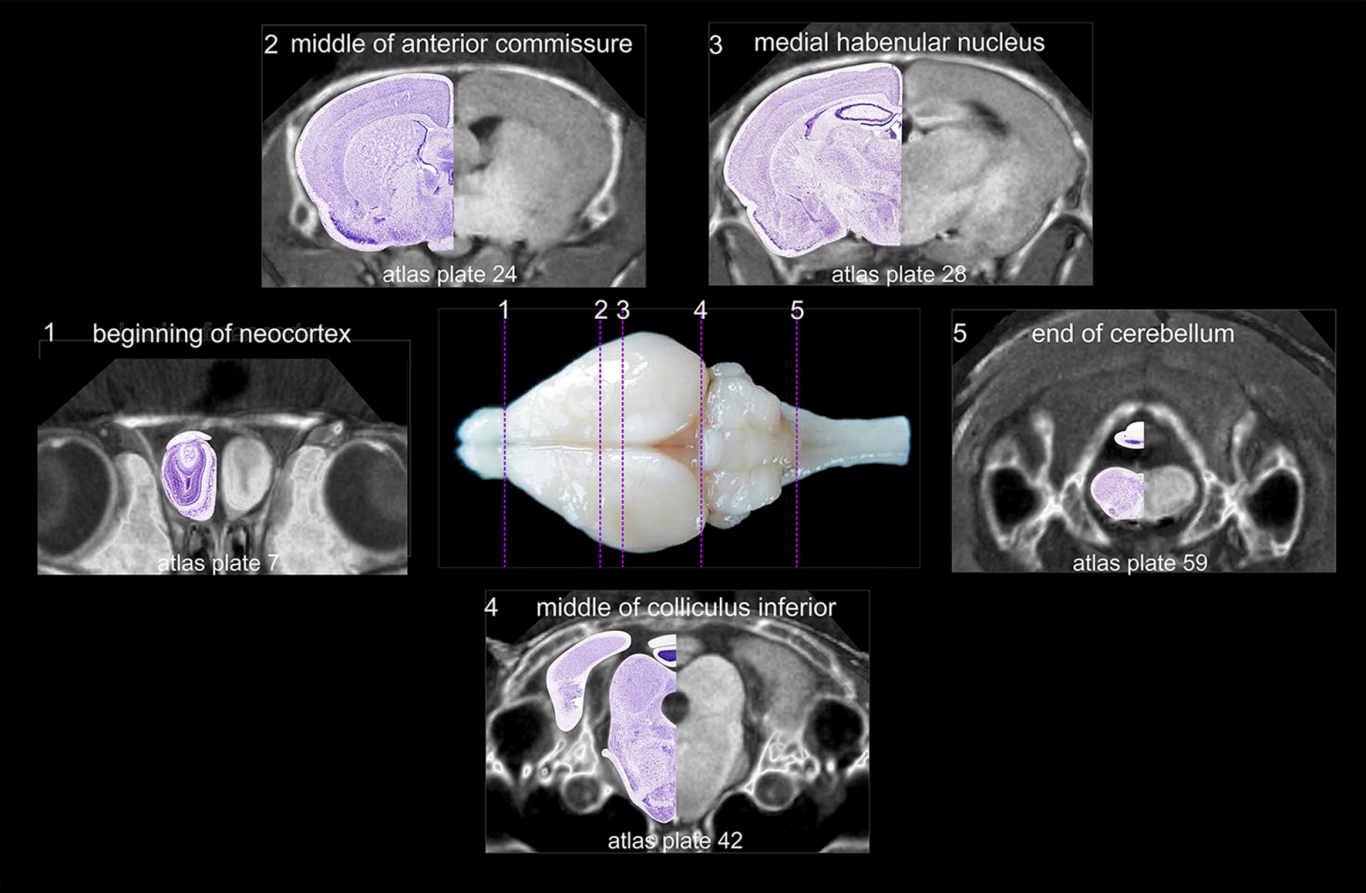
Indicative structures in histological and MRI brain series. The following structures were used (from rostral to caudal): beginning of neocortex (*1*), midline crossing of anterior commissure (*2*), distinct appearance of medial habenular nucleus (*3*), end of the superior colliculus (concurrent with the middle of the inferior colliculus) and (*4*) end of the cerebellum (*5*). Montages combine CT and MR scans and half of the corresponding Nissl-stained section. The anterior–posterior location of the corresponding atlas plates is indicated by dotted lines and respective numbers in the central brain image

The following structures that could clearly be determined both in histological sections and in MR slices were used as ‘indicative structures’ (Fig. 3): the rostral beginning of neocortex (1), the crossing of the anterior commissure (2), the distinct appearance of the medial habenular nucleus (3), the end of the superior colliculus concurrent with the middle of the inferior colliculus (4) and the end of the cerebellum (5). To judge brain size and to probe the consistency of individual histology series, the distances between indicative brain structures and the rostral pole of neocortex were evaluated and compared to the corresponding median distances in 13 MR scans (Fig. 3; Table 1).

**Table 1:** Distances between indicative structures in the atlas series

Distance from beginning of neocortex to	ATLAS hist. series (mm)	ATLAS MRI (mm)	All MRI (*N* = 13)	
			Median (mm)	Min/max (mm)
Middle of anterior commissure	5.95	5.78	6	5.7/6.3
Medial habenular nucleus	7.35	7.35	7.35	7.05/7.7
Middle of inferior colliculus	12.25	12.08	12.15	12.0/12.78
Posterior end of cerebellum	18.2	18.03	18.03	17.68/18.73

Distances of indicative structures relative to the rostral beginning of the neocortex were determined in the histological atlas series (column: ATLAS hist. series), in the atlas MRI series (column: ATLAS MRI) and across MRI series (columns: all MRI). Median values and minimal and maximal values are from thirteen MRI scans

The MR series that corresponded best to the median values was chosen as ‘ATLAS MRI’. The same distance measurements were performed in seven high-quality histological series. The series that corresponded best to the atlas MRI median values was designated as ‘ATLAS histology series’. Table 1 shows the conformance of the atlas histology series with the atlas MR scan and the median values of MRI series.

CT scans of the skull provide the interface to the brain coordinate system in vivo. Therefore, the available CT scans were overlaid to the atlas MRI. The CT scan matching best was chosen as ‘ATLAS CT’ series. For all CT scans the distances between bregma and the skull landmarks lambda, interaural line and occipital crest were calculated (Table 2). The comparison across animals corresponded well to the values of the atlas CT scan.

**Table 2:** Distances between landmarks on the gerbil skull

Distance between bregma and	ATLAS CT (mm)	All CT (*N* = 10)	
		Median (mm)	Min/max (mm)
Lambda	4.45	4.5	3.79/4.8
Interaural line	7.25	7.25	6.19/7.6
Occipital crest	9.98	9.95	9.79/10.9

Distances of skull landmarks lambda, interaural line and occipital crest are evaluated relative to bregma for the atlas CT scan (column: ATLAS CT) and as median distance values across all CT scans (columns: All CT). The range of values around the median is indicated by the minimum and maximum distance values taken from ten CT scans

### Preparation of images and plates

**Fig. 4:**
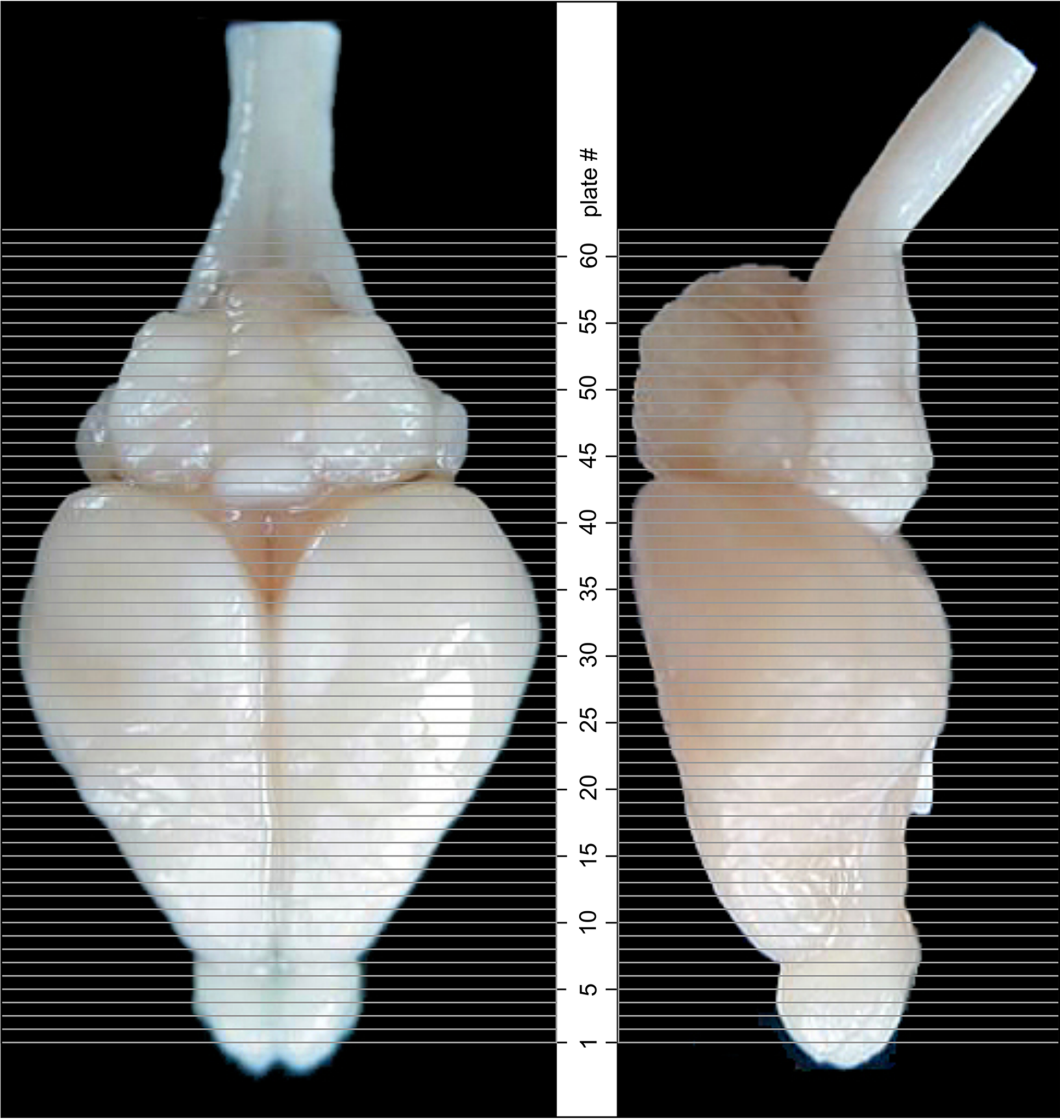
Anterior–posterior location of the atlas plates on the gerbil brain. *Upper panel* view from above. *Lower panel* side view. Distance between plates is 350 µm

For each 350 µm thick slice of the atlas MR series a corresponding Nissl-stained section of the atlas series was selected and grouped with the adjacent myelin-stained section to represent one of the 62 rostro-caudal levels (Fig. 4). Usually, every forth Nissl-stained section fitted best to the subsequent MR slice, which corresponded to a distance of 320 µm between the matching Nissl-stained sections. The 30 µm difference between the MR slices and the Nissl-stained sections can be explained by the shrinkage of the atlas brain due to histological processing, mainly fixation. This shrinkage is in the range of 8–10 % generally observed for cryo-protected frozen-cut brains with PFA fixation (4 %). Contrast and brightness of the images of the sections were corrected with Photoshop (CS6, Adobe Systems, San Jose, CA, USA), and distortions due to histological processing were compensated by slightly transforming the sections to optimize the congruency of anatomical structures between histological sections and MR images. Images were arranged in the atlas coordinate frame using CorelDraw graphics suite version X6 or X7 (Corel Corporation, Ottawa, ON, Canada). MR and CT images were adjusted according to the definition of the atlas coordinate system in 62 plates and reflect the in vivo orientation of the brain and skull. The images of cell- and myelin-stained sections were inserted in line with the corresponding MR image. The anterior–posterior coordinates of the plates are indicated relative to bregma, lambda, interaural line and the occipital crest. All outlines were drawn in CorelDraw on the base of the Nissl-stained section of each atlas plate. The structural boundaries seen in the corresponding myelin-stained section generally correlate well with these outlines.

### Anatomical structures, nomenclature and abbreviations

Anatomical structures were identified on the basis of cyto- and myeloarchitecture and their relative location. For comparison we mainly used the published atlases of the Mongolian gerbil brain (Loskota et al. 1974; Thiessen and Yahr 1977), the atlases and books for rat brain of Paxinos, Swanson and Zilles (Paxinos 1995, 2004; Paxinos and Watson 2007; Paxinos et al. 2009; Swanson 1992, 2004; Zilles 1985) and for mouse brain (Paxinos and Franklin 2001; Dong 2008; Franklin and Paxinos 2008; Watson and Paxinos 2010; Watson et al. 2012). Brain series stained for chemoarchitectonic markers were consulted to support the structural identification. Unfortunately, no unified neuroanatomical nomenclature exists to date (Swanson 2015). Therefore, we decided to use the widely accepted Paxinos nomenclature and abbreviations for naming structures. Auditory midbrain and brainstem nuclei for which gerbil specific terms were already established (Budinger et al. 2000, 2013; Mylius et al. 2013; Radtke-Schuller et al. 2015) were labeled according to these studies.

### Practical hints

Sectioning in atlas coordinates: It is also possible to section the brain in the standard atlas plane without the above described embedding procedure. In this case, the brain is positioned upside down on a flat surface so that it is seated with the cerebellum and cerebrum on the base. Then, part of the brain is cut off perpendicular to the base to create a surface for mounting the brain’s portion of interest on the cryostat platform. By subsequent sectioning of the brain parallel to this cutting surface the resulting sections correspond best to the frontal plane of the atlas.

Stereotaxic procedure: In addition to traditional landmarks and reference points such as lambda, bregma and interaural line, we recommend the occipital crest (Fig. 2) for anterior–posterior reference and adjustment of the skull in vivo. The traditional landmarks are often difficult to discern, show individual variations and cannot be accessed in some experimental approaches (e.g., interaural coordinates in auditory research where ear bars are avoided). In general, a higher precision of in vivo positioning of the skull can be achieved by using the specific pattern of skull profiles instead of single reference points [for profile oriented stereotaxic procedure see Schuller et al. (1986)].

**Index of structures**

The structures are listed in alphabetical order followed by their abbreviation and the plate number(s) of occurrence

**Table:** 

1st cerebellar lobule (lingula)	1Cb	46–48
2nd cerebellar lobule	2Cb	43–46
3rd cerebellar lobule	3Cb	43–49
3rd ventricle	3V	23–34
4th cerebellar lobule	4Cb	41–49
4th ventricle	4V	43–54
5th cerebellar lobule	5Cb	42–50
6th cerebellar lobule	6Cb	46–54
7th cerebellar lobule	7Cb	51–56
8th cerebellar lobule	8Cb	51–58
9th cerebellar lobule	9Cb	50–51
9th cerebellar lobule, a	9aCb	52–59
9th cerebellar lobule, b	9bCb	52–59
9th cerebellar lobule, c	9cCb	52–59
10th cerebellar lobule (nodule)	10Cb	50–55
**A**		
A11 dopamine cells	A11	30–31
A13 dopamine cells	A13	28–29
A5 noradrenaline cells	A5	44–47
abducens nerve	6n	46–47
abducens nucleus	6N	47
accessory nerve nucleus	11N	60–62
accessory neurosecretory nuclei	ANS	27–28
accumbens nucleus, core	AcbC	16–21
accumbens nucleus, shell	AcbSh	16–21
agranular insular cortex	AI	11–27
alveus of the hippocampus	alv	27–38
ambiguus nucleus, compact part	AmbC	52
ambiguus nucleus, loose part	AmbL	55
ambiguus nucleus, subcompact part	AmbSC	53–54
amygdalohippocampal area	AHi	29–33
amygdaloid fissure	af	31–32
amygdaloid intramedullary gray	IMG	27–28
amygdalopiriform transition area	APir	30–35
amygdalostriatal transition area	ASt	26–30
angular thalamic nucleus	AngT	28–28
ansoparamedian fissure	apmf	52–55
anterior amygdaloid area	AA	24–26
anterior auditory field	AAF	28–29
anterior cerebral artery	acer	23
anterior commissure, anterior part	aca	11–24
anterior commissure, intrabulbar part	aci	1–10
anterior commissure, posterior part	acp	23–25
anterior cortical amygdaloid nucleus	ACo	24–28
anterior hypothalamic area, anterior part	AHA	25–26
anterior hypothalamic area, central part	AHC	27–28
anterior hypothalamic area, posterior part	AHP	28
anterior olfactory nucleus, dorsal part	AOD	8–12
anterior olfactory nucleus, external part	AOE	6–10
anterior olfactory nucleus, lateral part	AOL	6–12
anterior olfactory nucleus, medial part	AOM	9–13
anterior olfactory nucleus, posterior part	AOP	14–16
anterior olfactory nucleus, ventral part	AOV	8–11
anterior olfactory nucleus, ventroposterior part	AOVP	11–15
anterior pretectal nucleus	APT	35
anterior pretectal nucleus, dorsal part	APTD	31–34
anterior pretectal nucleus, ventral part	APTV	32–34
anterior tegmental nucleus	ATg	40–41
anterodorsal thalamic nucleus	AD	26–27
anterolateral periolivary nucleus	ALPO	44
anteromedial thalamic nucleus	AM	25–28
anteromedial thalamic nucleus, ventral part	AMV	27
anterovent thalamic nucleus, dorsomedial part	AVDM	26–28
anteroventral periventricular nucleus	AVPe	23
anteroventral thalamic nucleus	AV	25
anteroventral thalamic nucleus, ventrolateral part	AVVL	26–27
aqueduct	Aq	35–42
arcuate hypothalamic nucleus	Arc	27–33
area postrema	AP	55–56
ascending fibers of the facial nerve	asc7	48
**B**		
Barrington’s nucleus	Bar	43–44
basal nucleus (Meynert)	B	24–29
basolateral amygdaloid nucleus, anterior part	BLA	25–29
basolateral amygdaloid nucleus, posterior part	BLP	27–32
basolateral amygdaloid nucleus, ventral part	BLV	25–27
basomedial amygdaloid nucleus, anterior part	BMA	25–27
basomedial amygdaloid nucleus, posterior part	BMP	28–31
bed nucleus of stria terminalis, fusiform part	Fu	23
bed nucleus of the accessory olfactory tract	BAOT	27
bed nucleus of the anterior commissure	BAC	24
bed nucleus of the stria terminalis	ST	22
bed nucleus of the stria terminalis, intraamygdaloid division	STIA	28–29
bed nucleus of the stria terminalis, lateral division, intermediate part	STLI	24
bed nucleus of the stria terminalis, lateral division, posterior part	STLP	23–24
bed nucleus of the stria terminalis, lateral division, ventral part	STLV	23–24
bed nucleus of the stria terminalis, medial division, anterior part	STMA	23–24
bed nucleus of the stria terminalis, medial division, posterior part	STMP	25–26
bed nucleus of the stria terminalis, medial division, ventral part	STMV	23–24
blood vessel	BV	21
Bötzinger complex	Bo	52
brachium of the inferior colliculus	bic	36–40
brachium of the superior colliculus	bsc	33–35
**C**		
caudal linear nucleus of the raphe	CLi	37–39
caudal periolivary nucleus	CPO	48
caudate putamen (striatum)	CPu	17–30
caudomedial entothinal cortex	CEnt	35–41
caudoventrolateral reticular nucleus	CVL	52–53
cell bridges of the ventral striatum	CB	20–22
central amygdaloid nucleus, capsular part	CeC	26–29
central amygdaloid nucleus, lateral division	CeL	27–28
central amygdaloid nucleus, medial division	CeM	25–29
central canal	CC	55–62
central cervical nucleus of the spinal cord	CeCv	56–62
central gray	CG	43
central gray of the pons	CGPn	45
central gray, alpha part	CGA	44–46
central gray, beta part	CGB	44–45
central gray, gamma part	CGG	46
central gray, nucleus O	CGO	44–45
central medial thalamic nucleus	CM	26–31
central nucleus of the inferior colliculus	CIC	39–42
centrolateral thalamic nucleus	CL	28–31
cerebellar white matter	cbw	43–57
cerebral peduncle	cp	28–39
choroid plexus	chp	24–54
cingulate cortex, area 1	Cg1	10–27
cingulate cortex, area 2	Cg2	19–27
cingulum	cg	17–34
claustrum	Cl	12–27
commissural stria terminalis	cst	26–27
commissure of the inferior colliculus	cic	42–43
commissure of the lateral lemniscus	cll	41–42
commissure of the superior colliculus	csc	34–36
copula of the pyramis	Cop	49–57
corpus callosum	cc	20–30
cortex-amygdala transition zone	CxA	24–26
crus 1 of the ansiform lobule	Crus1	43–54
crus 2 of the ansiform lobule	Crus2	49–55
cuneate fasciculus	cu	53–62
cuneate nucleus	Cu	52–62
cuneate nucleus, rotundus part	CuR	55–56
cuneiform nucleus	CnF	41–43
**D**		
decussation of the superior cerebellar peduncle	xscp	39–41
decussation of the trapezoid body	tzx	44–47
deep cerebral white matter	dcw	29–39
deep gray layer of the superior colliculus	DpG	33–41
deep white layer of the superior colliculus	DpWh	34–41
dentate gyrus	DG	30
dorsal acoustic stria	das	49–50
dorsal cochlear nucleus, deep core	DCDp	49–50
dorsal cochlear nucleus, fusiform layer	DCFu	48–50
dorsal cochlear nucleus, molecular layer	DCMo	48–50
dorsal cortex of the inferior colliculus	DCIC	40–43
dorsal corticospinal tract	dcs	60–62
dorsal endopiriform nucleus	DEn	12–32
dorsal fornix	df	26–27
dorsal hippocampal commissure	dhc	28–38
dorsal hypothalamic area	DA	29–30
dorsal lateral geniculate nucleus	DLG	29–33
dorsal lateral olfactory tract	dlo	5–12
dorsal motor nucleus of vagus	10N	53–58
dorsal nucleus of the lateral lemniscus	DNLL	41–42
dorsal paragigantocellular nucleus	DPGi	48–51
dorsal part of claustrum	DCl	16–26
dorsal peduncular cortex	DP	12–18
dorsal periolivary nucleus	DPO	45–47
dorsal raphe nucleus	DR	37–38
dorsal raphe nucleus, caudal part	DRC	43–44
dorsal raphe nucleus, dorsal part	DRD	39–42
dorsal raphe nucleus, lateral part	DRL	39–41
dorsal raphe nucleus, ventral part	DRV	39–42
dorsal spinocerebellar tract	dsc	52–62
dorsal subiculum	DS	33–36
dorsal tegmental decussation	dtgx	36–37
dorsal tegmental nucleus	DTg	44
dorsal tegmental nucleus, central part	DTgC	43
dorsal tegmental nucleus, pericentral part	DTgP	43
dorsal tenia tecta	DTT	11–18
dorsal transition zone	Dtr	12–15
dorsolateral orbital cortex	DLO	9–12
dorsolateral periaqueductal gray	DLPAG	36–42
dorsolateral periolivary nucleus	DLPO	44–47
dorsomedial hypothalamic nucleus	DM	29–32
dorsomedial hypothalamic nucleus, compact part	DMC	31
dorsomedial hypothalamic nucleus, dorsal part	DMD	31
dorsomedial hypothalamic nucleus, ventral part	DMV	31
dorsomedial nucleus of the inferior colliculus	DMIC	41–43
dorsomedial periaqueductal gray	DMPAG	35–42
dorsomedial spinal trigeminal nucleus	DMSp5	48–54
dorsomedial tegmental area	DMTg	43–45
dysgranular insular cortex	DI	13–27
**E**		
ectorhinal cortex	Ect	28–42
Edinger–Westphal nucleus	EW	36–38
entopeduncular nucleus	EP	27–28
entorhinal cortex	Ent	42
ependyma and subependymal layer	E	1–23
ethmoid thalamic nucleus	Eth	32
external capsule	ec	19–30
external cortex of the inferior colliculus	ECIC	38–44
external cuneate nucleus	ECu	52–56
external medullary lamina	eml	28–30
external plexiform layer of the accessory olfactory bulb	EPlA	5–9
external plexiform layer of the olfactory bulb	EPl	1–9
**F**		
F cell group of the vestibular complex	FVe	52
facial nerve	7n	45–47
facial nucleus, dorsal intermediate subnucleus	7DI	48–50
facial nucleus, dorsolateral subnucleus	7DL	48–50
facial nucleus, dorsomedial subnucleus	7DM	48–50
facial nucleus, lateral subnucleus	7L	49–51
facial nucleus, ventral intermediate subnucleus	7VI	48–50
facial nucleus, ventromedial subnucleus	7VM	48–50
fasciculus retroflexus	fr	27–36
fasciola cinereum	FC	29–33
field CA1 of the hippocampus	CA1	28–36
field CA2 of the hippocampus	CA2	28–32
field CA3 of the hippocampus	CA3	27–34
fimbria of the hippocampus	fi	22–30
flocculus	Fl	43–48
forceps major of the corpus callosum	fmj	34–39
forceps minor of the corpus callosum	fmi	13–18
fornix	f	23–32
frontal association cortex	FrA	7–9
frontal cortex, area 3	Fr3	11–17
**G**		
gelatinous layer of the caudal spinal trigeminal nucleus	Ge5	57–62
genu of the corpus callosum	gcc	19
genu of the facial nerve	g7	46–48
gigantocellular reticular nucleus	Gi	47–54
gigantocellular reticular nucleus, alpha part	GiA	48–51
gigantocellular reticular nucleus, ventral part	GiV	51–52
globular cell area, ventral cochlear nucleus	Gca	46–49
globus pallidus	GP	24–29
glomerular layer of the accessory olfactory bulb	GlA	5–10
glomerular layer of the olfactory bulb	Gl	1–11
glossopharyngeal nerve nucleus	9N	62
gracile fasciculus	gr	54–62
gracile nucleus	Gr	54–62
granular cell layer of the olfactory bulb	GrO	1–10
granular insular cortex	GI	13–27
granular layer of the dentate gyrus	GrDG	27–36
granule cell layer of cochlear nuclei	GrC	43–50
granule cell layer of the accessory olfactory bulb	GrA	3–10
**H**		
habenular commissure	hbc	31–32
hippocampal fissure	hif	30–34
hypoglossal nerve	12n	53–58
hypoglossal nucleus	12N	51–58
hypoglossal nucleus, geniohyoid part	12GH	57–58
**I**		
indusium griseum	IG	18–33
inferior cerebellar peduncle (restiform body)	icp	46–53
inferior olive, beta subnucleus	IOBe	56–58
inferior olive, cap of Kooy of the medial nucleus	IOK	56–57
inferior olive, dorsal nucleus	IOD	52–56
inferior olive, dorsomedial cell group	IODM	52–55
inferior olive, medial nucleus	IOM	52–55
inferior olive, principal nucleus	IOPr	52–56
inferior olive, subnucleus A of medial nucleus	IOA	57–58
inferior olive, subnucleus B of medial nucleus	IOB	56–58
inferior olive, subnucleus C of medial nucleus	IOC	56–58
infralimbic cortex	IL	14–18
interanterodorsal thalamic nucleus	IAD	26–27
interanteromedial thalamic nucleus	IAM	27
intercalated amygdaloid nucleus, main part	IM	26
intercalated nuclei of the amygdala	I	23–29
intercrural fissure	icf	49–54
interfascicular nucleus	IF	35–38
intermediate endopiriform nucleus	IEn	12–24
intermediate gray layer of the superior colliculus	InG	33–41
intermediate nucleus of the lateral lemniscus	INLL	40–42
intermediate reticular nucleus	IRt	46–62
intermediate white layer of the superior colliculus	InWh	33–41
intermediodorsal thalamic nucleus	IMD	28–30
intermedioventral thalamic commissure	imvc	29–30
internal capsule	ic	23–30
internal medullary lamina	iml	26
internal plexiform layer of the olfactory bulb	IPl	1–10
interpeduncular fossa	IPF	36
interpeduncular nucleus, caudal subnucleus	IPC	37–40
interpeduncular nucleus, dorsal subnucleus	IPD	38
interpeduncular nucleus, intermediate subnucleus	IPI	38–39
interpeduncular nucleus, lateral subnucleus	IPL	37–39
interpeduncular nucleus, rostral subnucleus	IPR	37–38
interposed cerebellar nucleus, anterior part	IntA	47–49
interposed cerebellar nucleus, dorsolateral hump	IntDL	47–50
interposed cerebellar nucleus, dorsomedial crest	IntDM	48–50
interposed cerebellar nucleus, posterior part	IntP	49–50
interposed cerebellar nucleus, posterior parvicellular part	IntPPC	49–50
interstitial nucleus of Cajal	InC	33–38
interstitial nucleus of the medulla	IB	57–62
interstitial nucleus of the posterior limb of the anterior commissure	IPAC	22–25
interventricular foramen	IVF	25–
islands of Calleja	ICj	16–22
islands of Calleja, major island	ICjM	20–21
isthmic reticular formation	isRt	39–42
**K**		
Kölliker–Fuse nucleus	KF	43–44
**L**		
lacunosum moleculare layer of the hippocampus	LMol	29–36
lambdoid septal zone	Ld	19–21
lateral accumbens shell	LAcbSh	19–21
lateral amygdaloid nucleus	La	26–31
lateral amygdaloid nucleus, dorsal part	LaD	27–30
lateral amygdaloid nucleus, ventral part	LaV	27–30
lateral (dentate) cerebellar nucleus	Lat	46–49
lateral cerebellar nucleus, parvicellular part	LatPC	46–48
lateral entorhinal cortex	LEnt	29–41
lateral habenular nucleus	LHb	28–31
lateral habenular nucleus, lateral part	LHbL	29–30
lateral habenular nucleus, medial part	LHbM	29–30
lateral lemniscus	ll	39–43
lateral mammillary nucleus	LM	33–35
lateral nucleus of the trapezoid body	LNTB	44–47
lateral olfactory tract	lo	5–25
lateral orbital cortex	LO	8–16
lateral parabrachial nucleus	LPB	45
lateral parabrachial nucleus, central part	LPBC	43–44
lateral parabrachial nucleus, crescent part	LPBCr	44
lateral parabrachial nucleus, internal part	LPBI	43–45
lateral paragigantocellular nucleus	LPGi	51–53
lateral paragigantocellular nucleus, alpha part	LPGiA	48–50
lateral paragigantocellular nucleus, external part	LPGiE	48–50
lateral parietal association cortex	LPtA	28–30
lateral periaqueductal gray	LPAG	35–42
lateral posterior thalamic nucleus, laterocaudal part	LPLC	32–33
lateral posterior thalamic nucleus, laterorostral part	LPLR	29–31
lateral posterior thalamic nucleus, mediocaudal part	LPMC	32–34
lateral posterior thalamic nucleus, mediorostral part	LPMR	29–32
lateral preoptic area	LPO	22–25
lateral recess of the 4th ventricle	LR4V	45–51
lateral reticular nucleus	LRt	54–59
lateral reticular nucleus, parvicellular part	LRtPC	56–58
lateral reticular nucleus, subtrigeminal part	LRtS5	55–56
lateral septal nucleus, dorsal part	LSD	19–25
lateral septal nucleus, intermediate part	LSI	18–23
lateral septal nucleus, ventral part	LSV	19–23
lateral stripe of the striatum	LSS	21–23
lateral superior olive	LSO	45–47
lateral terminal nucleus of the accessory optic tract	LT	33
lateral ventricle	LV	17–32
lateral vestibular nucleus	LVe	47–49
lateroanterior hypothalamic nucleus	LA	25–26
laterodorsal tegmental nucleus	LDTg	42–44
laterodorsal tegmental nucleus, ventral part	LDTgV	43
laterodorsal thalamic nucleus, dorsomedial part	LDDM	27–28
laterodorsal thalamic nucleus, ventrolateral part	LDVL	27–29
lateroventral periolivary nucleus	LVPO	44–47
layer 1 of cortex	1	11–33
layer 2 of cortex	2	11–33
layer 3 of cortex	3	11–33
layer 4 of cortex	4	14–16
lemina terminalis	LTer	24
linear nucleus of the medulla	Li	51–53
lithoid nucleus	Lth	33
locus coeruleus	LC	44–45
longitudinal fasciculus of the pons	lfp	40–43
**M**		
magnocellular nucleus of the lateral hypothalamus	MCLH	29
magnocellular nucleus of the posterior commissure	MCPC	33–34
magnocellular preoptic nucleus	MCPO	23–25
mammillary peduncle	mp	35–36
mammillary recess of the 3rd ventricle	MRe	34
mammillotegmental tract	mtg	33
mammillothalamic tract	mt	27–33
marginal zone of the medial geniculate	MZMG	33–35
matrix region of the medulla	Mx	50–59
medial (fastigial) cerebellar nucleus	Med	47–50
medial accessory oculomotor nucleus	MA3	34–35
medial amygdaloid nucleus, anterodorsal part	MeAD	26–27
medial amygdaloid nucleus, anteroventral part	MeAV	27
medial amygdaloid nucleus, posterodorsal part	MePD	27–29
medial amygdaloid nucleus, posteroventral part	MePV	28–29
medial cerebellar nucleus, dorsolateral protuberance	MedDL	49–50
medial cerebellar nucleus, lateral part	MedL	49–50
medial corticohypothalamic tract	mch	25
medial entorhinal cortex	MEnt	34–41
medial forebrain bundle	mfb	19–32
medial geniculate nucleus, dorsal part	MGD	33–36
medial geniculate nucleus, medial part	MGM	33–36
medial geniculate nucleus, ventral part	MGV	33–36
medial habenular nucleus	MHb	27–31
medial lemniscus	ml	28–54
medial lemniscal decussation	mlx	56
medial longitudinal fasciculus	mlf	37–62
medial mammillary nucleus, lateral part	ML	34–36
medial mammillary nucleus, medial part	MM	34–35
medial mammillary nucleus, median part	MnM	34–
medial nucleus of the trapezoid body	MNTB	43–47
medial orbital cortex	MO	8–14
medial parabrachial nucleus	MPB	43–45
medial parabrachial nucleus, external part	MPBE	44
medial parietal association cortex	MPtA	28–30
medial preoptic area	MPA	22–25
medial preoptic nucleus	MPO	24–25
medial pretectal nucleus	MPT	32
medial septal nucleus	MS	19–23
medial superior olive	MSO	44–47
medial terminal nucleus of the accessory optic tract	MT	35
medial tuberal nucleus	MTu	29–31
medial vestibular nucleus	MVe	53
medial vestibular nucleus, magnocellular part	MVeMC	46–52
medial vestibular nucleus, parvicellular part	MVePC	46–52
median accessory nucleus of the medulla	MnA	59–62
median eminence	ME	30–32
median preoptic nucleus	MnPO	22–24
median raphe nucleus	MnR	39–43
mediodorsal thalamic nucleus	MD	27
mediodorsal thalamic nucleus, central part	MDC	28–30
mediodorsal thalamic nucleus, lateral part	MDL	28–30
mediodorsal thalamic nucleus, medial part	MDM	28–30
medioventral periolivary nucleus	MVPO	44–47
medullary reticular nucleus, dorsal part	MdD	55–62
medullary reticular nucleus, ventral part	MdV	55–62
mesencehalic reticular formation	mRt	36–38
mesencephalic trigeminal nucleus	Me5	38–45
mesencephalic trigeminal tract	me5	43–45
microcellular tegmental nucleus	MiTg	38–39
middle cerebellar peduncle	mcp	40–46
middle cerebral artery	mcer	23
mitral cell layer of the accessory olfactory bulb	MiA	5–9
mitral cell layer of the olfactory bulb	Mi	1–10
molecular layer of the dentate gyrus	MoDG	27–37
molecular layer of the subiculum	MoS	37
motor root of the trigeminal nerve	m5	43–45
motor trigeminal nucleus	5N	44–45
motor trigeminal nucleus, anterior digastric part	5ADi	45–46
motor trigeminal nucleus, tensor tympani part	5TT	43–44
**N**		
navicular nucleus of the basal forebrain	Nv	17–18
nigrostriatal bundle	ns	27–33
nucleus of Darkschewitsch	Dk	33–35
nucleus of origin of efferents of the vestibular nerve	EVe	47
nucleus of Roller	Ro	52–56
nucleus of the brachium of the inferior colliculus	BIC	36–37
nucleus of the central acoustic tract	CAT	43–
nucleus of the fields of Forel	F	32–34
nucleus of the horizontal limb of the diagonal band	HDB	20–25
nucleus of the lateral olfactory tract	LOT	25–26
nucleus of the optic tract	OT	32–35
nucleus of the posterior commissure	PCom	33–34
nucleus of the solitary tract	Sol	49–50
nucleus of the solitary tract, commissural part	SolC	55–62
nucleus of the solitary tract, medial part	SolM	55–59
nucleus of the solitary tract, ventrolateral part	SolVL	55–57
nucleus of the vertical limb of the diagonal band	VDB	19–21
nucleus X	X	48–52
nucleus Y	Y	48
**O**		
obex	Obex	57
octopus cell area, ventral cochlear nucleus	Oca	47–49
oculomotor nerve	3n	36–37
oculomotor nucleus	3N	37–38
oculomotor nucleus, parvicellular part	3PC	36
olfactory nerve layer	ON	1–8
olfactory tubercle	Tu	16–23
olfactory ventricle (olfactory part of lateral ventricle)	OV	1–16
olivary pretectal nucleus	OPT	32–33
olivocerebellar tract	oc	50–54
olivocochlear bundle	ocb	46–47
optic chiasm	och	21–25
optic nerve layer of the superior colliculus	Op	33–40
optic tract	opt	26–33
oriens layer of the hippocampus	Or	27–36
oval paracentral thalamic nucleus	OPC	29–31
**P**		
p1 periaqueductal gray	p1PAG	32–34
p1 reticular formation	p1Rt	32–35
paraabducens nucleus	Pa6	46–47
parabigeminal nucleus	PBG	38–40
parabrachial pigmented nucleus of the ventral tegmental area	PBP	33–37
paracentral thalamic nucleus	PC	27–30
paracochlear glial substance	PCGS	46
parafascicular thalamic nucleus	PF	31–32
parafloccular sulcus	pfs	44–50
paraflocculus	PFl	43–50
parainterfascicular nucleus of the ventral tegmental area	PIF	36–37
paralemniscal nucleus	PL	40–42
paralemniscal nucleus, medial part	MPL	41–42
paramedian lobule	PM	49–56
paramedian raphe nucleus	PMnR	39–42
paramedian sulcus	pms	50–55
paranigral nucleus of the ventral tegmental area	PN	36–37
parapyramidal nucleus	PPy	49–50
pararubral nucleus	PaR	35–38
parasolitary nucleus	PSol	54–55
parastrial nucleus	PS	23–24
parasubiculum	PaS	35–42
parasubthalamic nucleus	PSTh	32
paratenial thalamic nucleus	PT	25–27
paraterete nucleus	PTe	29–30
paratrigeminal nucleus	Pa5	54–57
paratrochlear nucleus	Pa4	40–41
paraventricular hypothalamic nucleus, anterior parvicellular part	PaAP	25–26
paraventricular hypothalamic nucleus, medial magnocellular part	PaMM	27–28
paraventricular hypothalamic nucleus, medial parvicellular part	PaMP	27
paraventricular hypothalamic nucleus, posterior part	PaPo	28
paraventricular hypothalamic nucleus, ventral part	PaV	27
paraventricular thalamic nucleus	PV	27–28
paraventricular thalamic nucleus, anterior part	PVA	25–26
paraventricular thalamic nucleus, posterior part	PVP	29–31
paraxiphoid nucleus of thalamus	PaXi	27–29
parietal cortex, posterior area	PtP	28–31
parvicellular reticular nucleus	PCRt	46–54
peduncular part of lateral hypothalamus	PLH	26–33
pedunculopontine tegmental nucleus	PTg	38–42
pericollicular tegmental area	Pta	39–43
perifacial zone	P7	48–51
perifornical nucleus	PeF	29–30
perifornical part of lateral hypothalamus	PeFLH	29–32
perilemniscal nucleus, ventral part	PLV	41–43
peripeduncular nucleus	PP	33–35
perirhinal cortex	PRh	28–42
peritrigeminal zone	P5	43–46
periventricular hypothalamic nucleus	Pe	23–30
piriform cortex	Pir	11–33
polymorph layer of the dentate gyrus	PoDG	28–35
pontine nuclei	Pn	39–42
pontine raphe nucleus	PnR	43
pontine reticular nucleus, caudal part	PnC	44–47
pontine reticular nucleus, oral part	PnO	39–43
pontine reticular nucleus, ventral part	PnV	45–47
posterior commissure	pc	32–34
posterior hypothalamic area	PHA	33
posterior hypothalamic area, dorsal part	PHD	30–31
posterior hypothalamic nucleus	PH	31–32
posterior intralaminar thalamic nucleus	PIL	33–35
posterior limitans thalamic nucleus	PLi	33–35
posterior pretectal nucleus	PPT	33–35
posterior superior fissure	psf	43–54
posterior thalamic nuclear group	Po	28–33
posterior thalamic nuclear group, triangular part	PoT	33–35
posterodorsal raphe nucleus	PDR	39–42
posterodorsal tegmental nucleus	PDTg	45
posterolateral cortical amygdaloid nucleus	PLCo	27–29
posterolateral fissure	plf	43–55
posteromedial cortical amygdaloid nucleus	PMCo	29–33
posteromedian thalamic nucleus	PoMn	31
postsubiculum	Post	35–40
pre-Edinger–Westphal nucleus	PrEW	34–35
precentral fissure	pcn	43–46
precommissural nucleus	PrC	31–33
preculminate fissure	pcuf	43–47
precuneiform area	PrCnF	38–41
prelimbic cortex	PrL	9–18
premammillary nucleus, dorsal part	PMD	33
premammillary nucleus, ventral part	PMV	32–33
prepositus nucleus	Pr	47–53
prepyramidal fissure	ppf	51–56
prerubral field	PR	31–34
presubiculum	PrS	35–38
primary auditory cortex	Au1	28–33
primary auditory field	A1	29–33
primary fissure	prf	43–50
primary motor cortex	M1	11–28
primary somatosensory cortex	S1	24–30
primary somatosensory cortex, barrel field	S1BF	20–28
primary somatosensory cortex, dysgranular zone	S1DZ	16–28
primary somatosensory cortex, forelimb region	S1FL	15–24
primary somatosensory cortex, hindlimb region	S1HL	20–26
primary somatosensory cortex, jaw region	S1J	13–20
primary somatosensory cortex, oral dysgranular zone	S1DZO	18–19
primary somatosensory cortex, shoulder region	S1Sh	25–26
primary somatosensory cortex, trunk region	S1Tr	27–28
primary somatosensory cortex, upper lip region	S1ULp	18–28
primary visual cortex	V1	31–42
primary visual cortex, binocular area	V1B	32–39
primary visual cortex, monocular area	V1M	32–39
principal mammillary tract	pm	33–34
principal sensory trigeminal nucleus, dorsomedial part	Pr5DM	44–47
principal sensory trigeminal nucleus, ventrolateral part	Pr5VL	43–47
pyramidal cell layer of the hippocampus	Py	27–36
pyramidal decussation	pyx	57–62
pyramidal tract	py	43–58
**R**		
radiatum layer of the hippocampus	Rad	28–36
raphe interpositus nucleus	RIP	46–48
raphe magnus nucleus	RMg	44–51
raphe obscurus nucleus	ROb	50–58
raphe pallidus nucleus	RPa	43–58
red nucleus, magnocellular part	RMC	35–38
red nucleus, parvicellular part	RPC	35–36
reticluostrial nucleus	RtSt	26
reticular thalamic nucleus	Rt	26–30
reticulotegmental nucleus of the pons	RtTg	41–45
reticulotegmental nucleus of the pons, pericentral part	RtTgP	41–42
retroambiguus nucleus	RAmb	56–58
retrochiasmatic area	RCh	27–28
retrochiasmatic area, lateral part	RChL	27
retroethmoid nucleus	REth	33
retrorubral field	RRF	37–39
retrorubral nucleus	RR	39–40
retrosplenial dysgranular cortex	RSD	27–42
retrosplenial granular cortex	RSG	40
retrosplenial granular cortex, a region	RSGa	35–39
retrosplenial granular cortex, b region	RSGb	32–39
retrosplenial granular cortex, c region	RSGc	27–36
retrouniens area	RRe	31
reuniens thalamic nucleus	Re	26–30
rhabdoid nucleus	Rbd	39
rhinal fissure	rf	7–41
rhinal incisure	ri	7–12
rhomboid thalamic nucleus	Rh	27–30
rostral amygdalopiriform area	RAPir	28–30
rostral interstitial nucleus of medial longitudinal fasciculus	RI	32–33
rostral linear nucleus of the raphe	RLi	35–35
rostral periolivary nucleus	RPO	44
rostral ventral respiratory group	RVRG	53
rubrospinal tract	rs	41–62
**S**		
sagulum nucleus	Sag	41–43
scaphoid thalamic nucleus	Sc	32
secondary auditory cortex, dorsal area	AuD	28–36
secondary auditory cortex, ventral area	AuV	28–36
secondary fissure	sf	53–58
secondary motor cortex	M2	10–28
secondary somatosensory cortex	S2	18–28
secondary visual cortex, lateral area	V2L	31–41
secondary visual cortex, medial area	V2M	31–40
sensory root of the trigeminal nerve	s5	42–47
septofimbrial nucleus	SFi	22–25
septohippocampal nucleus	SHi	18–25
simple lobule	Sim	43–49
simplex fissure	simf	47–49
solitary nucleus, dorsolateral part	SolDL	55–57
solitary nucleus, ventral part	SolV	55–57
solitary tract	sol	50–57
spherical cell area, ventral cochlear nucleus	Sca	43–46
spinal trigeminal nucleus, caudal part	Sp5C	55–62
spinal trigeminal nucleus, interpolar part	Sp5I	49–56
spinal trigeminal nucleus, oral part	Sp5O	48–51
spinal trigeminal tract	sp5	48–62
spinal vestibular nucleus	SpVe	48–53
splenium of the corpus callosum	scc	31–33
stratum lucidum of the hippocampus	SLu	28–33
stria medullaris of the thalamus	sm	25–30
stria terminalis	st	23–31
strial part of the preoptic area	StA	23
subbrachial nucleus	SubB	36–38
subcoeruleus nucleus, alpha part	SubCA	44
subcoeruleus nucleus, dorsal part	SubCD	43–45
subcoeruleus nucleus, ventral part	SubCV	43–45
subcommissural organ	SCO	32–34
subfornical organ	SFO	25
subgeniculate nucleus	SubG	31–32
subiculum, transition area	STr	37–38
subincertal nucleus	SubI	29–30
sublenticular extended amygdala	EA	26
sublenticular extended amygdala, central part	EAC	25
submedius thalamic nucleus	Sub	30
submedius thalamic nucleus, dorsal part	SubD	28–29
submedius thalamic nucleus, ventral part	SubV	28–29
subparafascicular thalamic nucleus	SPF	31
subparafascicular thalamic nucleus, parvicellular part	SPFPC	31–32
subparaventricular zone of the hypothalamus	SPa	27–28
subpeduncular tegmental nucleus	SPTg	40–42
substantia innominata, basal part	SIB	21–24
substantia nigra, compact part	SNC	33–38
substantia nigra, reticular part	SNR	33–38
subthalamic nucleus	STh	30–32
superficial gray layer of the superior colliculus	SuG	33–41
superior cerebellar peduncle	scp	41–49
superior medullary velum	SMV	45–47
superior periolivary nucleus	SPN	44–47
superior thalamic radiation	str	31–32
superior vestibular nucleus	SuVe	46–47
suprachiasmatic nucleus	SCh	25
suprachiasmatic nucleus, dorsolateral part	SChDL	26
suprachiasmatic nucleus, ventromedial part	SChVM	26
suprageniculate thalamic nucleus	SG	33–36
supragenual nucleus	SGe	46
supramammillary decussation	sumx	34
supramammillary nucleus, lateral part	SuML	33–34
supramammillary nucleus, medial part	SuMM	33–34
supraoculomotor cap	Su3C	36–36
supraoculomotor periaqueductal gray	Su3	36–39
supraoptic decussation	sox	26–32
supraoptic nucleus	SO	23–27
supratrigeminal nucleus	Su5	43–45
**T**		
tectal gray	TG	33–35
tectospinal tract	ts	39–62
temporal association cortex	TeA	37–39
terete hypothalamic nucleus	Te	31
transverse fibers of the pons	tfp	39
trapezoid body	tz	43–49
triangular septal nucleus	TS	24–25
trigeminal transition zone	5Tr	44–46
trigeminal-solitary transition zone	5Sol	48–55
trigeminothalamic tract	tth	43–45
trochlear nerve	4n	41–44
trochlear nucleus	4N	39–40
trochlear nuclues shell region	4Sh	39–40
tuberal region of lateral hypothalamus	TuLH	27–31
**U**		
uvular fissure	uf	56–59
**V**		
vagus nerve	10n	52
ventral anterior thalamic nucleus	VA	27
ventral cochlear nucleus, anterior part	VCA	43–47
ventral cochlear nucleus, posterior part	VCP	47–49
ventral endopiriform nucleus	VEn	24–29
ventral geniculate nucleus	VG	29–34
ventral geniculate nucleus, magnocellular part	VGMC	31–32
ventral geniculate nucleus, parvicellular part	VGPC	31–32
ventral hippocampal commissure	vhc	25–27
ventral linear nucleus of the thalamus	VLi	33
ventral nucleus of the lateral lemniscus	VNLL	40
ventral nucleus of the lateral lemniscus, dorsal part	dVNLL	41–42
ventral nucleus of the lateral lemniscus, ventral part	vVNLL	41–43
ventral nucleus of the trapezoid body	VNTB	43–47
ventral orbital cortex	VO	9–16
ventral pallidum	VP	16–25
ventral part of claustrum	VCl	16–26
ventral posterior nucleus of the thalamus, parvicellular part	VPPC	31
ventral posterolateral thalamic nucleus	VPL	28–31
ventral posteromedial thalamic nucleus	VPM	28–32
ventral reuniens thalamic nucleus	VRe	27–30
ventral spinocerebellar tract	vsc	43–62
ventral subiculum	VS	31–36
ventral tegmental area	VTA	37–38
ventral tegmental area, rostral part	VTAR	34–35
ventral tegmental decussation	vtgx	35–37
ventral tegmental nucleus	VTg	42
ventral tenia tecta	VTT	11–16
ventral tuberomammillary nucleus	VTM	33–34
ventrolateral hypothalamic nucleus	VLH	27
ventrolateral hypothalamic tract	vlh	27
ventrolateral periaqueductal gray	VLPAG	37–42
ventrolateral preoptic nucleus	VLPO	23–24
ventrolateral thalamic nucleus	VL	27–30
ventromedial hypothalamic nucleus	VMH	28–31
ventromedial hypothalamic nucleus, central part	VMHC	29–30
ventromedial hypothalamic nucleus, dorsomedial part	VMHDM	29–30
venalamic nucleus, ventrolateral part	VMHVL	29–30
ventromedial nucleus of the hypothalamus shell	VMHSh	28–31
ventromedial preoptic nucleus	VMPO	23–24
ventromedial thalamic nucleus	VM	27–30
vestibulocerebellar nucleus	VeCb	46–49
vestibulocochlear nerve	8n	46–48
vestibulomesencephalic tract	veme	46–48
vestibulospinal tract	vesp	49
X		
xiphoid thalamic nucleus	Xi	27–28
**Z**		
zona incerta	ZI	28–29
zona incerta, caudal part	ZIC	34–34
zona incerta, dorsal part	ZID	30–33
zona incerta, rostral part	ZIR	26–27
zona incerta, ventral part	ZIV	30–33
zonal layer of the superior colliculus	Zo	33–41

**Index of abbreviations**

The abbreviations are listed in alphabetical order followed by the name of the structure and the plate number(s) of occurrence

**Table:** 

1	layer 1 of cortex	11–33	
2	layer 2 of cortex	11–33	
3	layer 3 of cortex	11–33	
4	layer 4 of cortex	14–16	
1Cb	1st cerebellar lobule (lingula)	46–48	
2Cb	2nd cerebellar lobule	43–46	
3Cb	3rd cerebellar lobule	43–49	
3n	oculomotor nerve	36–37	
3N	oculomotor nucleus	37–38	
3PC	oculomotor nucleus, parvicellular part	36	
3V	3rd ventricle	23–34	
4Cb	4th cerebellar lobule	41–49	
4n	trochlear nerve	41–44	
4N	trochlear nucleus	39–40	
4Sh	trochlear nuclues shell region	39–40	
4V	4th ventricle	43–54	
5ADi	motor trigeminal nucleus, anterior digastric part	45–46	
5Cb	5th cerebellar lobule	42–50	
5N	motor trigeminal nucleus	44–45	
5Sol	trigeminal-solitary transition zone	48–55	
5Tr	trigeminal transition zone	44–46	
5TT	motor trigeminal nucleus, tensor tympani part	43–44	
6Cb	6th cerebellar lobule	46–54	
6n	abducens nerve	46–47	
6N	abducens nucleus	47	
7Cb	7th cerebellar lobule	51–56	
7DI	facial nucleus, dorsal intermediate subnucleus	48–50	
7DL	facial nucleus, dorsolateral subnucleus	48–50	
7DM	facial nucleus, dorsomedial subnucleus	48–50	
7L	facial nucleus, lateral subnucleus	49–51	
7n	facial nerve	45–47	
7VI	facial nucleus, ventral intermediate subnucleus	48–50	
7VM	facial nucleus, ventromedial subnucleus	48–50	
8Cb	8th cerebellar lobule	51–58	
8n	vestibulocochlear nerve	46–48	
9aCb	9th cerebellar lobule, a	52–59	
9bCb	9th cerebellar lobule, b	52–59	
9Cb	9th cerebellar lobule	50–51	
9cCb	9th cerebellar lobule, c	52–59	
9N	glossopharyngeal nerve nucleus	62	
10Cb	10th cerebellar lobule (nodule)	50–55	
10N	dorsal motor nucleus of vagus	53–58	
10n	vagus nerve	52	
11N	accessory nerve nucleus	60–62	
12GH	hypoglossal nucleus, geniohyoid part	57–58	
12n	hypoglossal nerve	53–58	
12N	hypoglossal nucleus	51–58	
**A**			
A1	primary auditory field	29–33	
A11	A11 dopamine cells	30–31	
A13	A13 dopamine cells	28–29	
A5	A5 noradrenaline cells	44–47	
AA	anterior amygdaloid area	24–26	
AAF	anterior auditory field	28–29	
aca	anterior commissure, anterior part	11–24	
AcbC	accumbens nucleus, core	16–21	
AcbSh	accumbens nucleus, shell	16–21	
acer	anterior cerebral artery	23	
aci	anterior commissure, intrabulbar part	1–10	
ACo	anterior cortical amygdaloid nucleus	24–28	
acp	anterior commissure, posterior part	23–25	
AD	anterodorsal thalamic nucleus	26–27	
af	amygdaloid fissure	31–32	
AHA	anterior hypothalamic area, anterior part	25–26	
AHC	anterior hypothalamic area, central part	27–28	
AHi	amygdalohippocampal area	29–33	
AHP	anterior hypothalamic area, posterior part	28	
AI	agranular insular cortex	11–27	
ALPO	anterolateral periolivary nucleus	44	
alv	alveus of the hippocampus	27–38	
AM	anteromedial thalamic nucleus	25–28	
AmbC	ambiguus nucleus, compact part	52	
AmbL	ambiguus nucleus, loose part	55	
AmbSC	ambiguus nucleus, subcompact part	53–54	
AMV	anteromedial thalamic nucleus, ventral part	27	
AngT	angular thalamic nucleus	28–28	
ANS	accessory neurosecretory nuclei	27–28	
AOD	anterior olfactory nucleus, dorsal part	8–12	
AOE	anterior olfactory nucleus, external part	6–10	
AOL	anterior olfactory nucleus, lateral part	6–12	
AOM	anterior olfactory nucleus, medial part	9–13	
AOP	anterior olfactory nucleus, posterior part	14–16	
AOV	anterior olfactory nucleus, ventral part	8–11	
AOVP	anterior olfactory nucleus, ventroposterior part	11–15	
AP	area postrema	55–56	
APir	amygdalopiriform transition area	30–35	
apmf	ansoparamedian fissure	52–55	
APT	anterior pretectal nucleus	35	
APTD	anterior pretectal nucleus, dorsal part	31–34	
APTV	anterior pretectal nucleus, ventral part	32–34	
Aq	aqueduct	35–42	
Arc	arcuate hypothalamic nucleus	27–33	
asc7	ascending fibers of the facial nerve	48	
ASt	amygdalostriatal transition area	26–30	
ATg	anterior tegmental nucleus	40–41	
Au1	primary auditory cortex	28–33	
AuD	secondary auditory cortex, dorsal area	28–36	
AuV	secondary auditory cortex, ventral area	28–36	
AV	anteroventral thalamic nucleus	25	
AVDM	anterovent thalamic nucleus, dorsomedial part	26–28	
AVPe	anteroventral periventricular nucleus	23	
AVVL	anteroventral thalamic nucleus, ventrolateral part	26–27	
**B**			
B	basal nucleus (Meynert)	24–29	
BAC	bed nucleus of the anterior commissure	24	
BAOT	bed nucleus of the accessory olfactory tract	27	
Bar	Barrington’s nucleus	43–44	
bic	brachium of the inferior colliculus	36–40	
BIC	nucleus of the brachium of the inferior colliculus	36–37	
BLA	basolateral amygdaloid nucleus, anterior part	25–29	
BLP	basolateral amygdaloid nucleus, posterior part	27–32	
BLV	basolateral amygdaloid nucleus, ventral part	25–27	
BMA	basomedial amygdaloid nucleus, anterior part	25–27	
BMP	basomedial amygdaloid nucleus, posterior part	28–31	
Bo	Bötzinger complex	52	
bsc	brachium of the superior colliculus	33–35	
BV	blood vessel	21	
**C**			
CA1	field CA1 of the hippocampus	28–36	
CA2	field CA2 of the hippocampus	28–32	
CA3	field CA3 of the hippocampus	27–34	
CAT	nucleus of the central acoustic tract	43	
CB	cell bridges of the ventral striatum	20–22	
cbw	cerebellar white matter	43–57	
CC	central canal	55–62	
cc	corpus callosum	20–30	
CeC	central amygdaloid nucleus, capsular part	26–	29
CeCv	central cervical nucleus of the spinal cord	56–62	
CeL	central amygdaloid nucleus, lateral division	27–28	
CeM	central amygdaloid nucleus, medial division	25–29	
CEnt	caudomedial entothinal cortex	35–41	
CG	central gray	43	
cg	cingulum	17–34	
Cg1	cingulate cortex, area 1	10–27	
Cg2	cingulate cortex, area 2	19–27	
CGA	central gray, alpha part	44–46	
CGB	central gray, beta part	44–45	
CGG	central gray, gamma part	46	
CGO	central gray, nucleus O	44–45	
CGPn	central gray of the pons	45	
chp	choroid plexus	24–54	
CIC	central nucleus of the inferior colliculus	39–42	
cic	commissure of the inferior colliculus	42–43	
CL	centrolateral thalamic nucleus	28–31	
Cl	claustrum	12–27	
CLi	caudal linear nucleus of the raphe	37–39	
cll	commissure of the lateral lemniscus	41–42	
CM	central medial thalamic nucleus	26–31	
CnF	cuneiform nucleus	41–43	
Cop	copula of the pyramis	49–57	
cp	cerebral peduncle	28–39	
CPO	caudal periolivary nucleus	48	
CPu	caudate putamen (striatum)	17–30	
Crus1	crus 1 of the ansiform lobule	43–54	
Crus2	crus 2 of the ansiform lobule	49–55	
csc	commissure of the superior colliculus34–36		
cst	commissural stria terminalis	26–27	
cu	cuneate fasciculus	53–62	
Cu	cuneate nucleus	52–62	
CuR	cuneate nucleus, rotundus part	55–56	
CVL	caudoventrolateral reticular nucleus	52–53	
CxA	cortex-amygdala transition zone	24–26	
**D**			
DA	dorsal hypothalamic area	29–30	
das	dorsal acoustic stria	49–50	
DCDp	dorsal cochlear nucleus, deep core	49–50	
DCFu	dorsal cochlear nucleus, fusiform layer	48–50	
DCIC	dorsal cortex of the inferior colliculus	40–43	
DCl	dorsal part of claustrum	16–26	
DCMo	dorsal cochlear nucleus, molecular layer	48–50	
dcs	dorsal corticospinal tract	60–62	
dcw	deep cerebral white matter	29–39	
DEn	dorsal endopiriform nucleus	12–32	
df	dorsal fornix	26–27	
DG	dentate gyrus	30	
dhc	dorsal hippocampal commissure	28–38	
DI	dysgranular insular cortex	13–27	
Dk	nucleus of Darkschewitsch	33–35	
DLG	dorsal lateral geniculate nucleus	29–33	
dlo	dorsal lateral olfactory tract	5–12	
DLO	dorsolateral orbital cortex	9–12	
DLPAG	dorsolateral periaqueductal gray	36–42	
DLPO	dorsolateral periolivary nucleus	44–47	
DM	dorsomedial hypothalamic nucleus	29–32	
DMC	dorsomedial hypothalamic nucleus, compact part	31	
DMD	dorsomedial hypothalamic nucleus, dorsal part	31	
DMIC	dorsomedial nucleus of the inferior colliculus	41–43	
DMPAG	dorsomedial periaqueductal gray	35–42	
DMSp5	dorsomedial spinal trigeminal nucleus	48–54	
DMTg	dorsomedial tegmental area	43–45	
DMV	dorsomedial hypothalamic nucleus, ventral part	31	
DNLL	dorsal nucleus of the lateral lemniscus	41–42	
DP	dorsal peduncular cortex	12–18	
DpG	deep gray layer of the superior colliculus	33–41	
DPGi	dorsal paragigantocellular nucleus	48–51	
DPO	dorsal periolivary nucleus	45–47	
DpWh	deep white layer of the superior colliculus	34–41	
DR	dorsal raphe nucleus	37–38	
DRC	dorsal raphe nucleus, caudal part	43–44	
DRD	dorsal raphe nucleus, dorsal part	39–42	
DRL	dorsal raphe nucleus, lateral part	39–41	
DRV	dorsal raphe nucleus, ventral part	39–42	
DS	dorsal subiculum	33–36	
dsc	dorsal spinocerebellar tract	52–62	
DTg	dorsal tegmental nucleus	44	
DTgC	dorsal tegmental nucleus, central part	43	
DTgP	dorsal tegmental nucleus, pericentral part	43	
dtgx	dorsal tegmental decussation	36–37	
Dtr	dorsal transition zone	12–15	
DTT	dorsal tenia tecta	11–18	
dVNLL	ventral nucleus of the lateral lemniscus, dorsal part	41–42	
**E**			
E	ependyma and subependymal layer	1–23	
EA	sublenticular extended amygdala	26	
EAC	sublenticular extended amygdala, central part	25	
ec	external capsule	19–30	
ECIC	external cortex of the inferior colliculus	38–44	
Ect	ectorhinal cortex	28–42	
ECu	external cuneate nucleus	52–56	
eml	external medullary lamina	28–30	
Ent	entorhinal cortex	42	
EP	entopeduncular nucleus	27–28	
EPl	external plexiform layer of the olfactory bulb	1–9	
EPlA	external plexiform layer of the accessory olfactory bulb	5–9	
Eth	ethmoid thalamic nucleus	32	
EVe	nucleus of origin of efferents of the vestibular nerve	47	
EW	Edinger–Westphal nucleus	36–38	
**F**			
f	fornix	23–32	
F	nucleus of the fields of Forel	32–34	
FC	fasciola cinereum	29–33	
fi	fimbria of the hippocampus	22–30	
Fl	flocculus	43–48	
fmi	forceps minor of the corpus callosum	13–18	
fmj	forceps major of the corpus callosum	34–39	
fr	fasciculus retroflexus	27–36	
Fr3	frontal cortex, area 3	11–17	
FrA	frontal association cortex	7–9	
Fu	bed nucleus of stria terminalis, fusiform part	23	
FVe	F cell group of the vestibular complex	52	
**G**			
g7	genu of the facial nerve	46–48	
Gca	globular cell area, ventral cochlear nucleus	46–49	
gcc	genu of the corpus callosum	19	
Ge5	gelatinous layer of the caudal spinal trigeminal nucleus	57–62	
Gi	gigantocellular reticular nucleus	47–54	
GI	granular insular cortex	13–27	
GiA	gigantocellular reticular nucleus, alpha part	48–51	
GiV	gigantocellular reticular nucleus, ventral part	51–52	
Gl	glomerular layer of the olfactory bulb	1–11	
GlA	glomerular layer of the accessory olfactory bulb	5–10	
GP	globus pallidus	24–29	
gr	gracile fasciculus	54–62	
Gr	gracile nucleus	54–62	
GrA	granule cell layer of the accessory olfactory bulb	3–10	
GrC	granule cell layer of cochlear nuclei	43–50	
GrDG	granular layer of the dentate gyrus	27–36	
GrO	granular cell layer of the olfactory bulb	1–10	
**H**			
hbc	habenular commissure	31–32	
HDB	nucleus of the horizontal limb of the diagonal band	20–25	
hif	hippocampal fissure	30–34	
**I**			
I	intercalated nuclei of the amygdala	23–29	
IAD	interanterodorsal thalamic nucleus	26–27	
IAM	interanteromedial thalamic nucleus	27	
IB	interstitial nucleus of the medulla	57–62	
ic	internal capsule	23–30	
icf	intercrural fissure	49–54	
ICj	islands of Calleja	16–22	
ICjM	islands of Calleja, major island	20–21	
icp	inferior cerebellar peduncle (restiform body)	46–53	
IEn	intermediate endopiriform nucleus	12–24	
IF	interfascicular nucleus	35–38	
IG	indusium griseum	18–33	
IL	infralimbic cortex	14–18	
IM	intercalated amygdaloid nucleus, main part	26	
IMD	intermediodorsal thalamic nucleus	28–30	
IMG	amygdaloid intramedullary gray	27–28	
iml	internal medullary lamina	26	
imvc	intermedioventral thalamic commissure	29–30	
InC	interstitial nucleus of Cajal	33–38	
InG	intermediate gray layer of the superior colliculus	33–41	
INLL	intermediate nucleus of the lateral lemniscus	40–42	
IntA	interposed cerebellar nucleus, anterior part	47–49	
IntDL	interposed cerebellar nucleus, dorsolateral hump	47–50	
IntDM	interposed cerebellar nucleus, dorsomedial crest	48–50	
IntP	interposed cerebellar nucleus, posterior part	49–50	
IntPPC	interposed cerebellar nucleus, posterior parvicellular part	49–50	
InWh	intermediate white layer of the superior colliculus	33–41	
IOA	inferior olive, subnucleus A of medial nucleus	57–58	
IOB	inferior olive, subnucleus B of medial nucleus	56–58	
IOBe	inferior olive, beta subnucleus	56–58	
IOC	inferior olive, subnucleus C of medial nucleus	56–58	
IOD	inferior olive, dorsal nucleus	52–56	
IODM	inferior olive, dorsomedial cell group	52–55	
IOK	inferior olive, cap of Kooy of the medial nucleus	56–57	
IOM	inferior olive, medial nucleus	52–55	
IOPr	inferior olive, principal nucleus	52–56	
IPAC	interstitial nucleus of the posterior limb of the anterior commissure	22–25	
IPC	interpeduncular nucleus, caudal subnucleus	37–40	
IPD	interpeduncular nucleus, dorsal subnucleus	38	
IPF	interpeduncular fossa	36	
IPI	interpeduncular nucleus, intermediate subnucleus	38–39	
IPl	internal plexiform layer of the olfactory bulb	1–10	
IPL	interpeduncular nucleus, lateral subnucleus	37–39	
IPR	interpeduncular nucleus, rostral subnucleus	37–38	
IRt	intermediate reticular nucleus	46–62	
isRt	isthmic reticular formation	39–42	
IVF	interventricular foramen	25	
**K**			
KF	Kölliker–Fuse nucleus	43–44	
**L**			
La	lateral amygdaloid nucleus	26–31	
LA	lateroanterior hypothalamic nucleus	25–26	
LAcbSh	lateral accumbens shell	19–21	
LaD	lateral amygdaloid nucleus, dorsal part	27–30	
Lat	lateral (dentate) cerebellar nucleus	46–49	
LatPC	lateral cerebellar nucleus, parvicellular part	46–48	
LaV	lateral amygdaloid nucleus, ventral part	27–30	
LC	locus coeruleus	44–45	
Ld	lambdoid septal zone	19–21	
LDDM	laterodorsal thalamic nucleus, dorsomedial part	27–28	
LDTg	laterodorsal tegmental nucleus	42–44	
LDTgV	laterodorsal tegmental nucleus, ventral part	43	
LDVL	laterodorsal thalamic nucleus, ventrolateral part	27–29	
LEnt	lateral entorhinal cortex	29–41	
lfp	longitudinal fasciculus of the pons	40–43	
LHb	lateral habenular nucleus	28–31	
LHbL	lateral habenular nucleus, lateral part	29–30	
LHbM	lateral habenular nucleus, medial part	29–30	
Li	linear nucleus of the medulla	51–53	
ll	lateral lemniscus	39–43	
LM	lateral mammillary nucleus	33–35	
LMol	lacunosum moleculare layer of the hippocampus	29–36	
LNTB	lateral nucleus of the trapezoid body	44–47	
lo	lateral olfactory tract	5–25	
LO	lateral orbital cortex	8–16	
LOT	nucleus of the lateral olfactory tract	25–26	
LPAG	lateral periaqueductal gray	35–42	
LPB	lateral parabrachial nucleus	45	
LPBC	lateral parabrachial nucleus, central part	43–44	
LPBCr	lateral parabrachial nucleus, crescent part	44	
LPBI	lateral parabrachial nucleus, internal part	43–45	
LPGi	lateral paragigantocellular nucleus	51–53	
LPGiA	lateral paragigantocellular nucleus, alpha part	48–50	
LPGiE	lateral paragigantocellular nucleus, external part	48–50	
LPLC	lateral posterior thalamic nucleus, laterocaudal part	32–33	
LPLR	lateral posterior thalamic nucleus, laterorostral part	29–31	
LPMC	lateral posterior thalamic nucleus, mediocaudal part	32–34	
LPMR	lateral posterior thalamic nucleus, mediorostral part	29–32	
LPO	lateral preoptic area	22–25	
LPtA	lateral parietal association cortex	28–30	
LR4V	lateral recess of the 4th ventricle	45–51	
LRt	lateral reticular nucleus	54–59	
LRtPC	lateral reticular nucleus, parvicellular part	56–58	
LRtS5	lateral reticular nucleus, subtrigeminal part	55–56	
LSD	lateral septal nucleus, dorsal part	19–25	
LSI	lateral septal nucleus, intermediate part	18–23	
LSO	lateral superior olive	45–47	
LSS	lateral stripe of the striatum	21–23	
LSV	lateral septal nucleus, ventral part	19–23	
LT	lateral terminal nucleus of the accessory optic tract	33	
LTer	lemina terminalis	24	
Lth	lithoid nucleus	33	
LV	lateral ventricle	17–32	
LVe	lateral vestibular nucleus	47–49	
LVPO	lateroventral periolivary nucleus	44–47	
**M**			
M1	primary motor cortex	11–28	
M2	secondary motor cortex	10–28	
m5	motor root of the trigeminal nerve	43–45	
MA3	medial accessory oculomotor nucleus	34–35	
mcer	middle cerebral artery	23	
mch	medial corticohypothalamic tract	25	
MCLH	magnocellular nucleus of the lateral hypothalamus	29	
mcp	middle cerebellar peduncle	40–46	
MCPC	magnocellular nucleus of the posterior commissure	33–34	
MCPO	magnocellular preoptic nucleus	23–25	
MD	mediodorsal thalamic nucleus	27	
MDC	mediodorsal thalamic nucleus, central part	28–30	
MdD	medullary reticular nucleus, dorsal part	55–62	
MDL	mediodorsal thalamic nucleus, lateral part	28–30	
MDM	mediodorsal thalamic nucleus, medial part	28–30	
MdV	medullary reticular nucleus, ventral part	55–62	
ME	median eminence	30–32	
Me5	mesencephalic trigeminal nucleus	38–45	
me5	mesencephalic trigeminal tract	43–45	
MeAD	medial amygdaloid nucleus, anterodorsal part	26–27	
MeAV	medial amygdaloid nucleus, anteroventral part	27	
Med	medial (fastigial) cerebellar nucleus	47–50	
MedDL	medial cerebellar nucleus, dorsolateral protuberance	49–50	
MedL	medial cerebellar nucleus, lateral part	49–50	
MEnt	medial entorhinal cortex	34–41	
MePD	medial amygdaloid nucleus, posterodorsal part	27–29	
MePV	medial amygdaloid nucleus, posteroventral part	28–29	
mfb	medial forebrain bundle	19–32	
MGD	medial geniculate nucleus, dorsal part	33–36	
MGM	medial geniculate nucleus, medial part	33–36	
MGV	medial geniculate nucleus, ventral part	33–36	
MHb	medial habenular nucleus	27–31	
Mi	mitral cell layer of the olfactory bulb	1–10	
MiA	mitral cell layer of the accessory olfactory bulb	5–9	
MiTg	microcellular tegmental nucleus	38–39	
ml	medial lemniscus	28–54	
ML	medial mammillary nucleus, lateral part	34–36	
mlf	medial longitudinal fasciculus	37–62	
mlx	medial lemniscal decussation	56	
MM	medial mammillary nucleus, medial part	34–35	
MnA	median accessory nucleus of the medulla	59–62	
MnM	medial mammillary nucleus, median part	34	
MnPO	median preoptic nucleus	22–24	
MnR	median raphe nucleus	39–43	
MNTB	medial nucleus of the trapezoid body	43–47	
MO	medial orbital cortex	8–14	
MoDG	molecular layer of the dentate gyrus	27–37	
MoS	molecular layer of the subiculum	37	
mp	mammillary peduncle	35–36	
MPA	medial preoptic area	22–25	
MPB	medial parabrachial nucleus	43–45	
MPBE	medial parabrachial nucleus, external part	44	
MPL	paralemniscal nucleus, medial part	41–42	
MPO	medial preoptic nucleus	24–25	
MPT	medial pretectal nucleus	32	
MPtA	medial parietal association cortex	28–30	
MRe	mammillary recess of the 3rd ventricle	34	
mRt	mesencehalic reticular formation	36–38	
MS	medial septal nucleus	19–23	
MSO	medial superior olive	44–47	
mt	mammillothalamic tract	27–33	
MT	medial terminal nucleus of the accessory optic tract	35	
mtg	mammillotegmental tract	33	
MTu	medial tuberal nucleus	29–31	
MVe	medial vestibular nucleus	53	
MVeMC	medial vestibular nucleus, magnocellular part	46–52	
MVePC	medial vestibular nucleus, parvicellular part	46–52	
MVPO	medioventral periolivary nucleus	44–47	
Mx	matrix region of the medulla	50–59	
MZMG	marginal zone of the medial geniculate	33–35	
**N**			
ns	nigrostriatal bundle	27–33	
Nv	navicular nucleus of the basal forebrain	17–18	
**O**			
Obex	obex	57	
oc	olivocerebellar tract	50–54	
Oca	octopus cell area, ventral cochlear nucleus	47–49	
ocb	olivocochlear bundle	46–47	
och	optic chiasm	21–25	
ON	olfactory nerve layer	1–8	
Op	optic nerve layer of the superior colliculus	33–40	
OPC	oval paracentral thalamic nucleus	29–31	
OPT	olivary pretectal nucleus	32–33	
opt	optic tract	26–33	
Or	oriens layer of the hippocampus	27–36	
OT	nucleus of the optic tract	32–35	
OV	olfactory ventricle (olfactory part of lateral ventricle)	1–16	
**P**			
p1PAG	p1 periaqueductal gray	32–34	
p1Rt	p1 reticular formation	32–35	
P5	peritrigeminal zone	43–46	
P7	perifacial zone	48–51	
Pa4	paratrochlear nucleus	40–41	
Pa5	paratrigeminal nucleus	54–57	
Pa6	paraabducens nucleus	46–47	
PaAP	paraventricular hypothalamic nucleus, anterior parvicellular part	25–26	
PaMM	paraventricular hypothalamic nucleus, medial magnocellular part	27–28	
PaMP	paraventricular hypothalamic nucleus, medial parvicellular part	27	
PaPo	paraventricular hypothalamic nucleus, posterior part	28	
PaR	pararubral nucleus	35–38	
PaS	parasubiculum	35–42	
PaV	paraventricular hypothalamic nucleus, ventral part	27	
PaXi	paraxiphoid nucleus of thalamus	27–29	
PBG	parabigeminal nucleus	38–40	
PBP	parabrachial pigmented nucleus of the ventral tegmental area	33–37	
PC	paracentral thalamic nucleus	27–30	
pc	posterior commissure	32–34	
PCGS	paracochlear glial substance	46	
pcn	precentral fissure	43–46	
PCom	nucleus of the posterior commissure	33–34	
PCRt	parvicellular reticular nucleus	46–54	
pcuf	preculminate fissure	43–47	
PDR	posterodorsal raphe nucleus	39–42	
PDTg	posterodorsal tegmental nucleus	45	
Pe	periventricular hypothalamic nucleus	23–30	
PeF	perifornical nucleus	29–30	
PeFLH	perifornical part of lateral hypothalamus	29–32	
PF	parafascicular thalamic nucleus	31–32	
PFl	paraflocculus	43–50	
pfs	parafloccular sulcus	44–50	
PH	posterior hypothalamic nucleus	31–32	
PHA	posterior hypothalamic area	33	
PHD	posterior hypothalamic area, dorsal part	30–31	
PIF	parainterfascicular nucleus of the ventral tegmental area	36–37	
PIL	posterior intralaminar thalamic nucleus	33–35	
Pir	piriform cortex	11–33	
PL	paralemniscal nucleus	40–42	
PLCo	posterolateral cortical amygdaloid nucleus	27–29	
plf	posterolateral fissure	43–55	
PLH	peduncular part of lateral hypothalamus	26–33	
PLi	posterior limitans thalamic nucleus	33–35	
PLV	perilemniscal nucleus, ventral part	41–43	
PM	paramedian lobule	49–56	
pm	principal mammillary tract	33–34	
PMCo	posteromedial cortical amygdaloid nucleus	29–33	
PMD	premammillary nucleus, dorsal part	33	
PMnR	paramedian raphe nucleus	39–42	
pms	paramedian sulcus	50–55	
PMV	premammillary nucleus, ventral part	32–33	
PN	paranigral nucleus of the ventral tegmental area	36–37	
Pn	pontine nuclei	39–42	
PnC	pontine reticular nucleus, caudal part	44–47	
PnO	pontine reticular nucleus, oral part	39–43	
PnR	pontine raphe nucleus	43	
PnV	pontine reticular nucleus, ventral part	45–47	
Po	posterior thalamic nuclear group	28–33	
PoDG	polymorph layer of the dentate gyrus	28–35	
PoMn	posteromedian thalamic nucleus	31	
Post	postsubiculum	35–40	
PoT	posterior thalamic nuclear group, triangular part	33–35	
PP	peripeduncular nucleus	33–35	
ppf	prepyramidal fissure	51–56	
PPT	posterior pretectal nucleus	33–35	
PPy	parapyramidal nucleus	49–50	
Pr	prepositus nucleus	47–53	
PR	prerubral field	31–34	
Pr5DM	principal sensory trigeminal nucleus, dorsomedial part	44–47	
Pr5VL	principal sensory trigeminal nucleus, ventrolateral part	43–47	
PrC	precommissural nucleus	31–33	
PrCnF	precuneiform area	38–41	
PrEW	pre-Edinger–Westphal nucleus	34–35	
prf	primary fissure	43–50	
PRh	perirhinal cortex	28–42	
PrL	prelimbic cortex	9–18	
PrS	presubiculum	35–38	
PS	parastrial nucleus	23–24	
psf	posterior superior fissure	43–54	
PSol	parasolitary nucleus	54–55	
PSTh	parasubthalamic nucleus	32	
PT	paratenial thalamic nucleus	25–27	
Pta	pericollicular tegmental area	39–43	
PTe	paraterete nucleus	29–30	
PTg	pedunculopontine tegmental nucleus	38–42	
PtP	parietal cortex, posterior area	28–31	
PV	paraventricular thalamic nucleus	27–28	
PVA	paraventricular thalamic nucleus, anterior part	25–26	
PVP	paraventricular thalamic nucleus, posterior part	29–31	
Py	pyramidal cell layer of the hippocampus	27–36	
py	pyramidal tract	43–58	
pyx	pyramidal decussation	57–62	
**R**			
Rad	radiatum layer of the hippocampus	28–36	
RAmb	retroambiguus nucleus	56–58	
RAPir	rostral amygdalopiriform area	28–30	
Rbd	rhabdoid nucleus	39	
RCh	retrochiasmatic area	27–28	
RChL	retrochiasmatic area, lateral part	27	
Re	reuniens thalamic nucleus	26–30	
REth	retroethmoid nucleus	33	
rf	rhinal fissure	7–41	
Rh	rhomboid thalamic nucleus	27–30	
ri	rhinal incisure	7–12	
RI	rostral interstitial nucleus of medial longitudinal fasciculus	32–33	
RIP	raphe interpositus nucleus	46–48	
RLi	rostral linear nucleus of the raphe	35–35	
RMC	red nucleus, magnocellular part	35–38	
RMg	raphe magnus nucleus	44–51	
Ro	nucleus of Roller	52–56	
ROb	raphe obscurus nucleus	50–58	
RPa	raphe pallidus nucleus	43–58	
RPC	red nucleus, parvicellular part	35–36	
RPO	rostral periolivary nucleus	44	
RR	retrorubral nucleus	39–40	
RRe	retrouniens area	31	
RRF	retrorubral field	37–39	
rs	rubrospinal tract	41–62	
RSD	retrosplenial dysgranular cortex	27–42	
RSG	retrosplenial granular cortex	40	
RSGa	retrosplenial granular cortex, a region	35–39	
RSGb	retrosplenial granular cortex, b region	32–39	
RSGc	retrosplenial granular cortex, c region	27–36	
Rt	reticular thalamic nucleus	26–30	
RtSt	reticluostrial nucleus	26	
RtTg	reticulotegmental nucleus of the pons	41–45	
RtTgP	reticulotegmental nucleus of the pons, pericentral part	41–42	
RVRG	rostral ventral respiratory group	53	
**S**			
S1	primary somatosensory cortex	24–30	
S1BF	primary somatosensory cortex, barrel field	20–28	
S1DZ	primary somatosensory cortex, dysgranular zone	16–28	
S1DZO	primary somatosensory cortex, oral dysgranular zone	18–19	
S1FL	primary somatosensory cortex, forelimb region	15–24	
S1HL	primary somatosensory cortex, hindlimb region	20–26	
S1J	primary somatosensory cortex, jaw region	13–20	
S1Sh	primary somatosensory cortex, shoulder region	25–26	
S1Tr	primary somatosensory cortex, trunk region	27–28	
S1ULp	primary somatosensory cortex, upper lip region	18–28	
S2	secondary somatosensory cortex	18–28	
s5	sensory root of the trigeminal nerve	42–47	
Sag	sagulum nucleus	41–43	
Sc	scaphoid thalamic nucleus	32	
Sca	spherical cell area, ventral cochlear nucleus	43–46	
scc	splenium of the corpus callosum	31–33	
SCh	suprachiasmatic nucleus	25	
SChDL	suprachiasmatic nucleus, dorsolateral part	26	
SChVM	suprachiasmatic nucleus, ventromedial part	26	
SCO	subcommissural organ	32–34	
scp	superior cerebellar peduncle	41–49	
sf	secondary fissure	53–58	
SFi	septofimbrial nucleus	22–25	
SFO	subfornical organ	25	
SG	suprageniculate thalamic nucleus	33–36	
SGe	supragenual nucleus	46	
SHi	septohippocampal nucleus	18–25	
SIB	substantia innominata, basal part	21–24	
Sim	simple lobule	43–49	
simf	simplex fissure	47–49	
SLu	stratum lucidum of the hippocampus	28–33	
sm	stria medullaris of the thalamus	25–30	
SMV	superior medullary velum	45–47	
SNC	substantia nigra, compact part	33–38	
SNR	substantia nigra, reticular part	33–38	
SO	supraoptic nucleus	23–27	
Sol	nucleus of the solitary tract	49–50	
sol	solitary tract	50–57	
SolC	nucleus of the solitary tract, commissural part	55–62	
SolDL	solitary nucleus, dorsolateral part	55–57	
SolM	nucleus of the solitary tract, medial part	55–59	
SolV	solitary nucleus, ventral part	55–57	
SolVL	nucleus of the solitary tract, ventrolateral part	55–57	
sox	supraoptic decussation	26–32	
sp5	spinal trigeminal tract	48–62	
Sp5C	spinal trigeminal nucleus, caudal part	55–62	
Sp5I	spinal trigeminal nucleus, interpolar part	49–56	
Sp5O	spinal trigeminal nucleus, oral part	48–51	
SPa	subparaventricular zone of the hypothalamus	27–28	
SPF	subparafascicular thalamic nucleus	31	
SPFPC	subparafascicular thalamic nucleus, parvicellular part	31–32	
SPN	superior periolivary nucleus	44–47	
SPTg	subpeduncular tegmental nucleus	40–42	
SpVe	spinal vestibular nucleus	48–53	
ST	bed nucleus of the stria terminalis	22	
st	stria terminalis	23–31	
StA	strial part of the preoptic area	23	
STh	subthalamic nucleus	30–32	
STIA	bed nucleus of the stria terminalis, intraamygdaloid division	28–29	
STLI	bed nucleus of the stria terminalis, lateral division, intermediate part	24	
STLP	bed nucleus of the stria terminalis, lateral division, posterior part	23–24	
STLV	bed nucleus of the stria terminalis, lateral division, ventral part	23–24	
STMA	bed nucleus of the stria terminalis, medial division, anterior part	23–24	
STMP	bed nucleus of the stria terminalis, medial division, posterior part	25–26	
STMV	bed nucleus of the stria terminalis, medial division, ventral part	23–24	
STr	subiculum, transition area	37–38	
str	superior thalamic radiation	31–32	
Su3	supraoculomotor periaqueductal gray	36–39	
Su3C	supraoculomotor cap	36–36	
Su5	supratrigeminal nucleus	43–45	
Sub	submedius thalamic nucleus	30	
SubB	subbrachial nucleus	36–38	
SubCA	subcoeruleus nucleus, alpha part	44	
SubCD	subcoeruleus nucleus, dorsal part	43–45	
SubCV	subcoeruleus nucleus, ventral part	43–45	
SubD	submedius thalamic nucleus, dorsal part	28–29	
SubG	subgeniculate nucleus	31–32	
SubI	subincertal nucleus	29–30	
SubV	submedius thalamic nucleus, ventral part	28–29	
SuG	superficial gray layer of the superior colliculus	33–41	
SuML	supramammillary nucleus, lateral part	33–34	
SuMM	supramammillary nucleus, medial part	33–34	
sumx	supramammillary decussation	34	
SuVe	superior vestibular nucleus	46–47	
**T**			
Te	terete hypothalamic nucleus	31	
TeA	temporal association cortex	37–39	
tfp	transverse fibers of the pons	39	
TG	tectal gray	33–35	
ts	tectospinal tract	39–62	
TS	triangular septal nucleus	24–25	
tth	trigeminothalamic tract	43–45	
Tu	olfactory tubercle	16–23	
TuLH	tuberal region of lateral hypothalamus	27–31	
tz	trapezoid body	43–49	
tzx	decussation of the trapezoid body	44–47	
**U**			
uf	uvular fissure	56–59	
**V**			
V1	primary visual cortex	31–42	
V1B	primary visual cortex, binocular area	32–39	
V1M	primary visual cortex, monocular area	32–39	
V2L	secondary visual cortex, lateral area	31–41	
V2M	secondary visual cortex, medial area	31–40	
VA	ventral anterior thalamic nucleus	27	
VCA	ventral cochlear nucleus, anterior part	43–47	
VCl	ventral part of claustrum	16–26	
VCP	ventral cochlear nucleus, posterior part	47–49	
VDB	nucleus of the vertical limb of the diagonal band	19–21	
VeCb	vestibulocerebellar nucleus	46–49	
veme	vestibulomesencephalic tract	46–48	
VEn	ventral endopiriform nucleus	24–29	
vesp	vestibulospinal tract	49	
VG	ventral geniculate nucleus	29–34	
VGMC	ventral geniculate nucleus, magnocellular part	31–32	
VGPC	ventral geniculate nucleus, parvicellular part	31–32	
vhc	ventral hippocampal commissure	25–27	
VL	ventrolateral thalamic nucleus	27–30	
VLH	ventrolateral hypothalamic nucleus	27	
vlh	ventrolateral hypothalamic tract	27	
VLi	ventral linear nucleus of the thalamus	33	
VLPAG	ventrolateral periaqueductal gray	37–42	
VLPO	ventrolateral preoptic nucleus	23–24	
VM	ventromedial thalamic nucleus	27–30	
VMH	ventromedial hypothalamic nucleus	28–31	
VMHC	ventromedial hypothalamic nucleus, central part	29–30	
VMHDM	ventromedial hypothalamic nucleus, dorsomedial part	29–30	
VMHSh	ventromedial nucleus of the hypothalamus shell	28–31	
VMHVL	ventromedial hypothalamic nucleus, ventrolateral part	29–30	
VMPO	ventromedial preoptic nucleus	23–24	
VNLL	ventral nucleus of the lateral lemniscus	40	
VNTB	ventral nucleus of the trapezoid body	43–47	
VO	ventral orbital cortex	9–16	
VP	ventral pallidum	16–25	
VPL	ventral posterolateral thalamic nucleus	28–31	
VPM	ventral posteromedial thalamic nucleus	28–32	
VPPC	ventral posterior nucleus of the thalamus, parvicellular part	31	
VRe	ventral reuniens thalamic nucleus	27–30	
VS	ventral subiculum	31–36	
vsc	ventral spinocerebellar tract	43–62	
VTA	ventral tegmental area	37–38	
VTAR	ventral tegmental area, rostral part	34–35	
VTg	ventral tegmental nucleus	42	
vtgx	ventral tegmental decussation	35–37	
VTM	ventral tuberomammillary nucleus	33–34	
VTT	ventral tenia tecta	11–16	
vVNLL	ventral nucleus of the lateral lemniscus, ventral part	41–43	
**X**			
X	nucleus X	48–52	
Xi	xiphoid thalamic nucleus	27–28	
xscp	decussation of the superior cerebellar peduncle	39–41	
**Y**			
Y	nucleus Y	48	
**Z**			
ZI	zona incerta	28–29	
ZIC	zona incerta, caudal part	34–34	
ZID	zona incerta, dorsal part	30–33	
ZIR	zona incerta, rostral part	26–27	
ZIV	zona incerta, ventral part	30–33	
Zo	zonal layer of the superior colliculus	33–41	


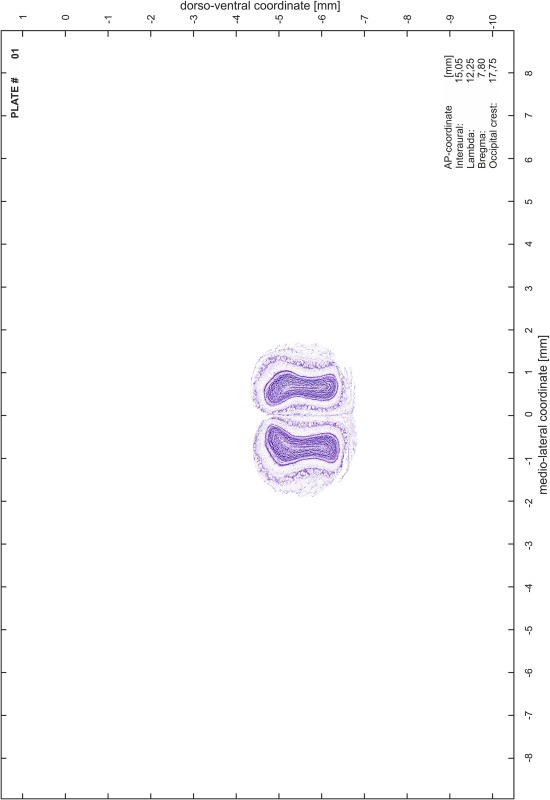

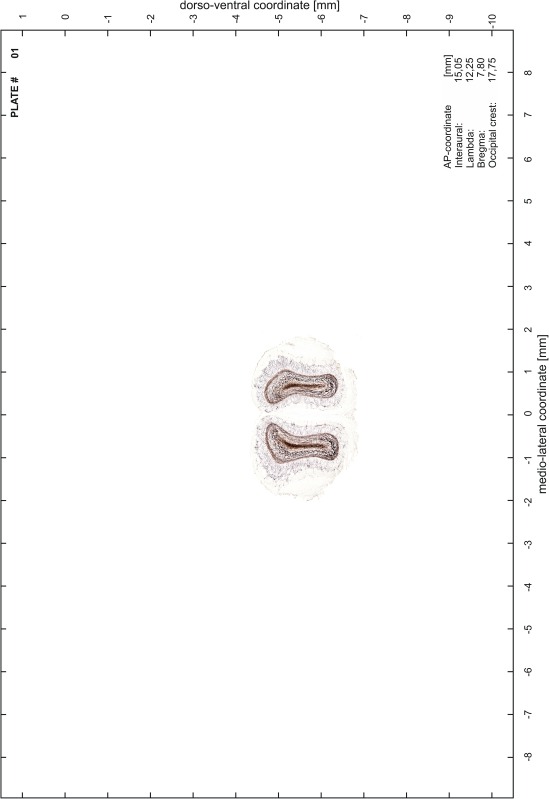

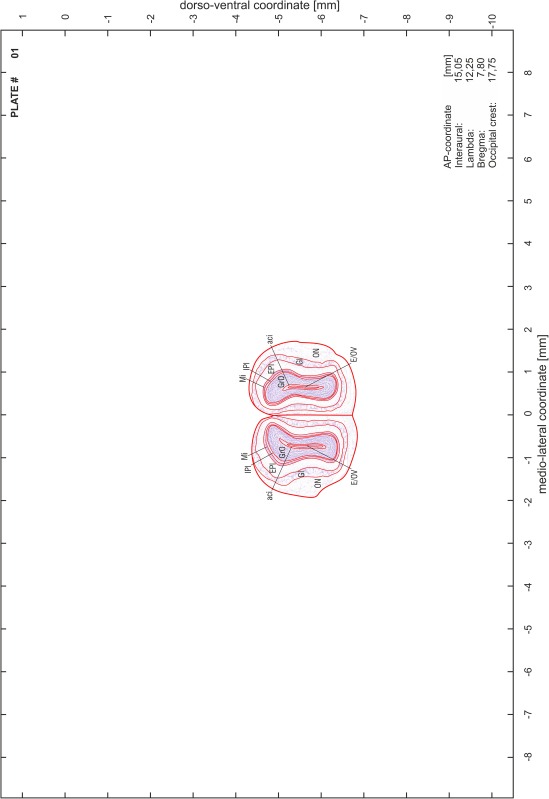

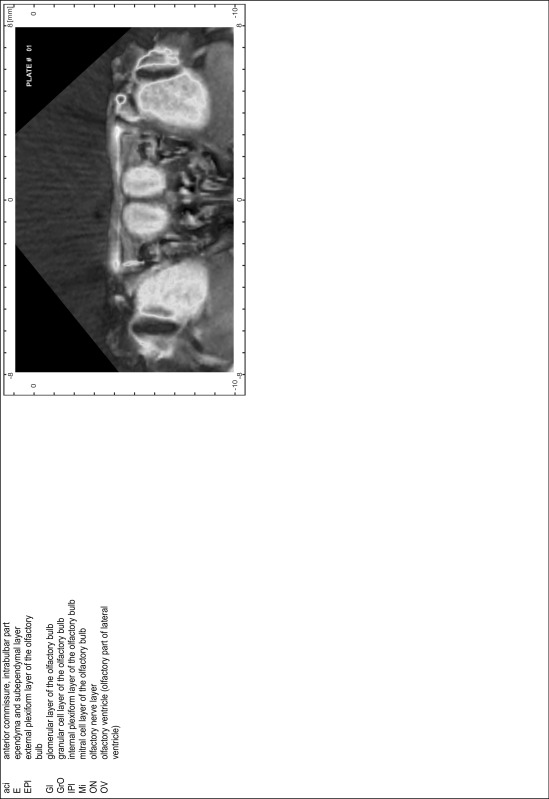

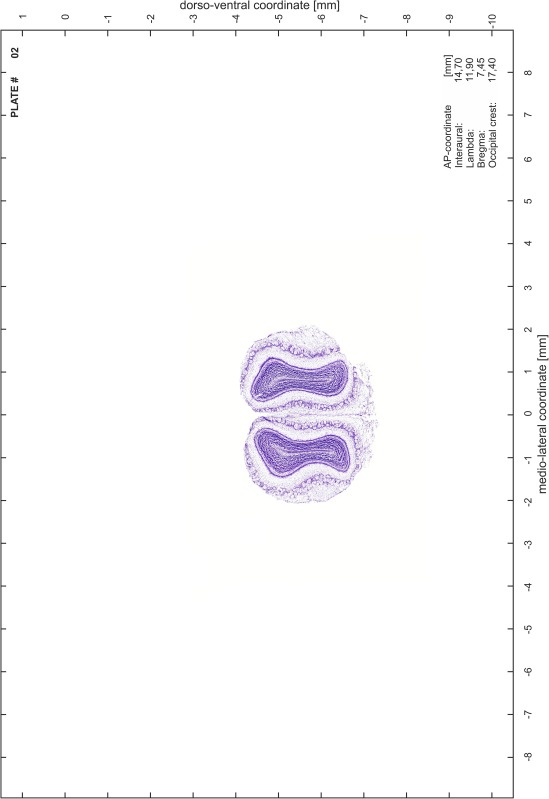

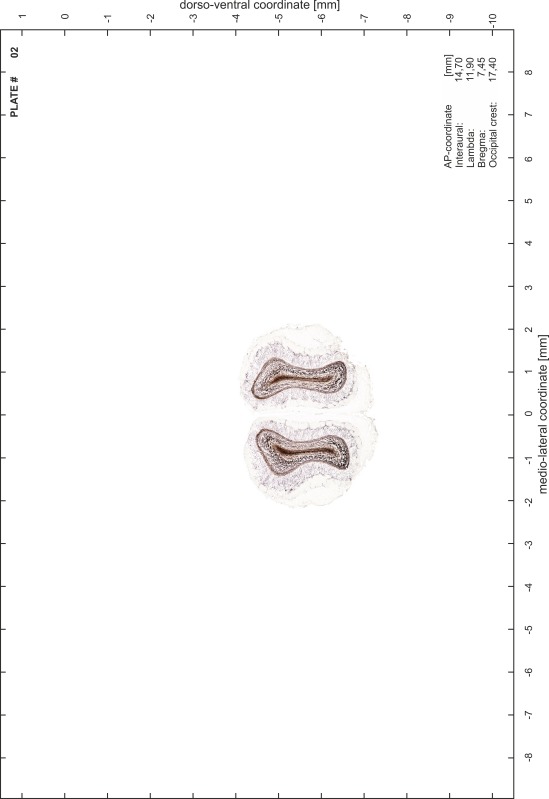

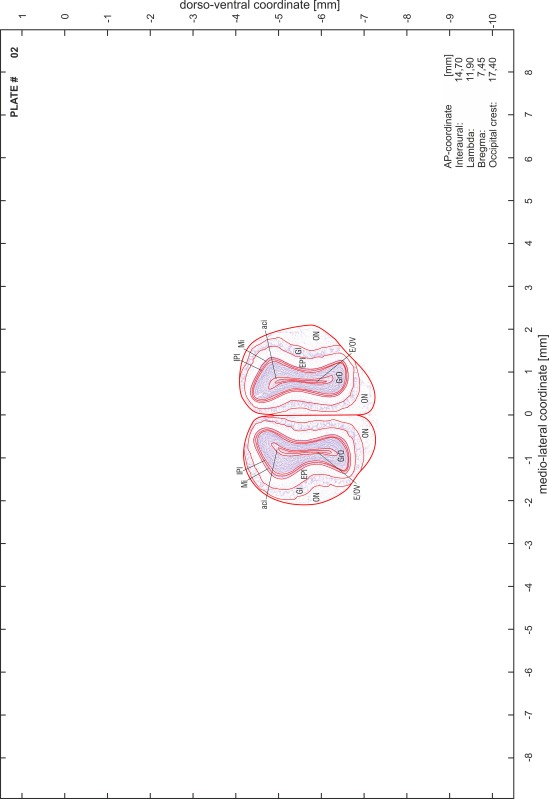

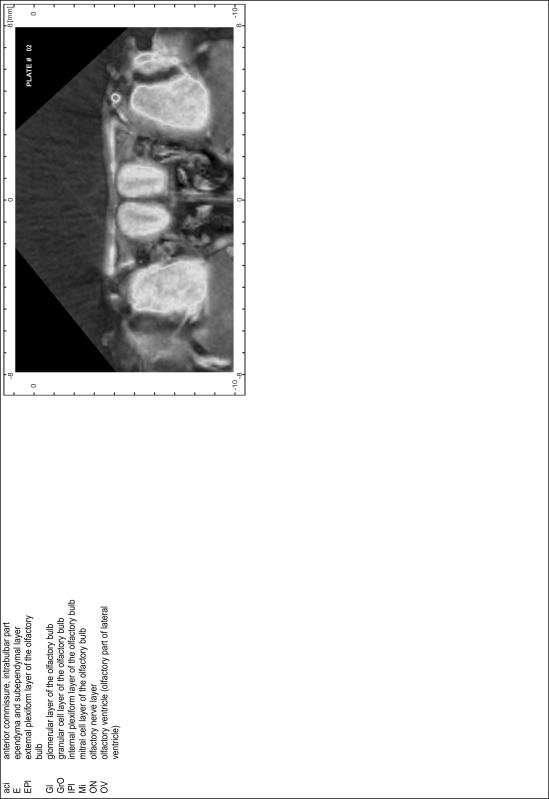

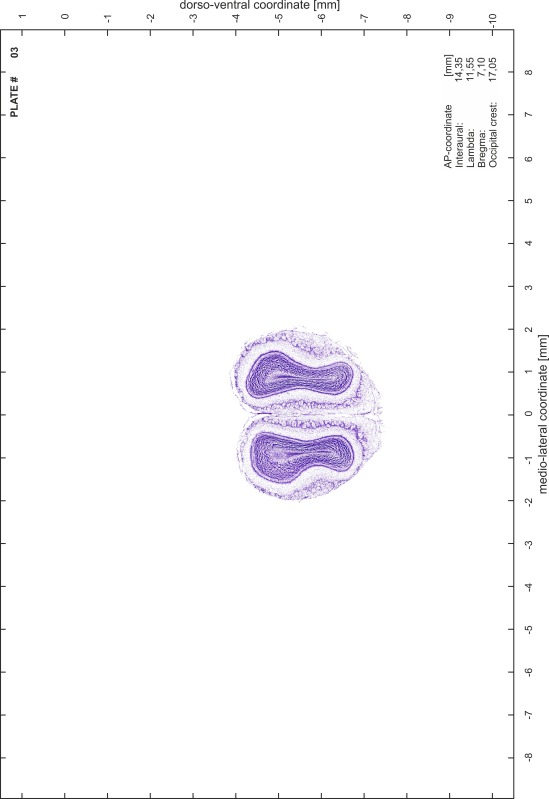

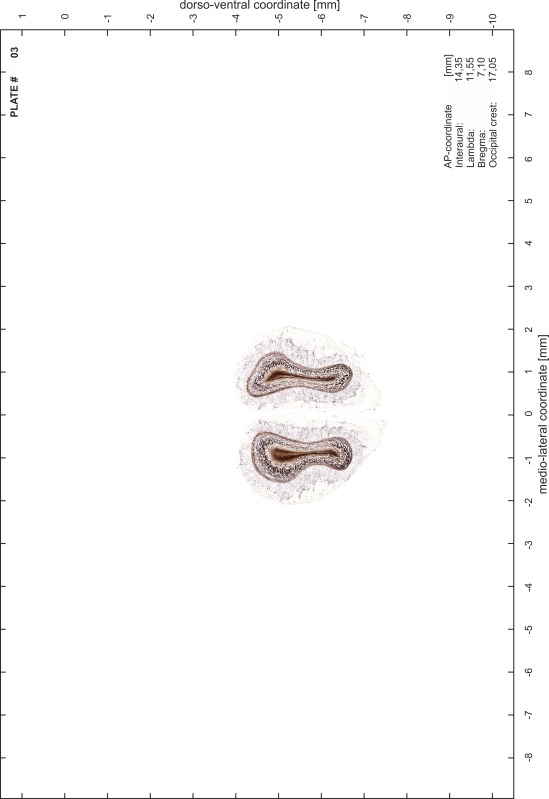

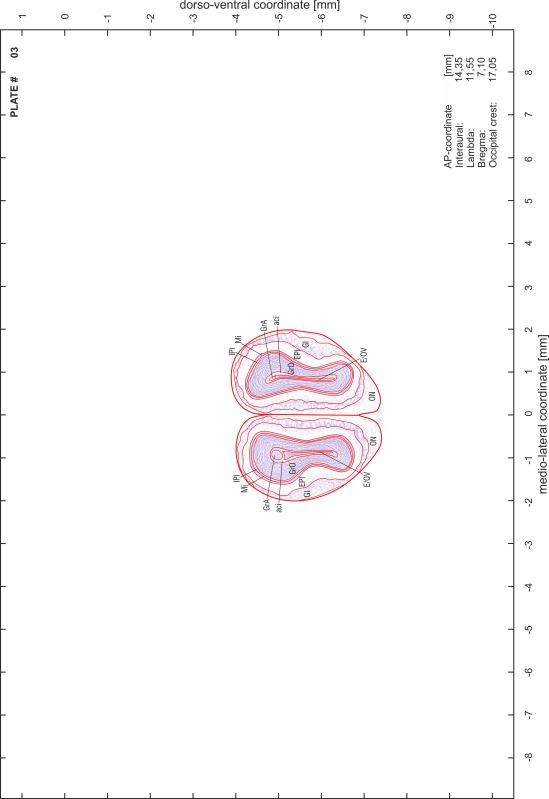

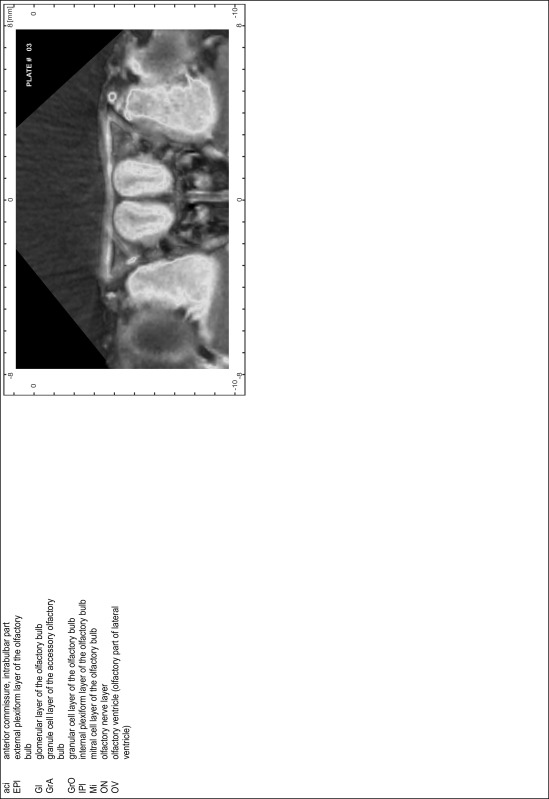

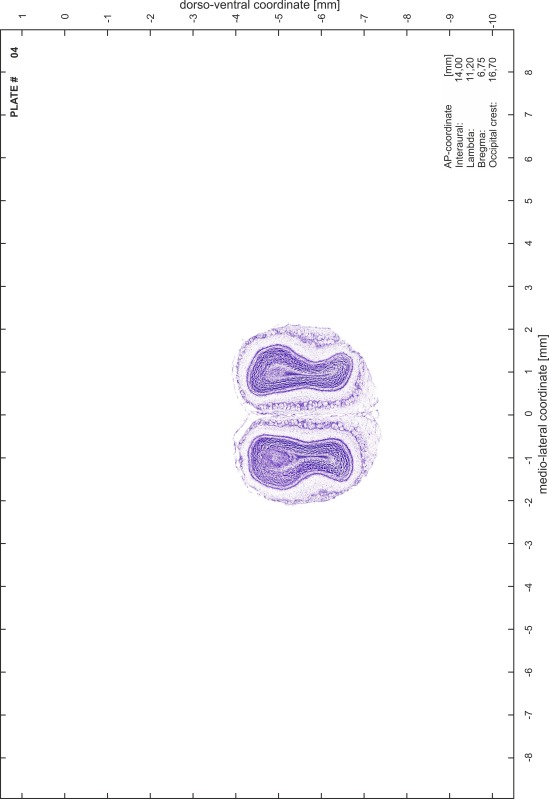

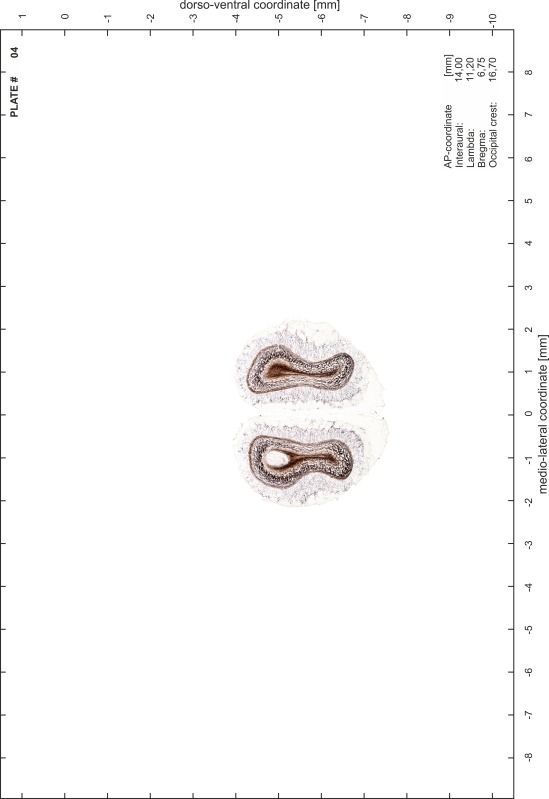

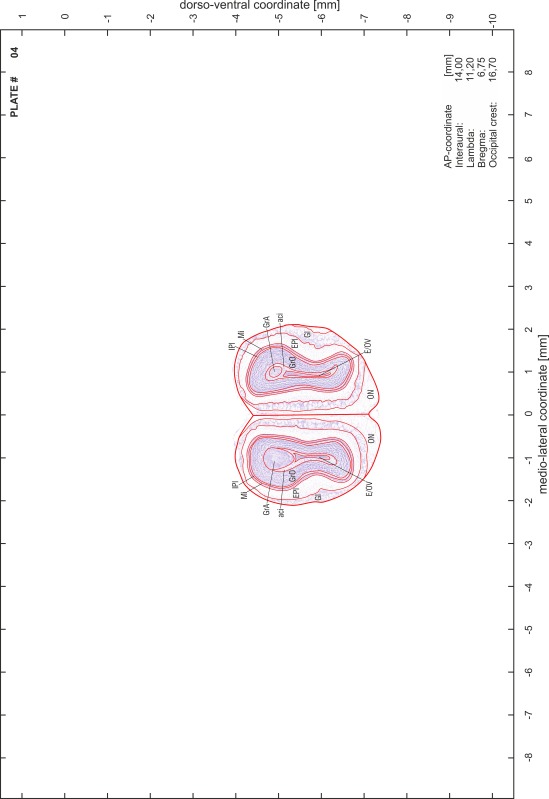

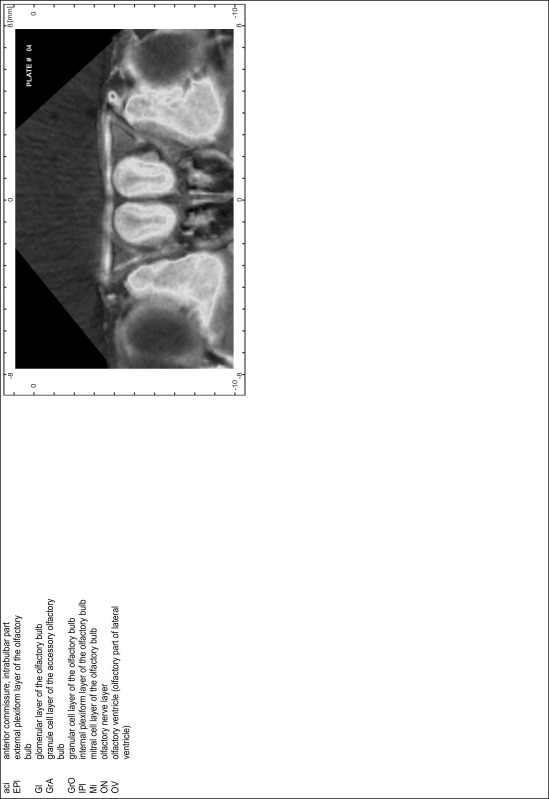

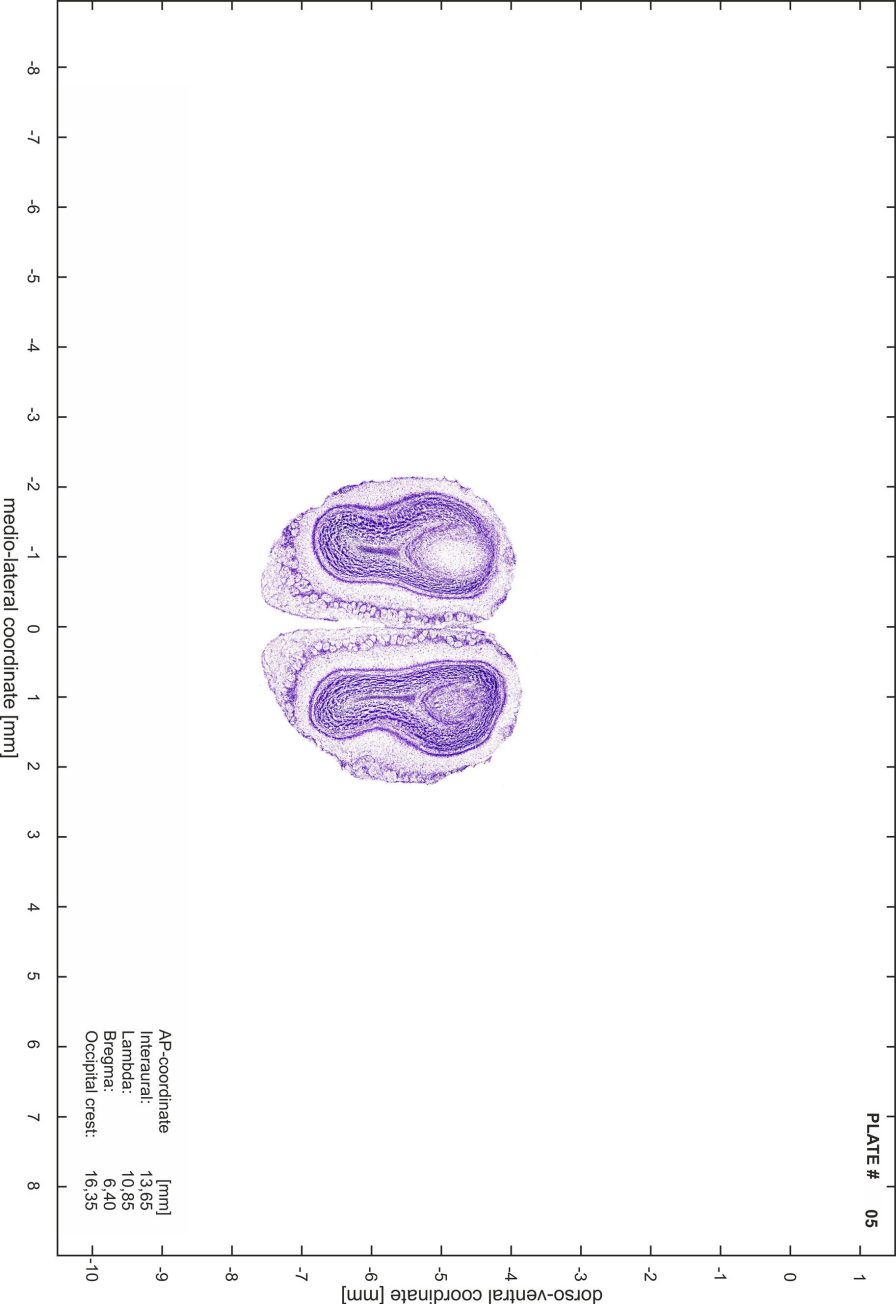

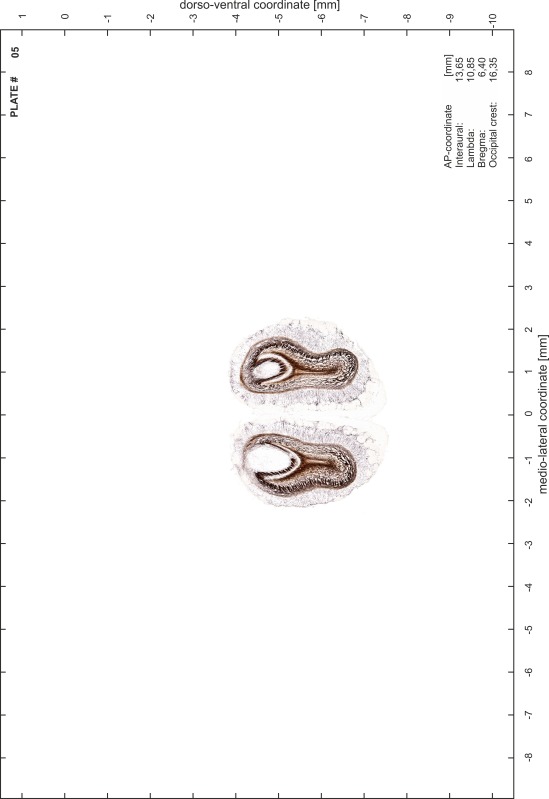

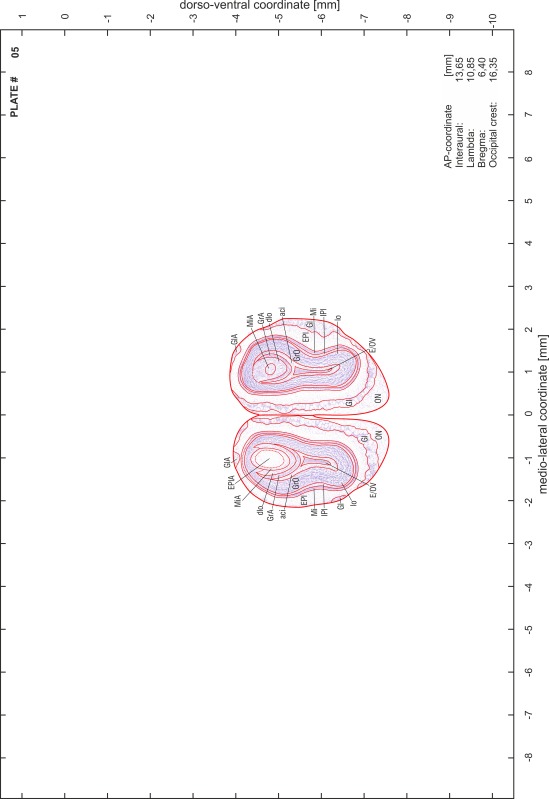

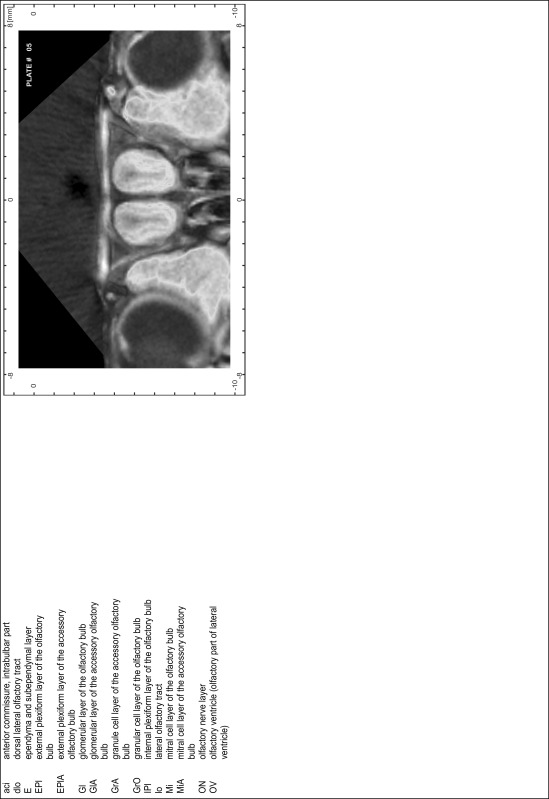

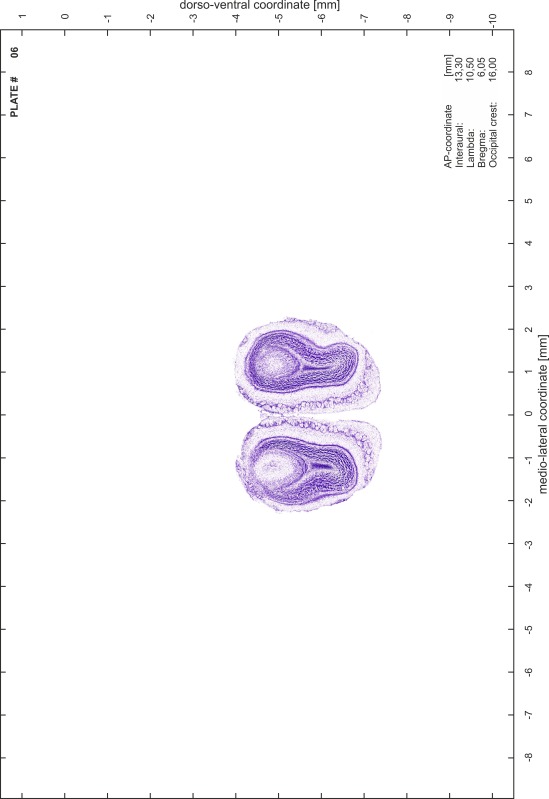

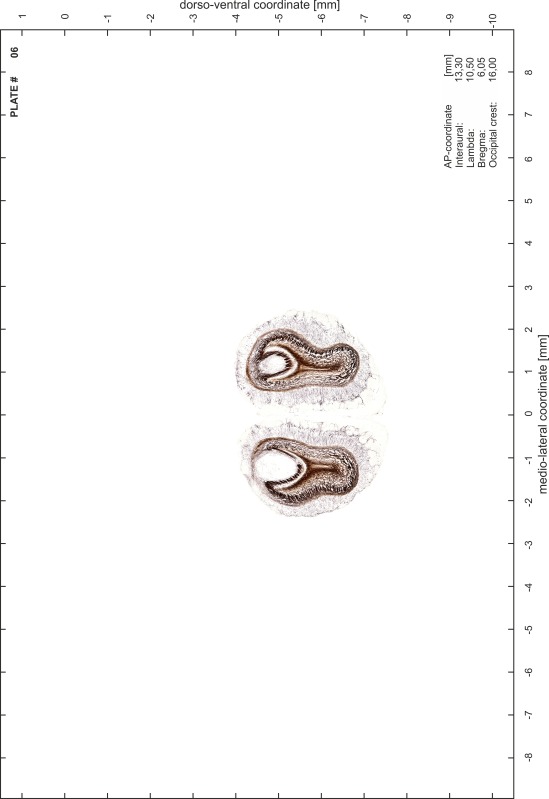

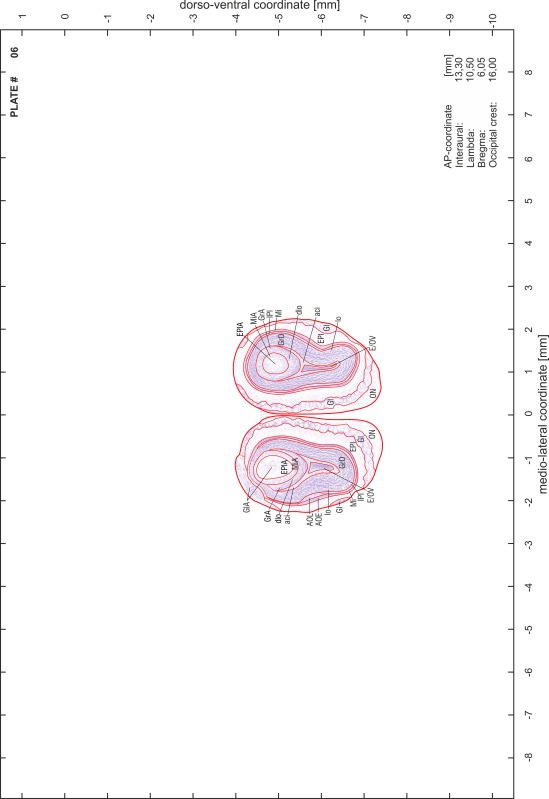

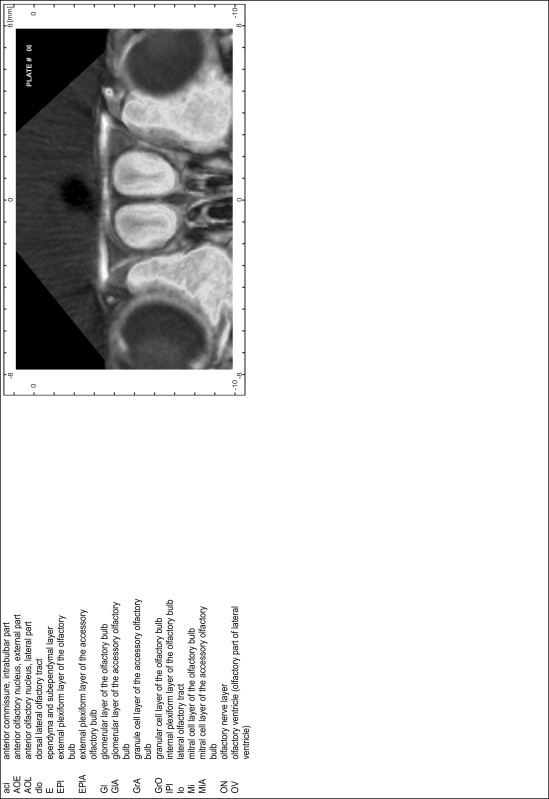

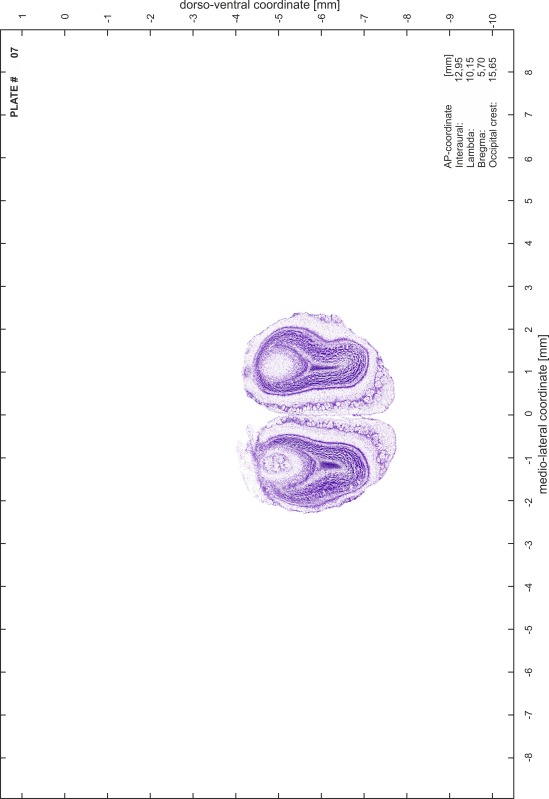

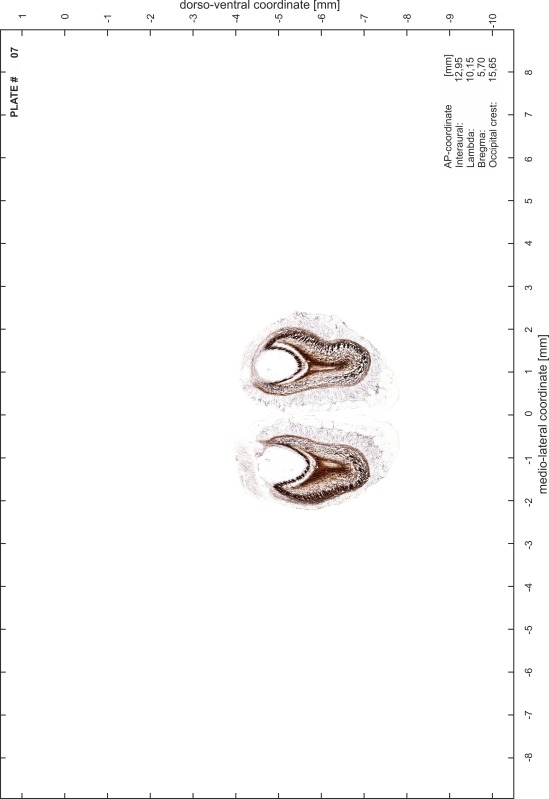

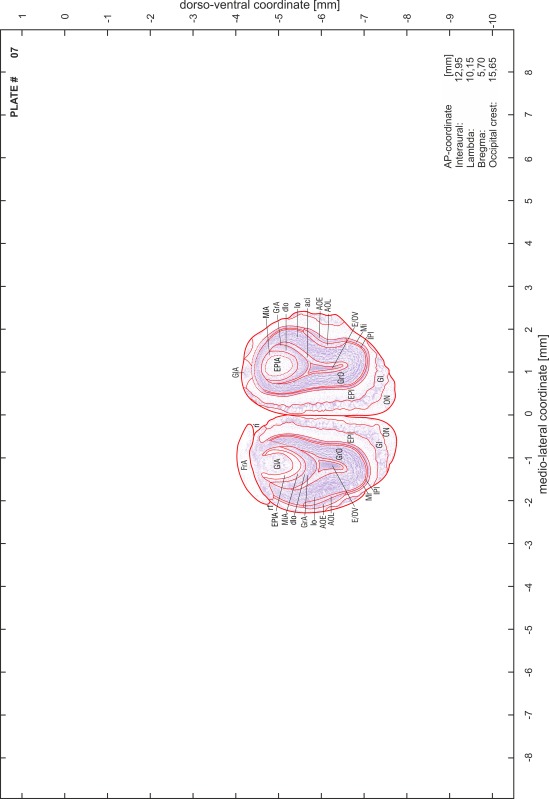

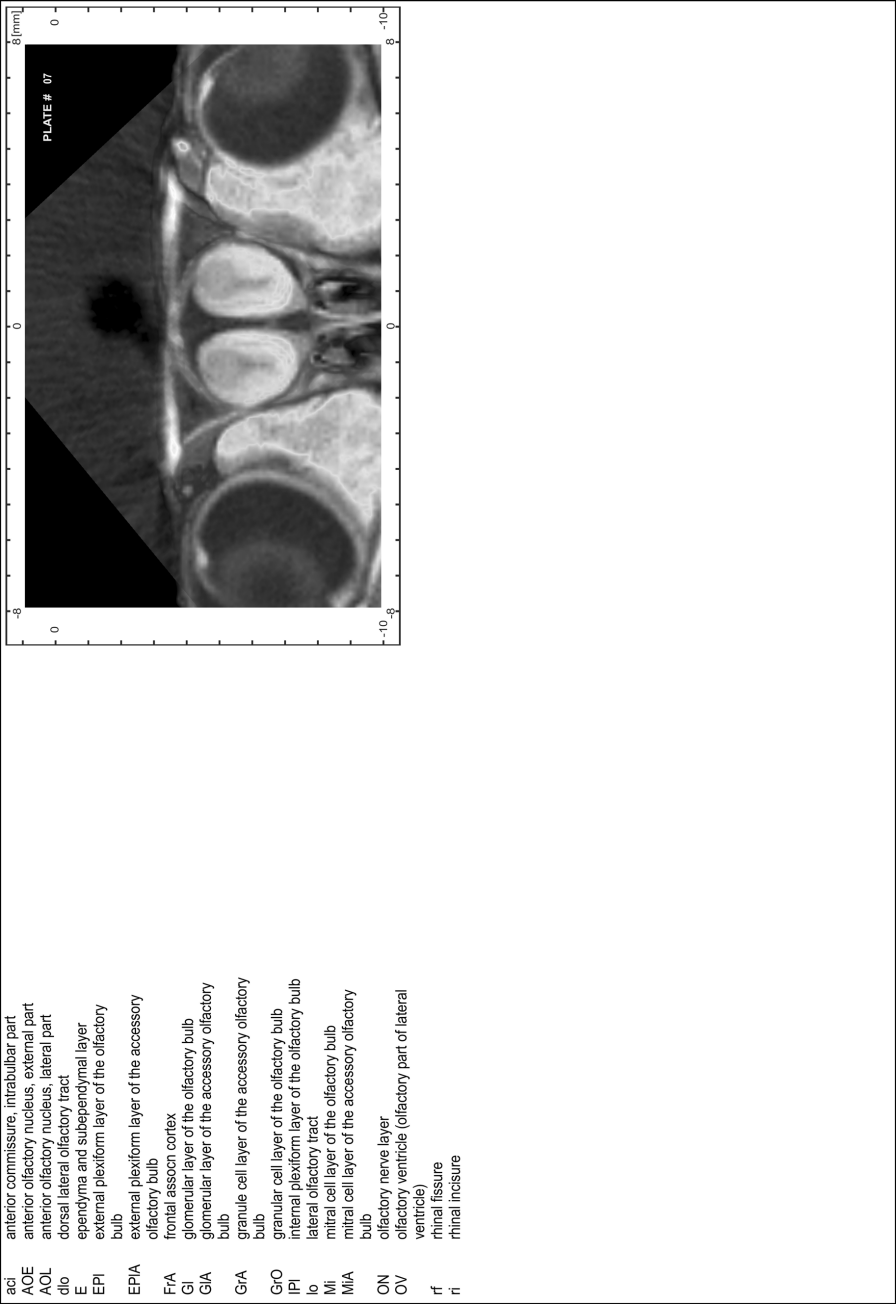

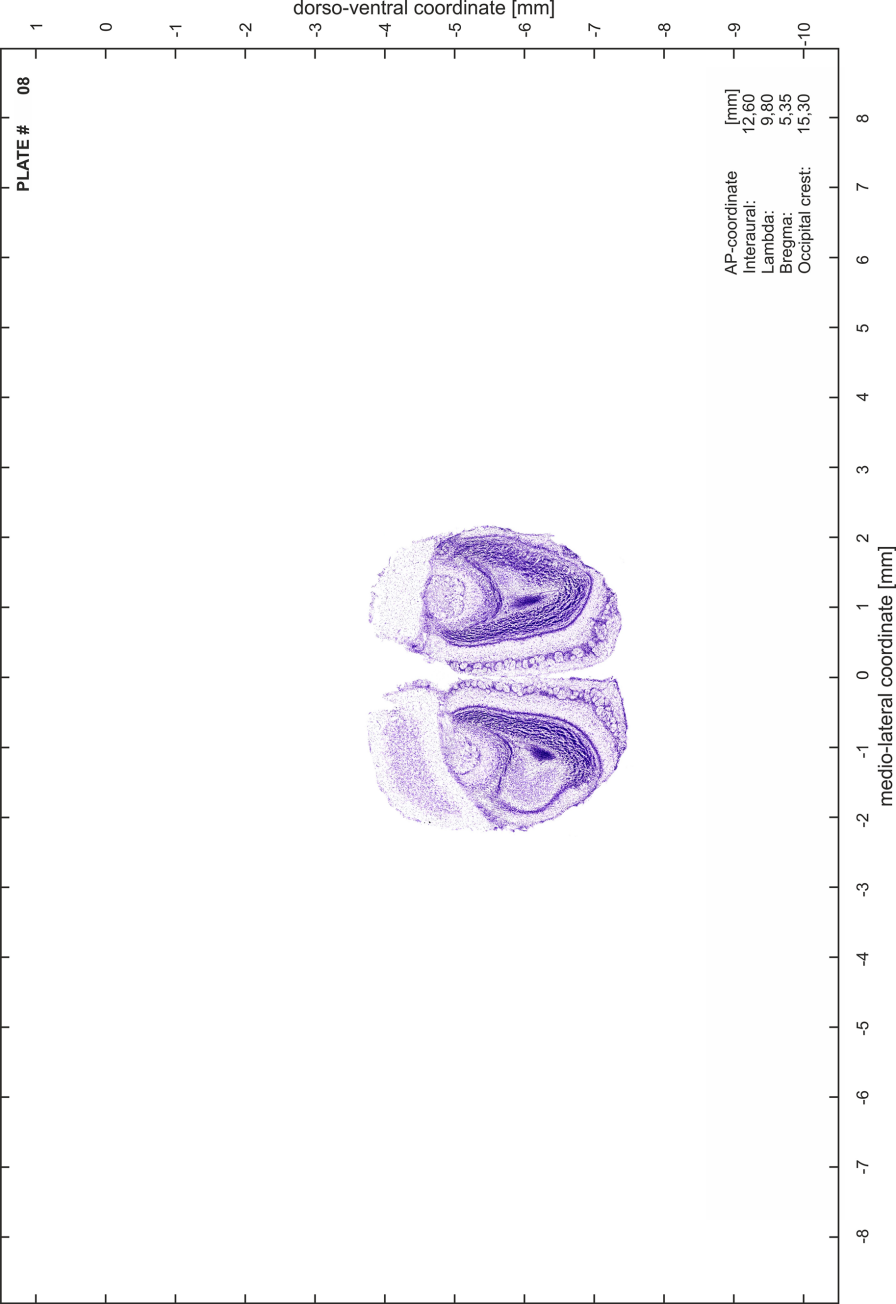

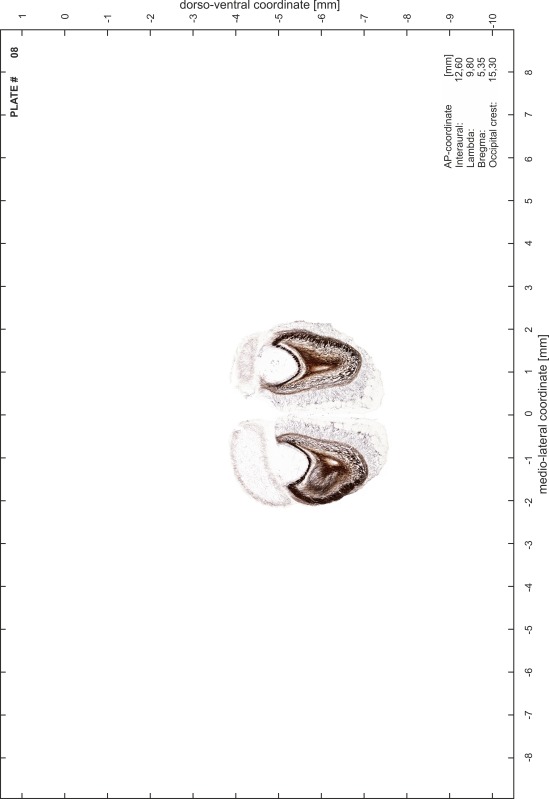

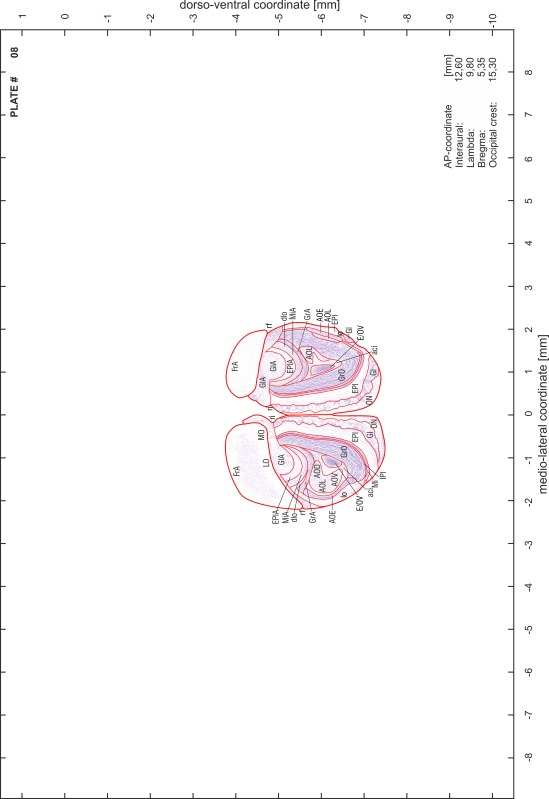

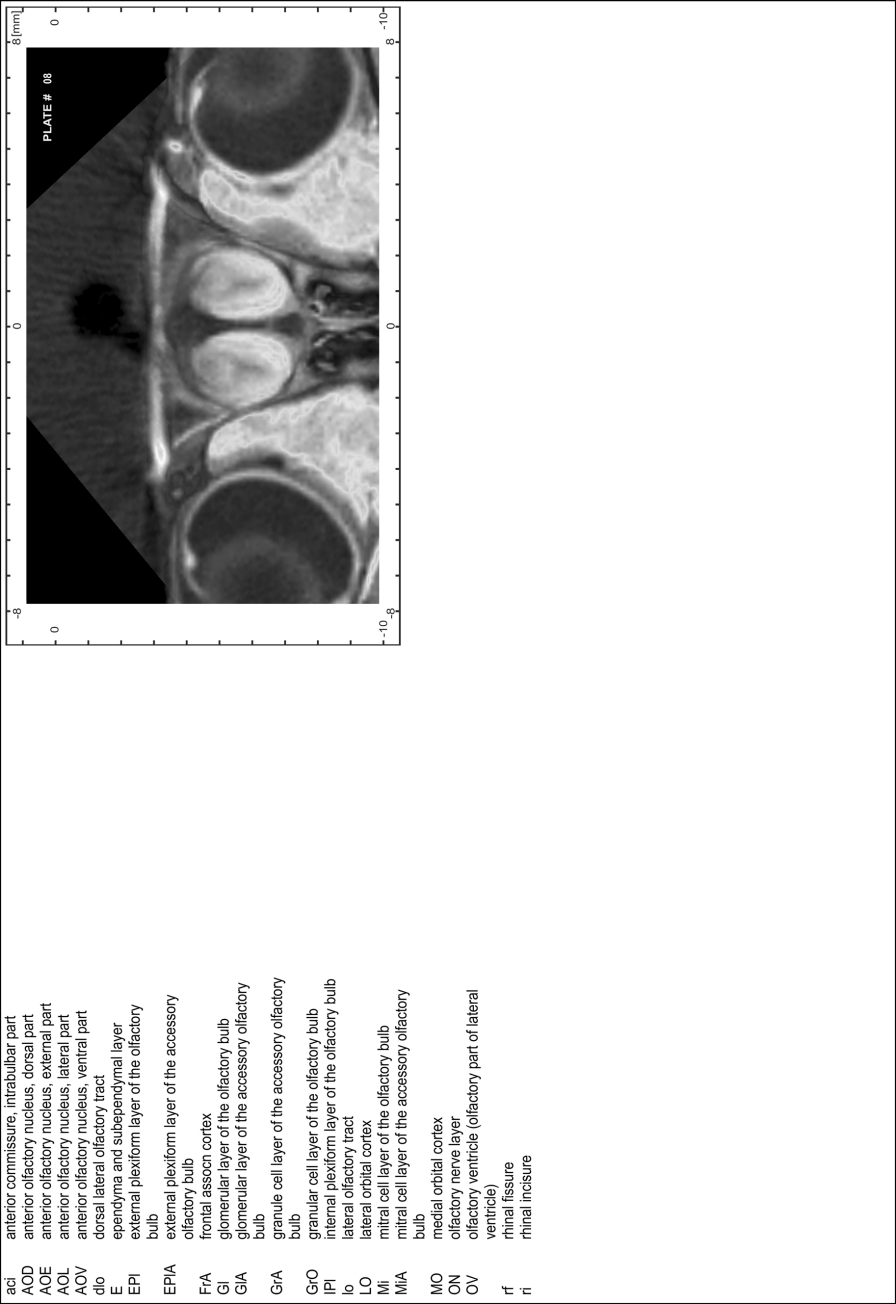

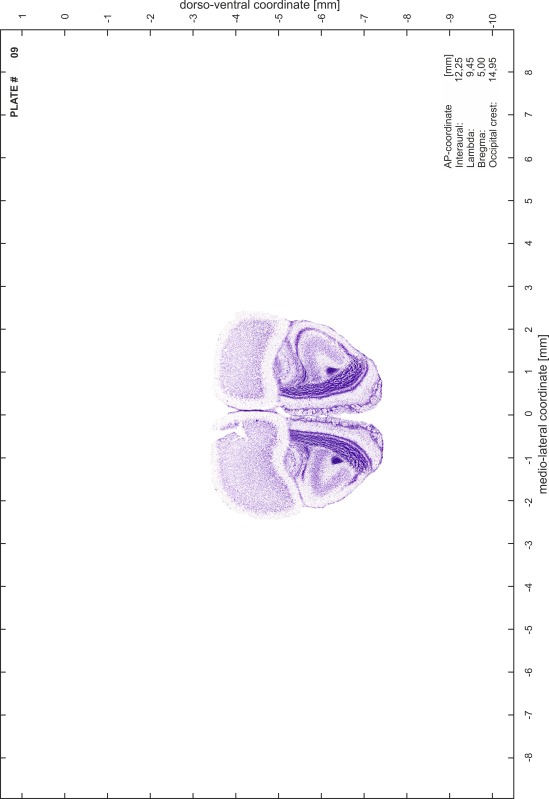

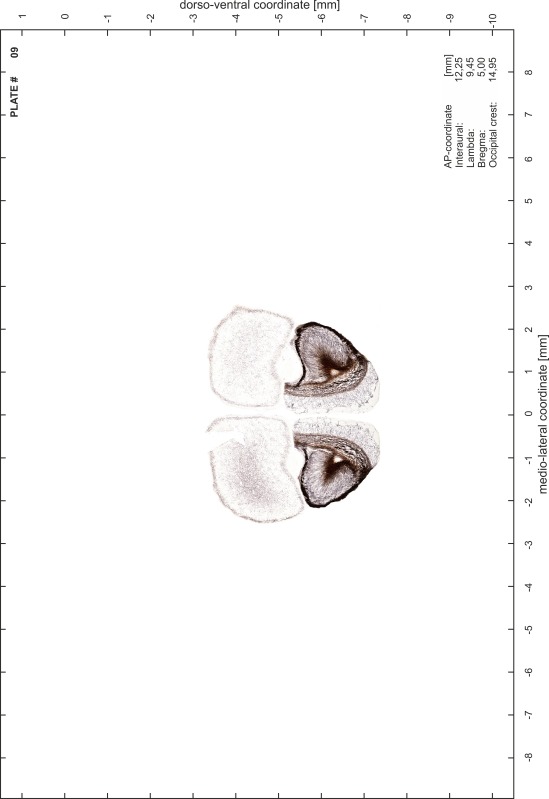

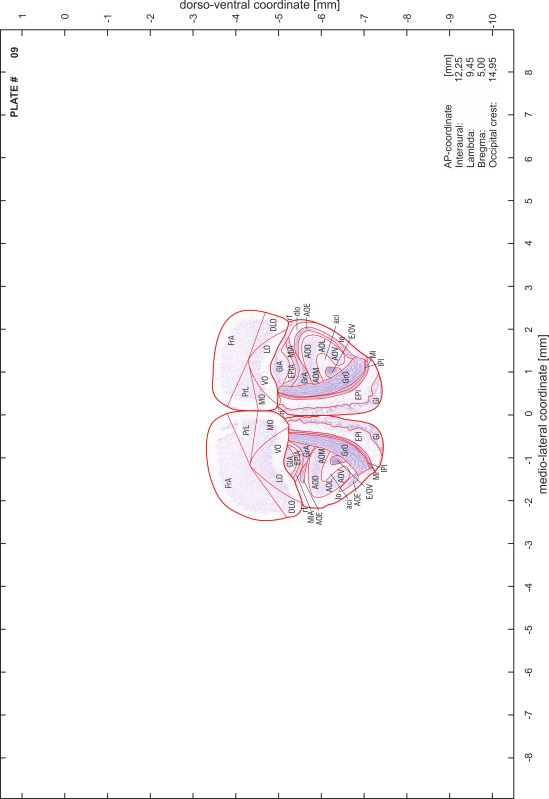

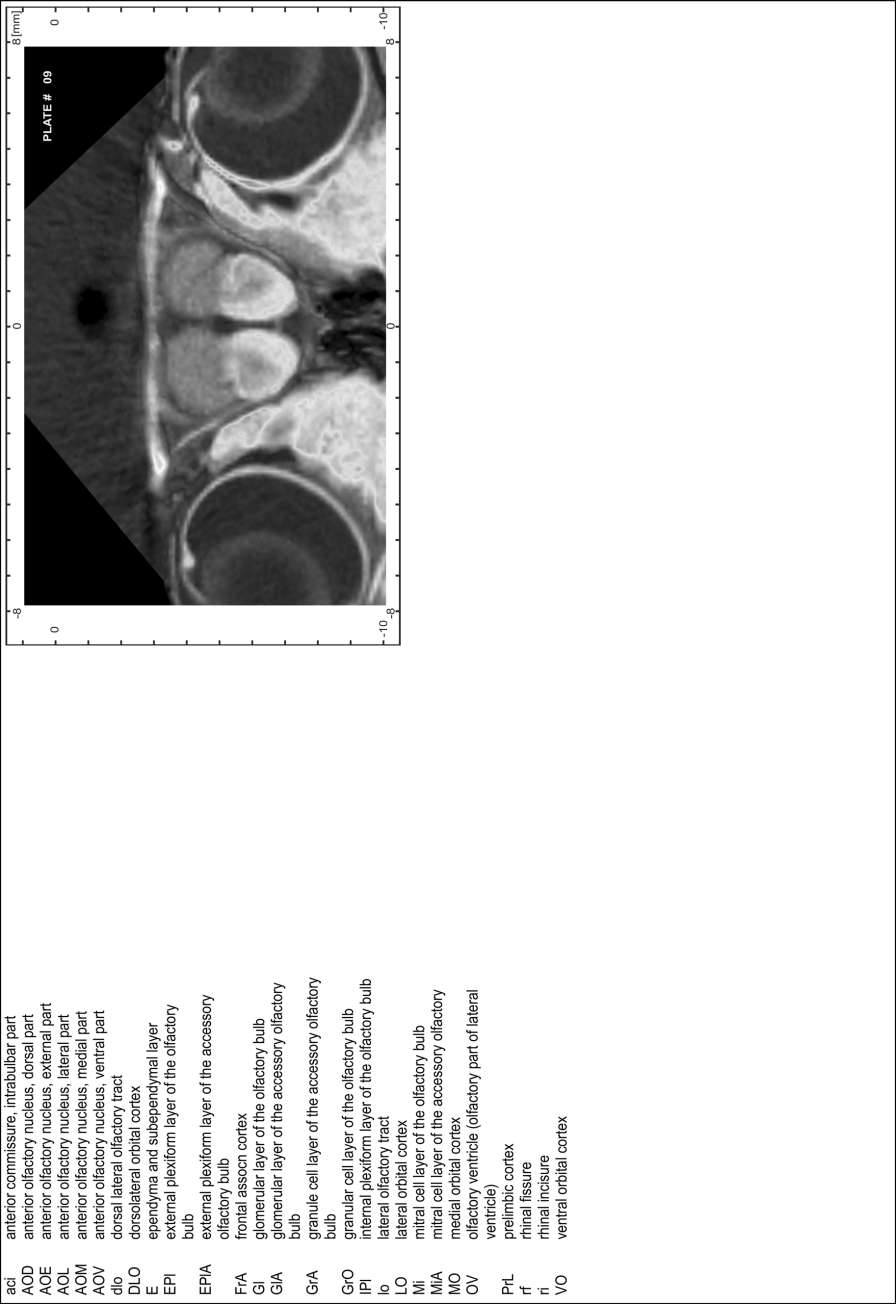

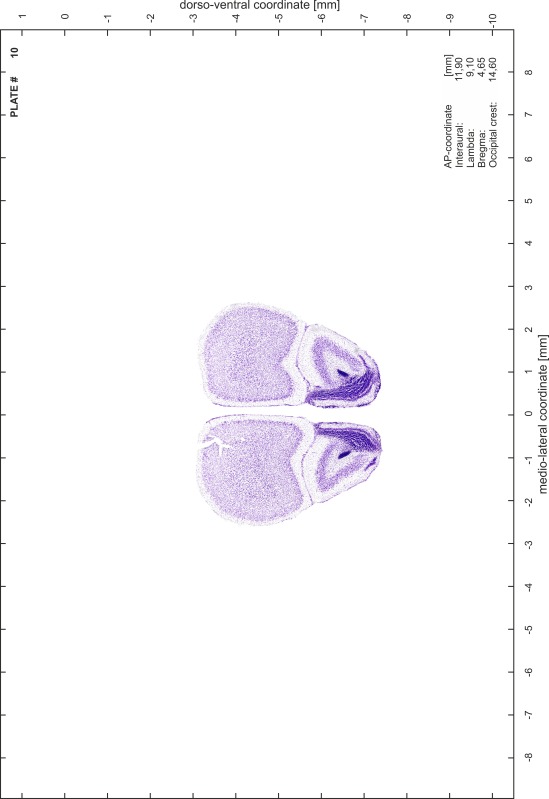

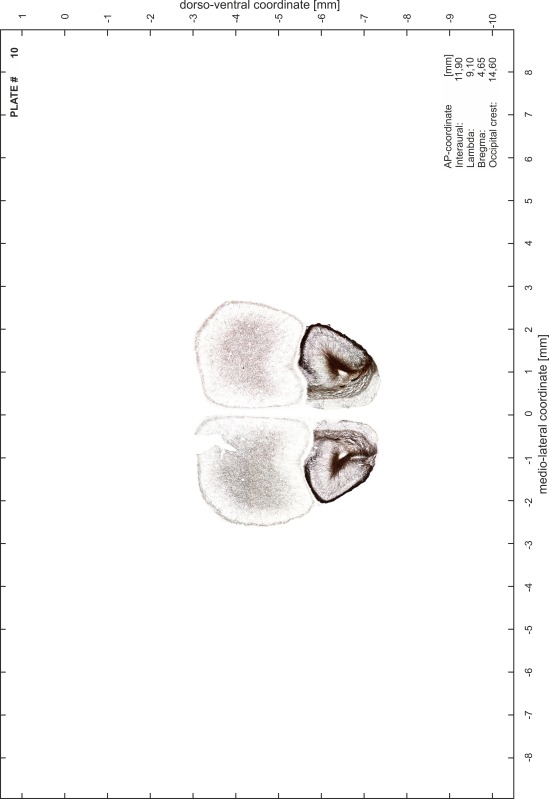

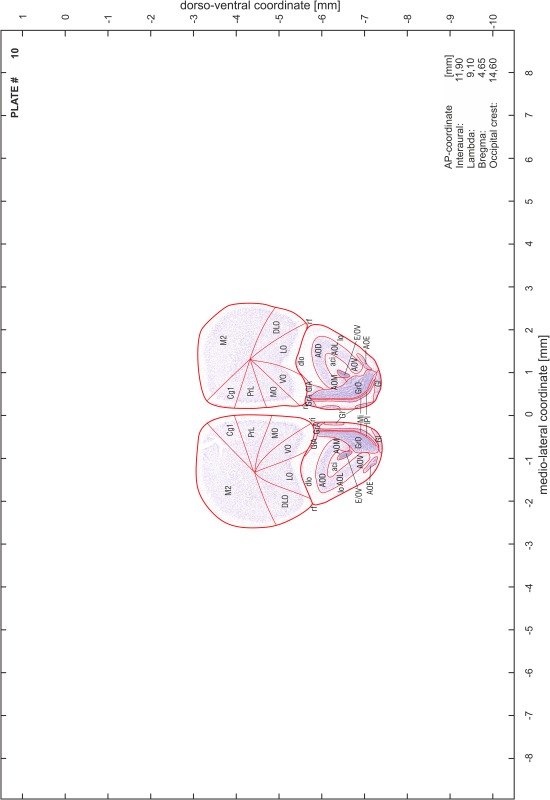

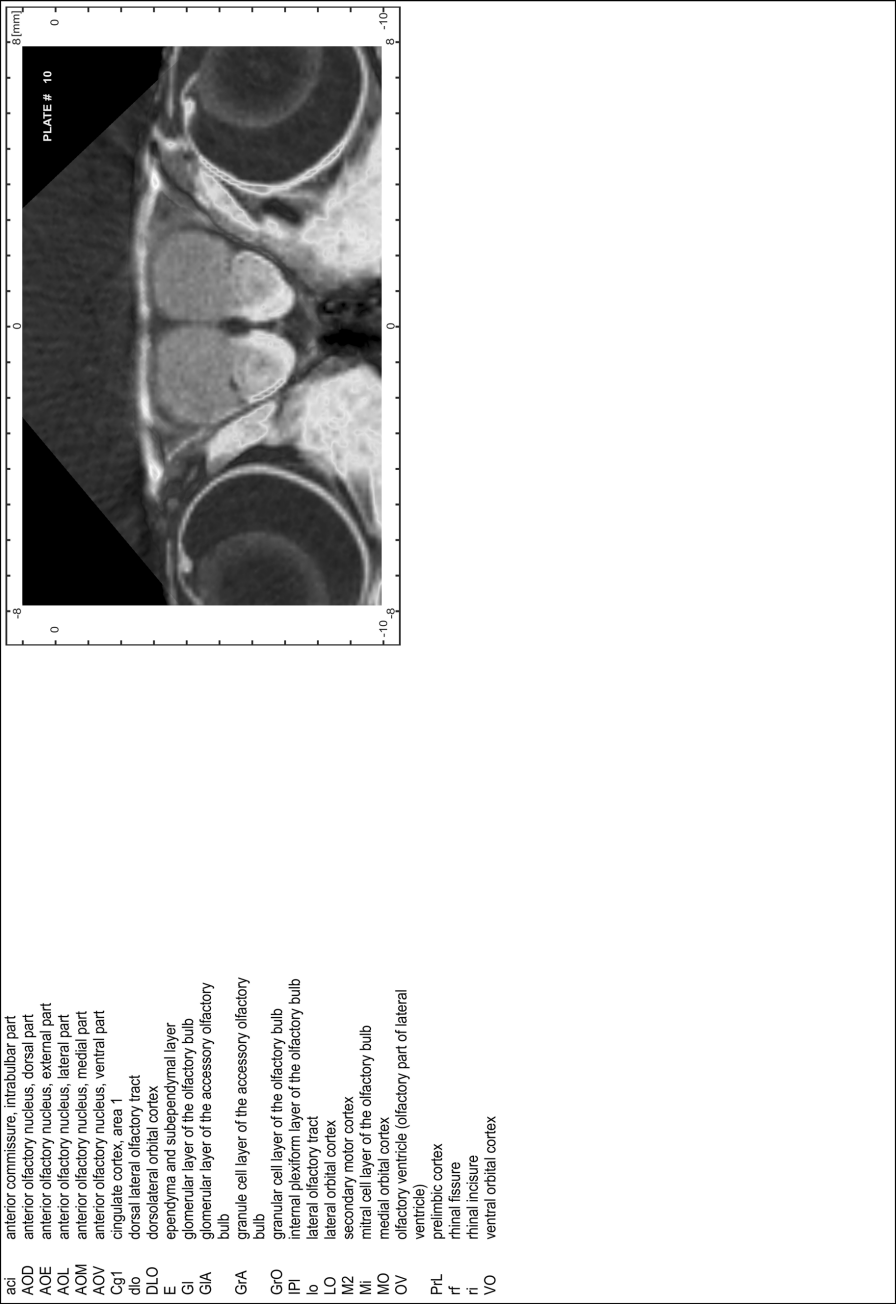

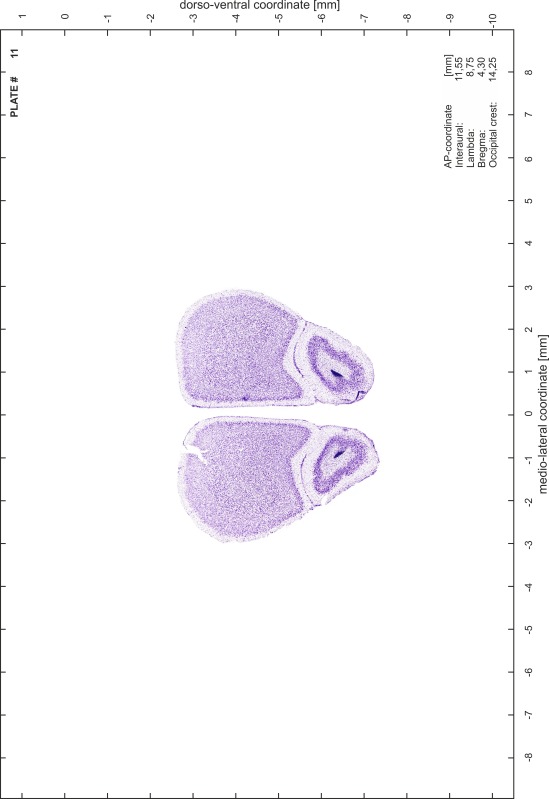

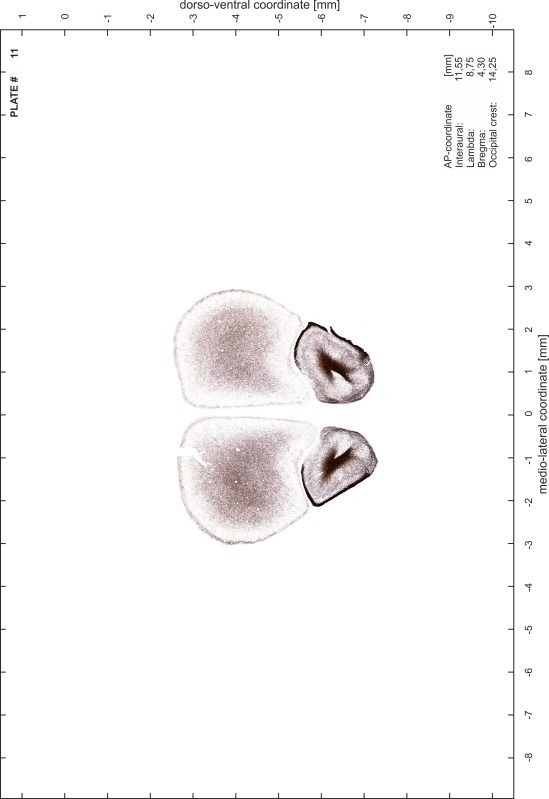

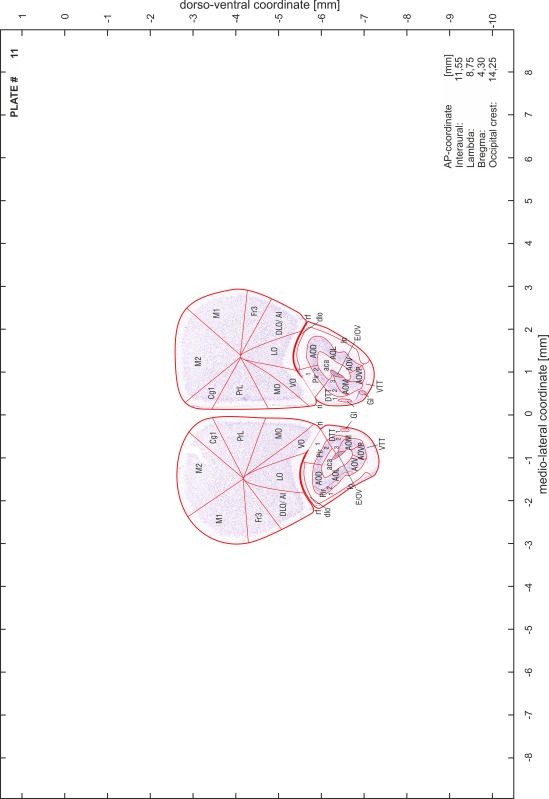

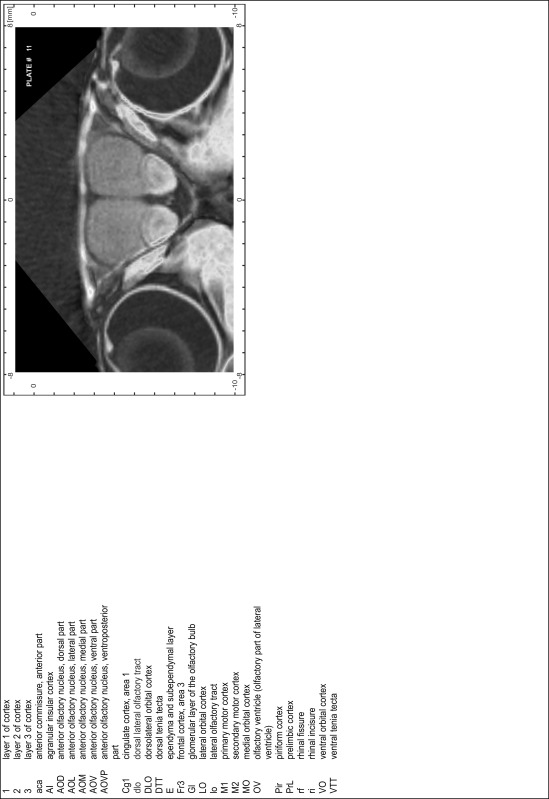

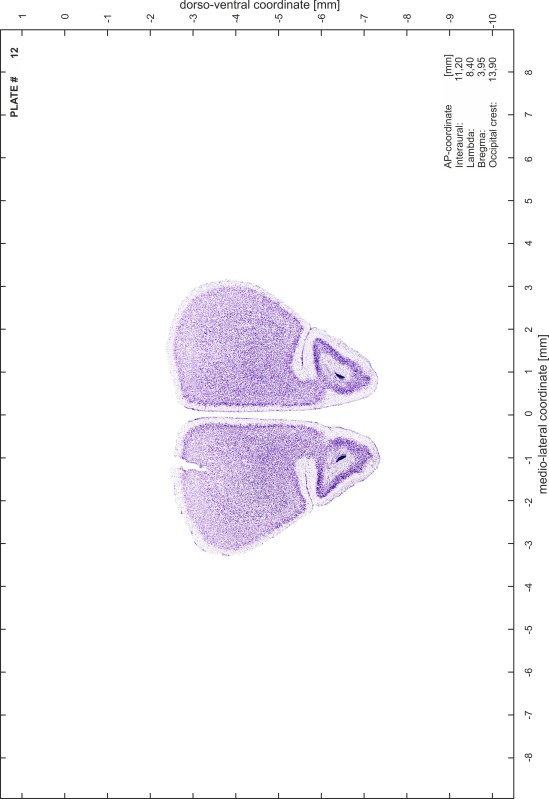

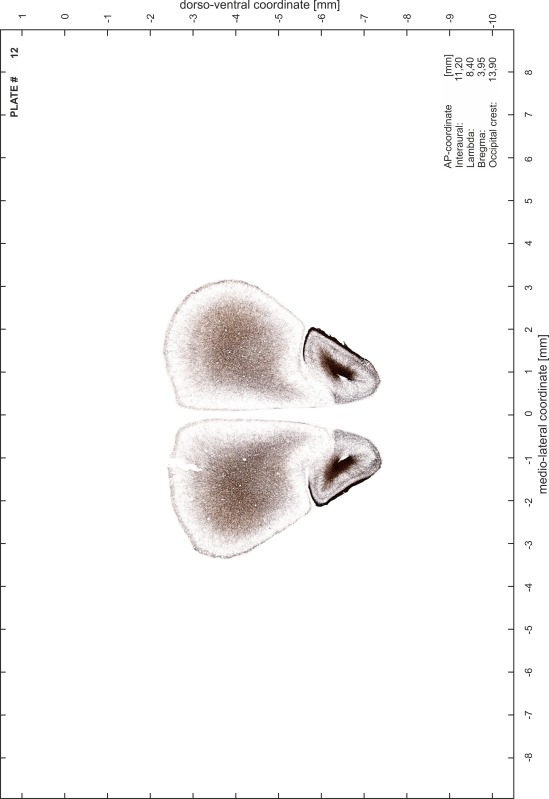

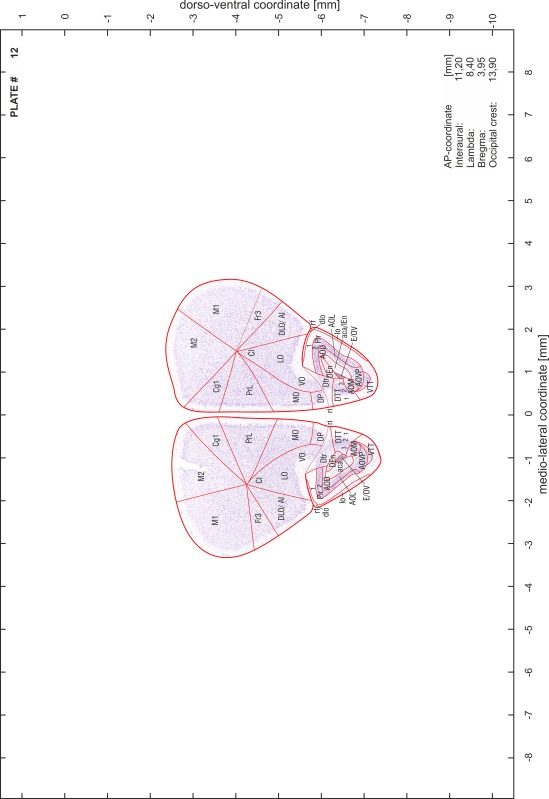

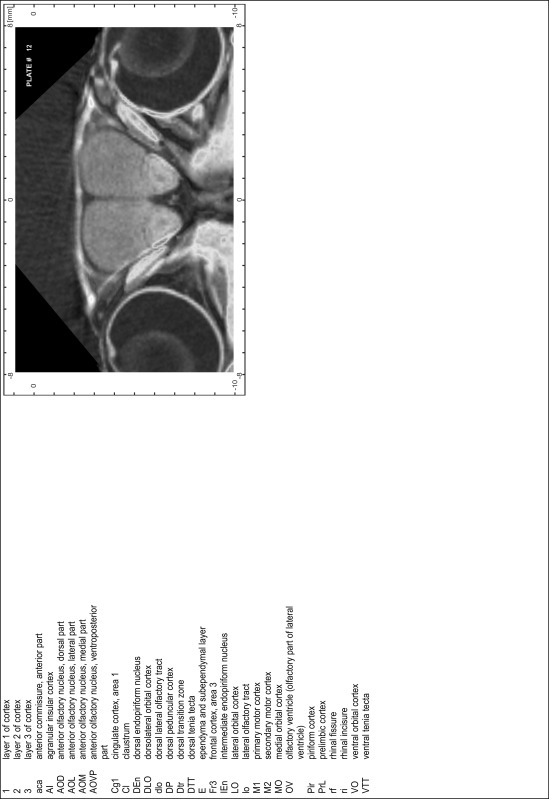

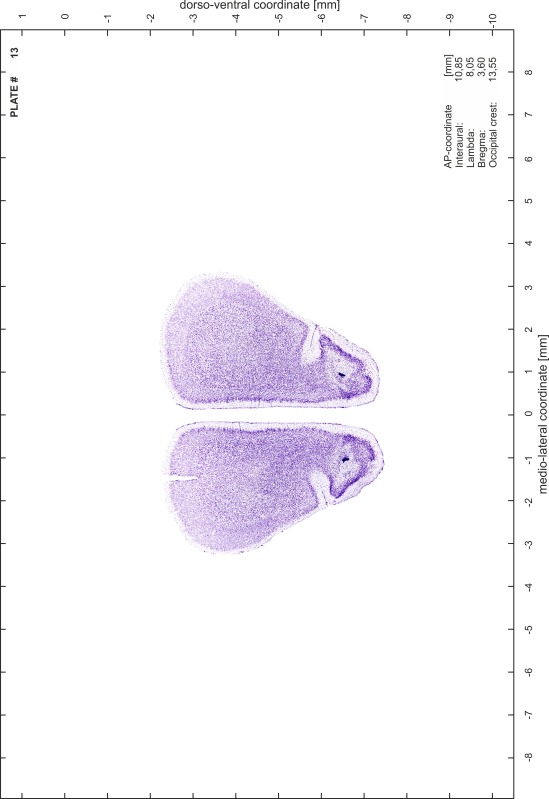

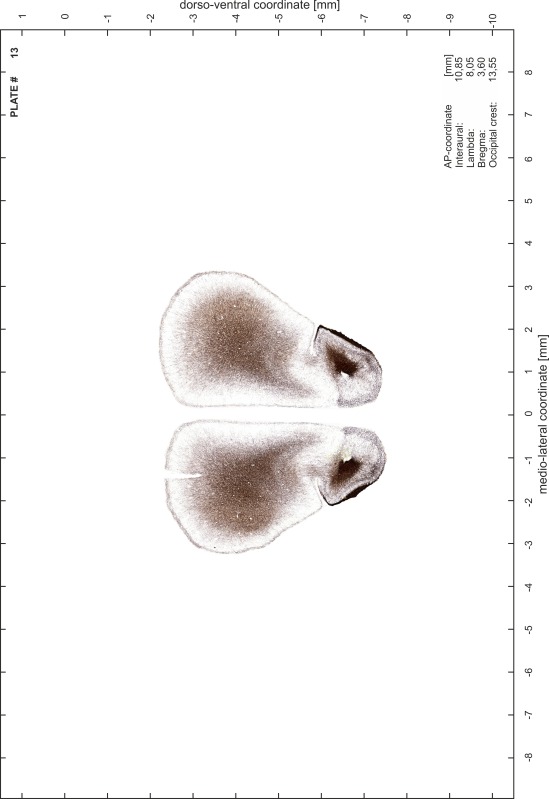

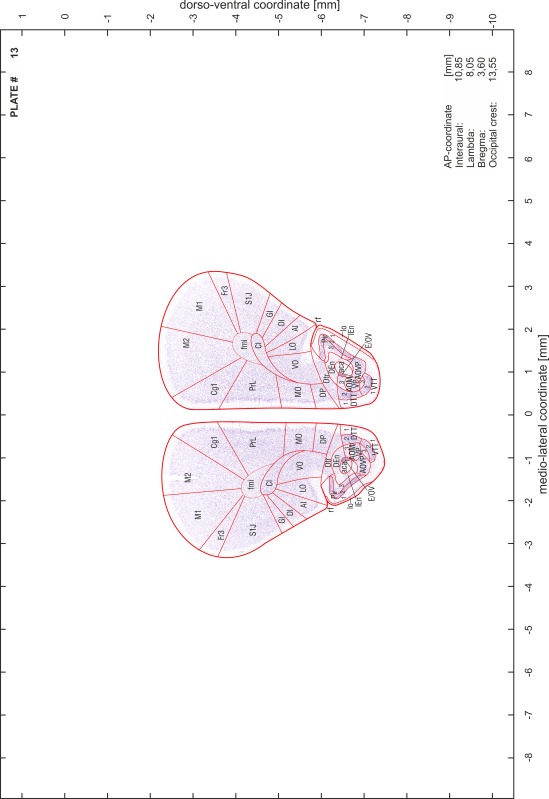

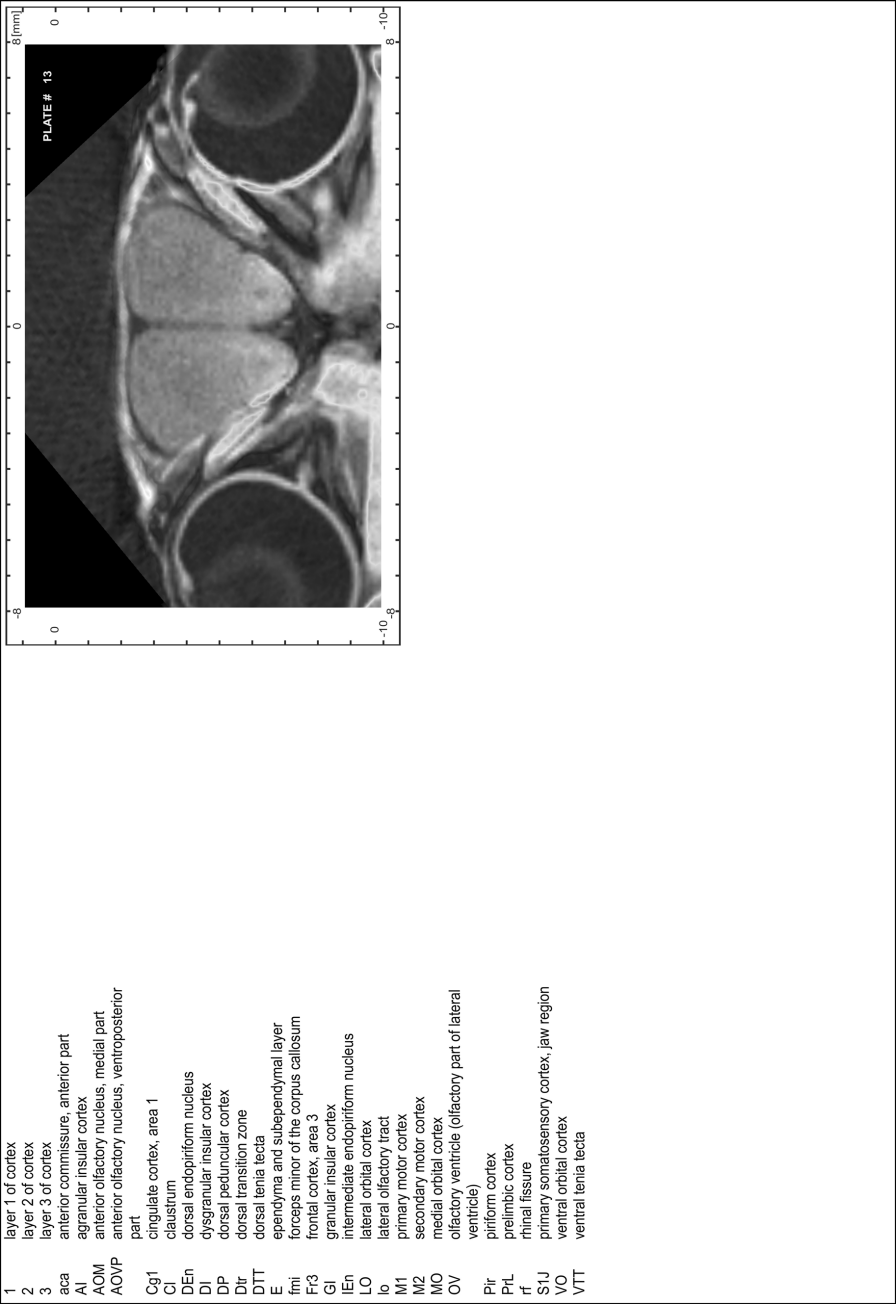

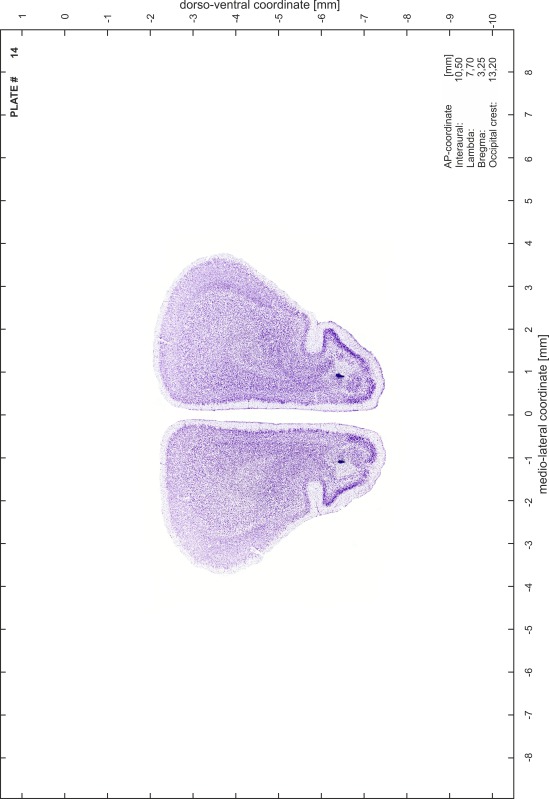

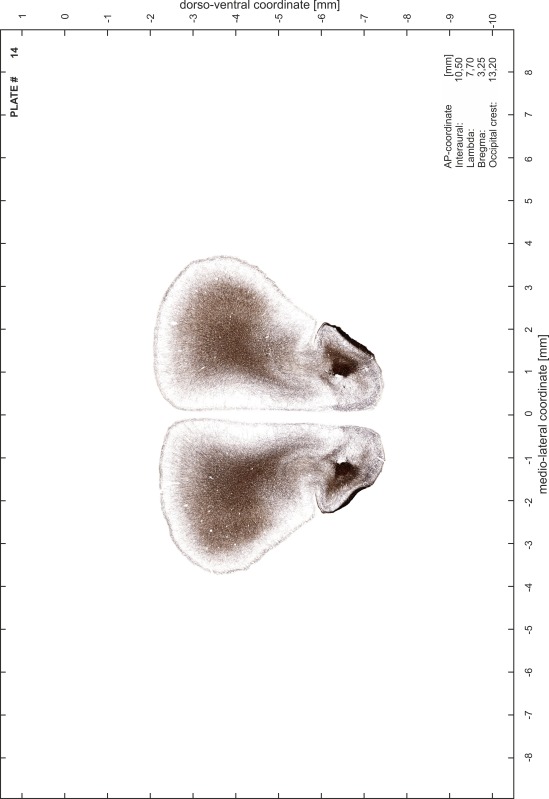

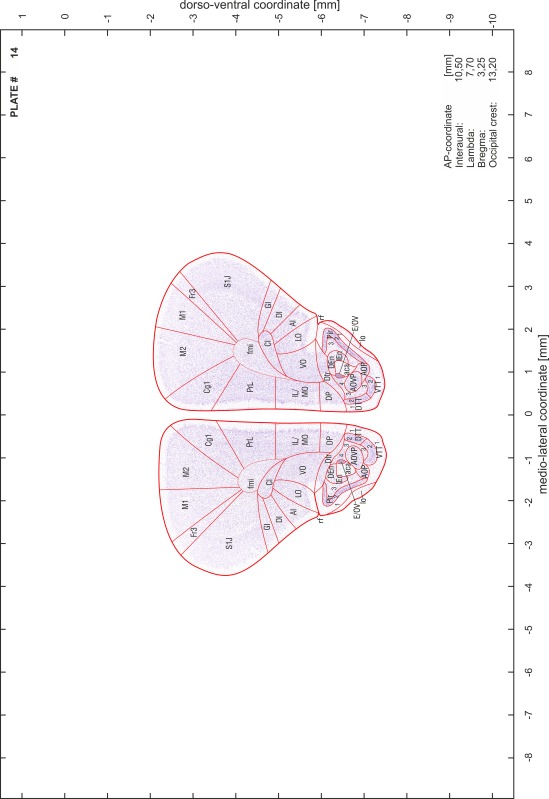

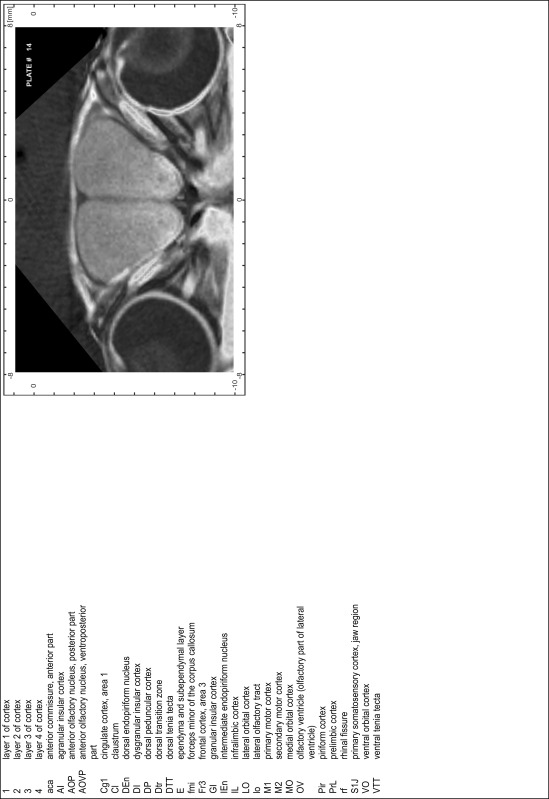

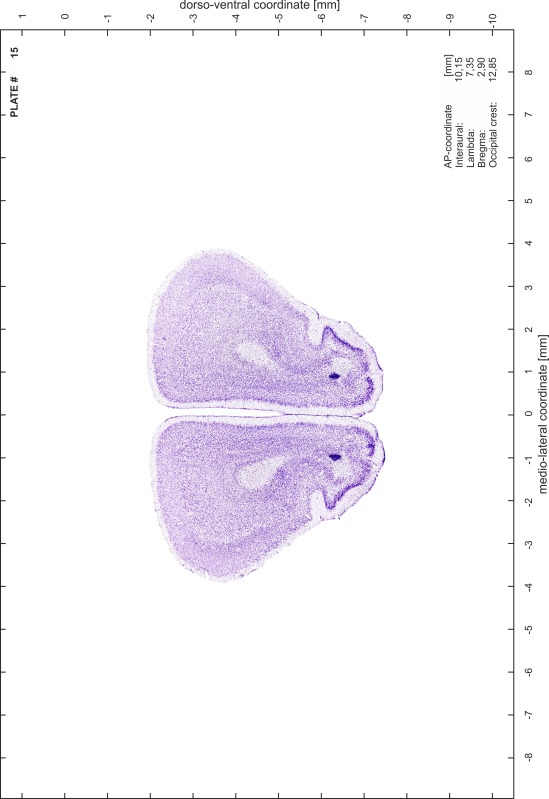

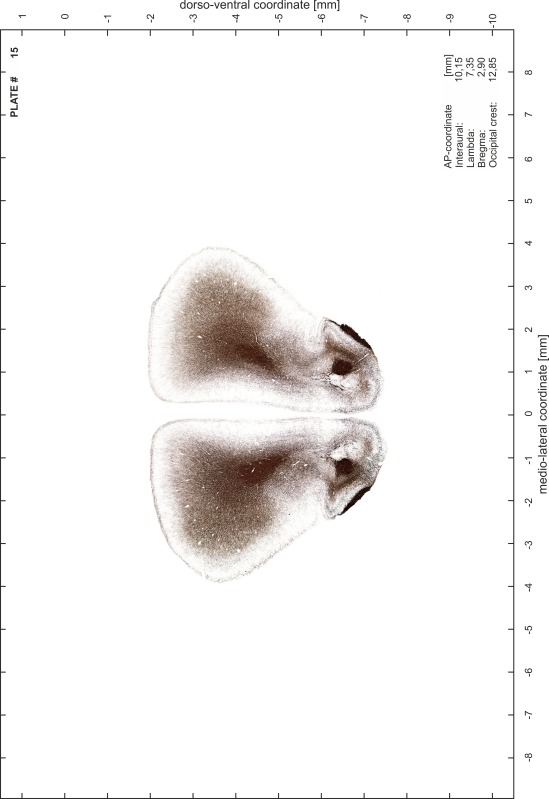

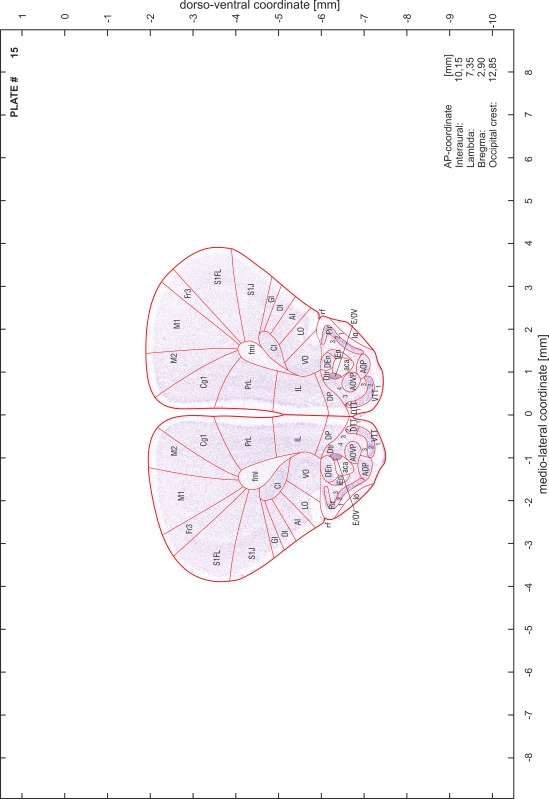

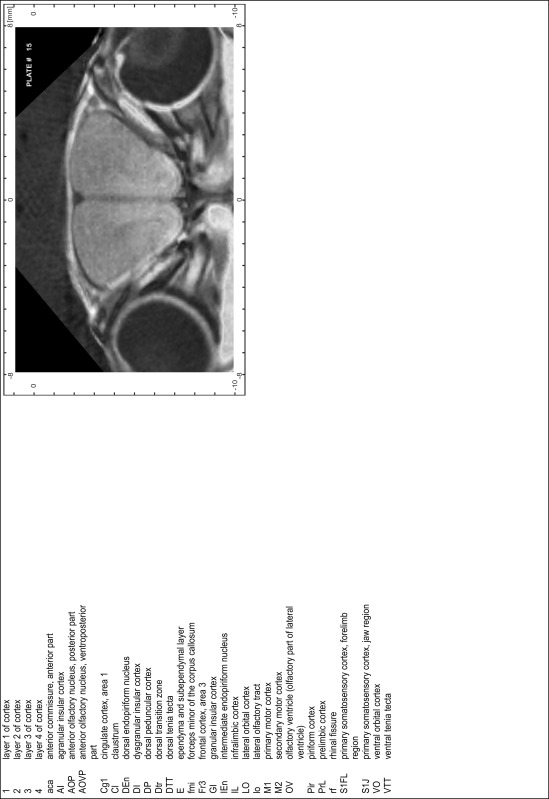

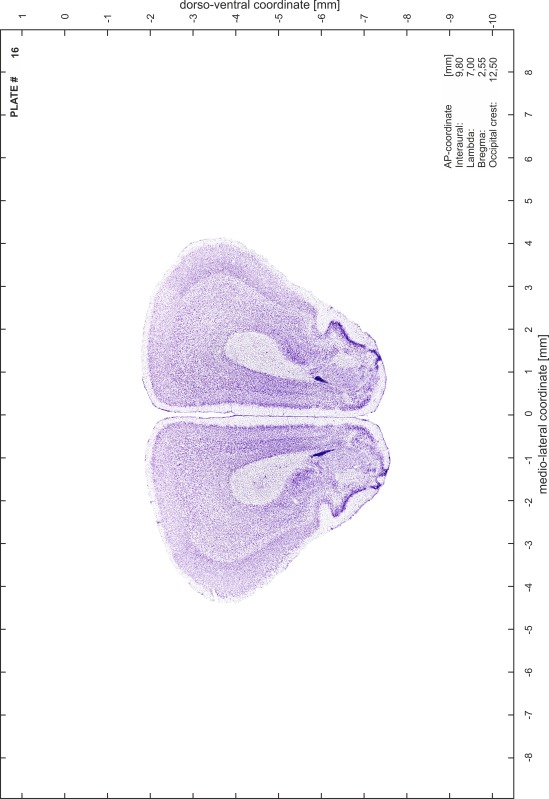

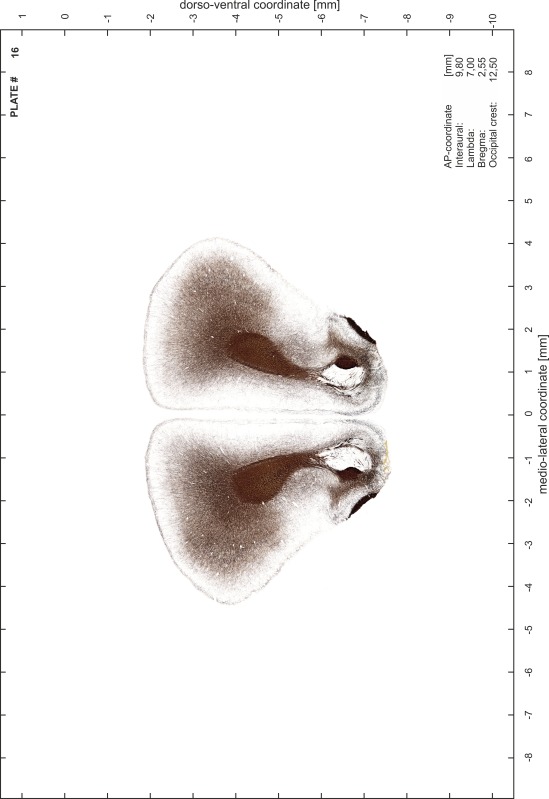

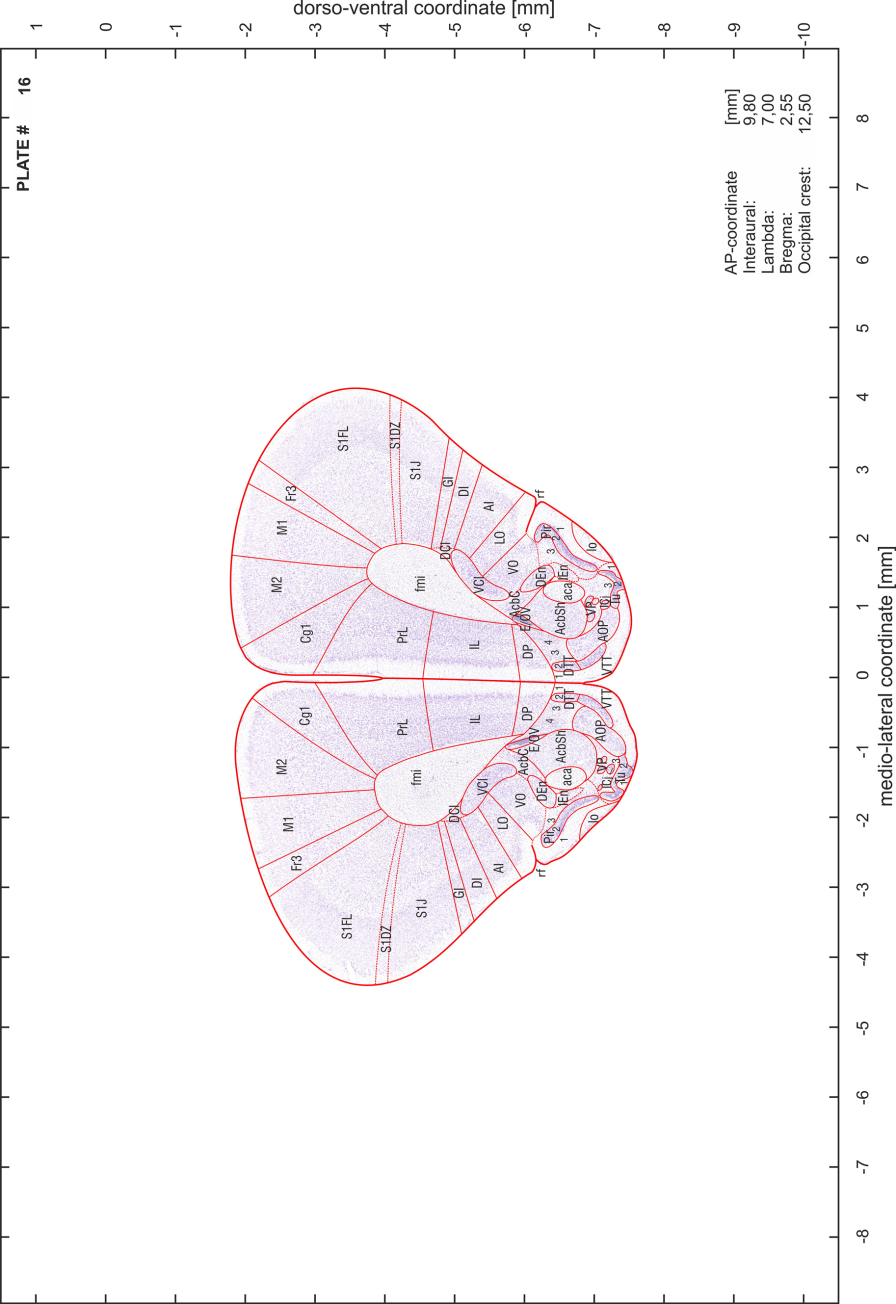

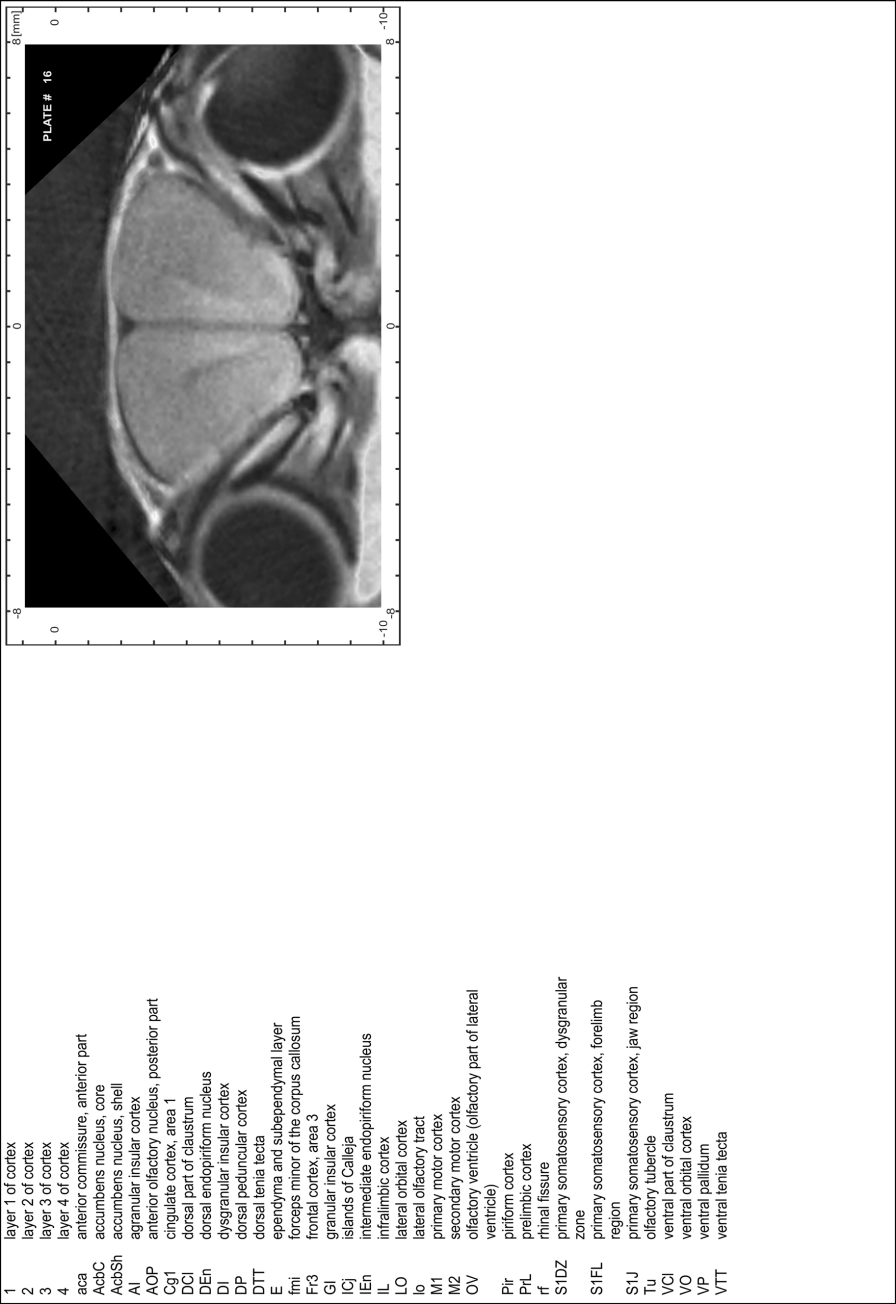

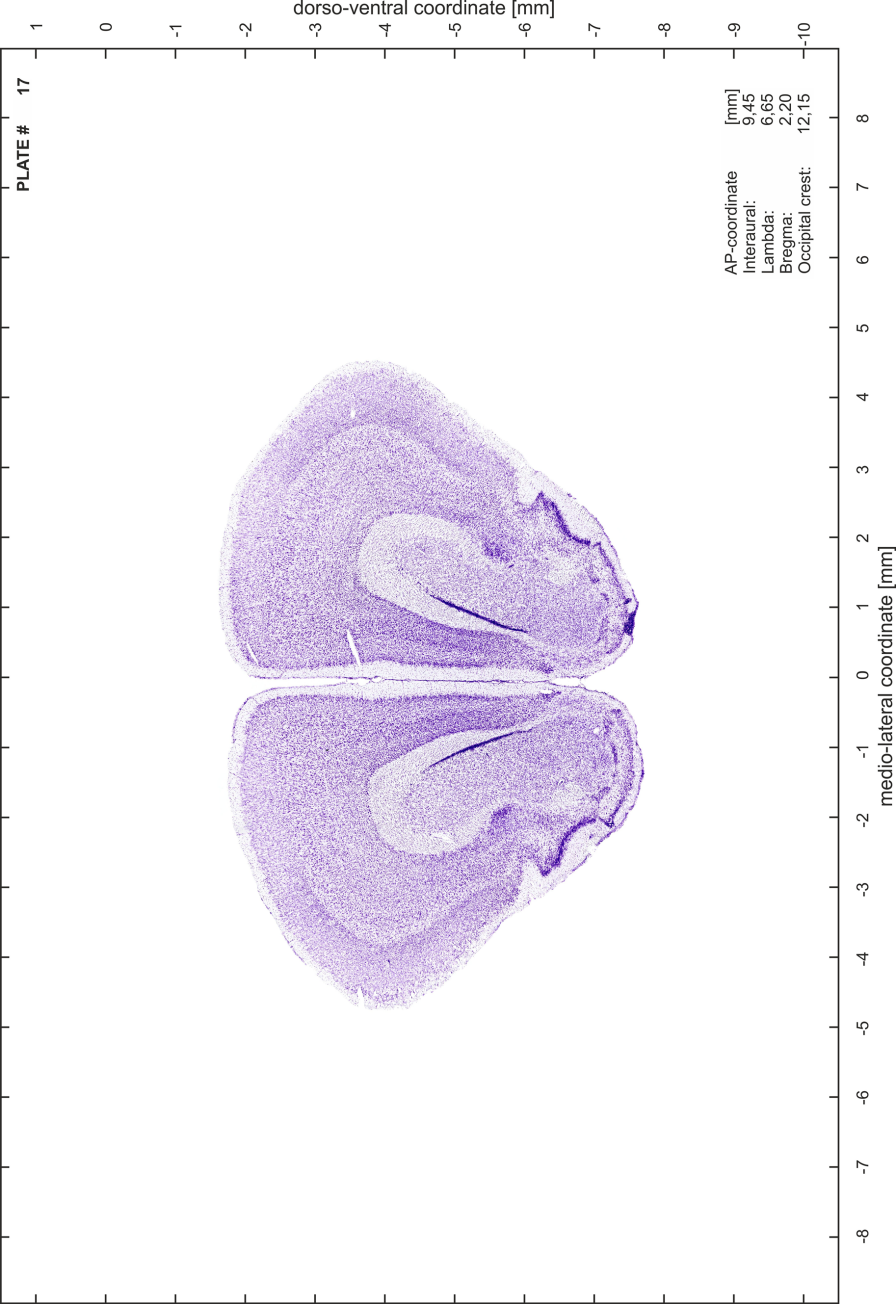

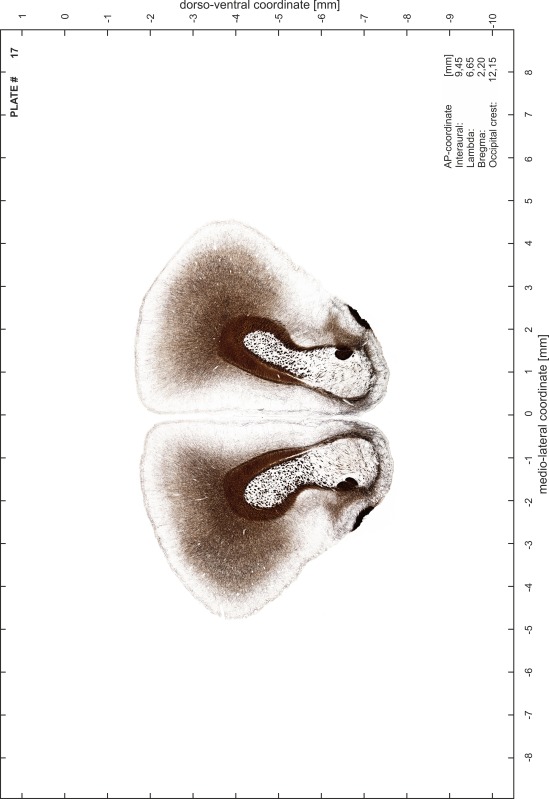

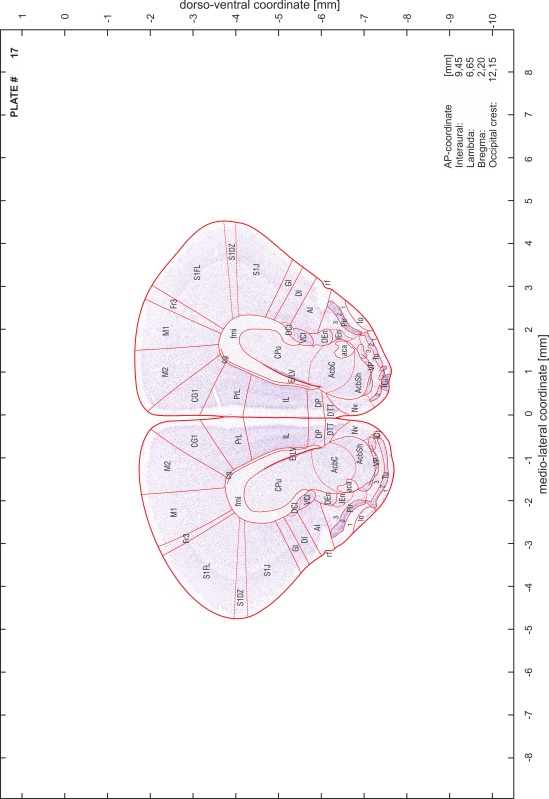

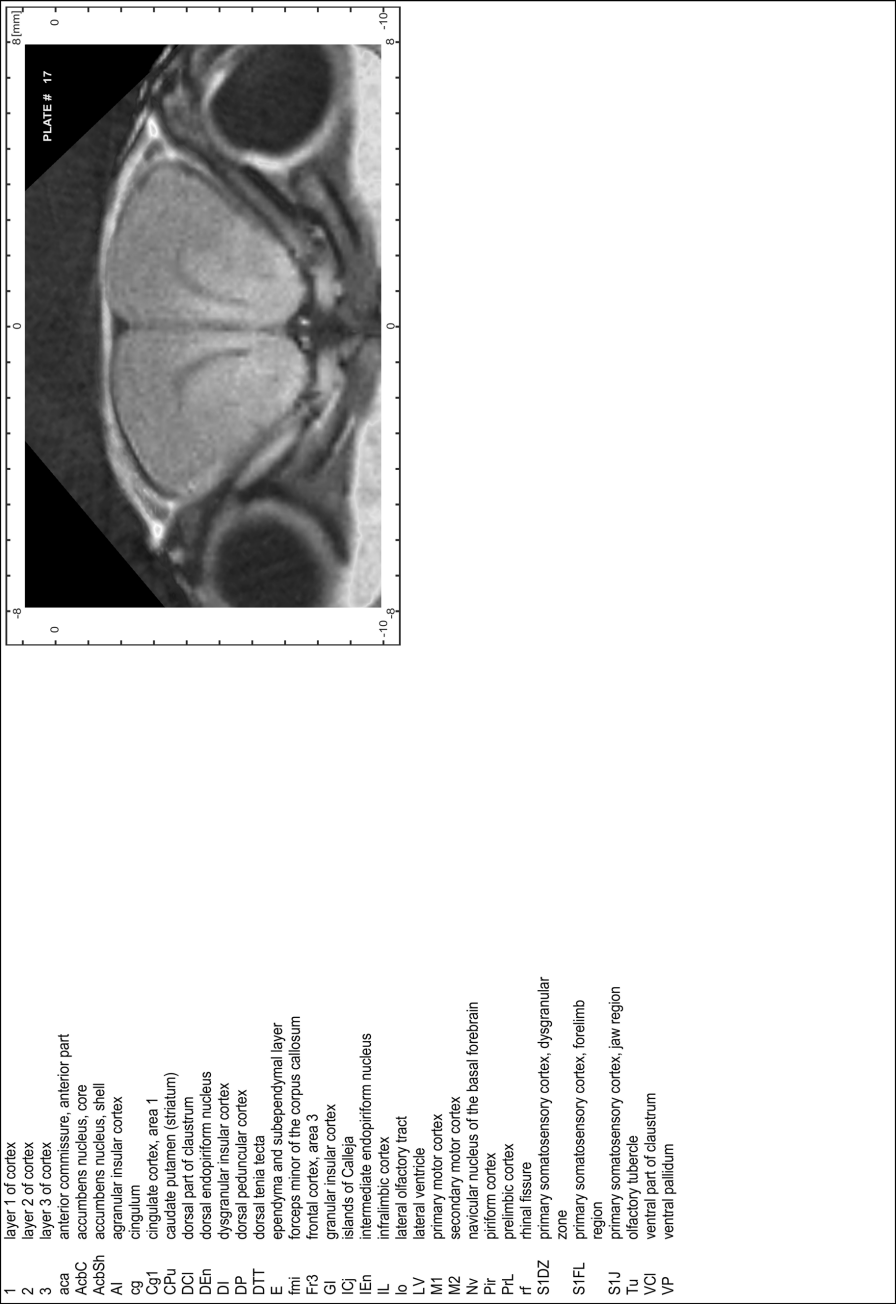

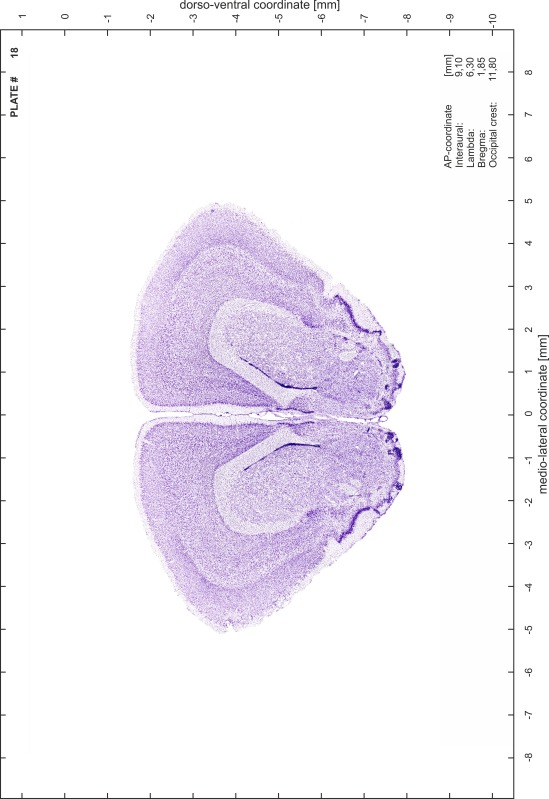

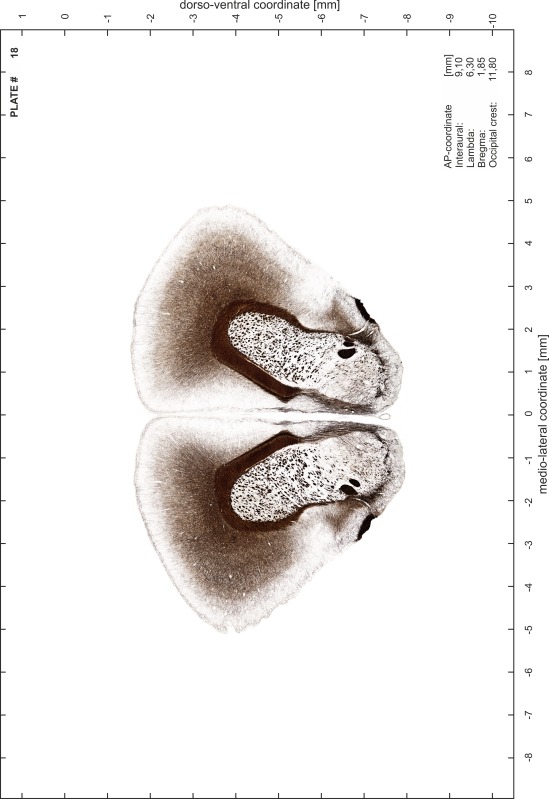

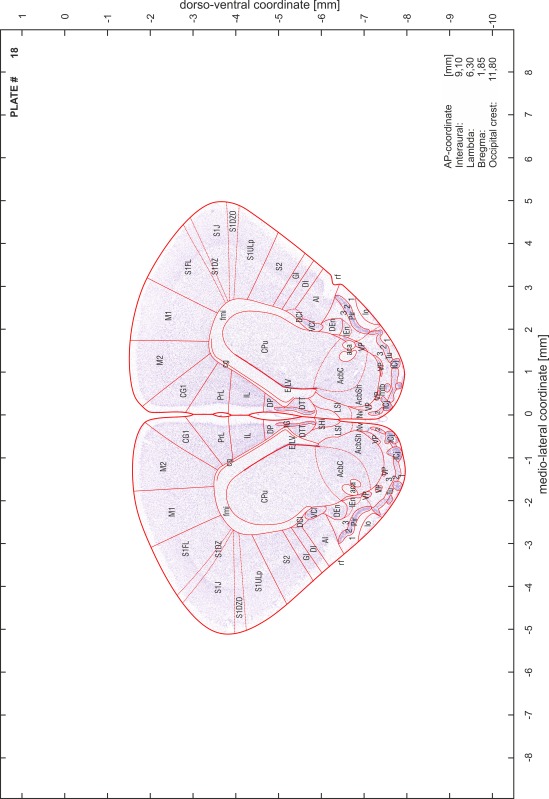

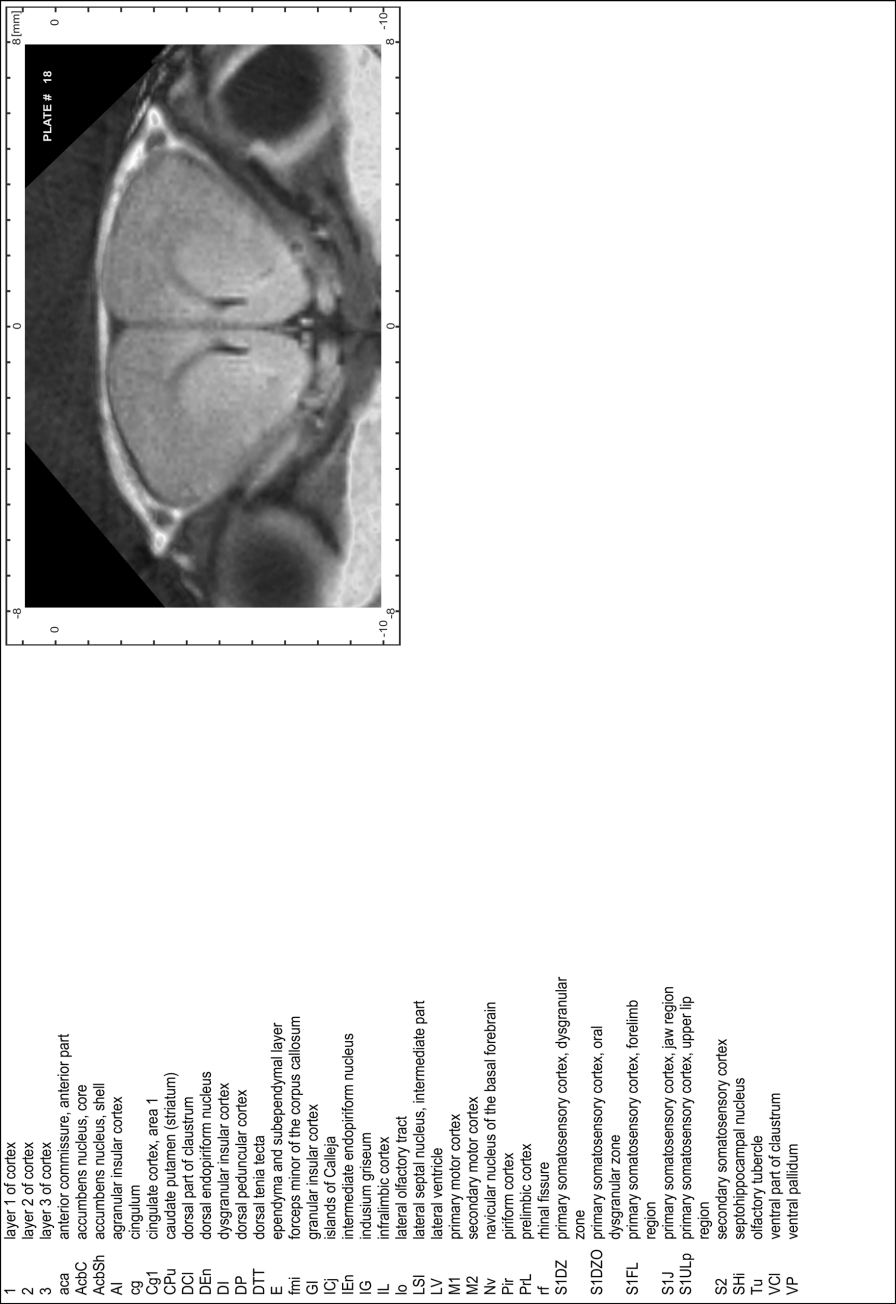

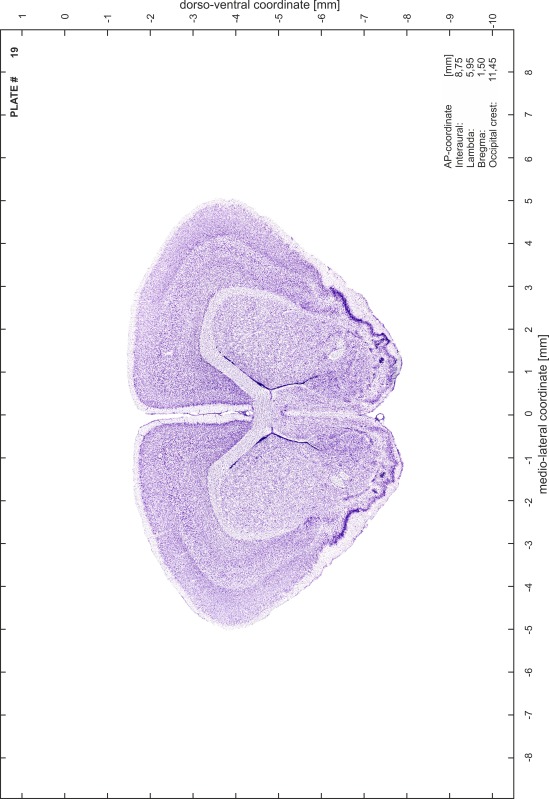

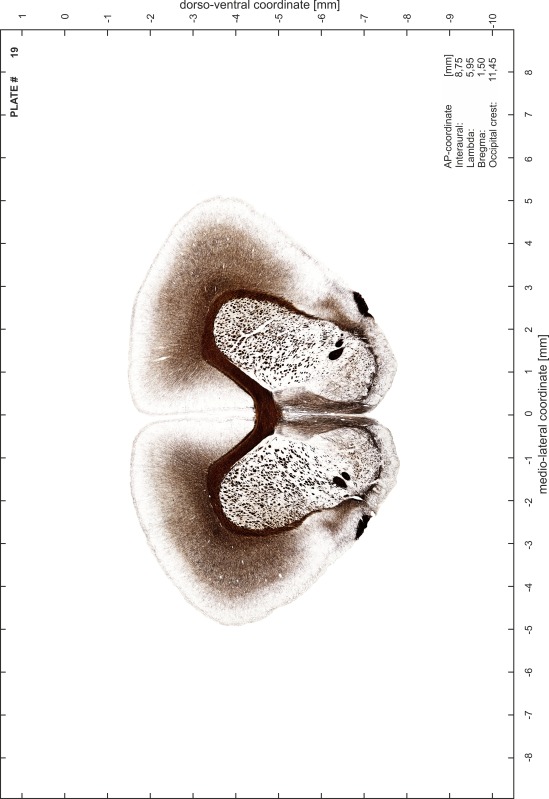

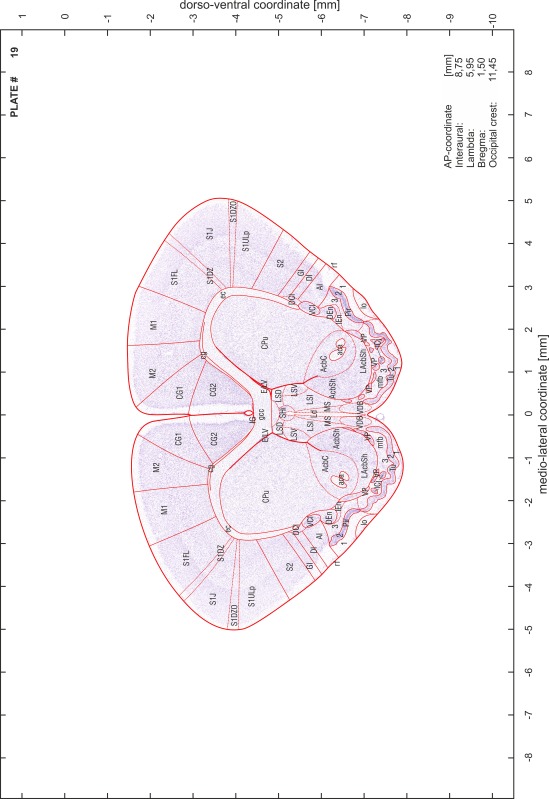

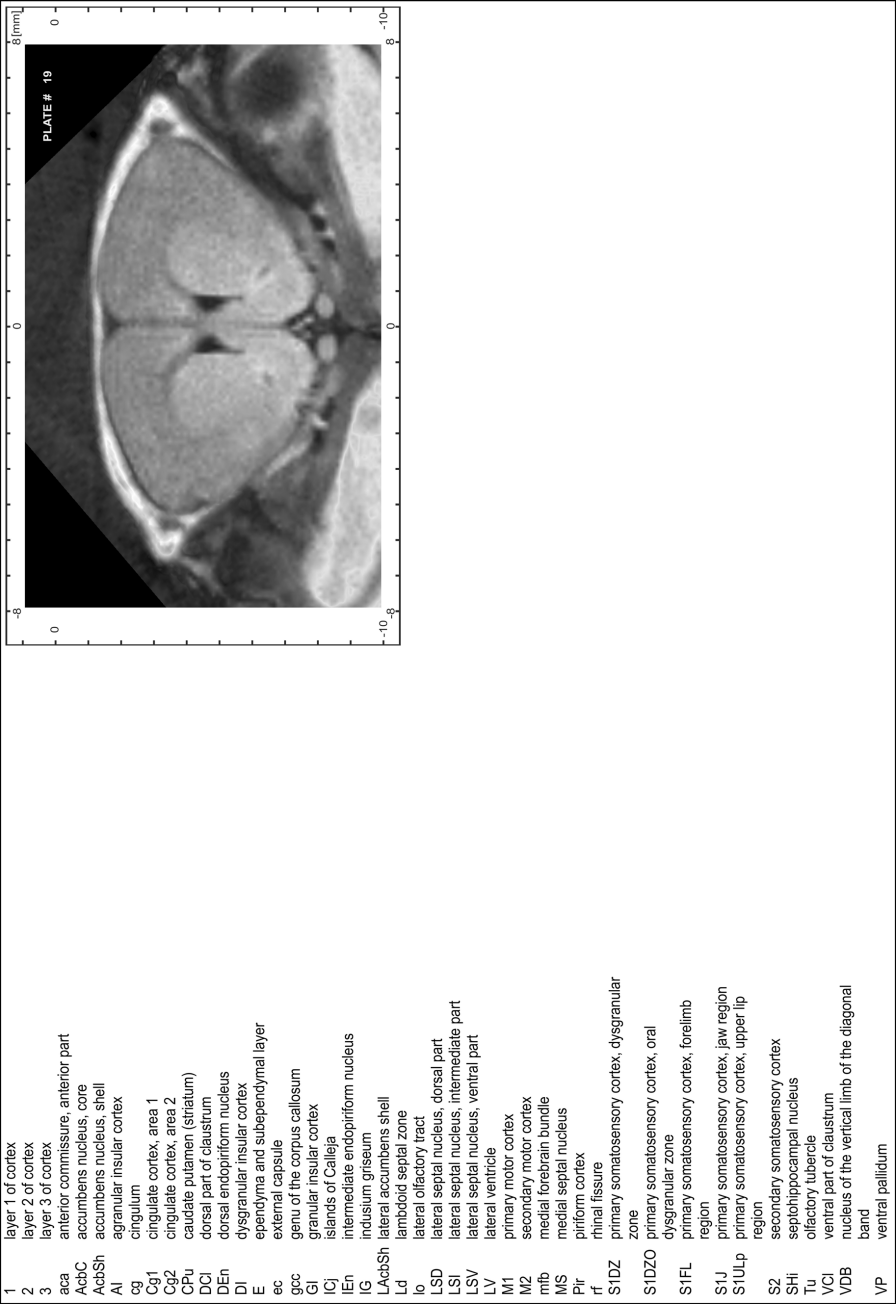

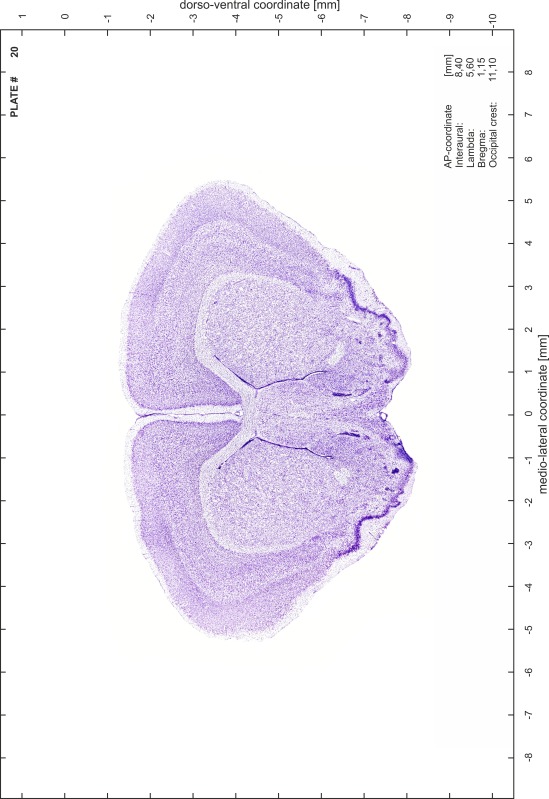

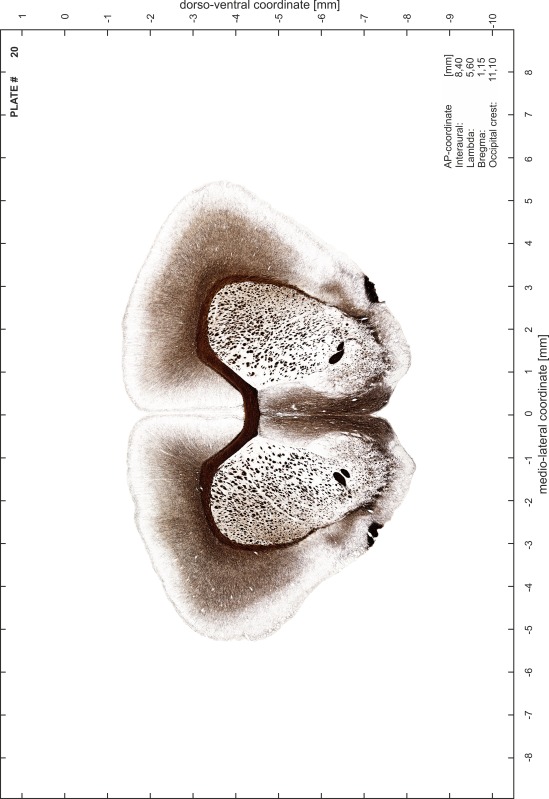

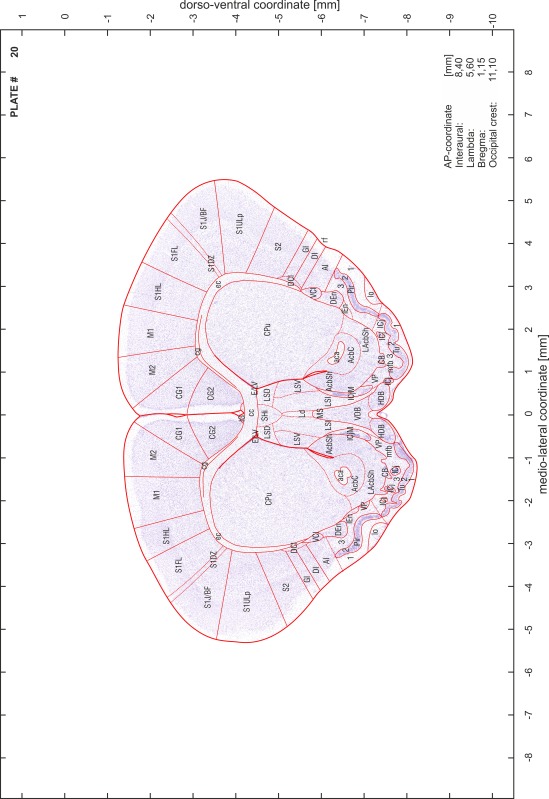

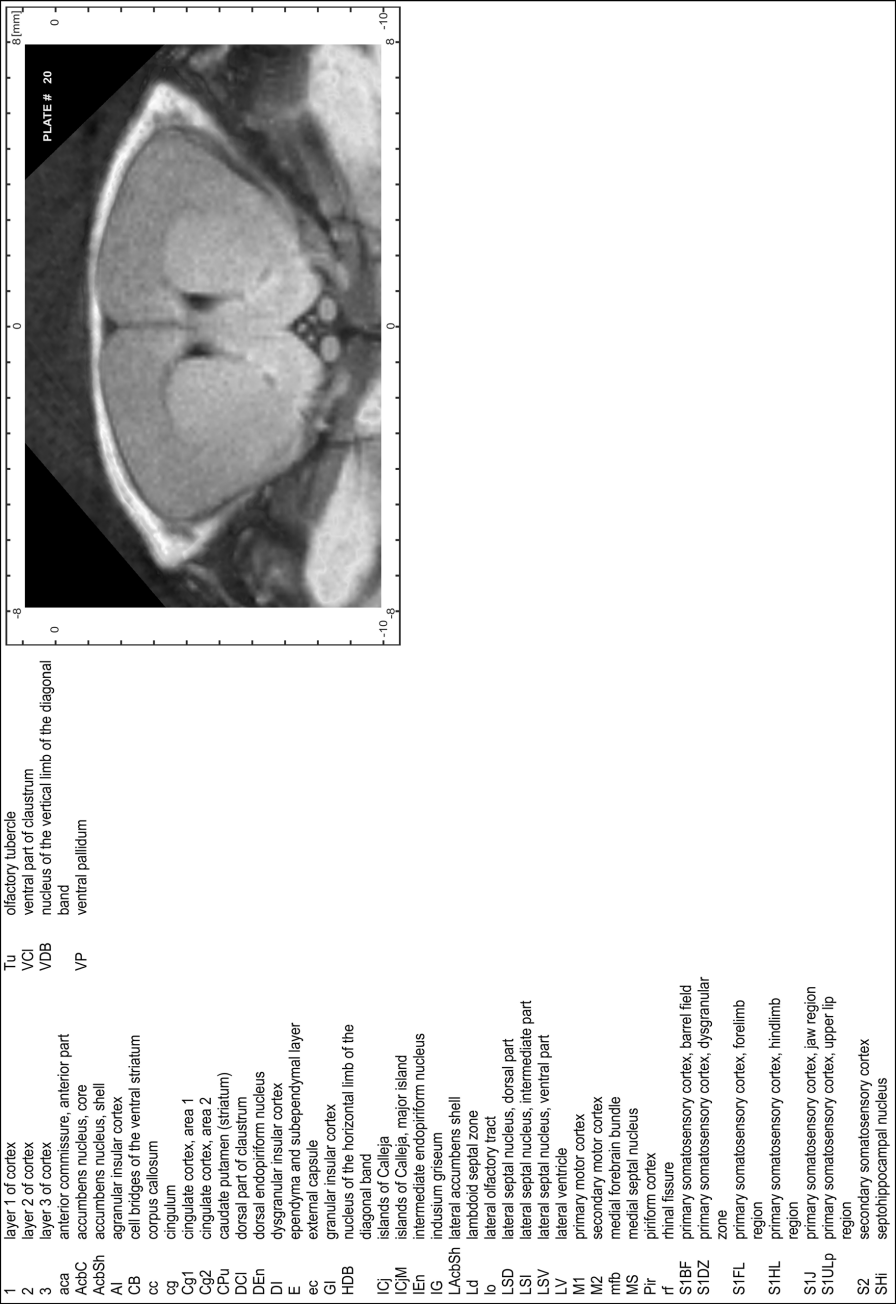

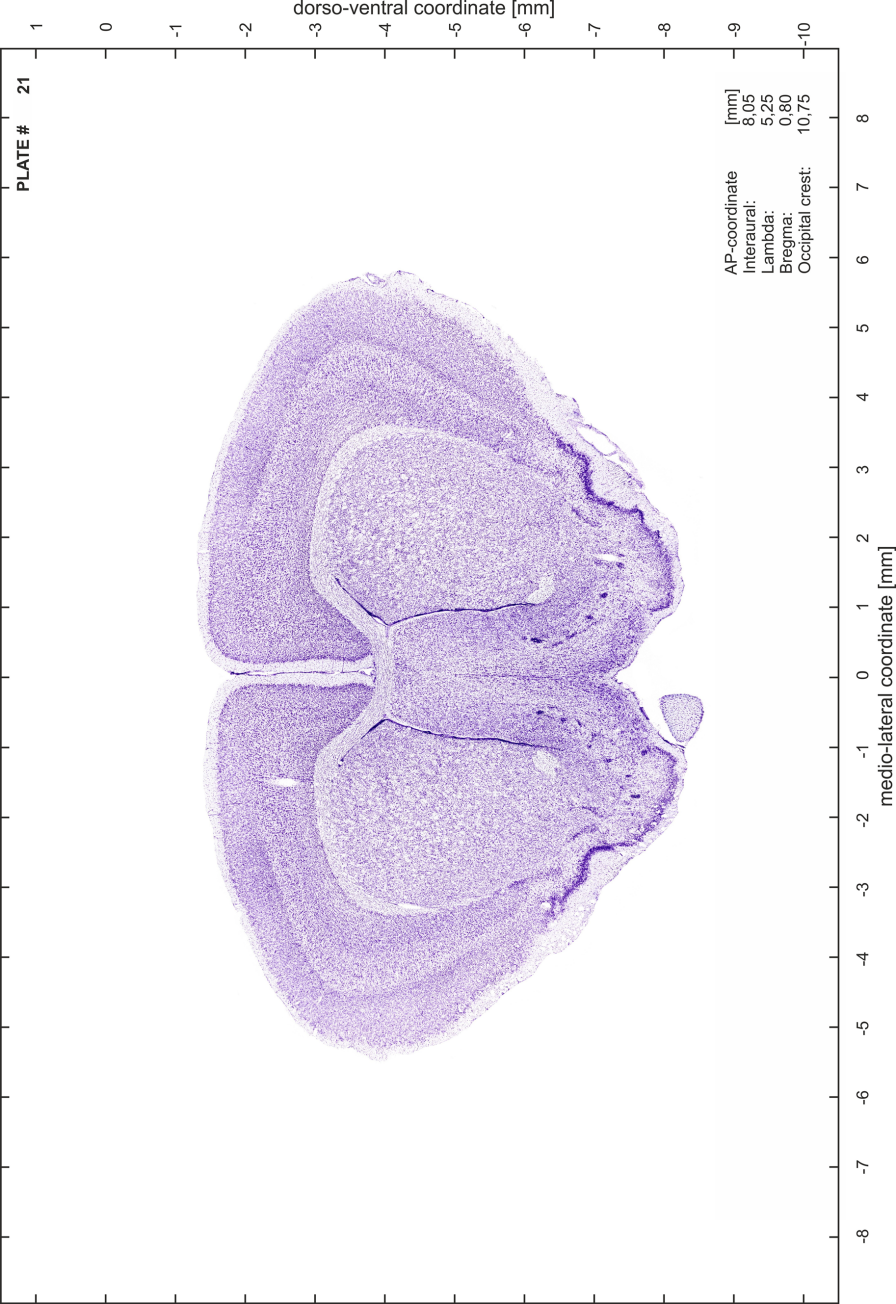

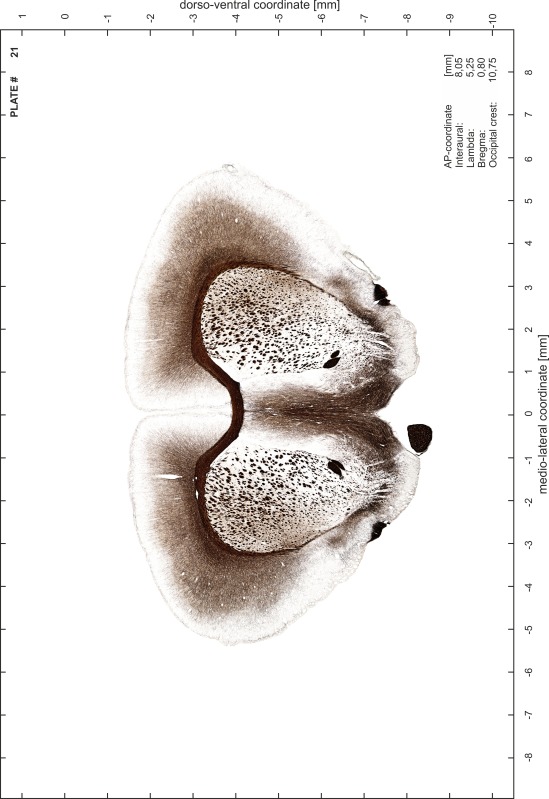

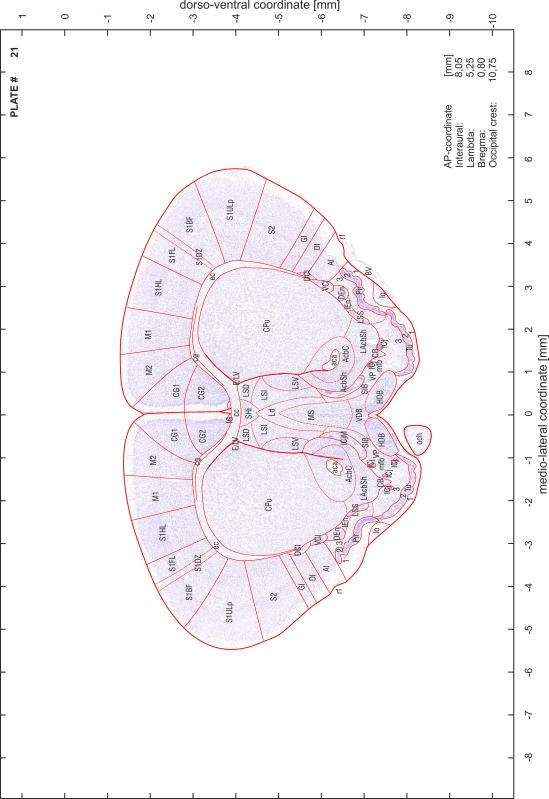

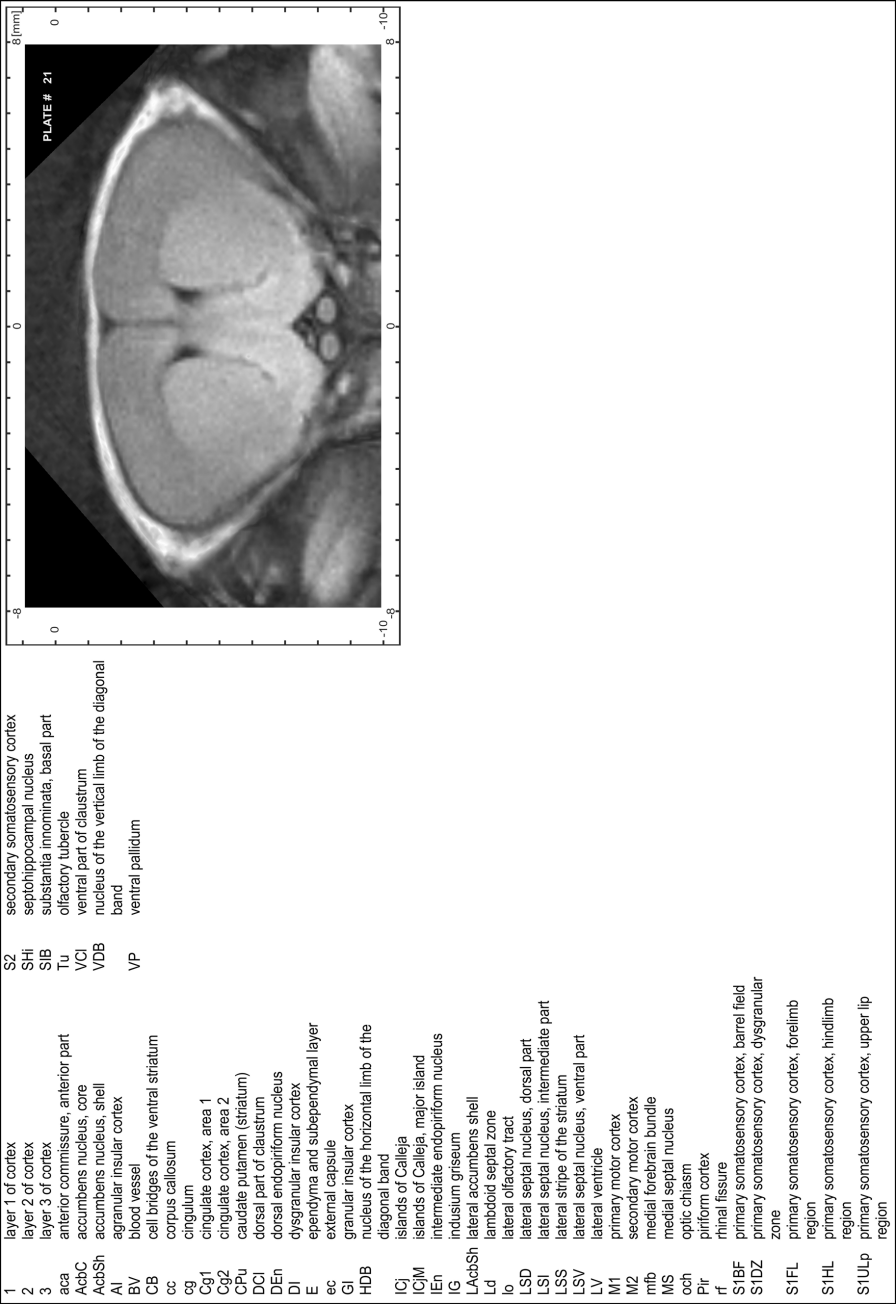

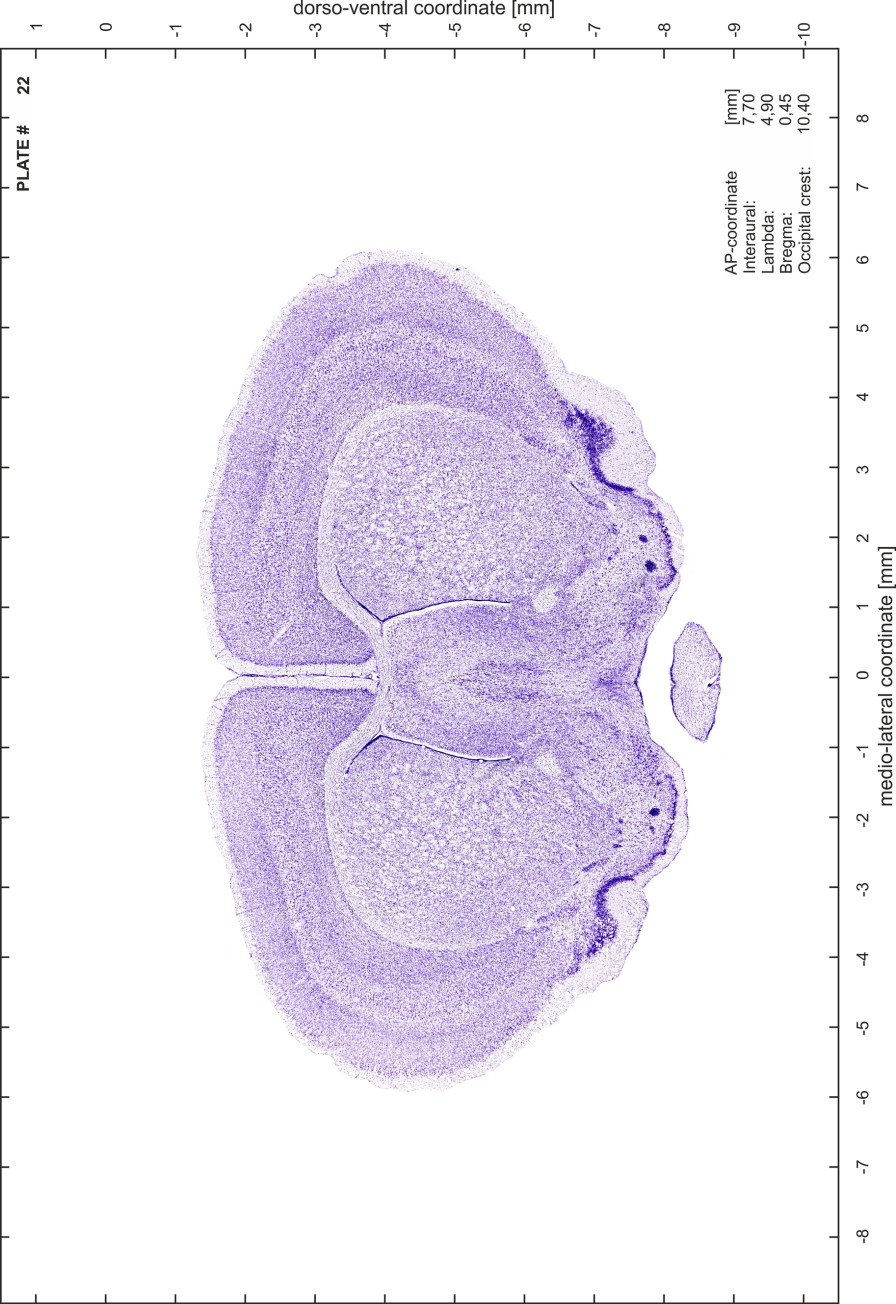

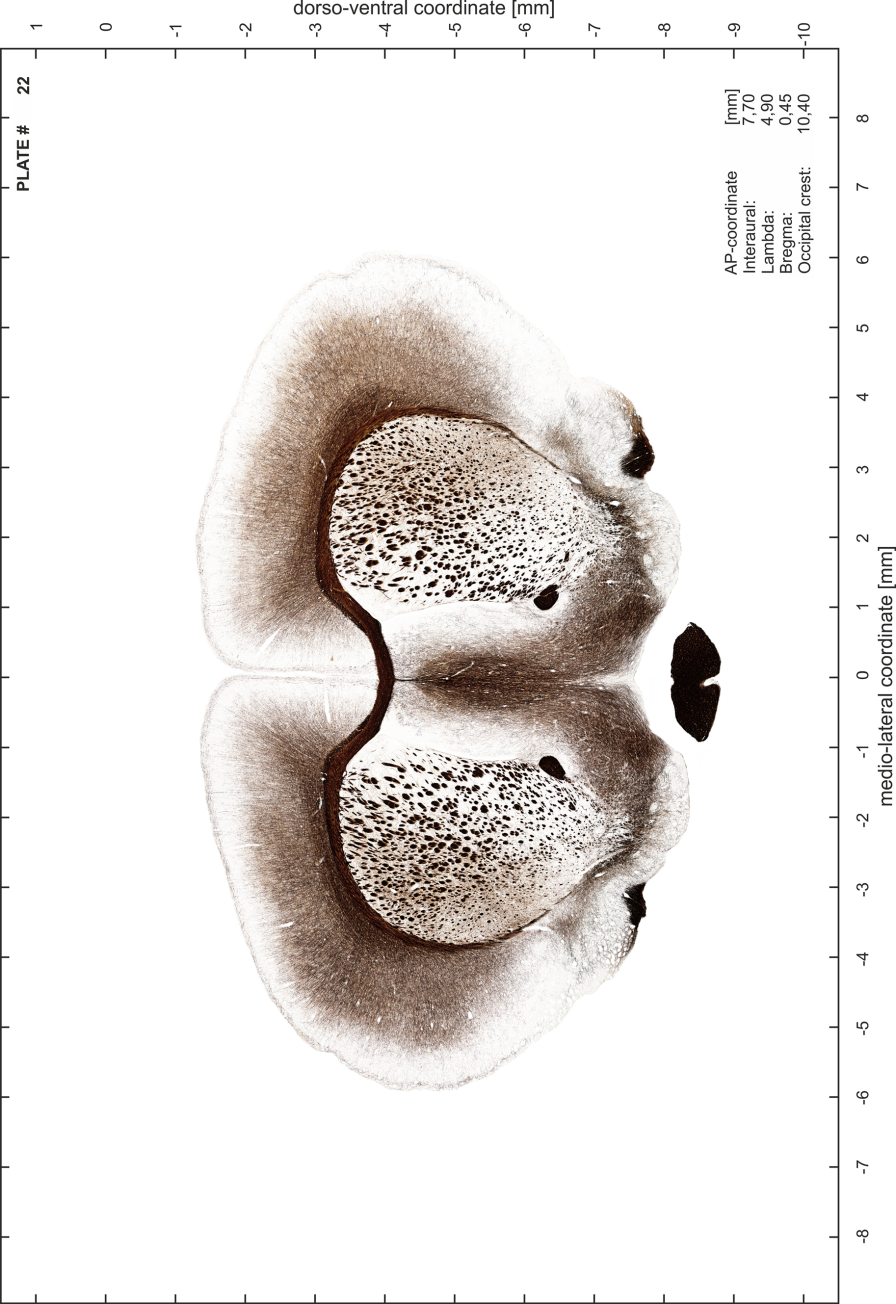

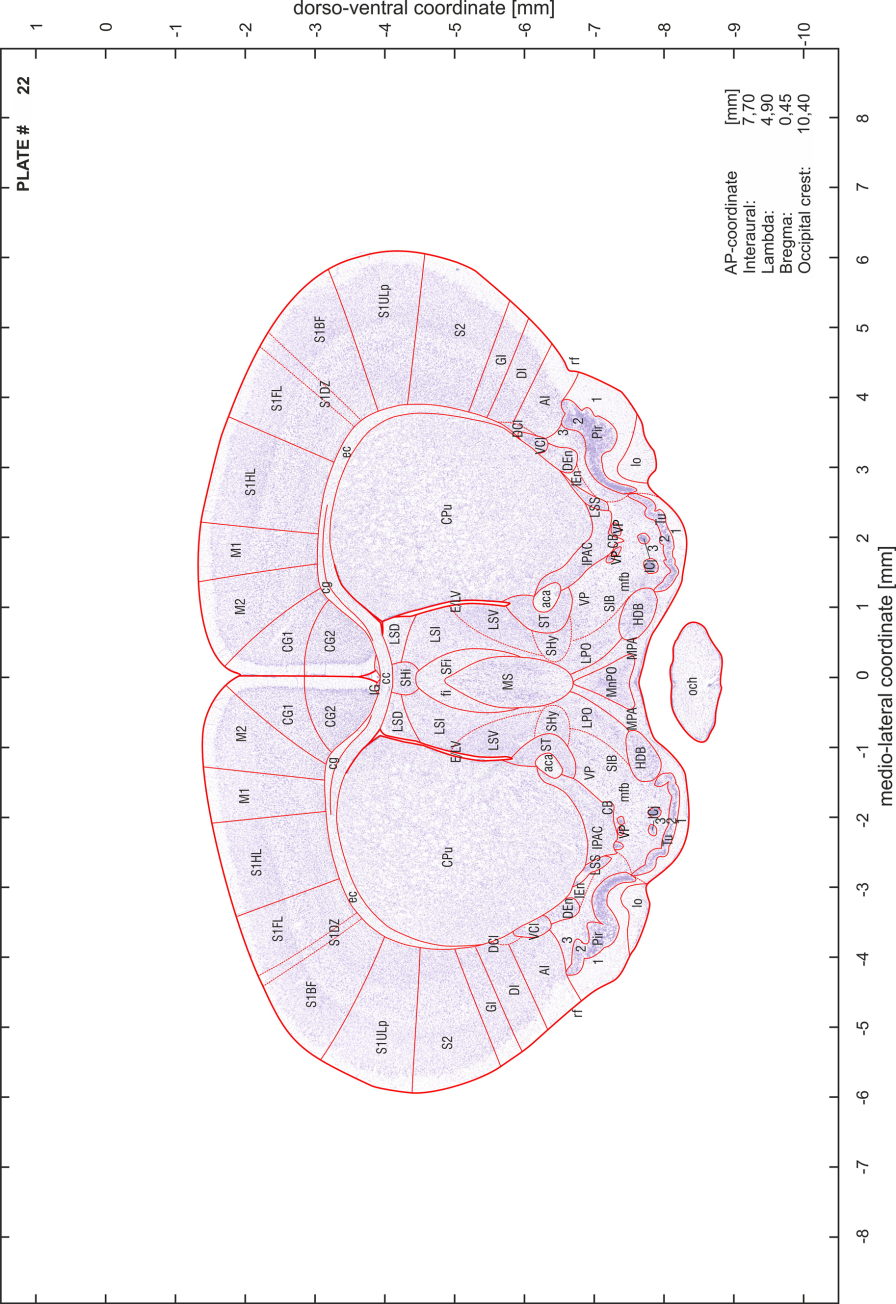

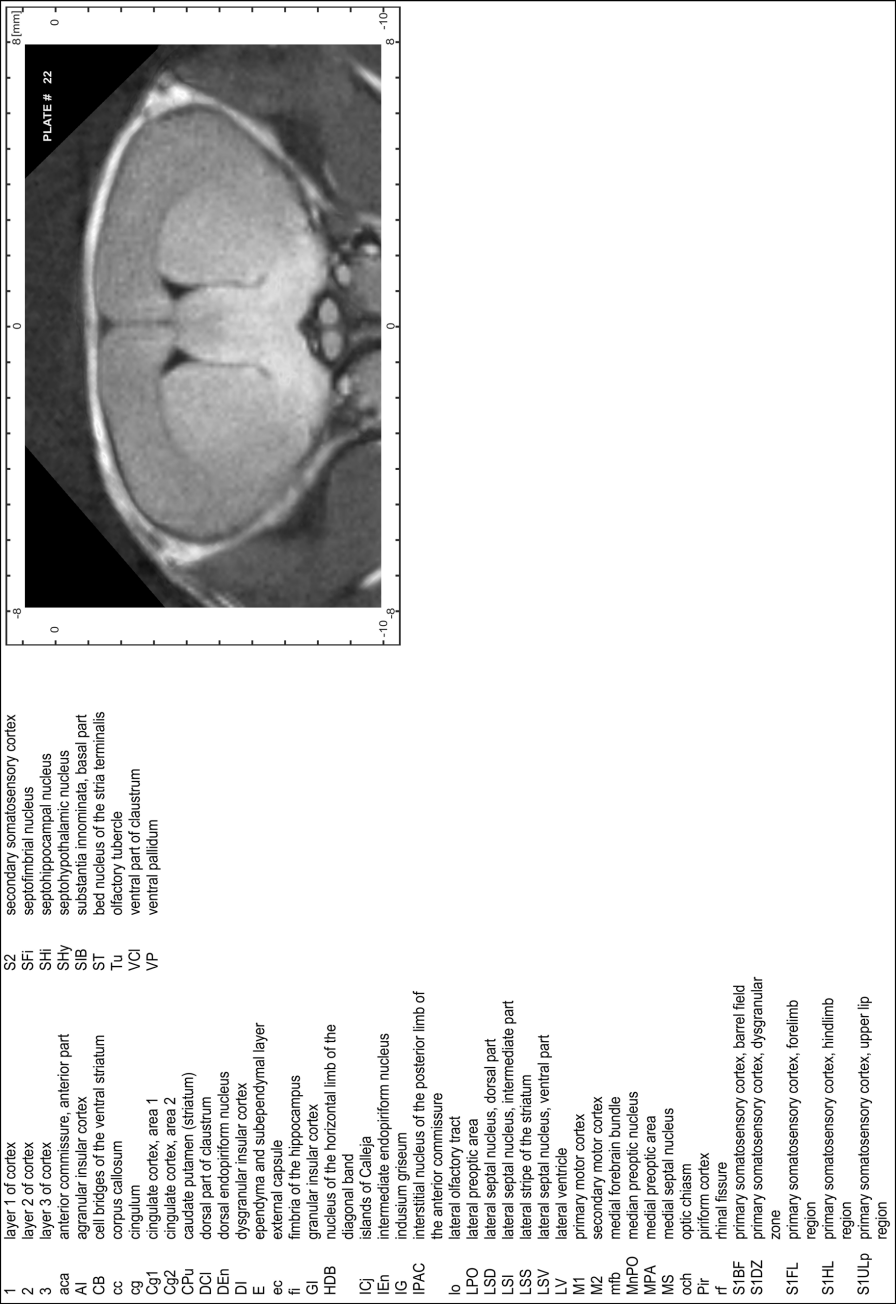

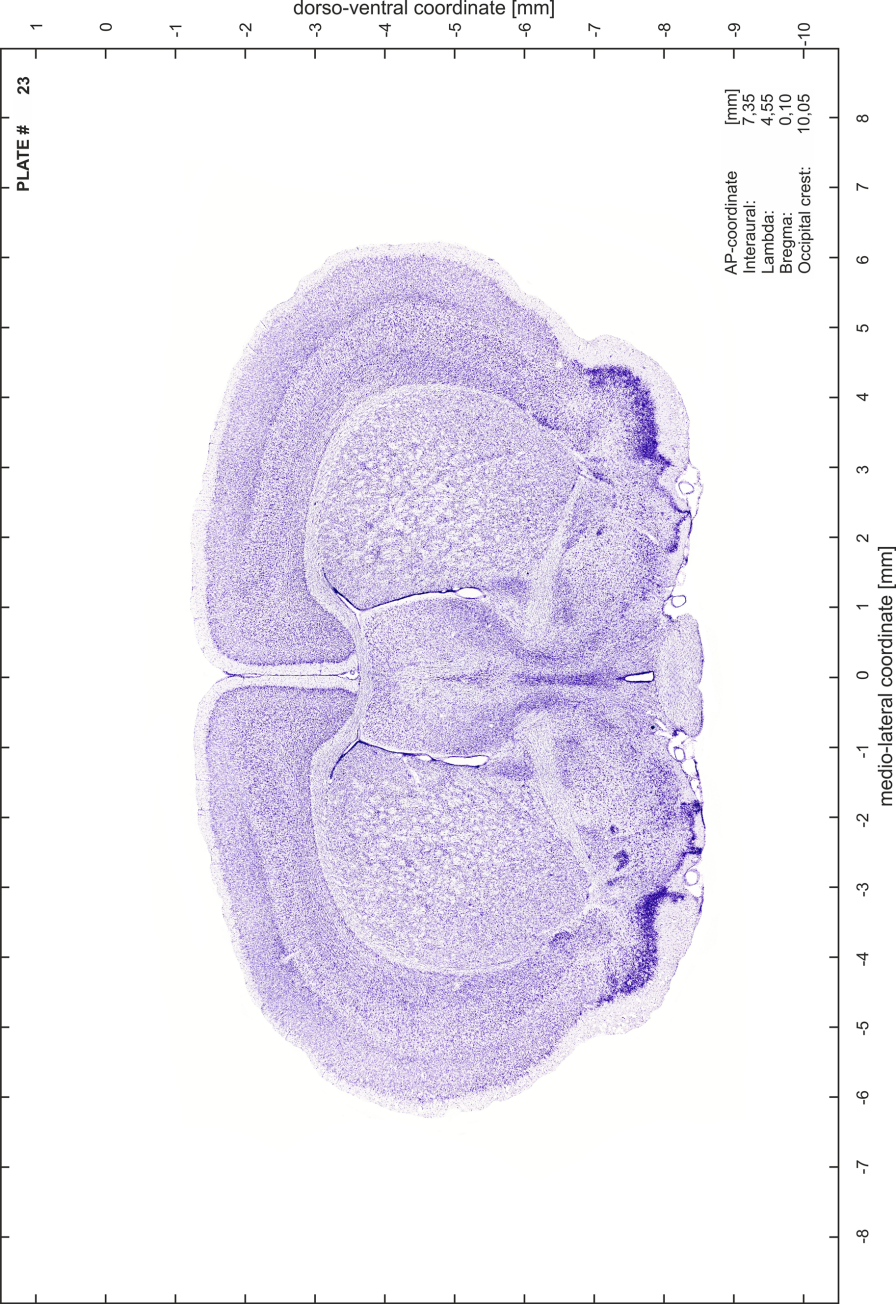

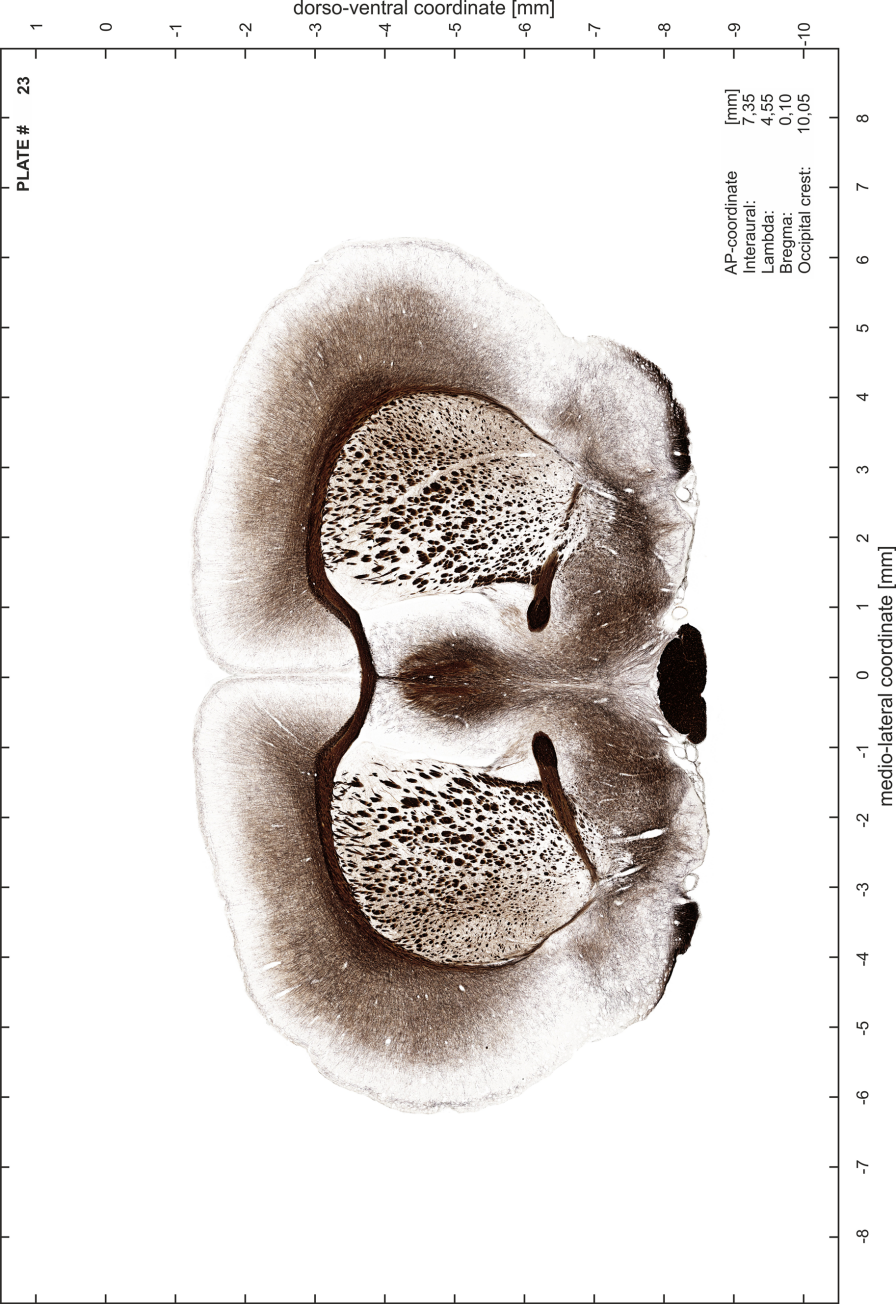

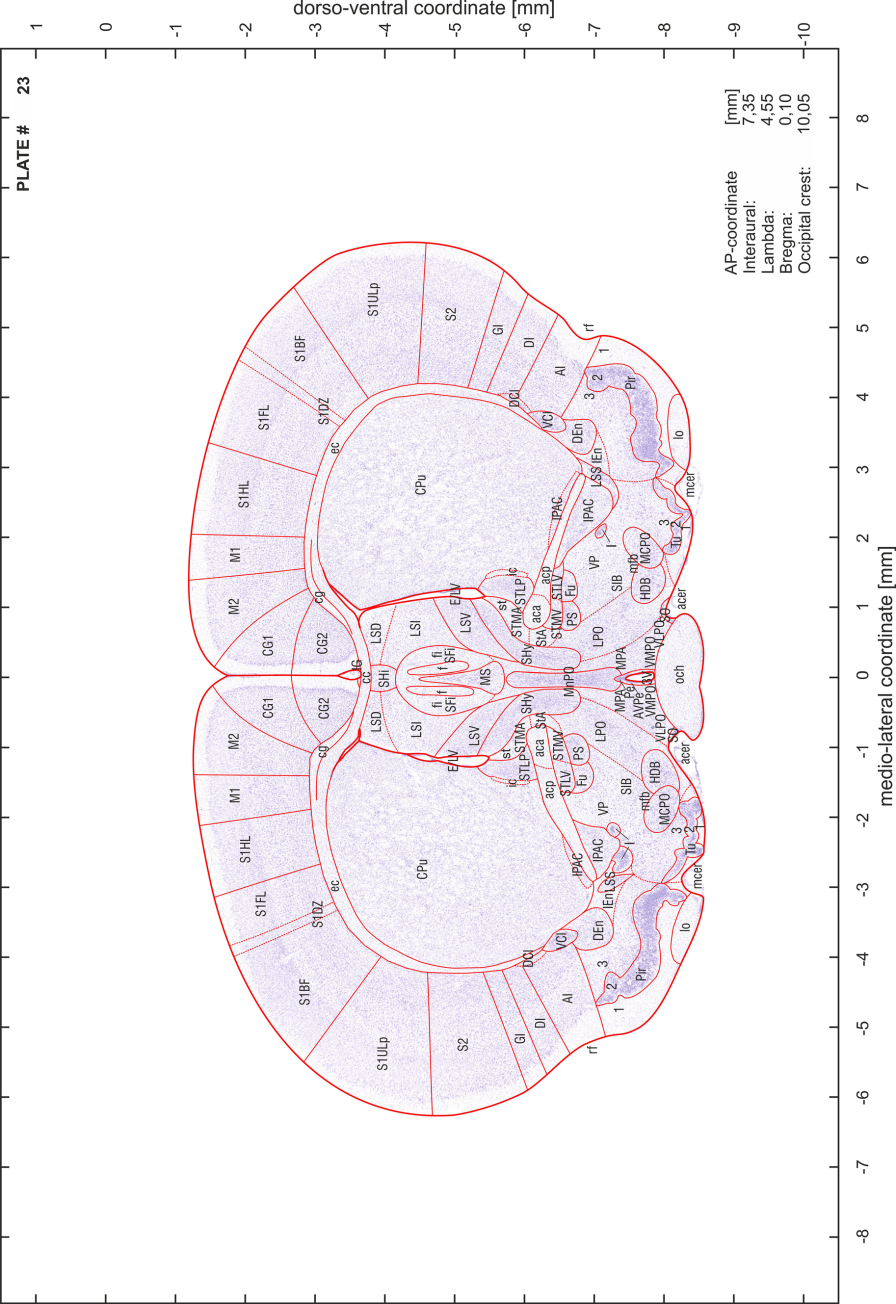

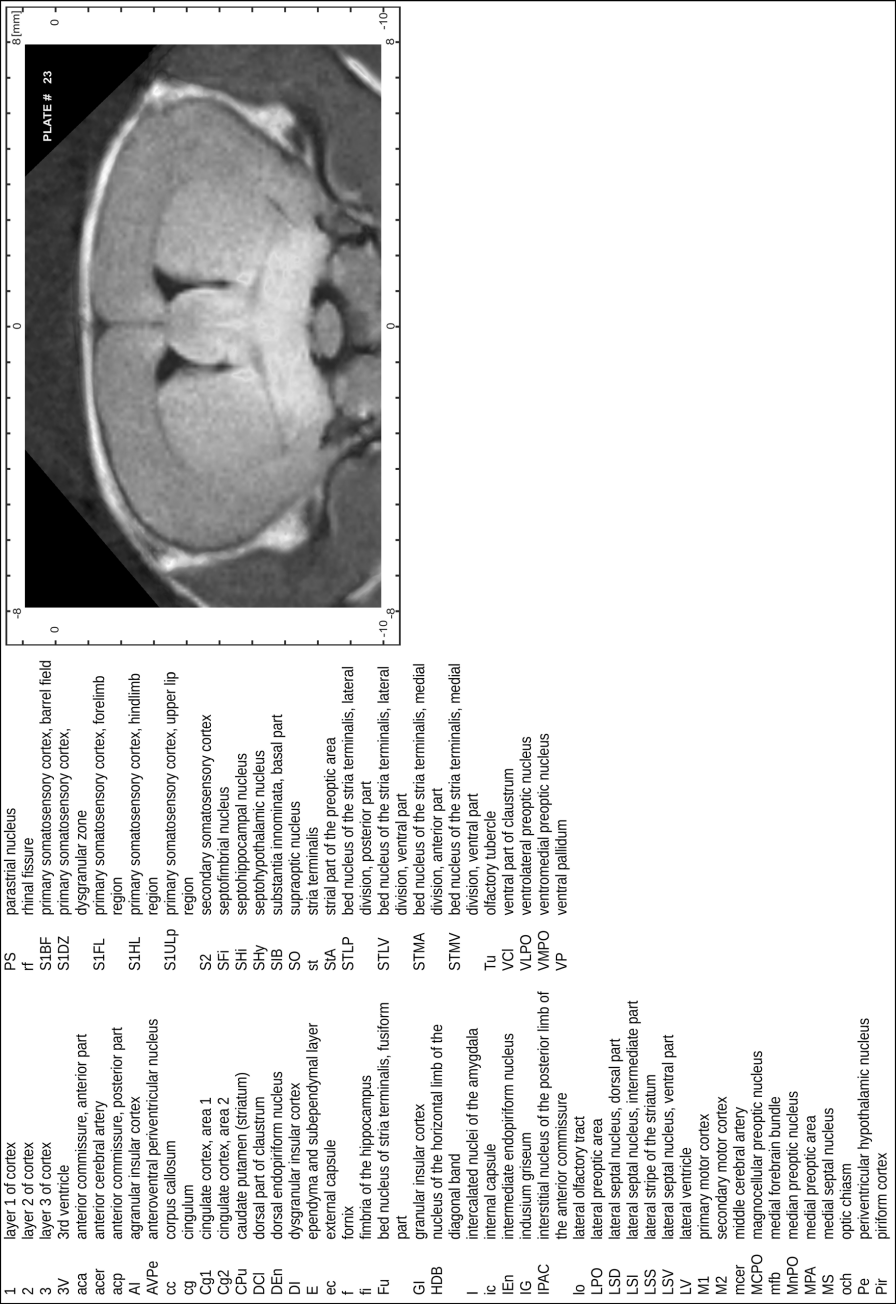

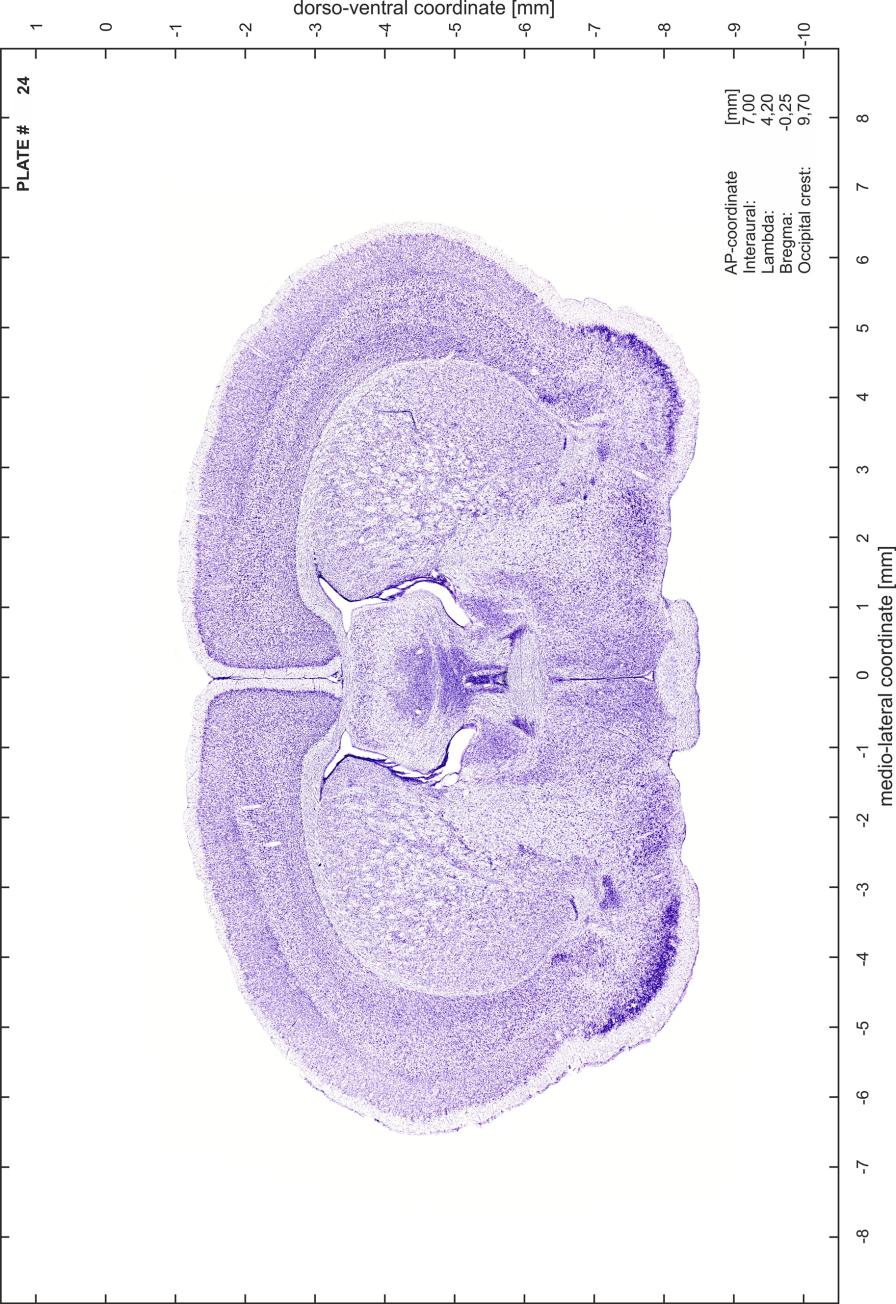

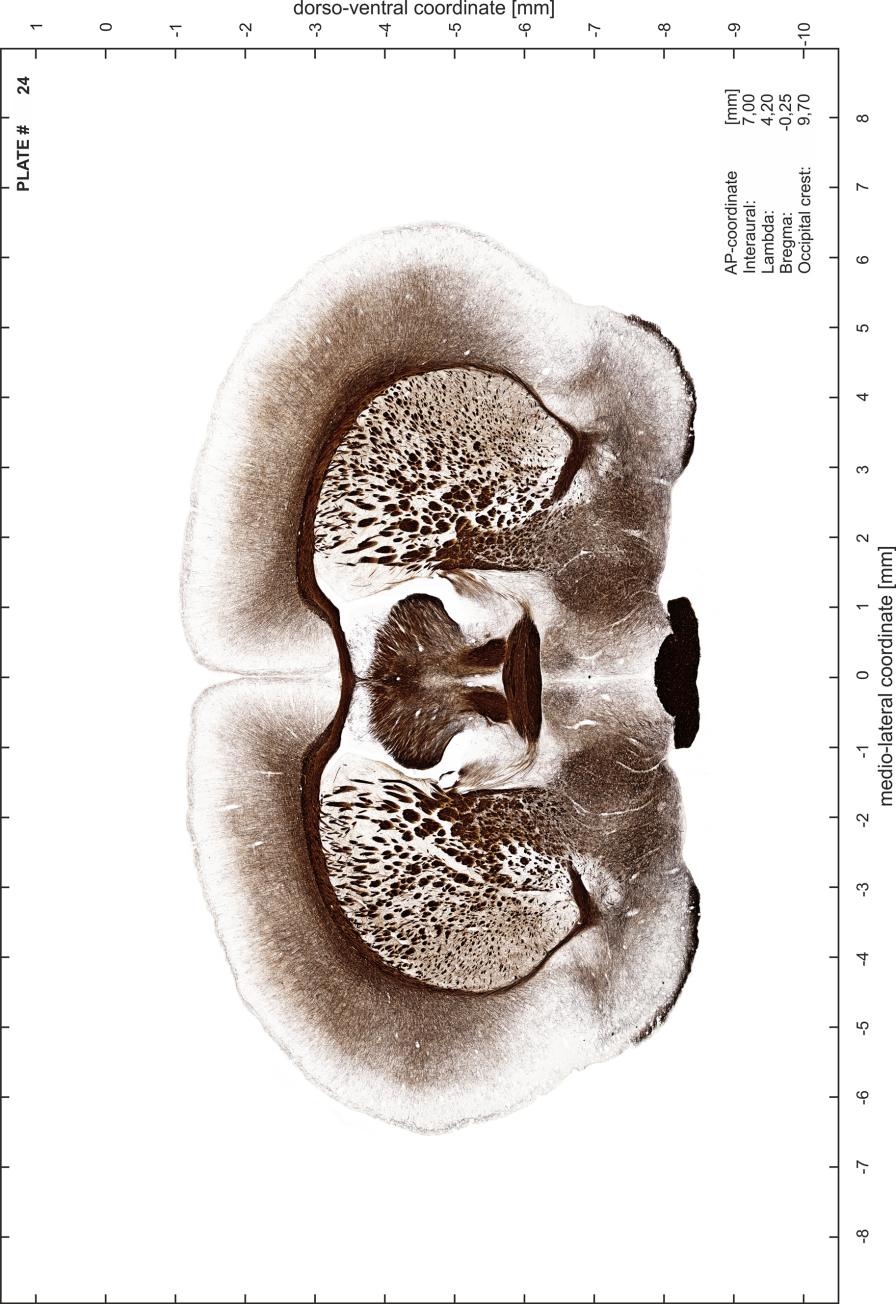

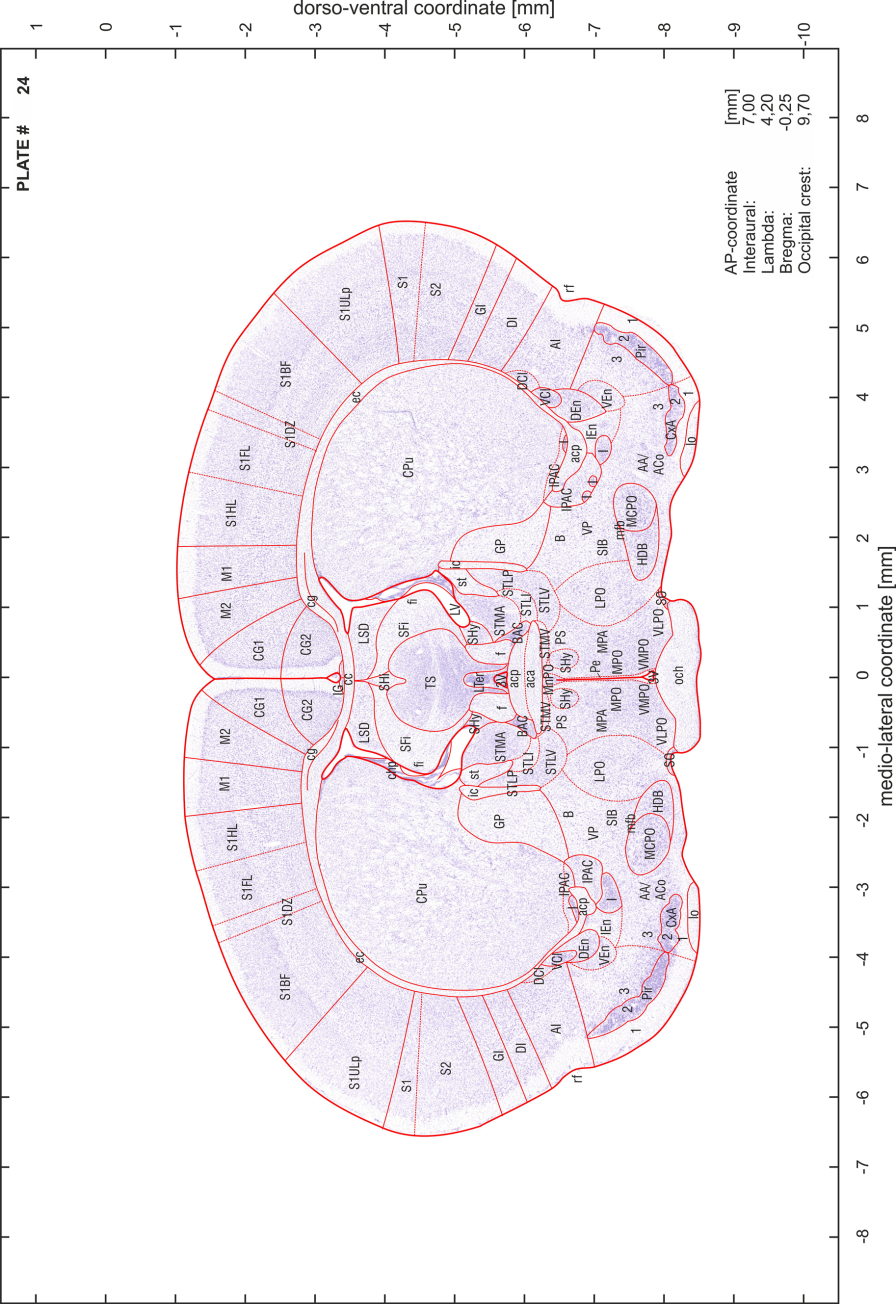

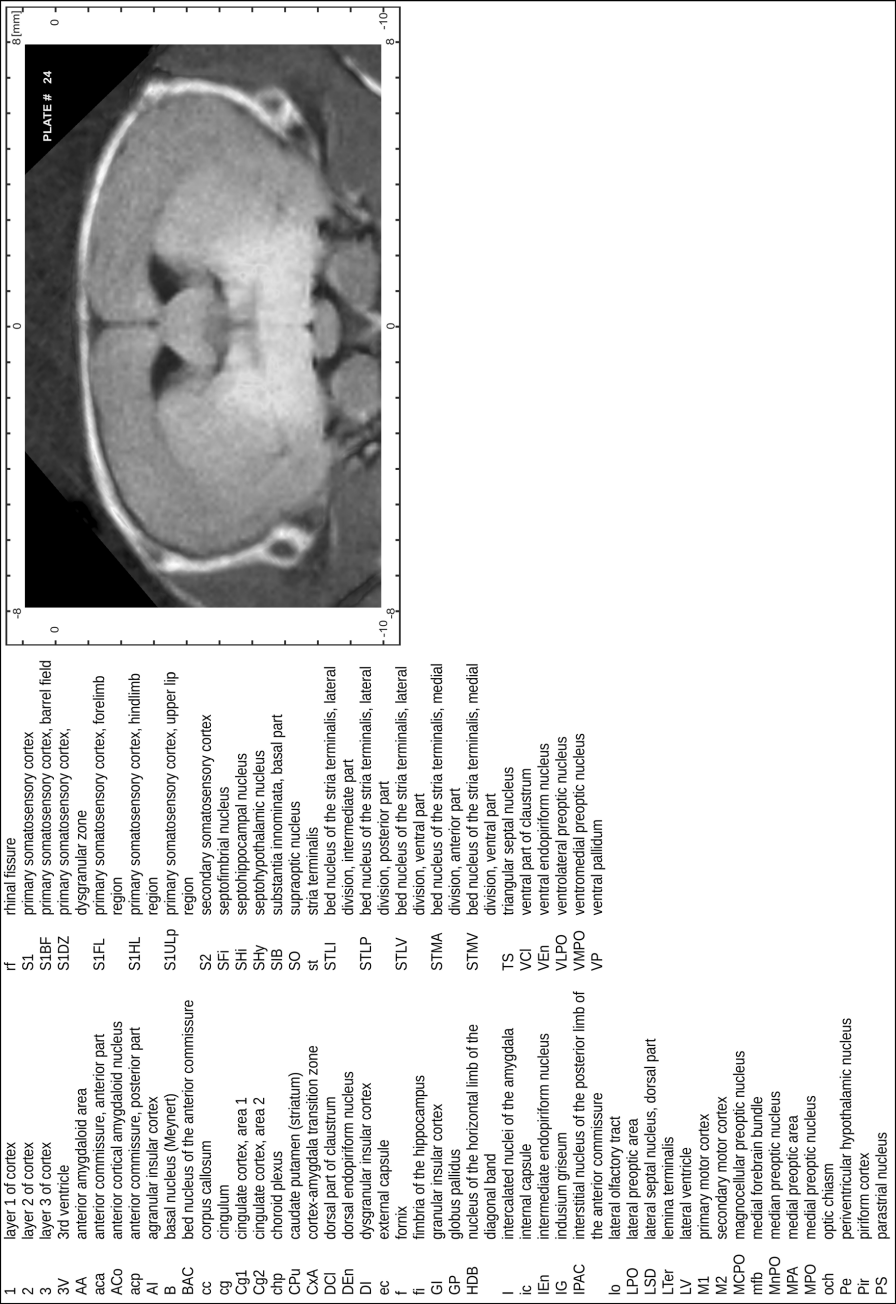

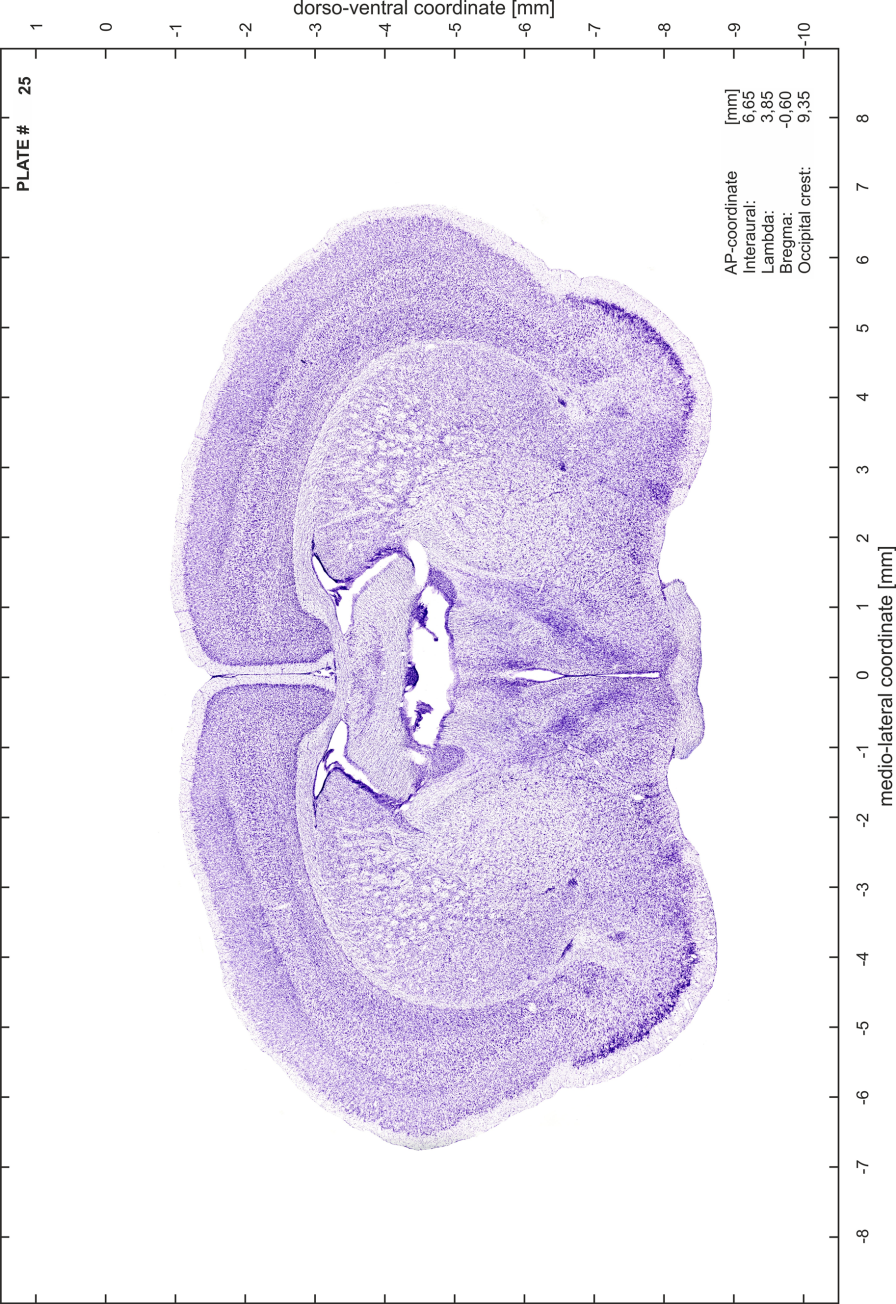

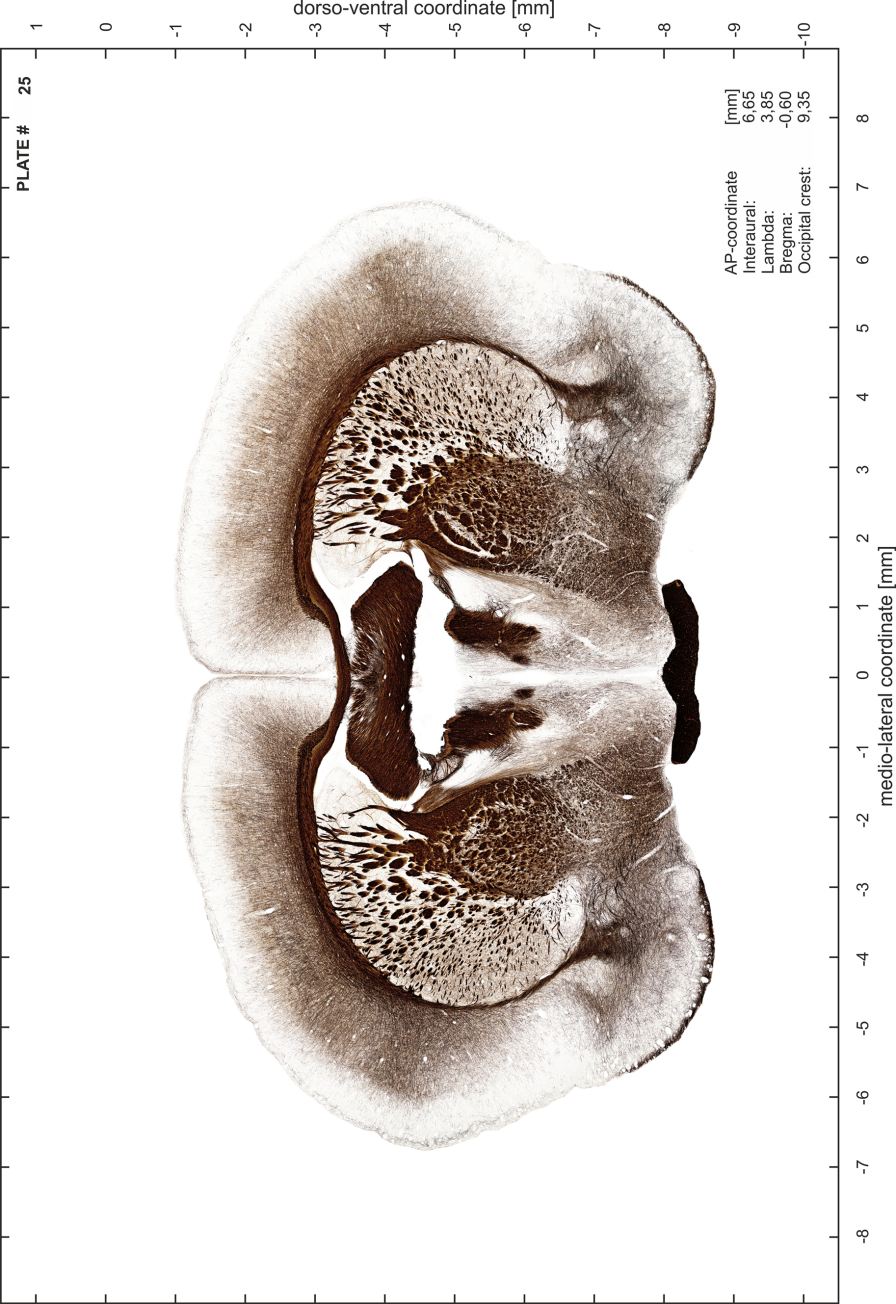

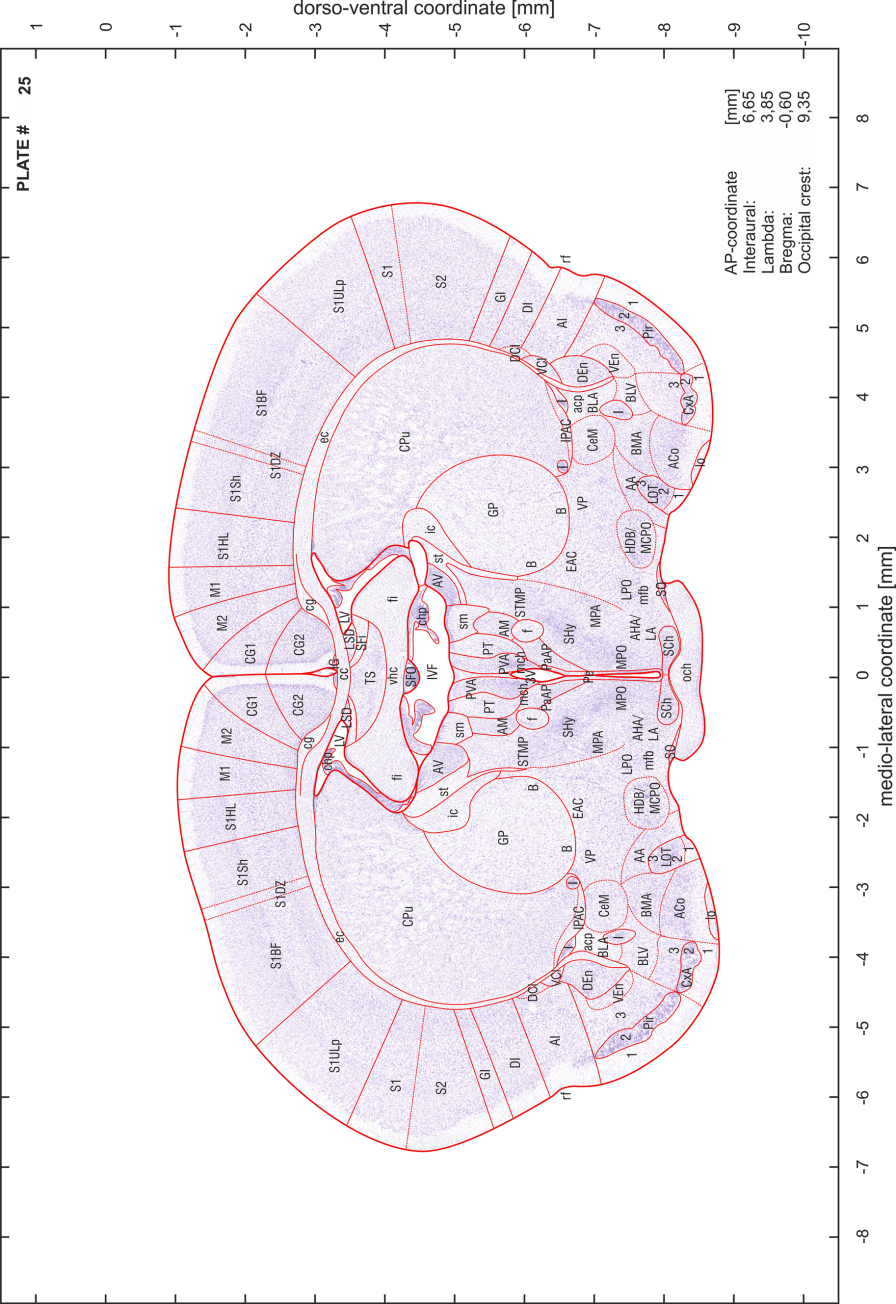

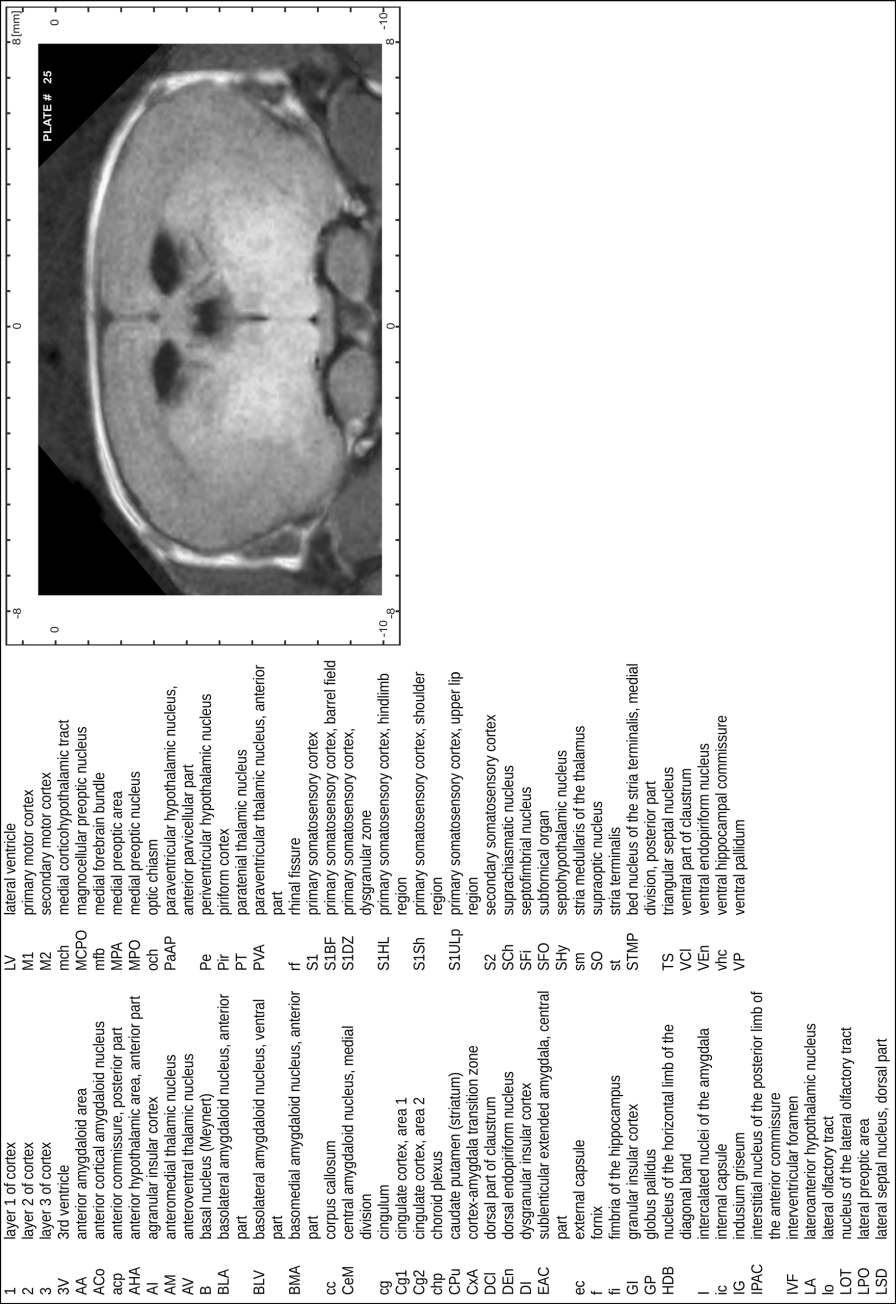

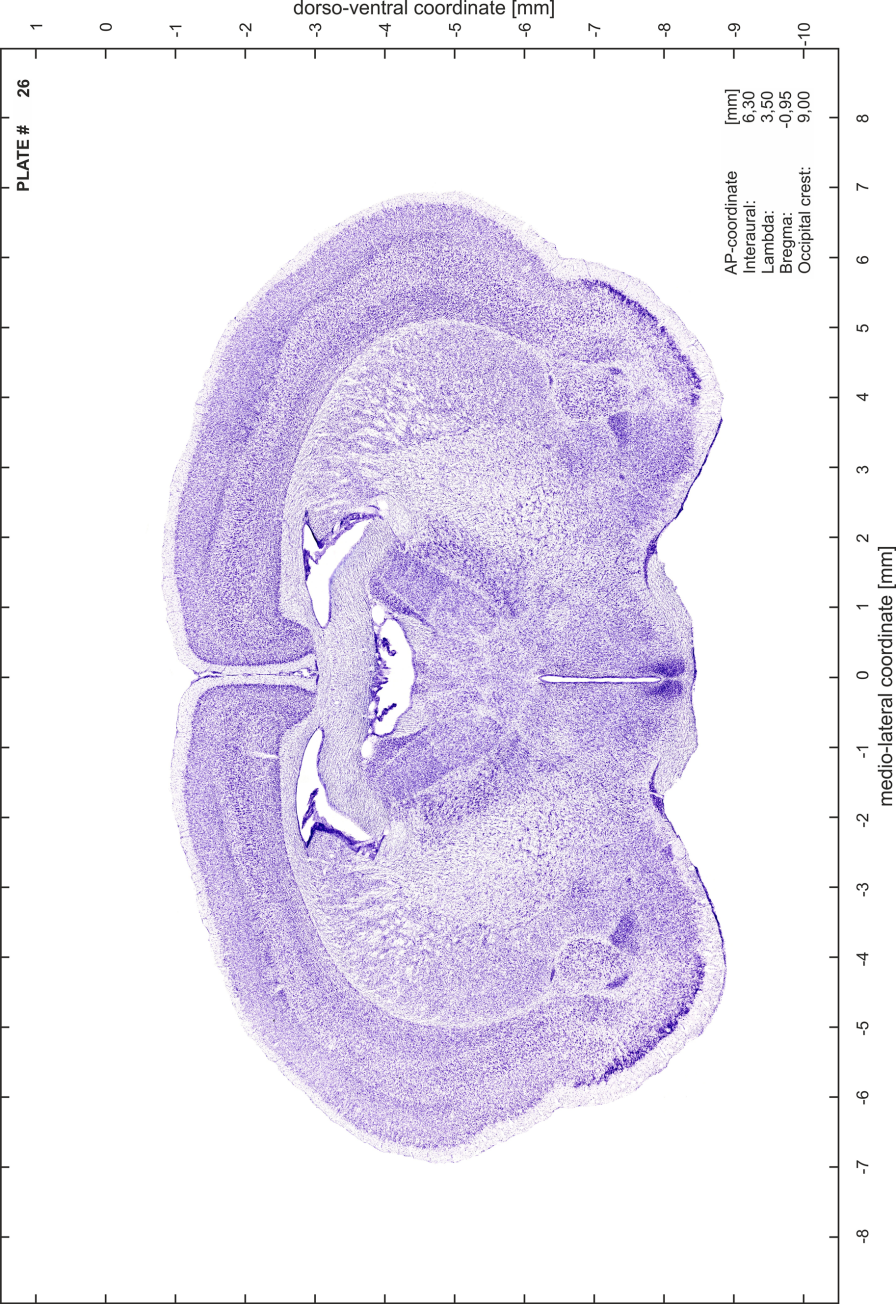

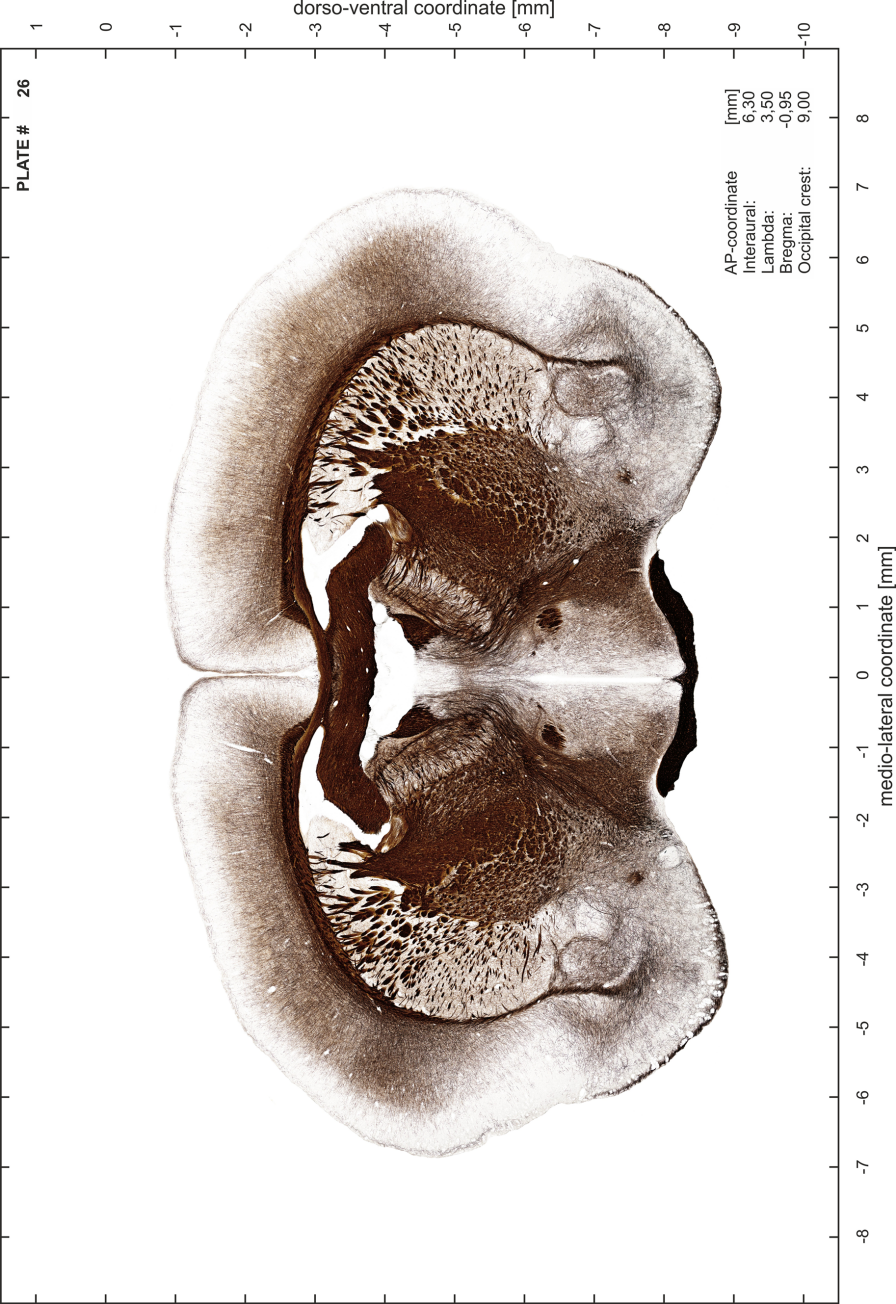

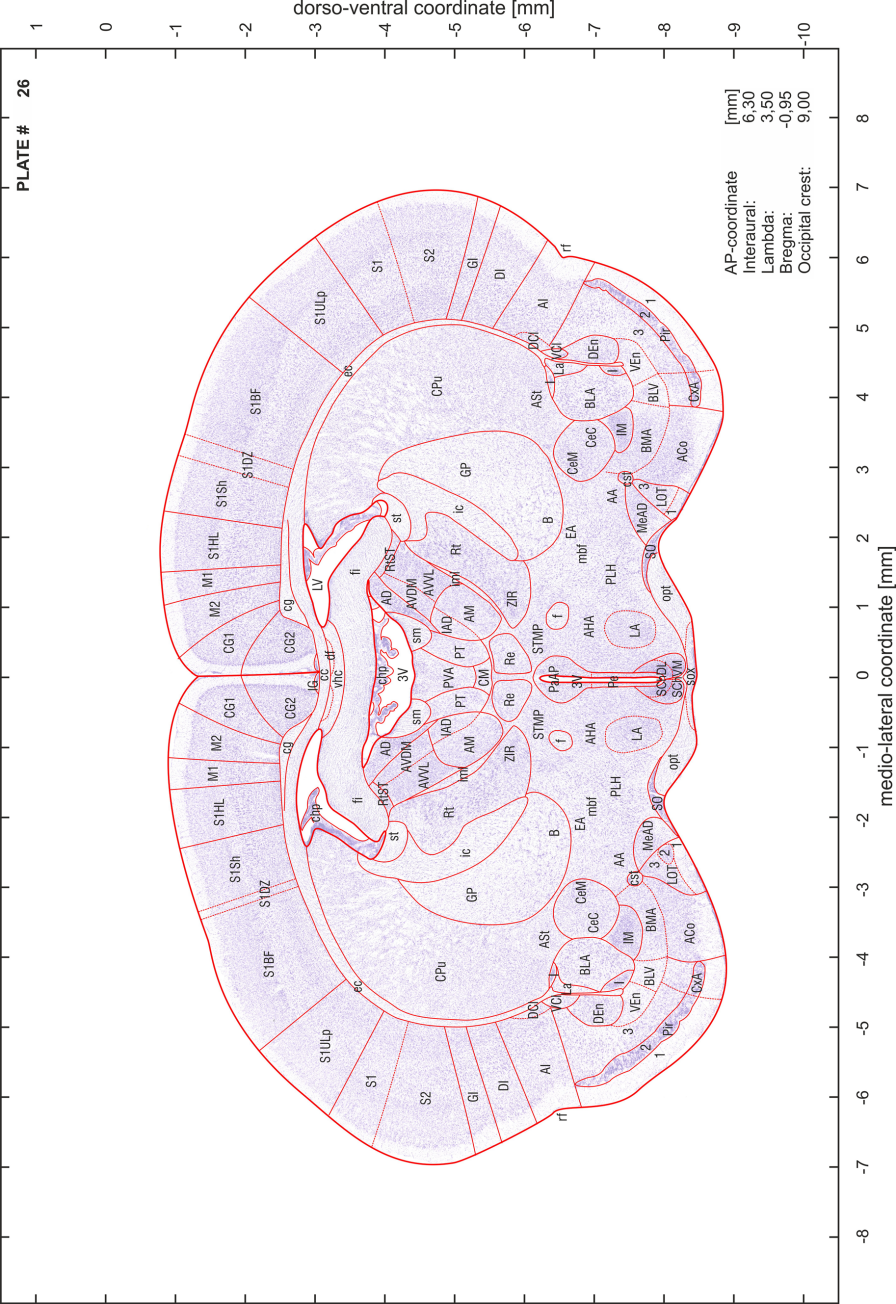

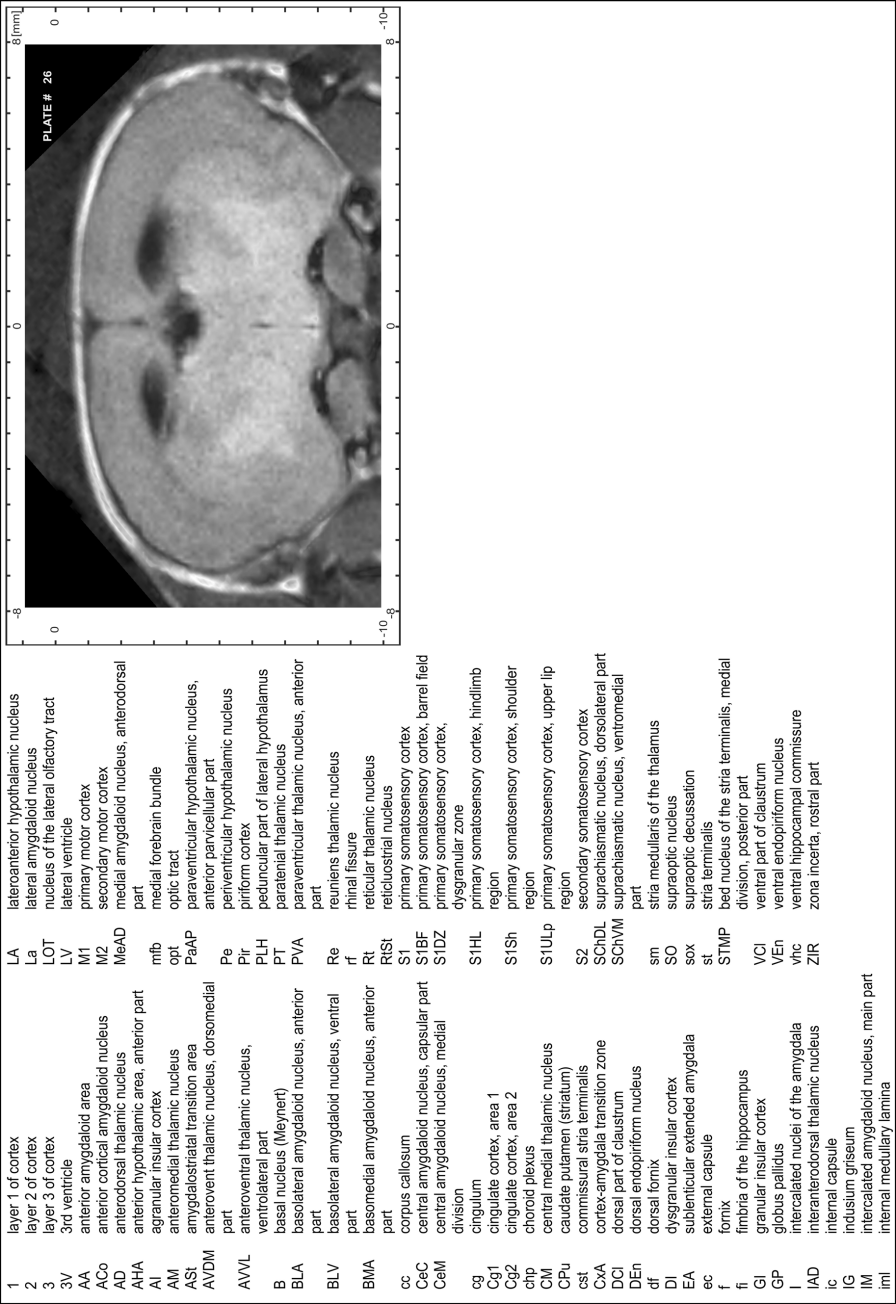

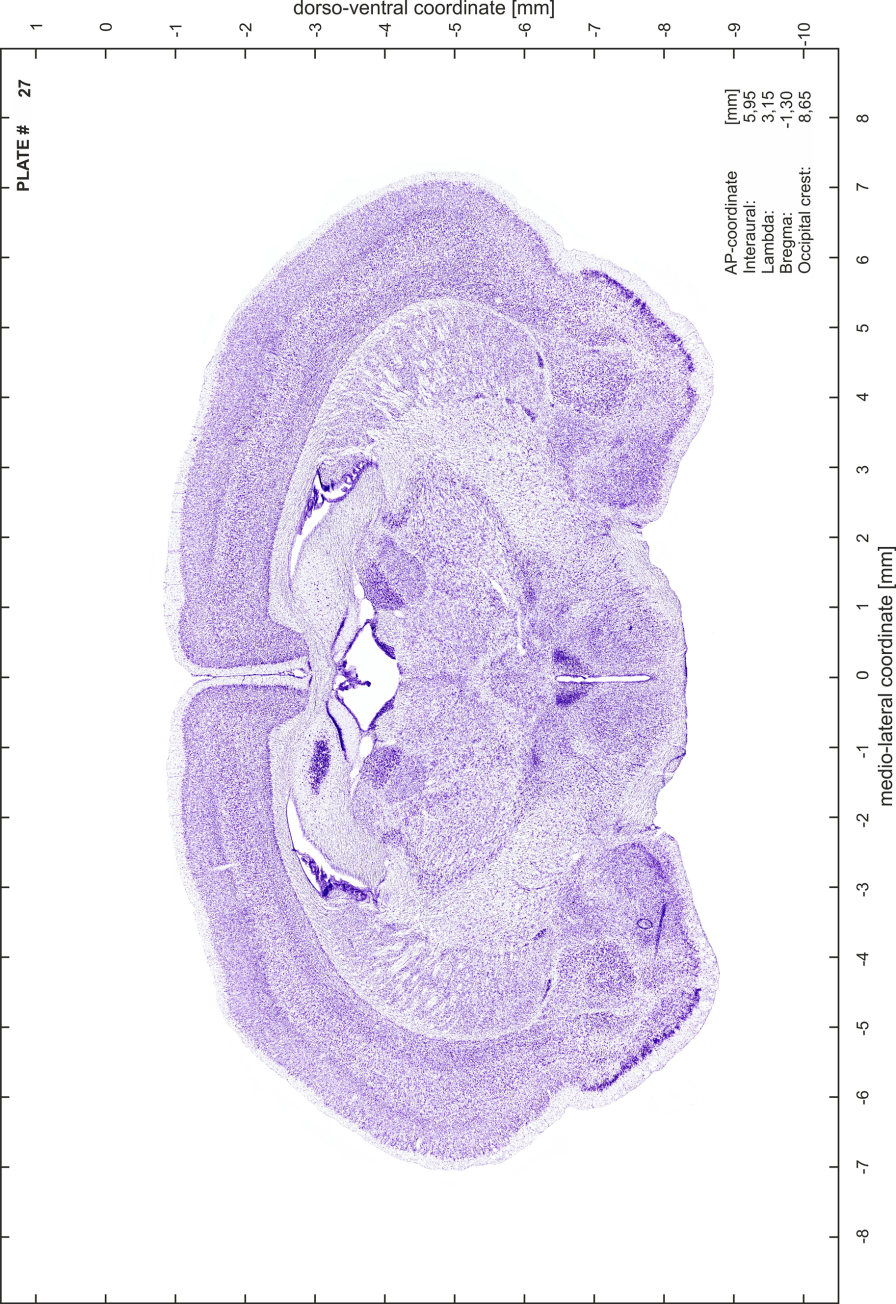

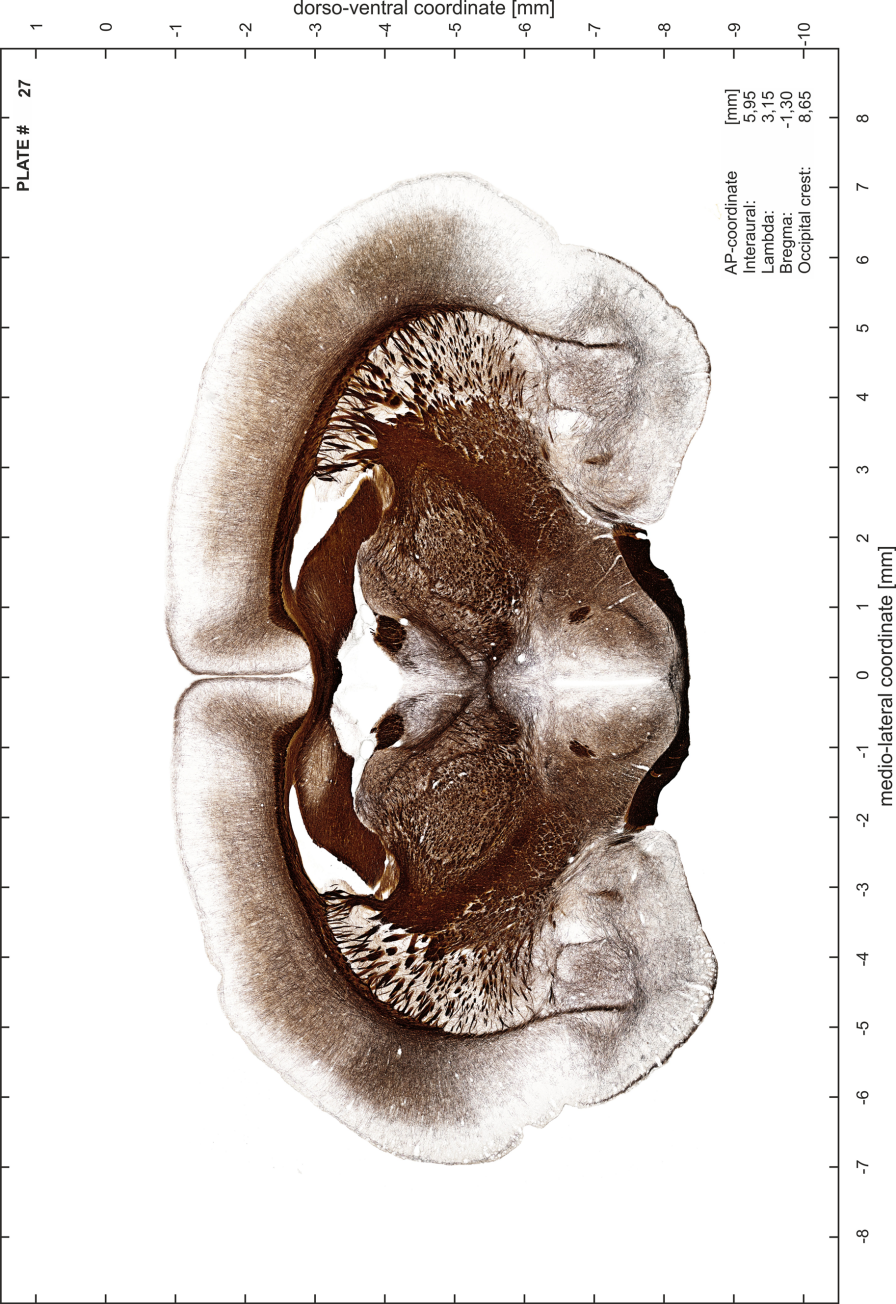

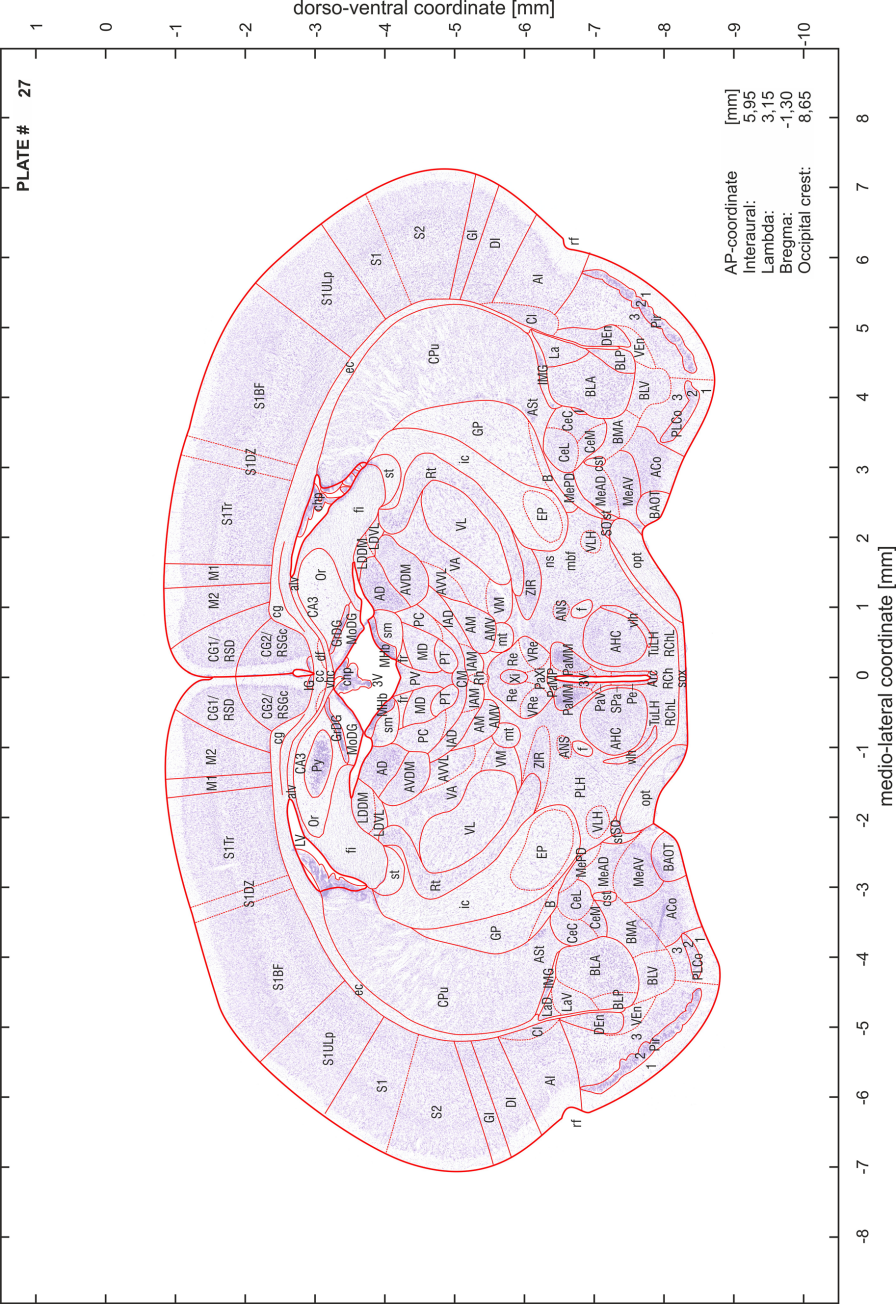

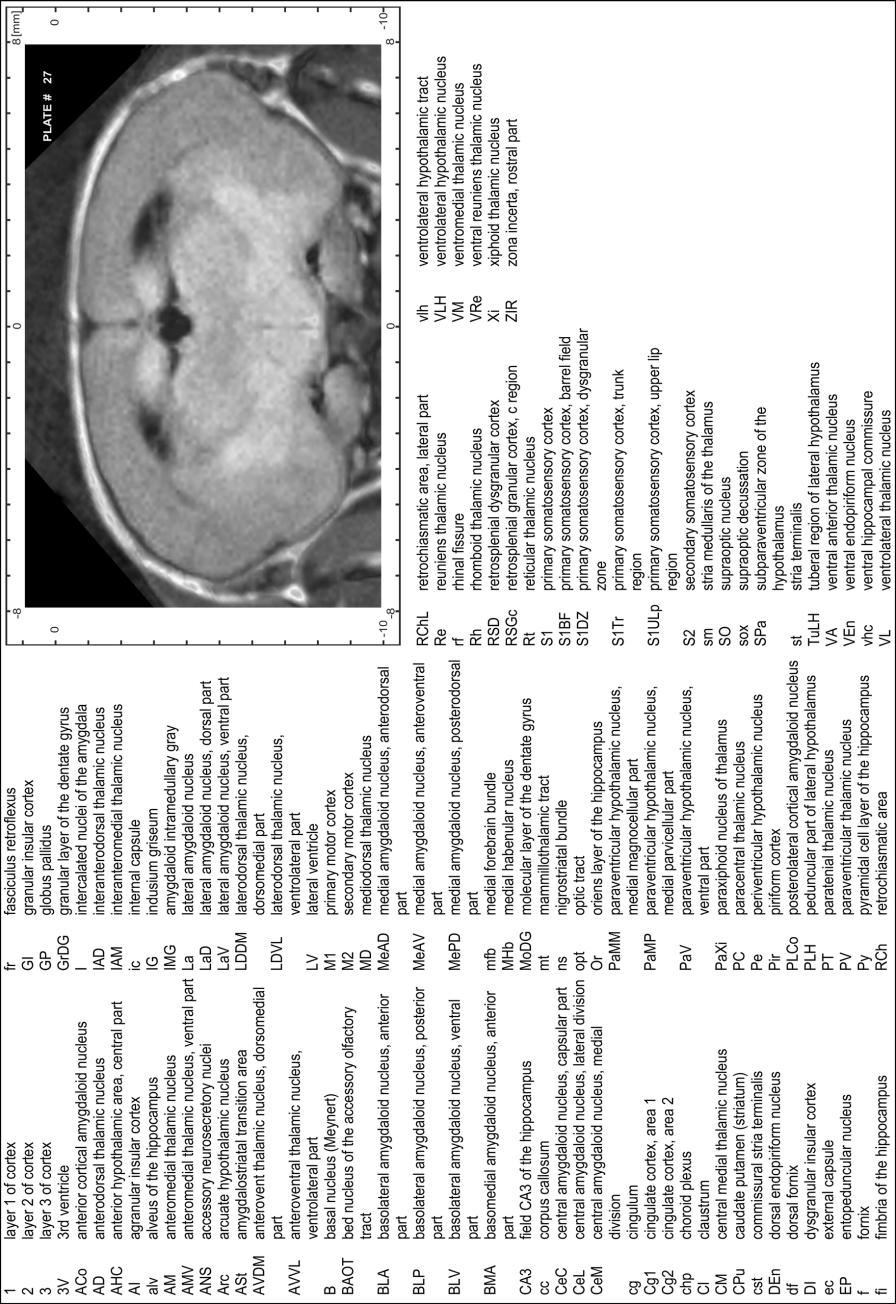

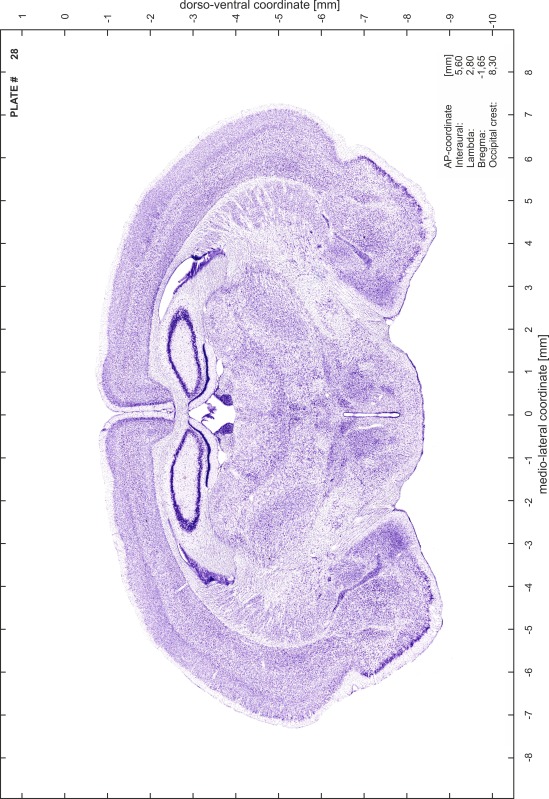

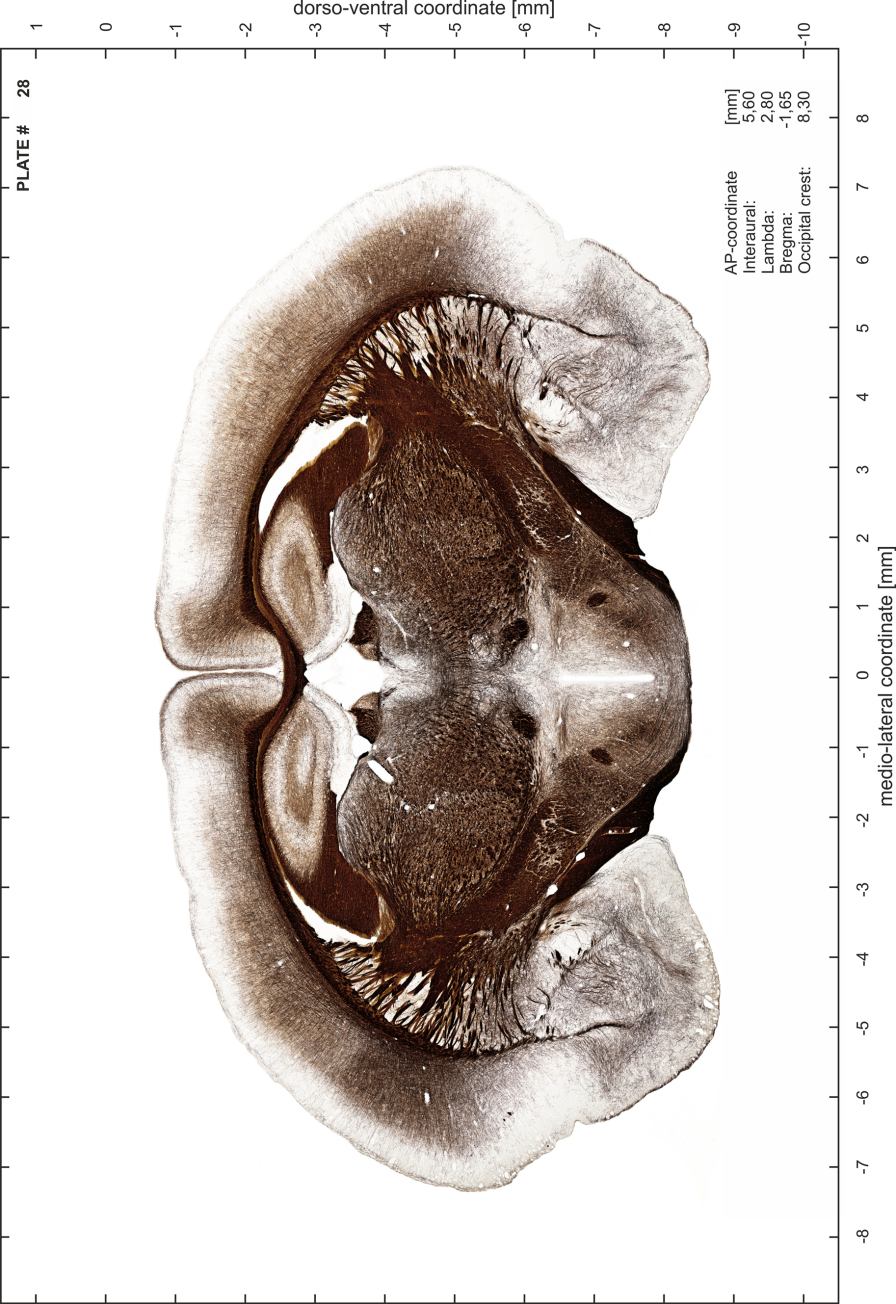

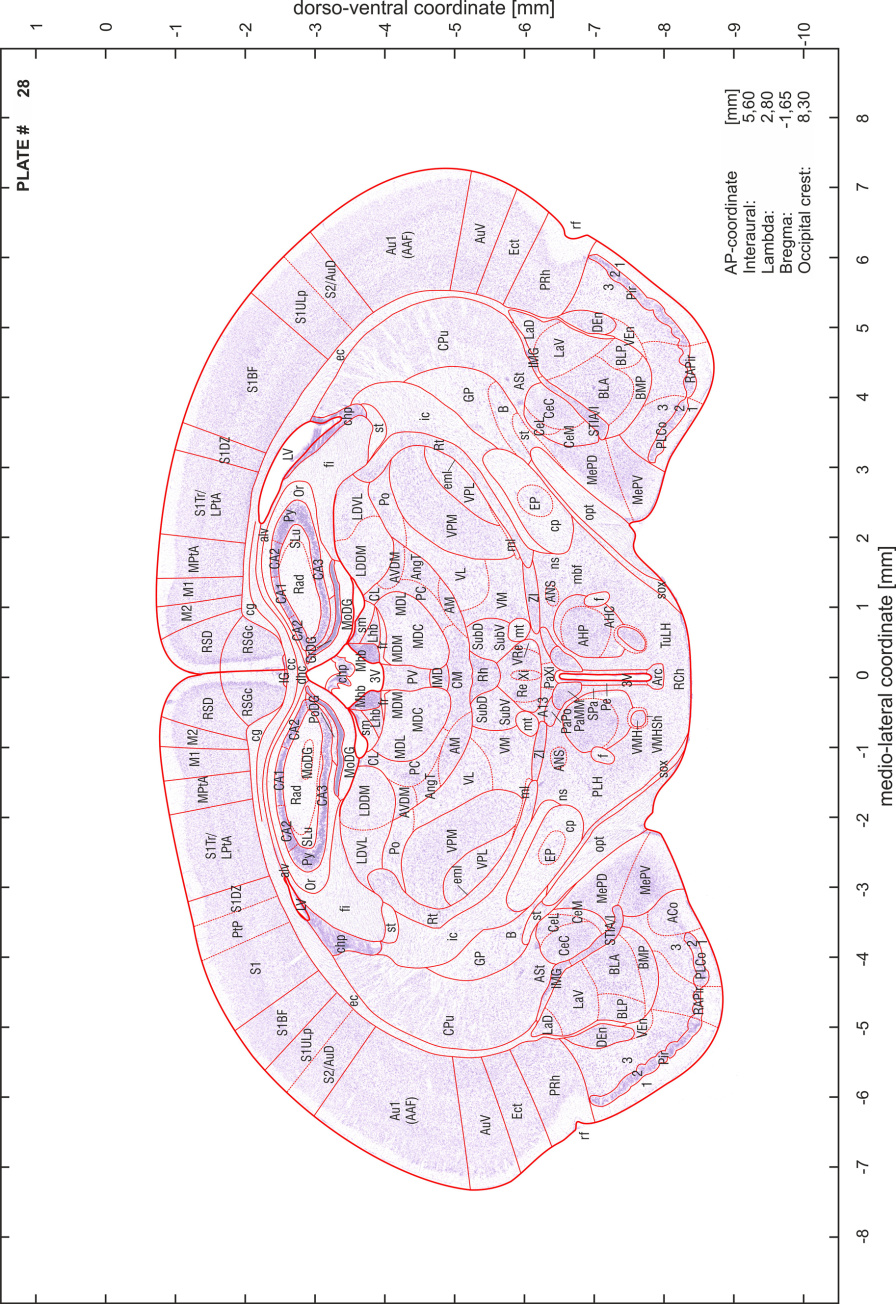

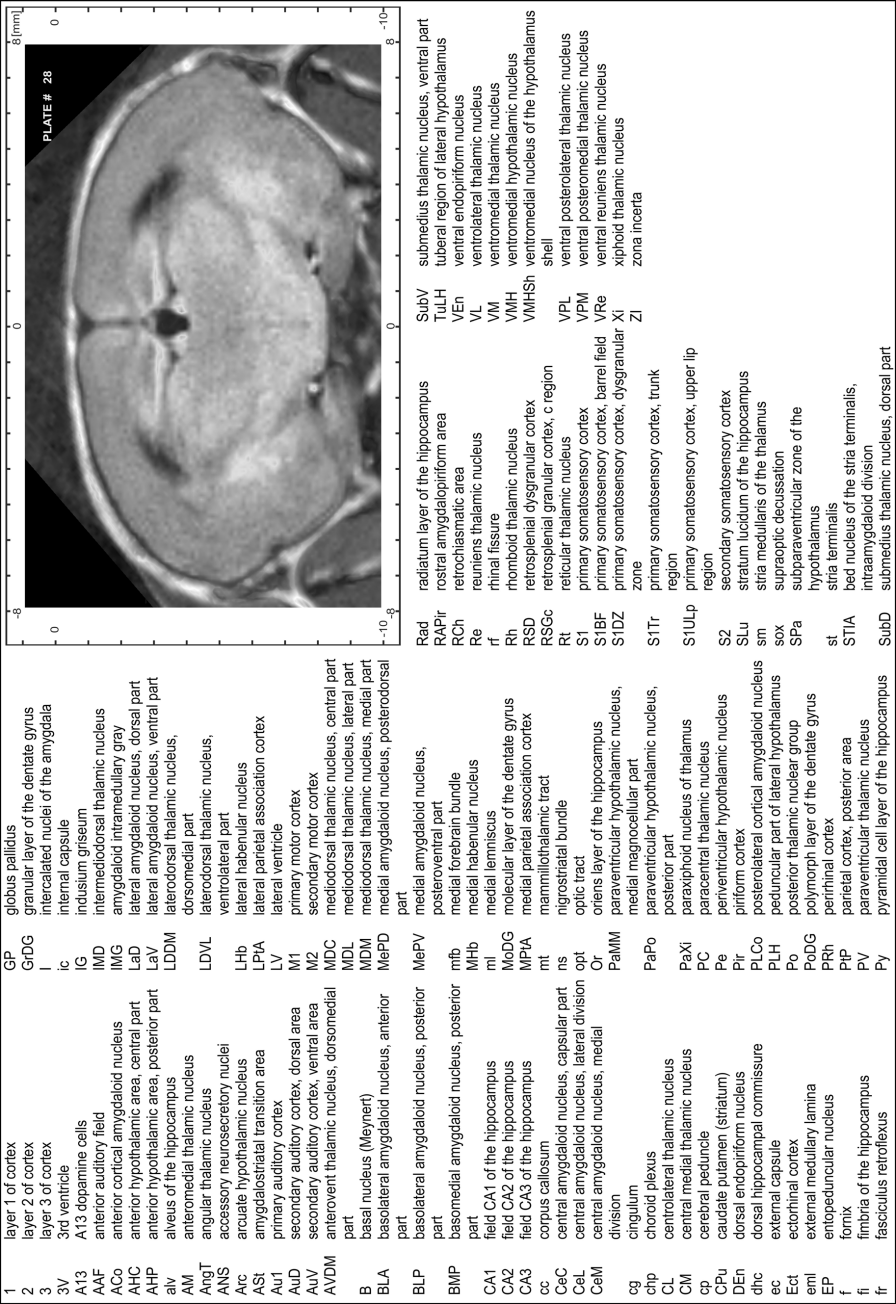

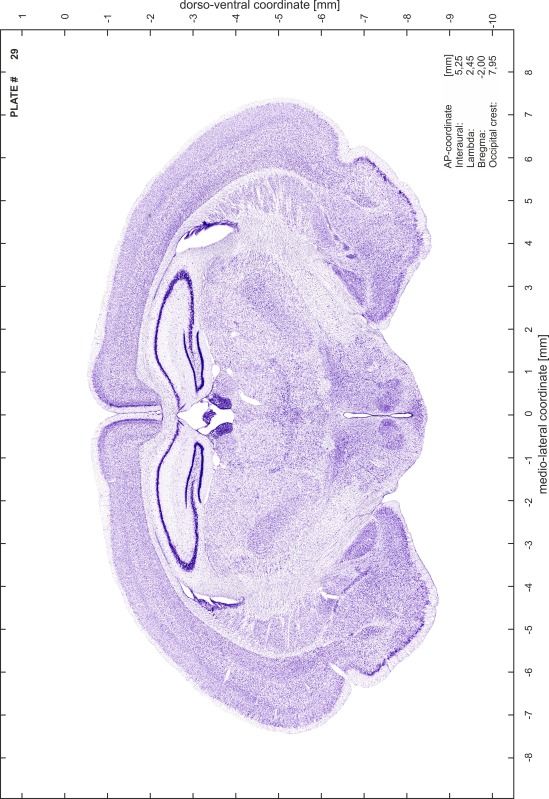

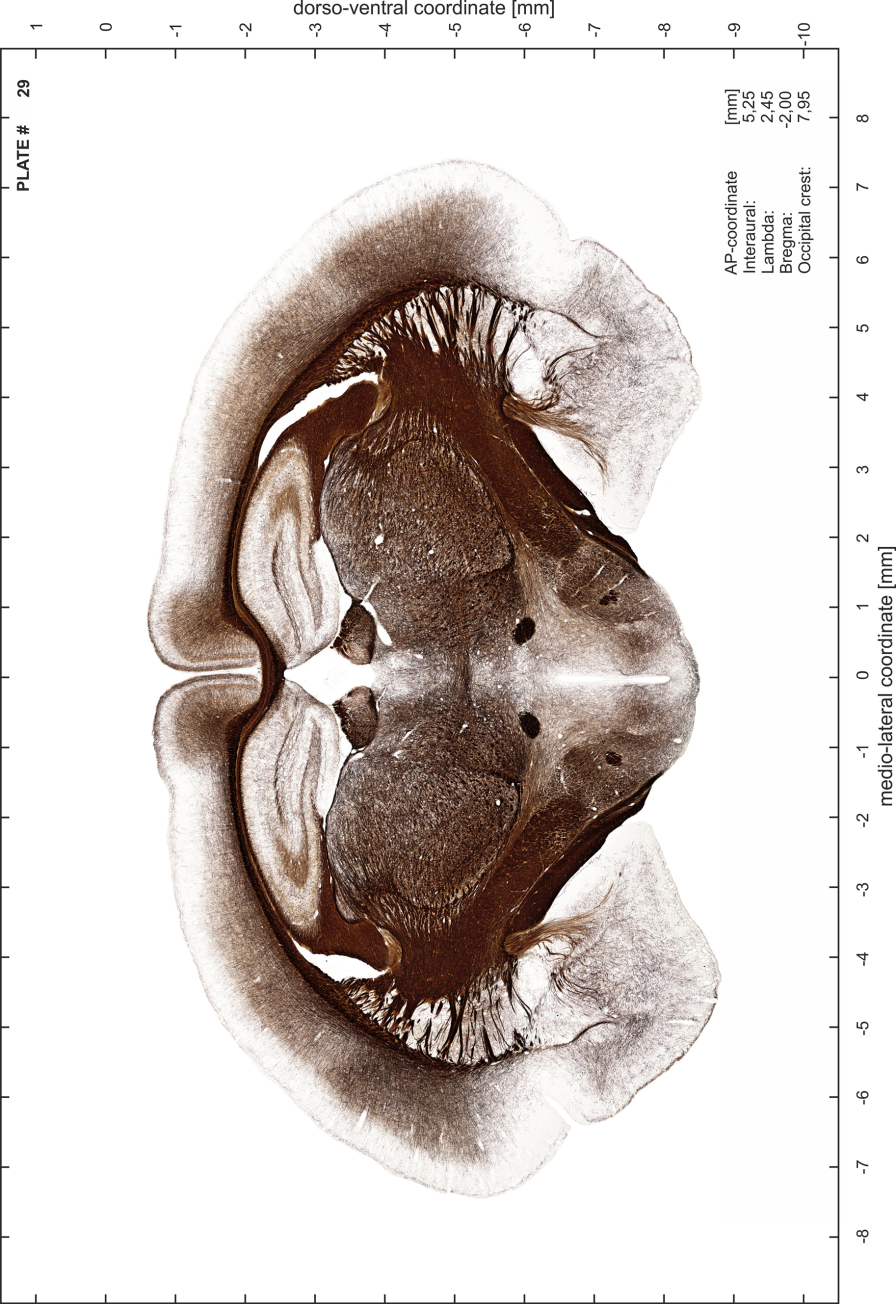

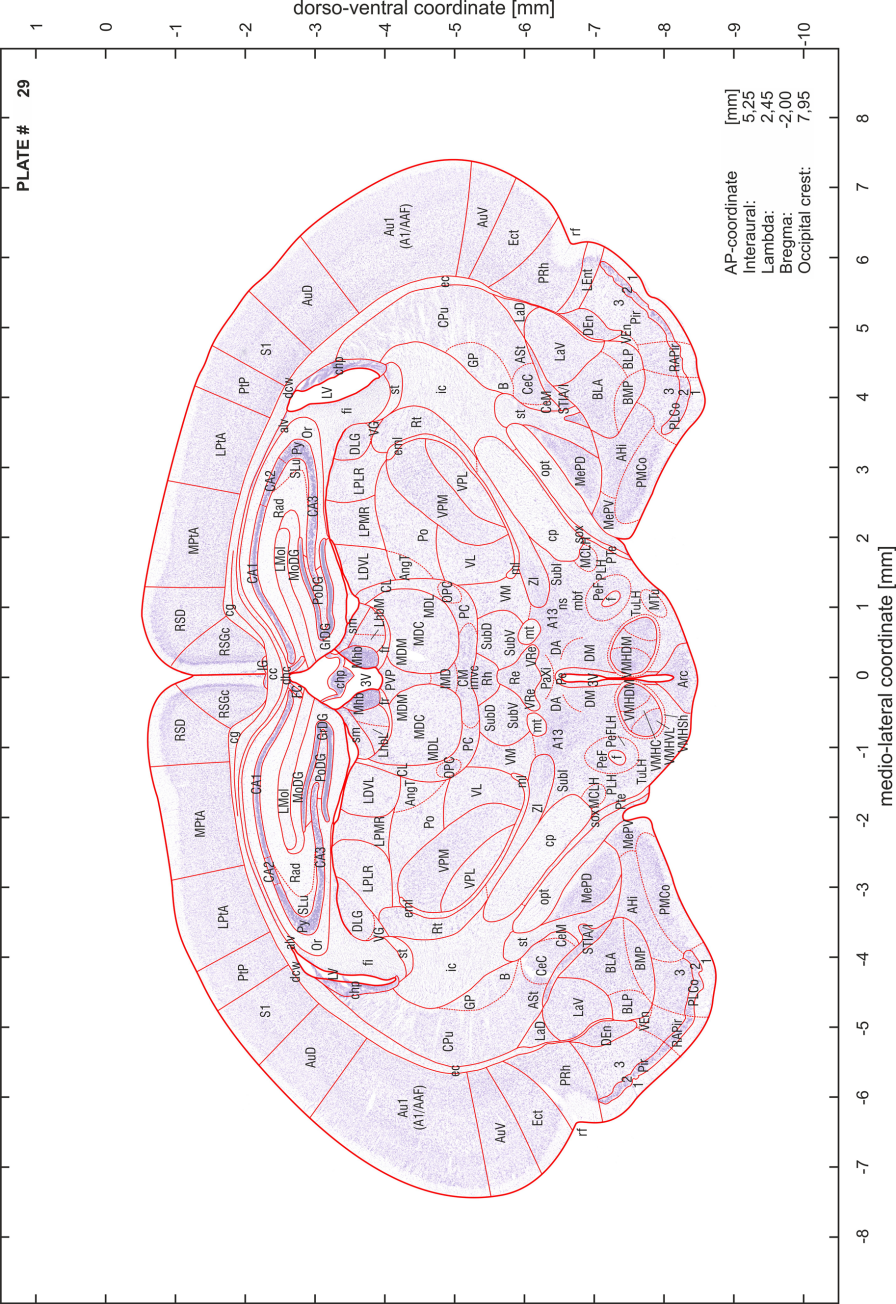

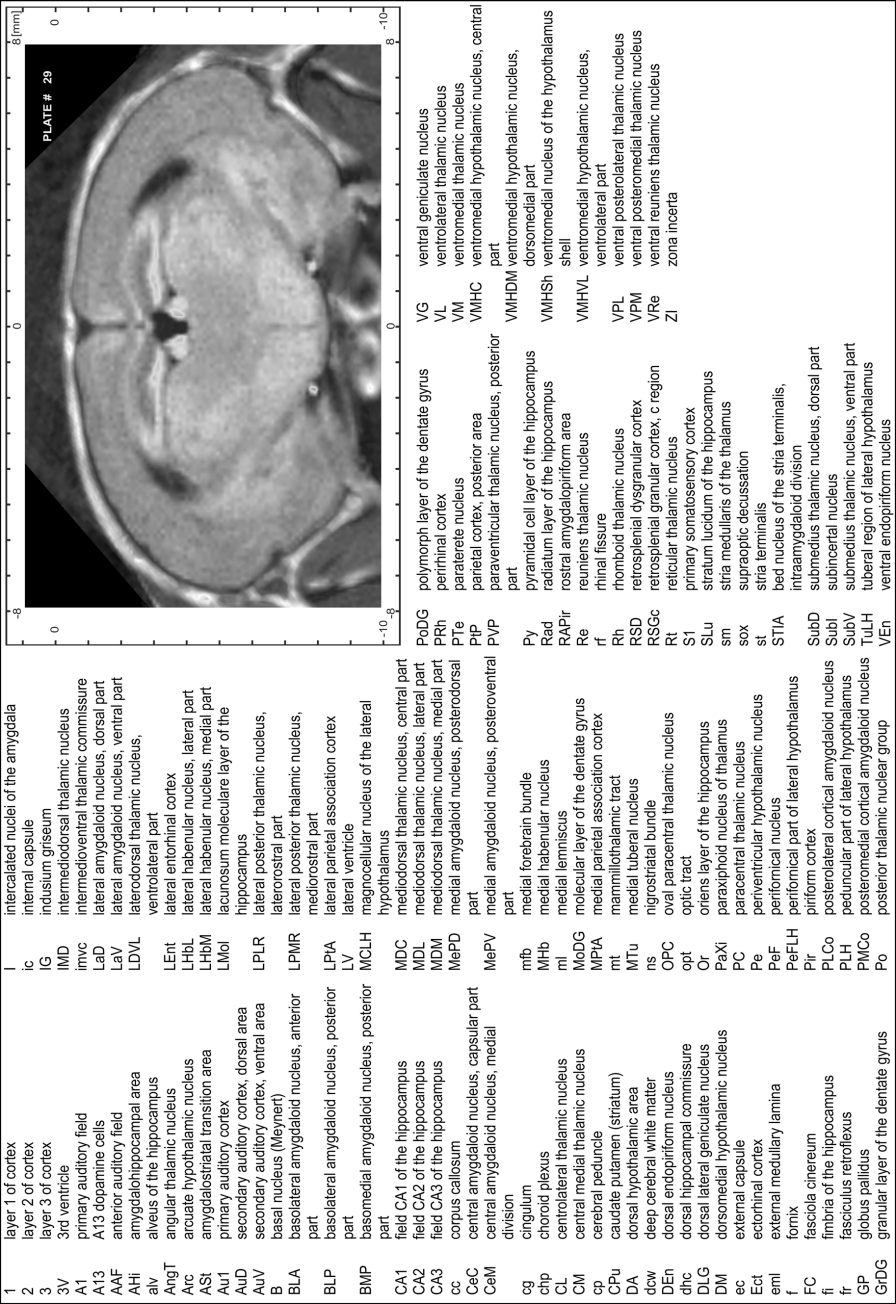

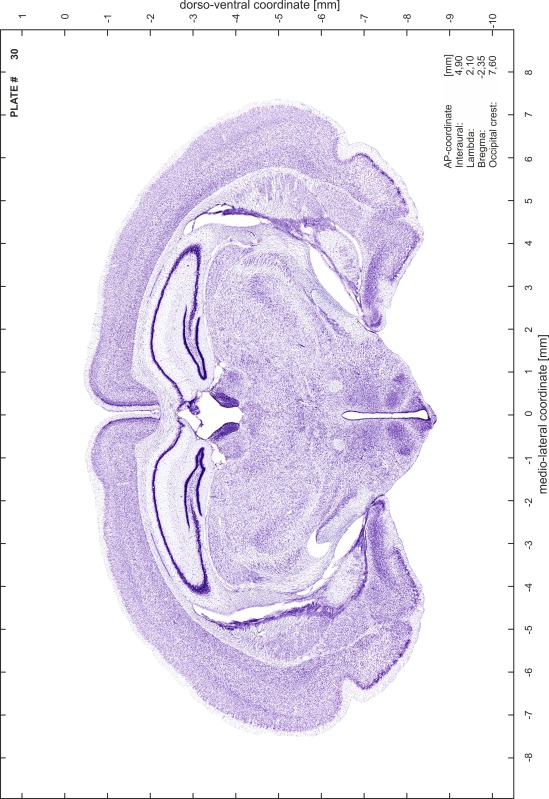

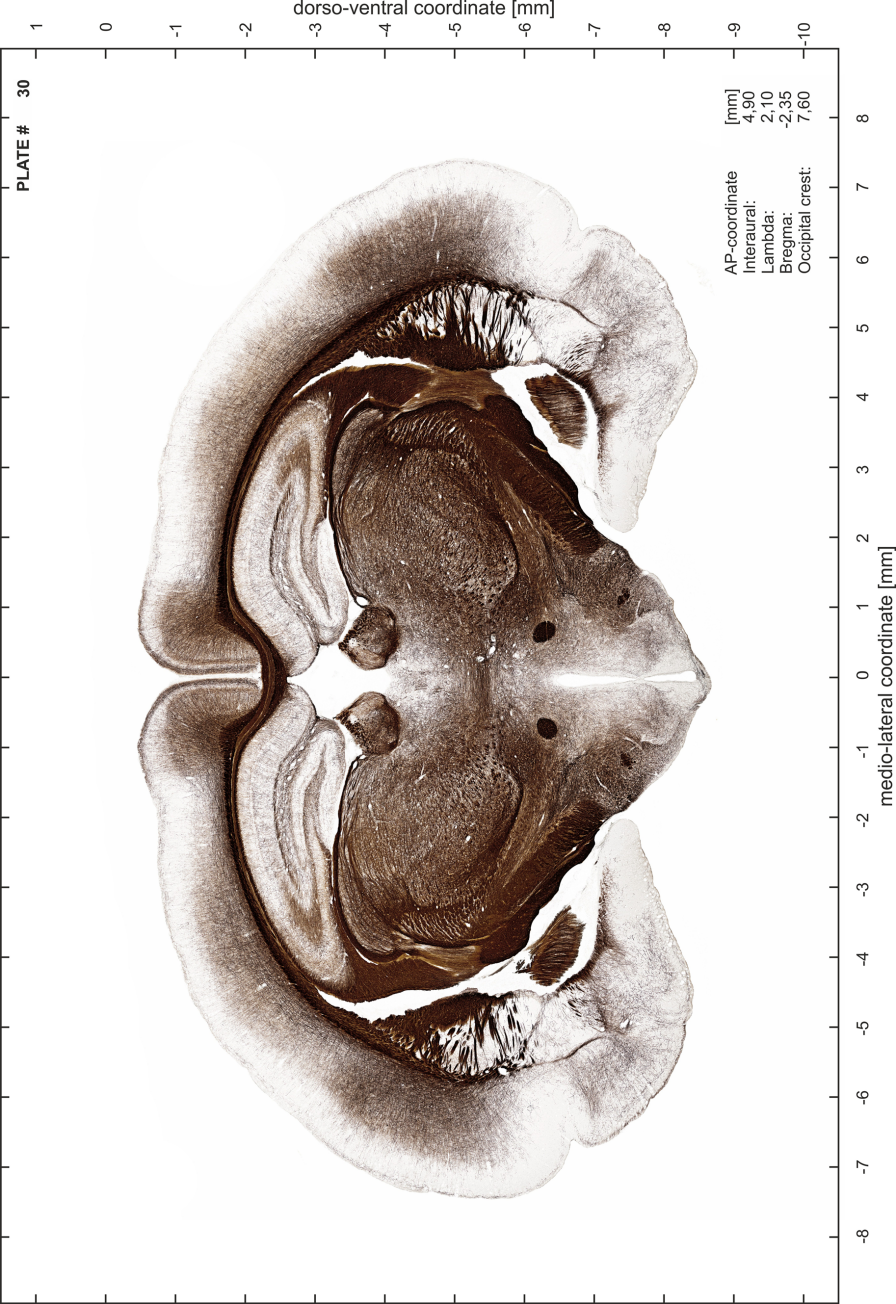

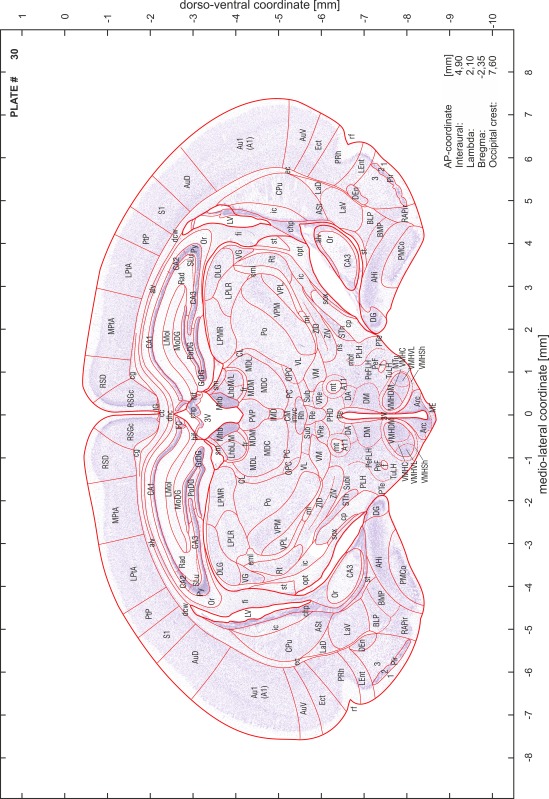

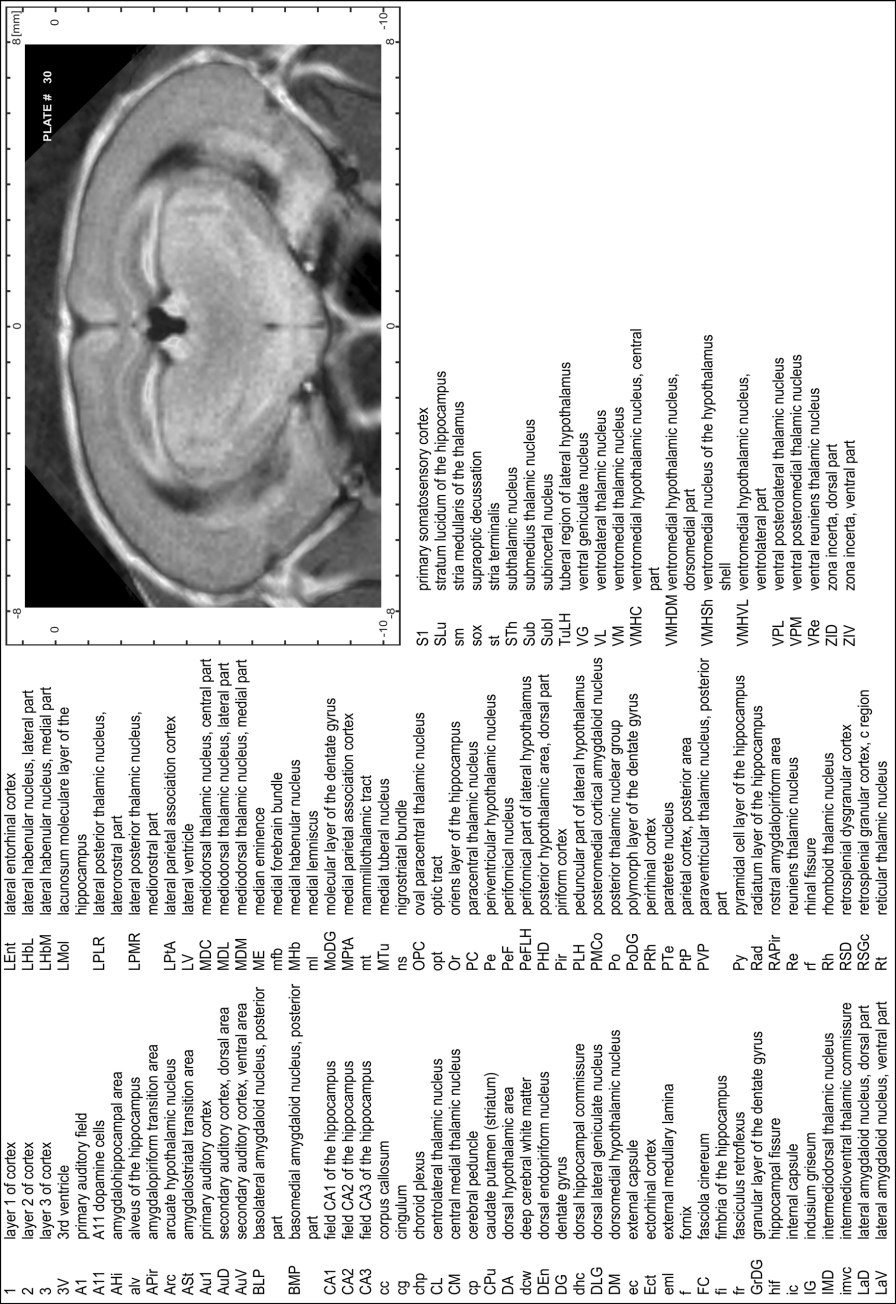

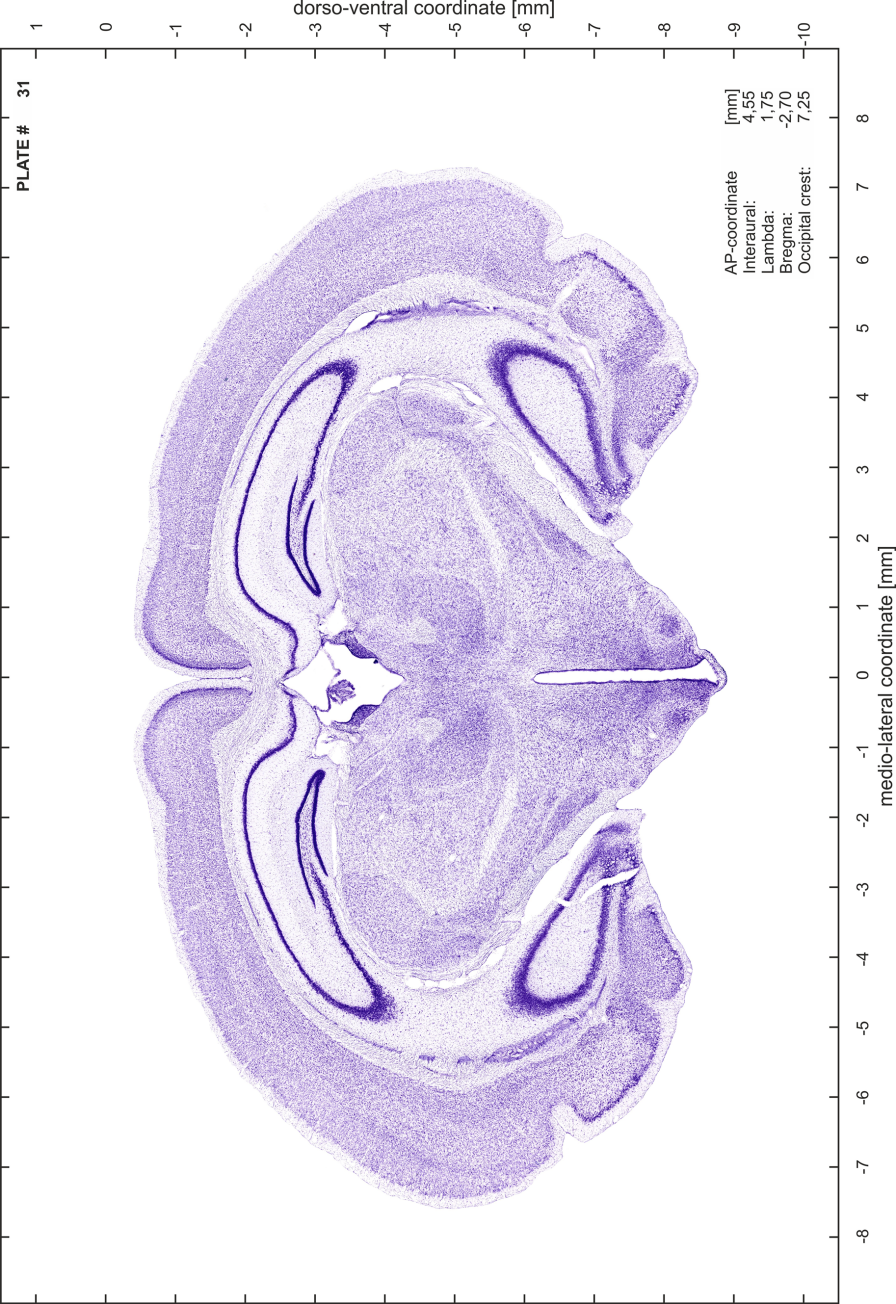

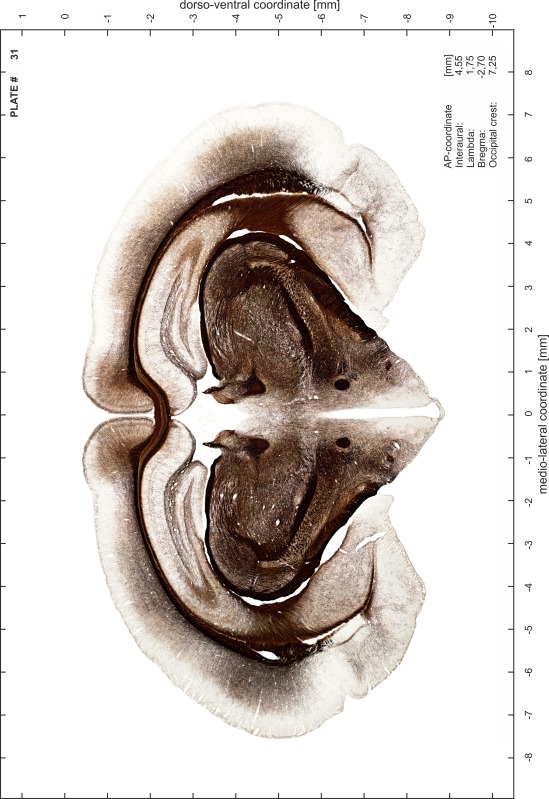

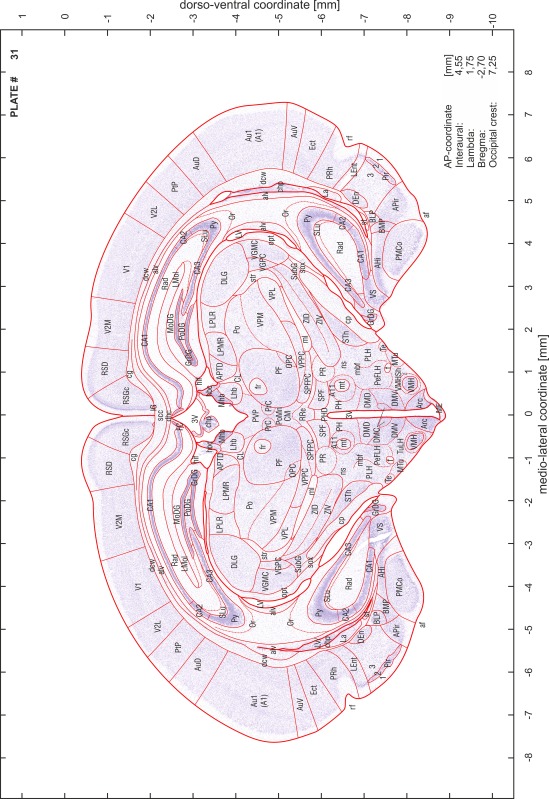

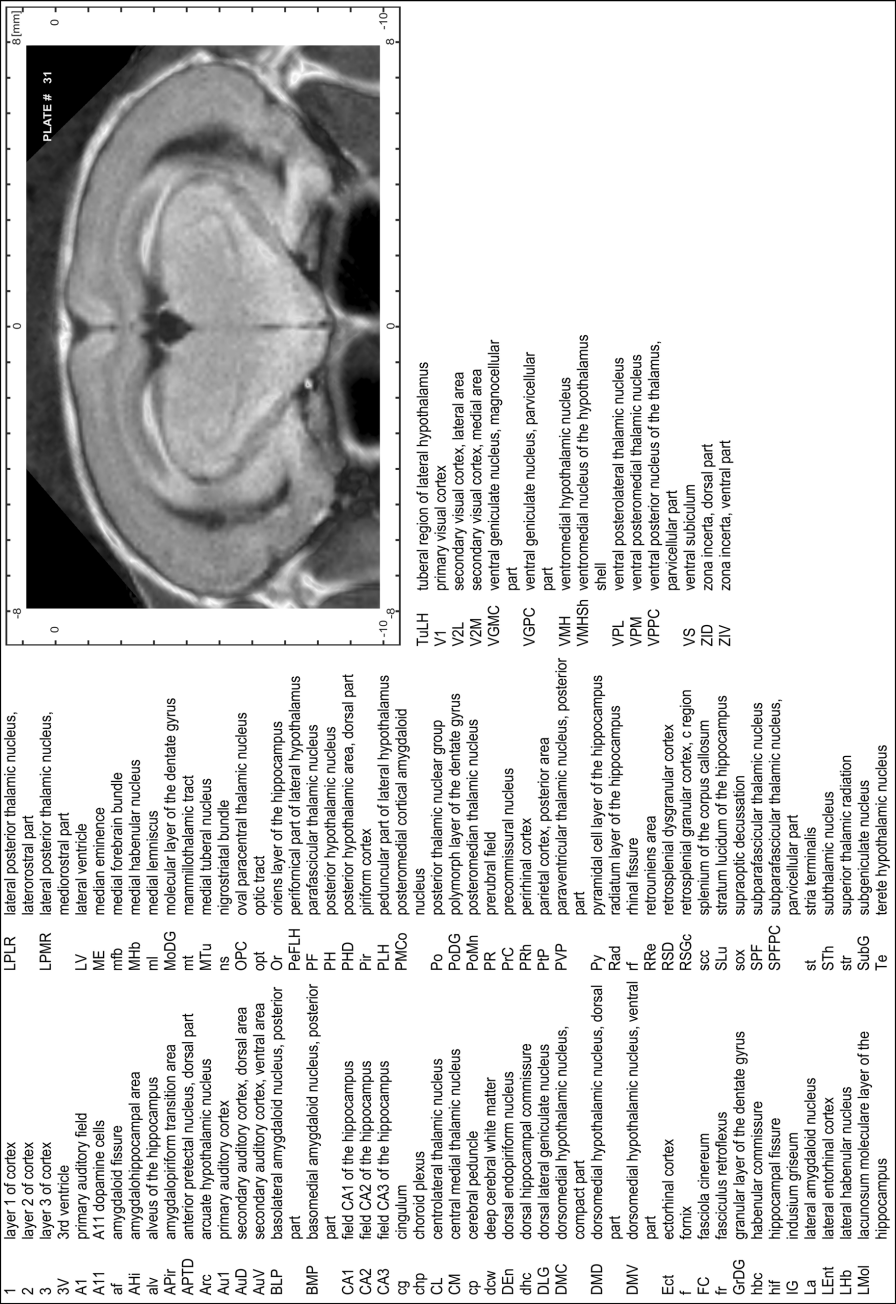

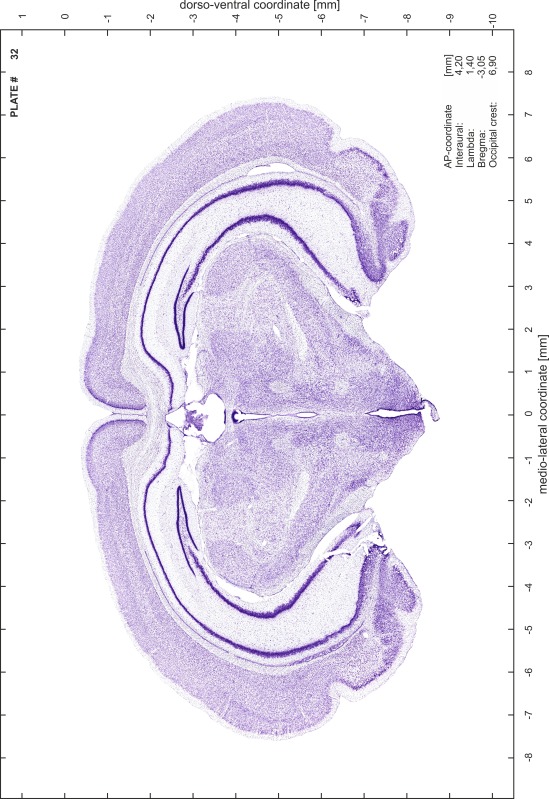

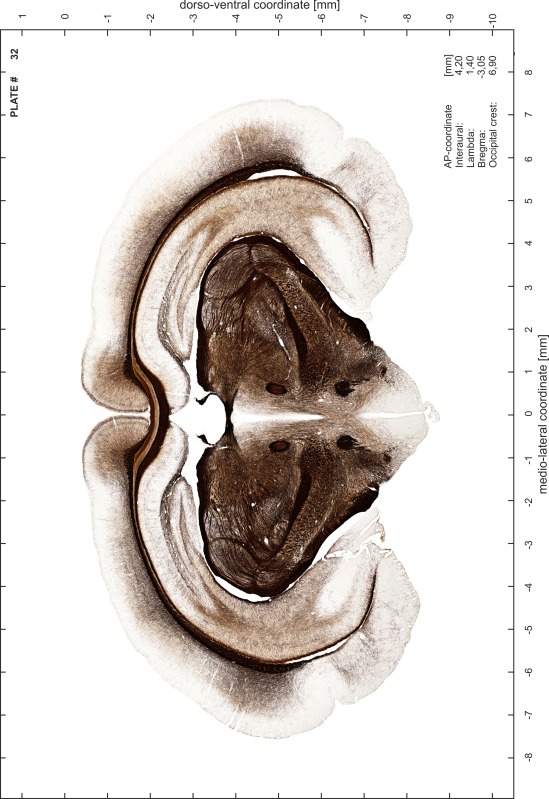

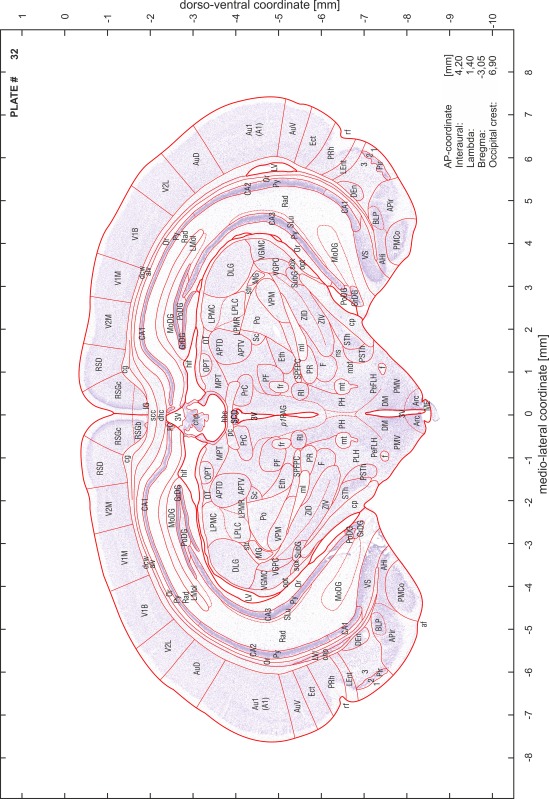

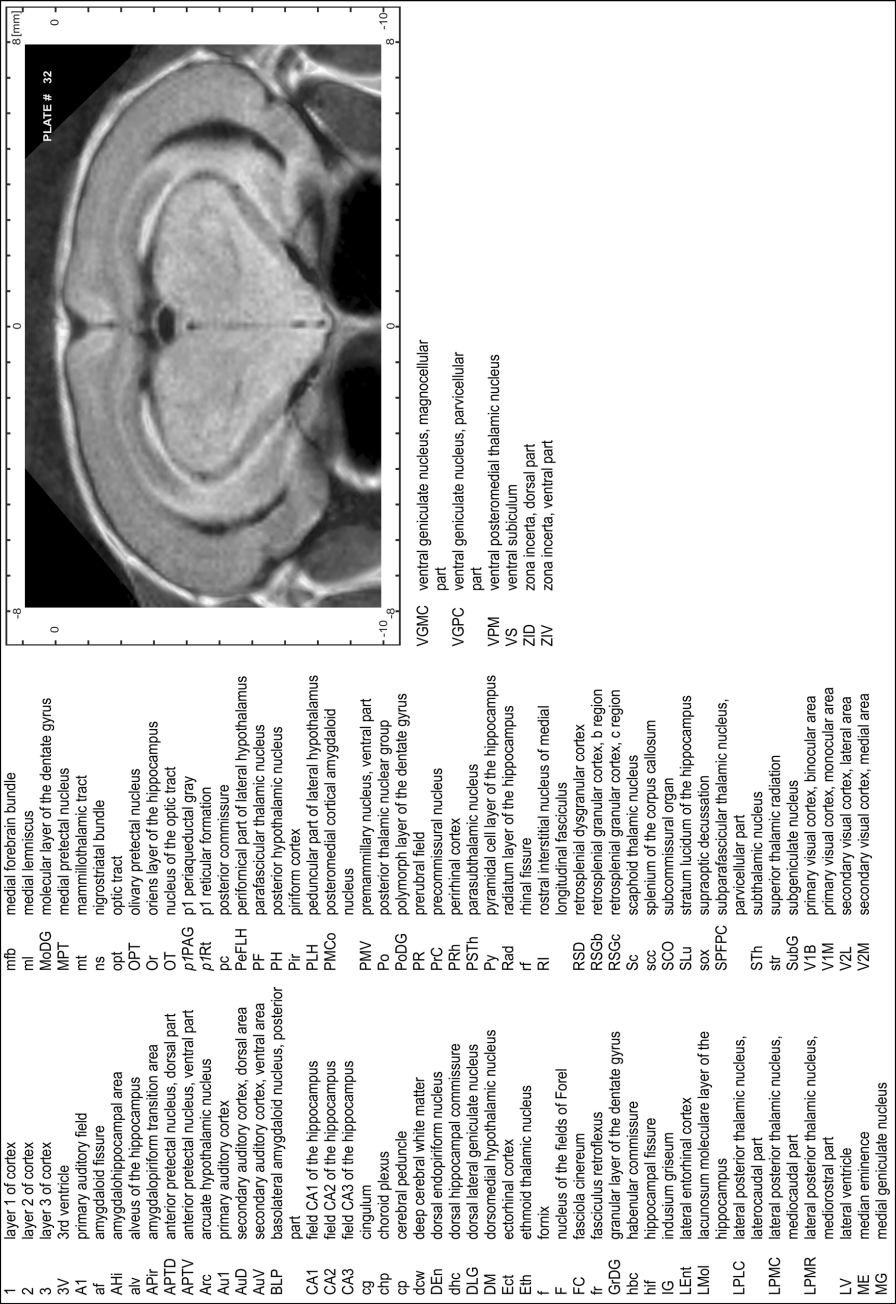

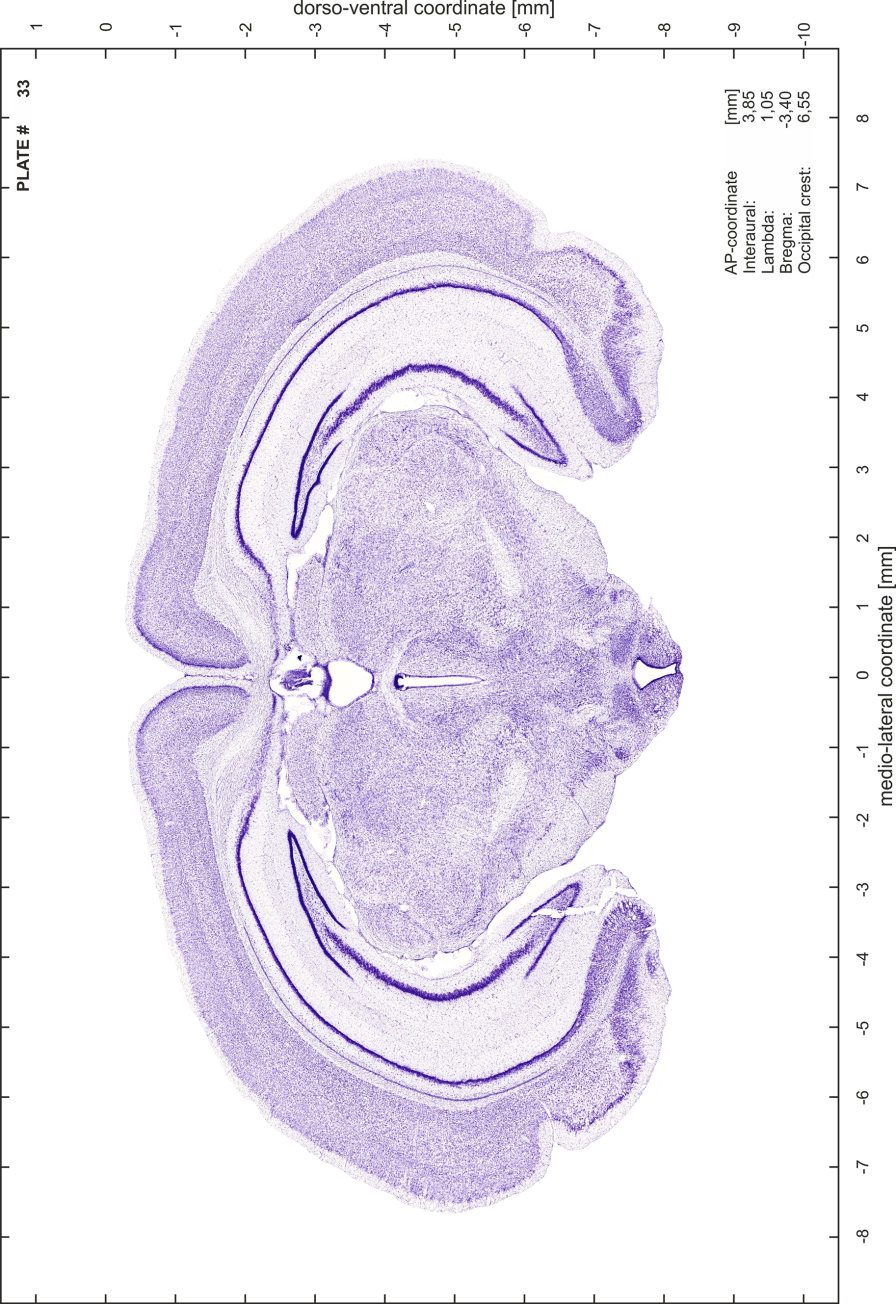

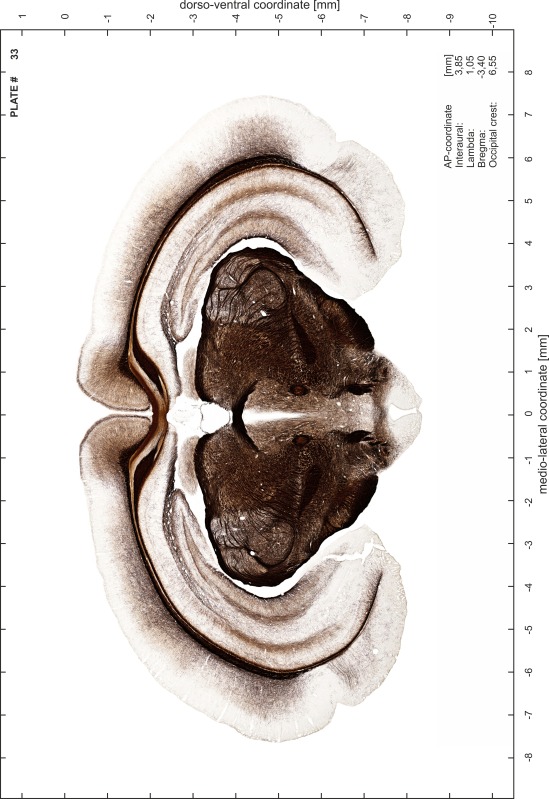

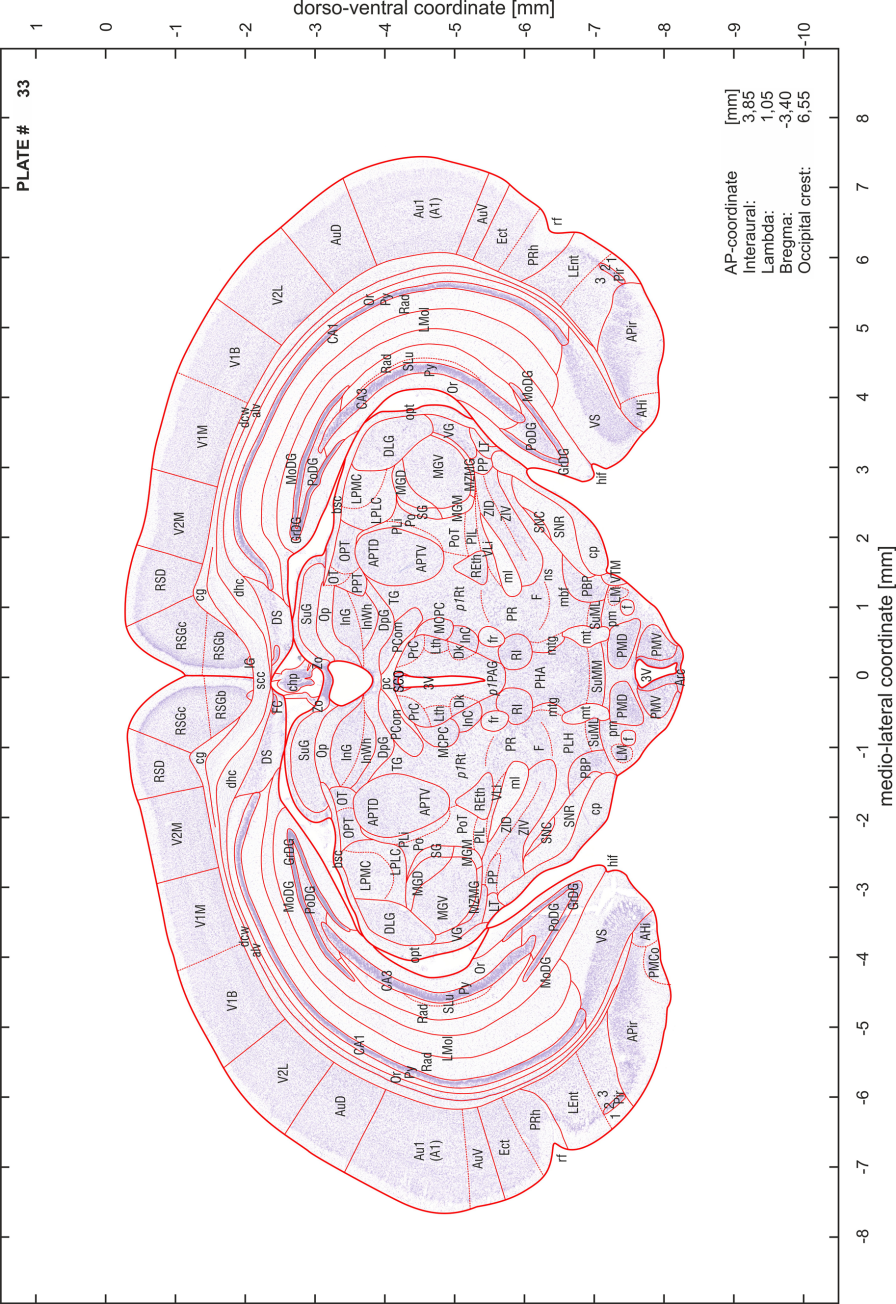

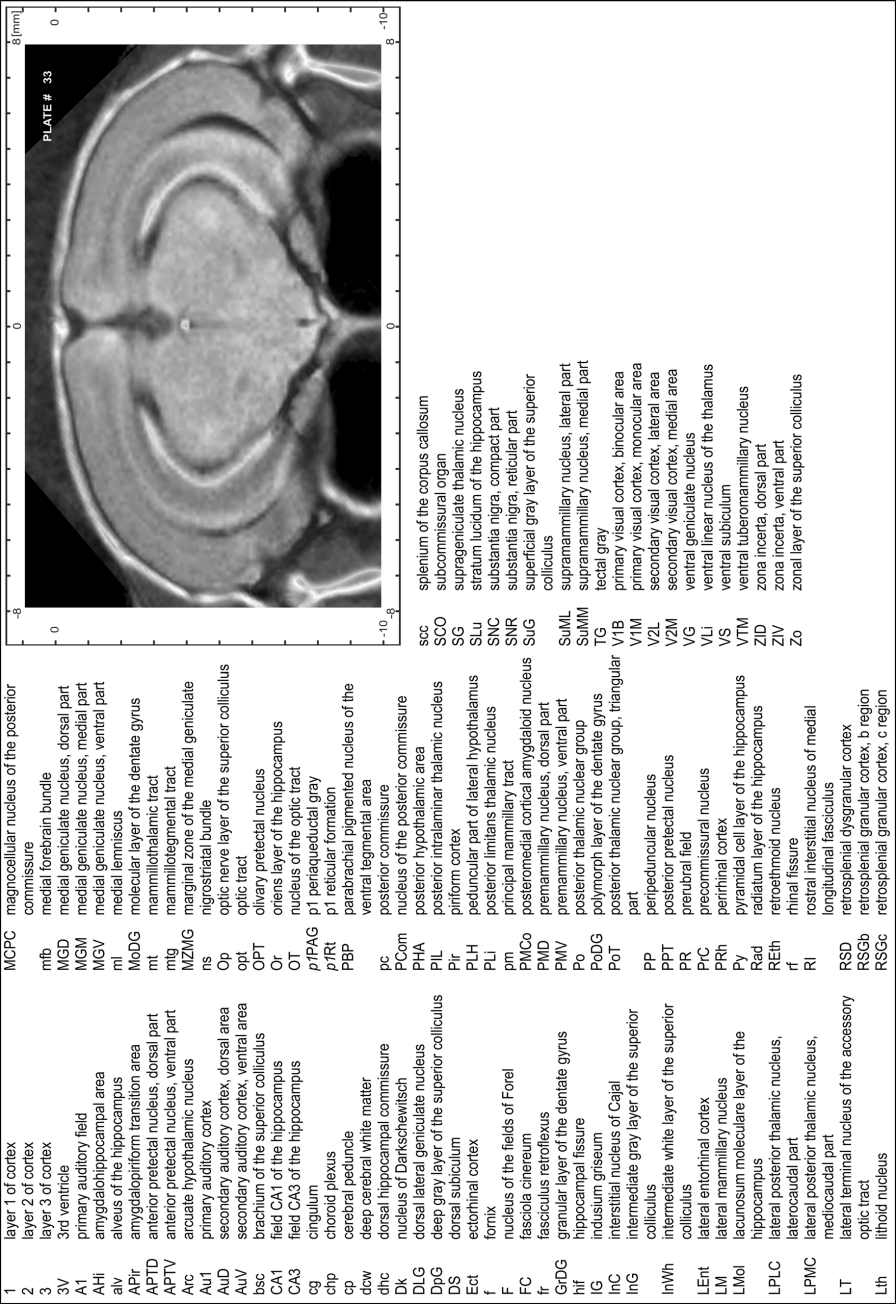

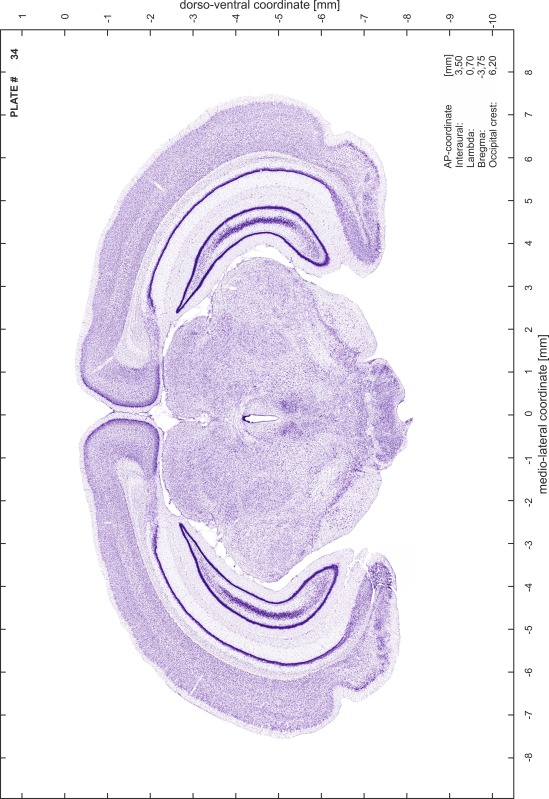

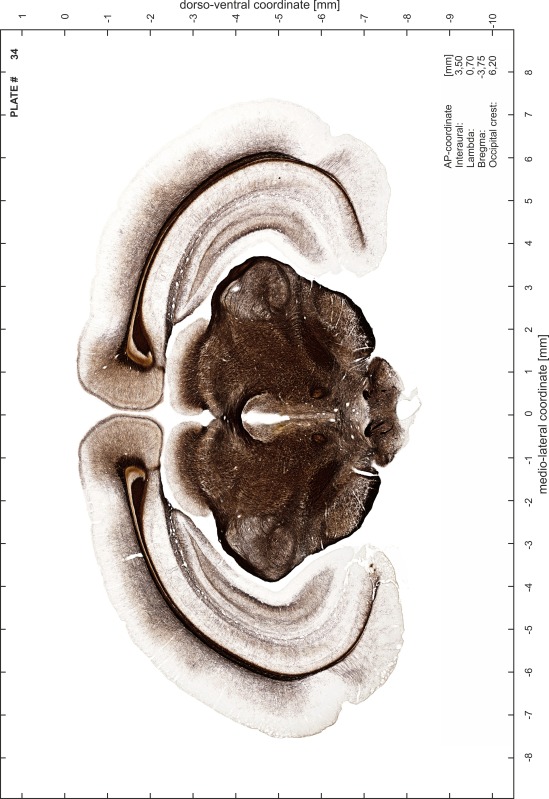

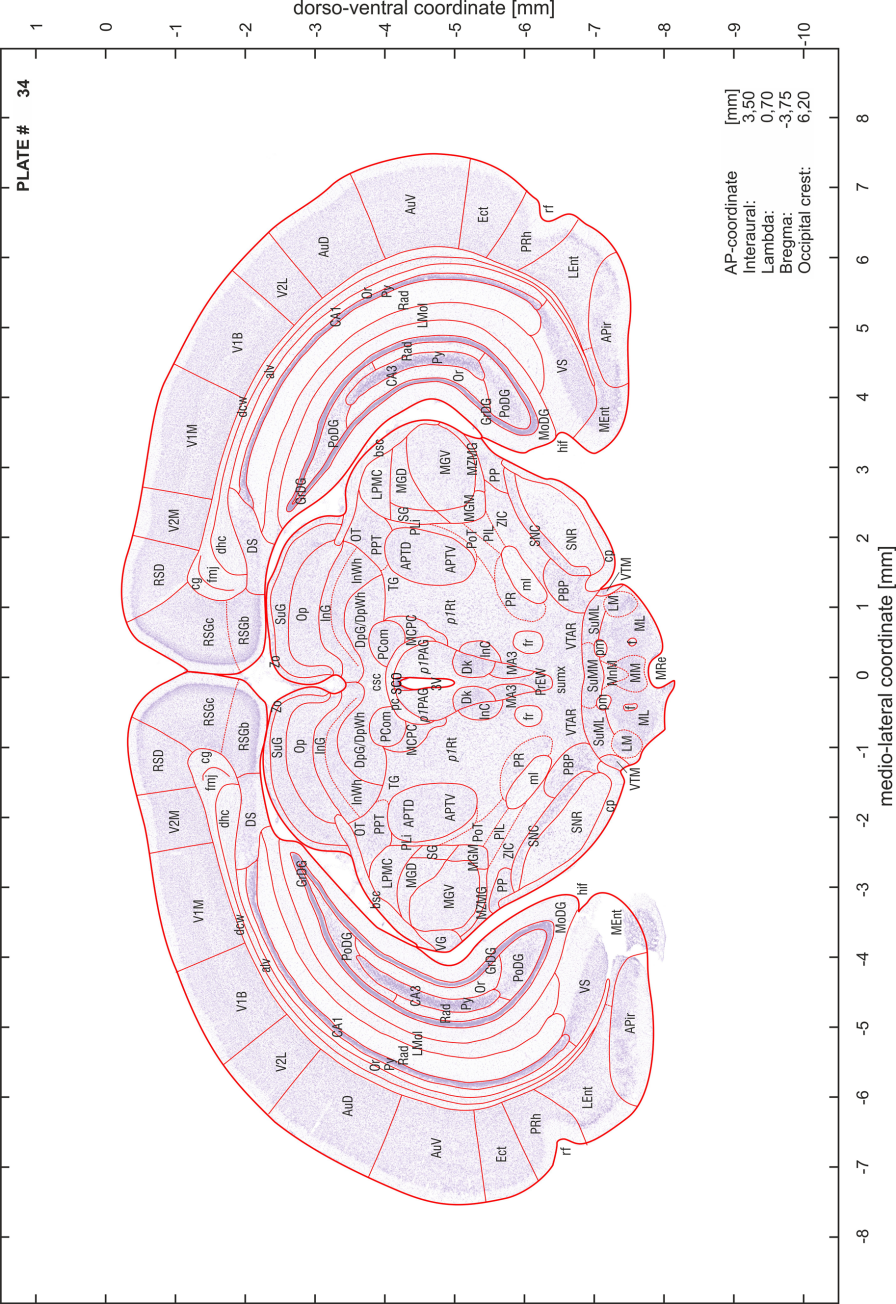

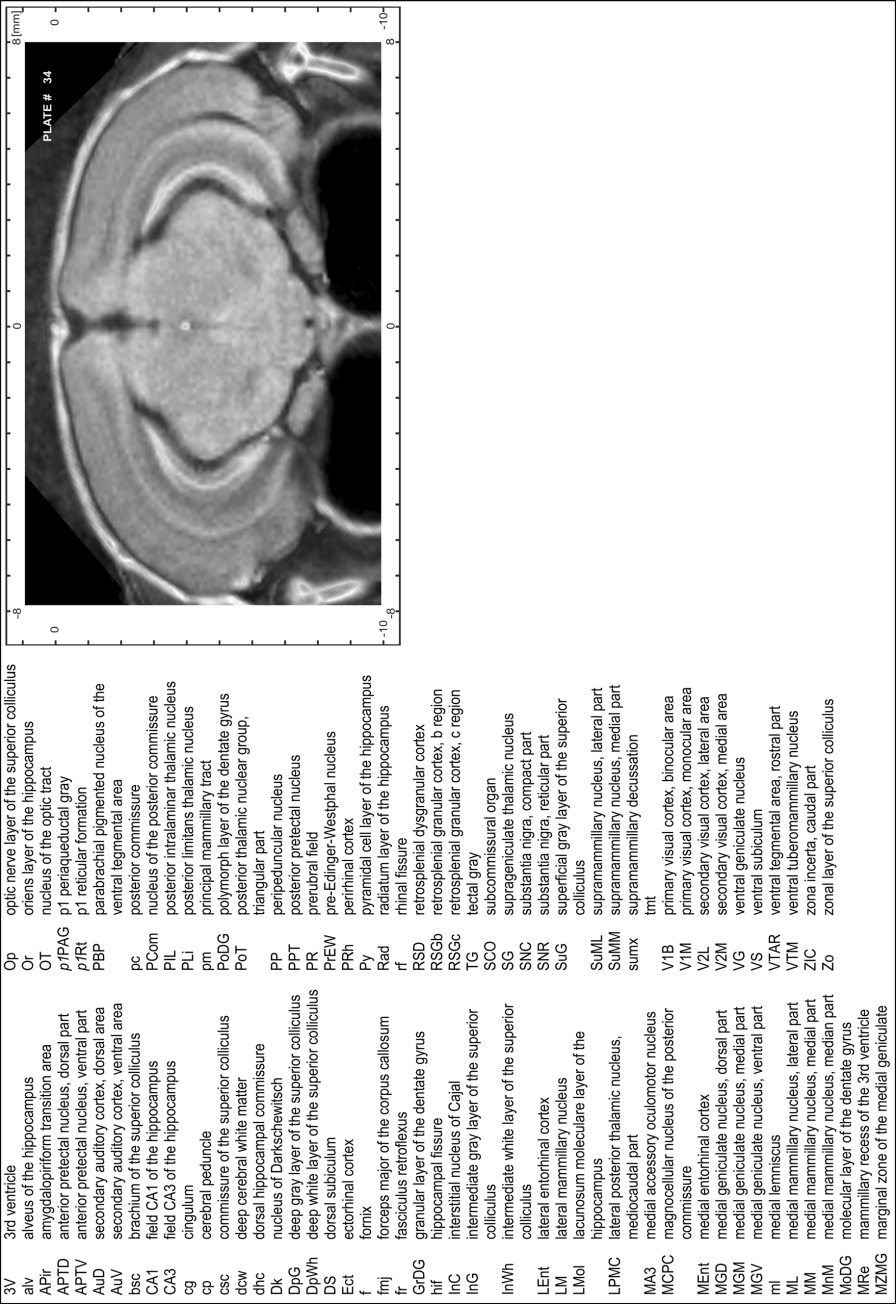

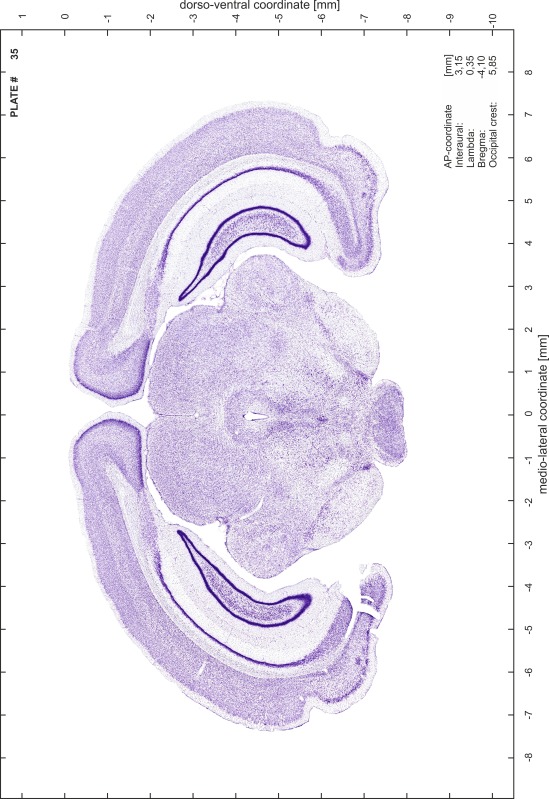

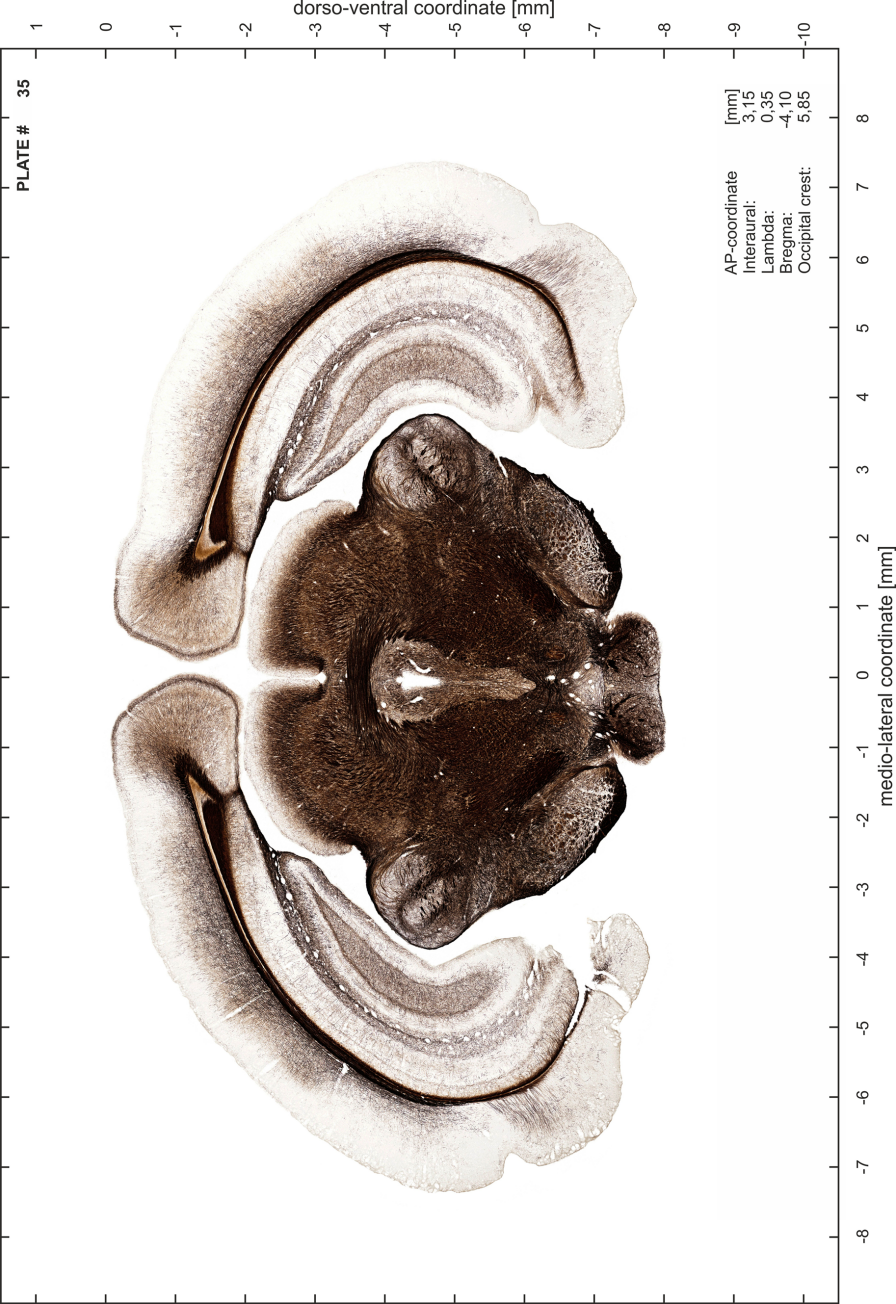

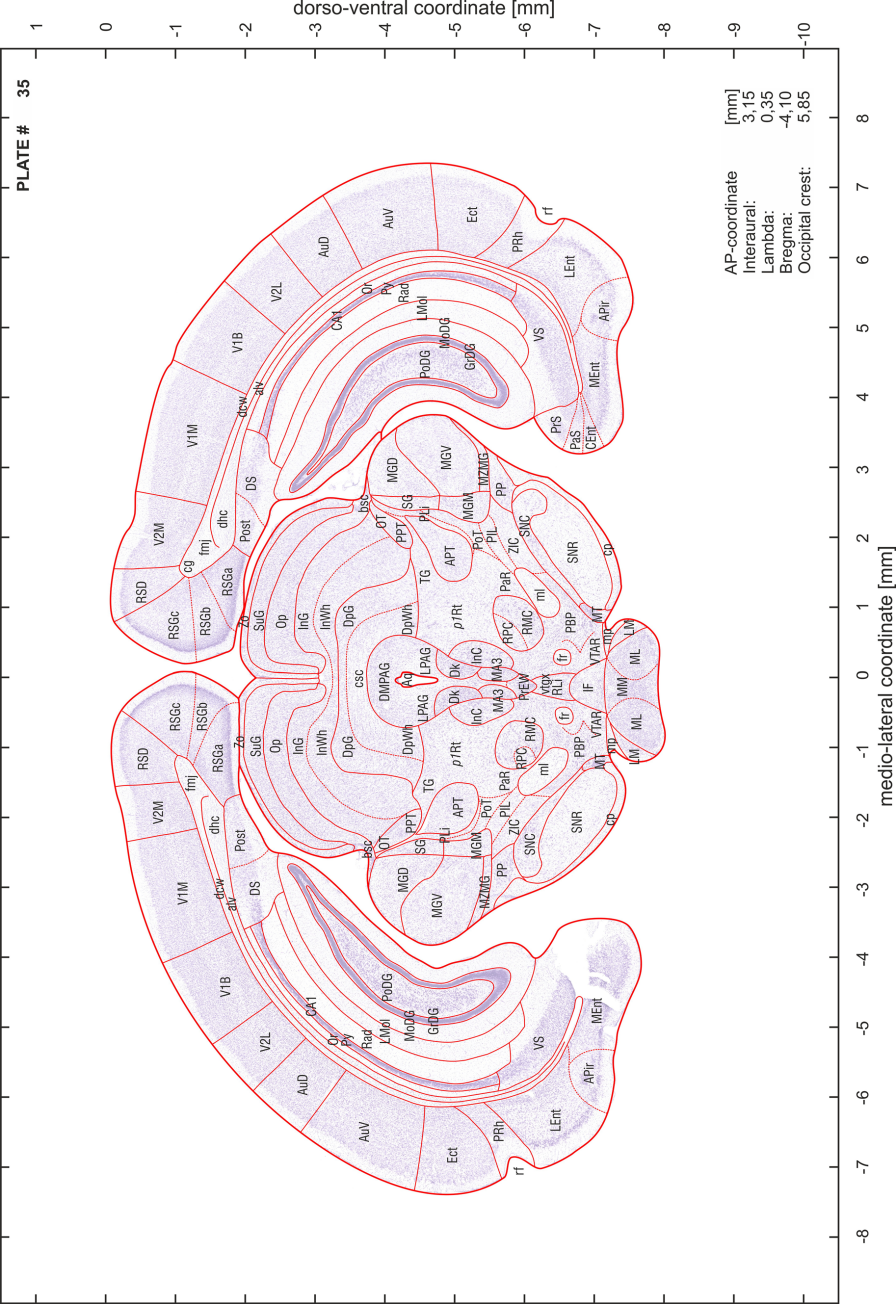

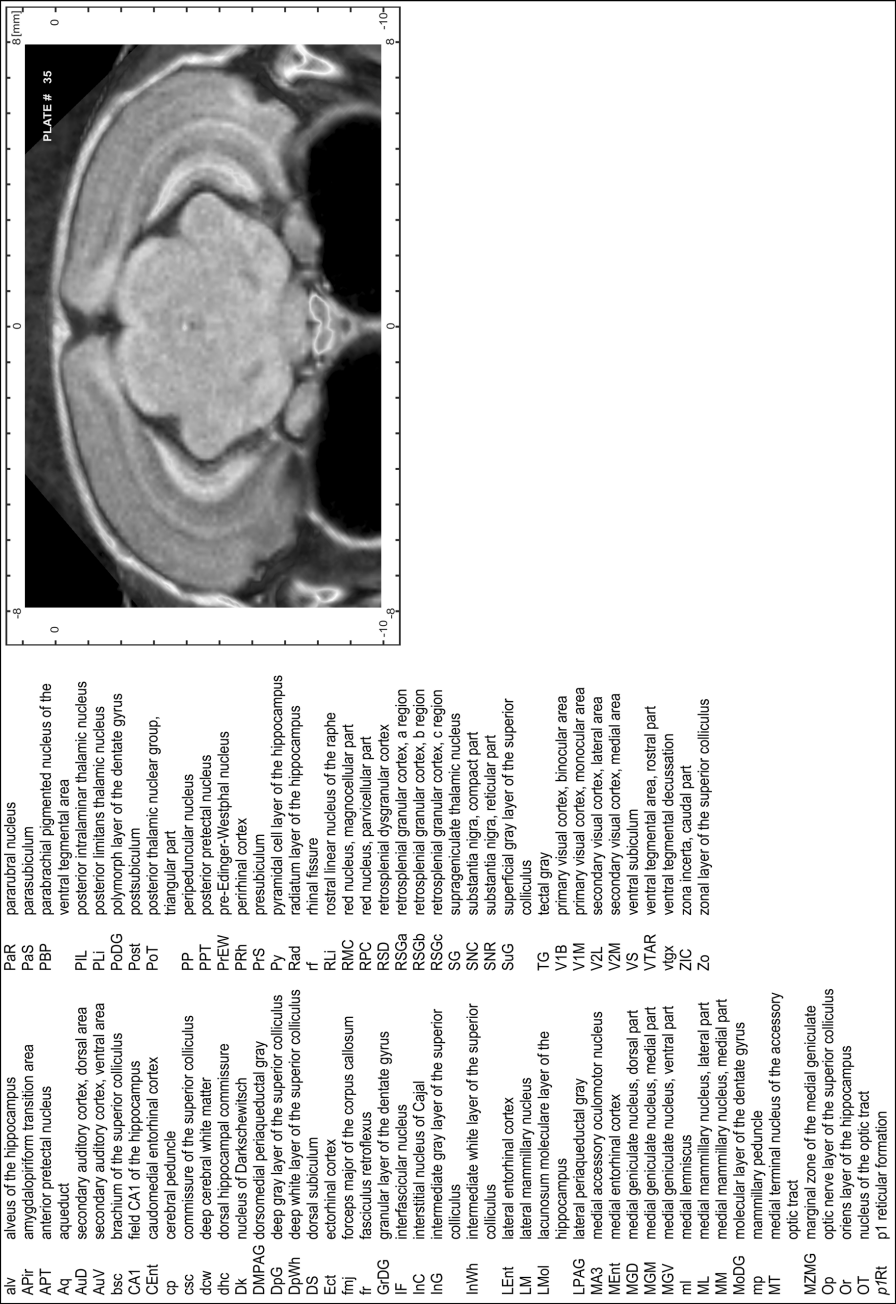

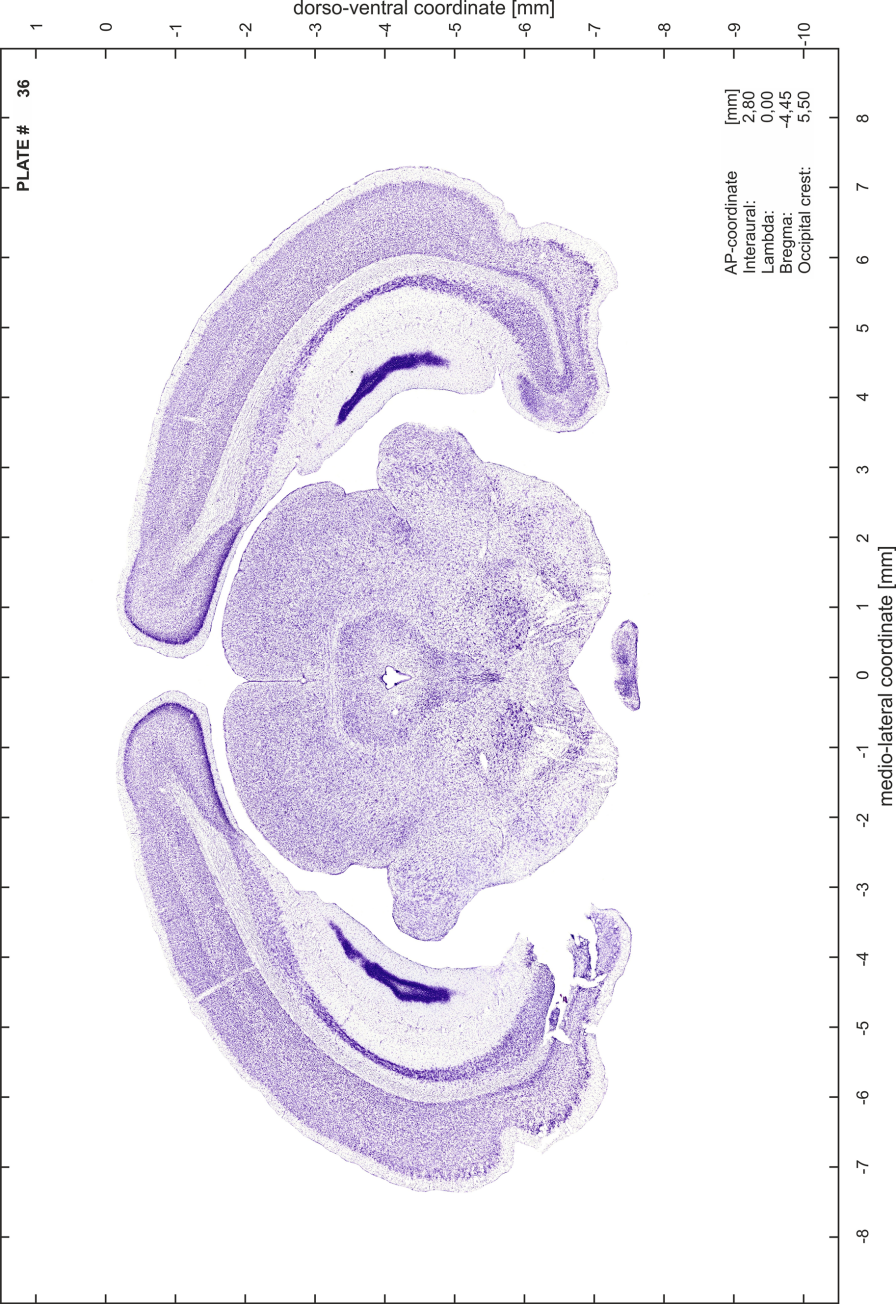

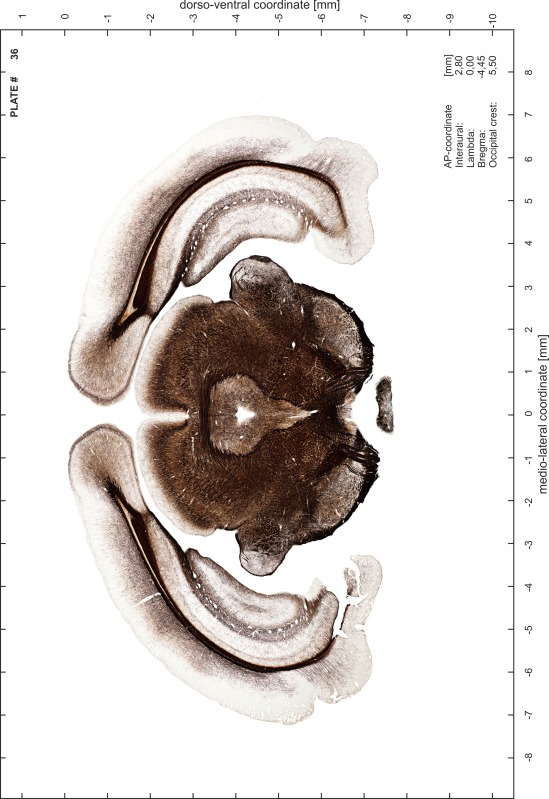

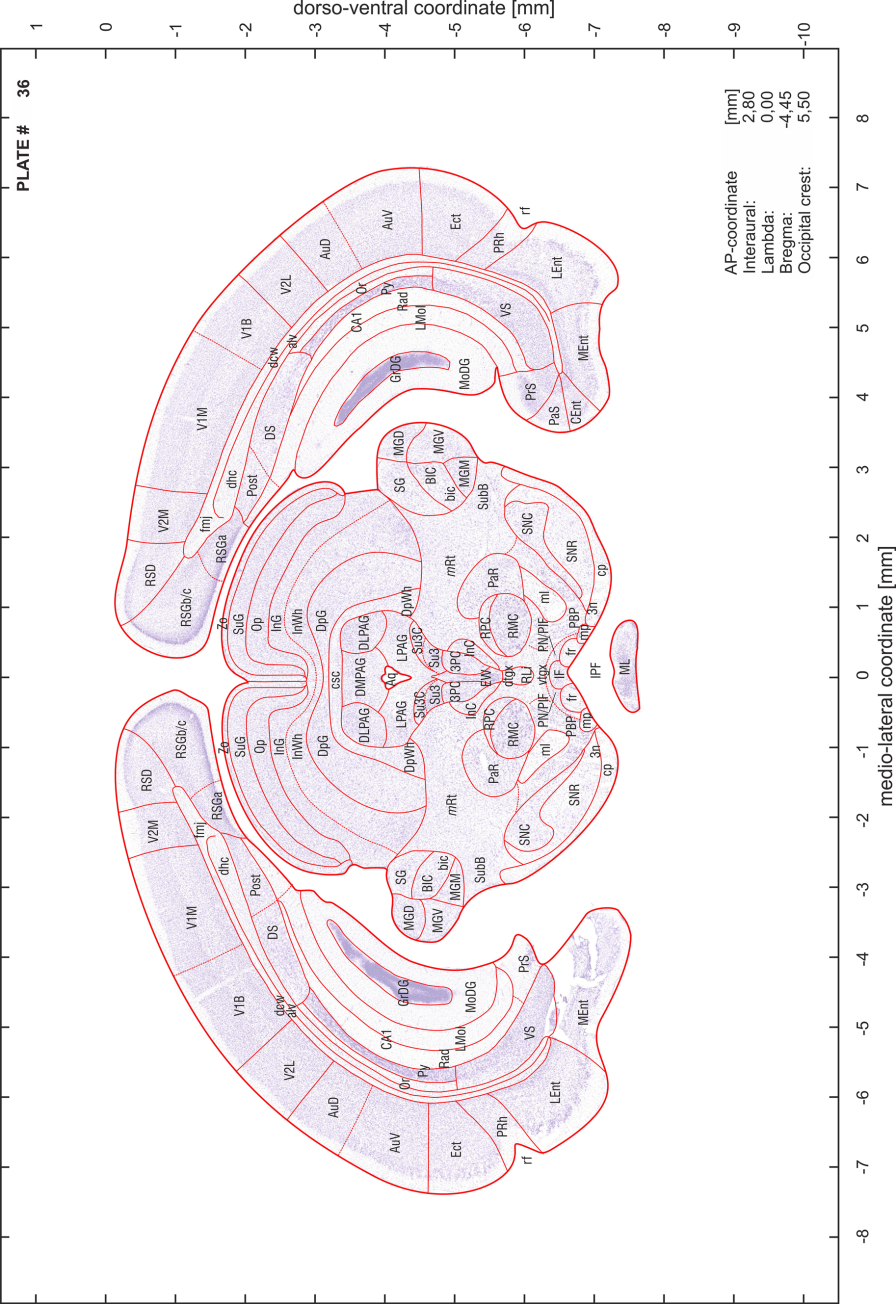

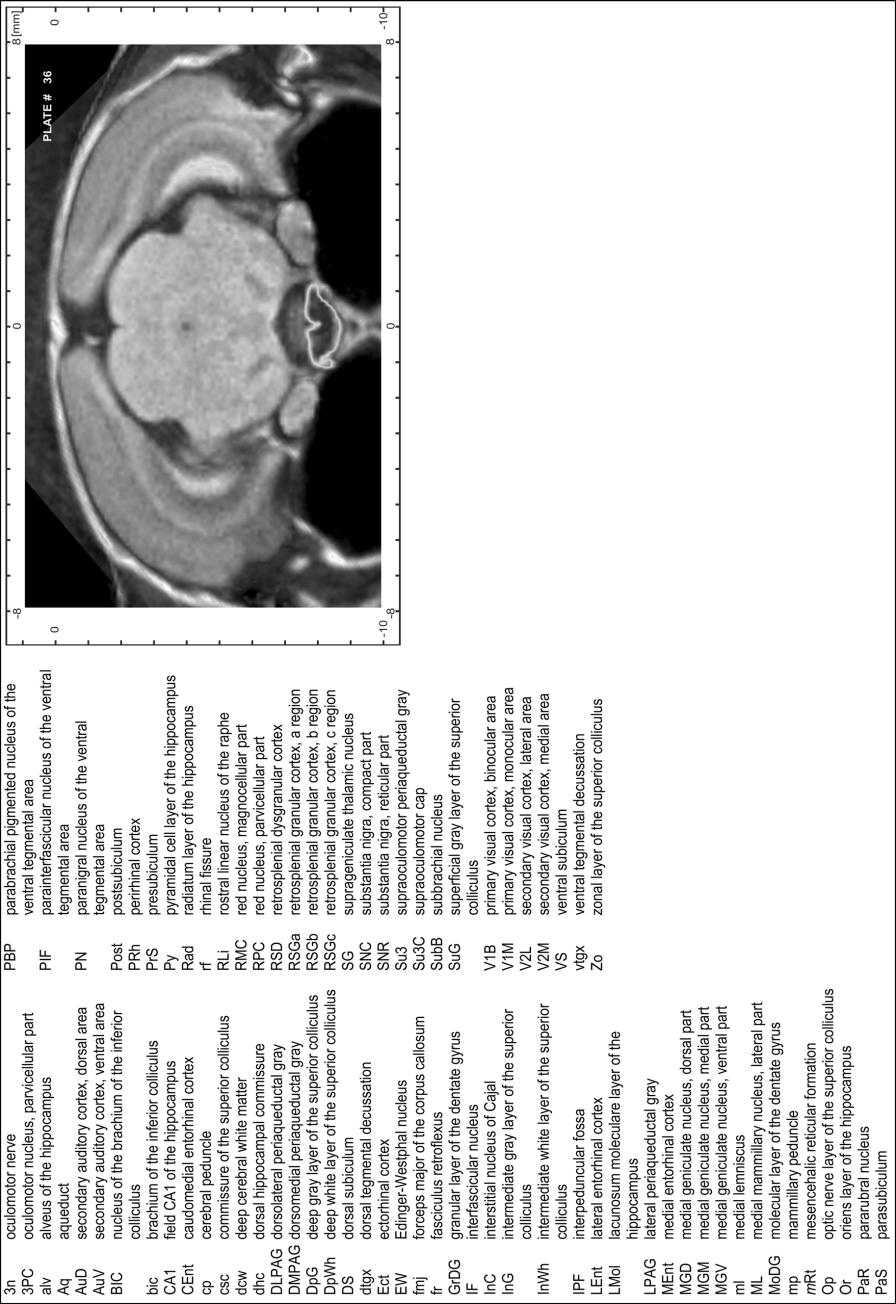

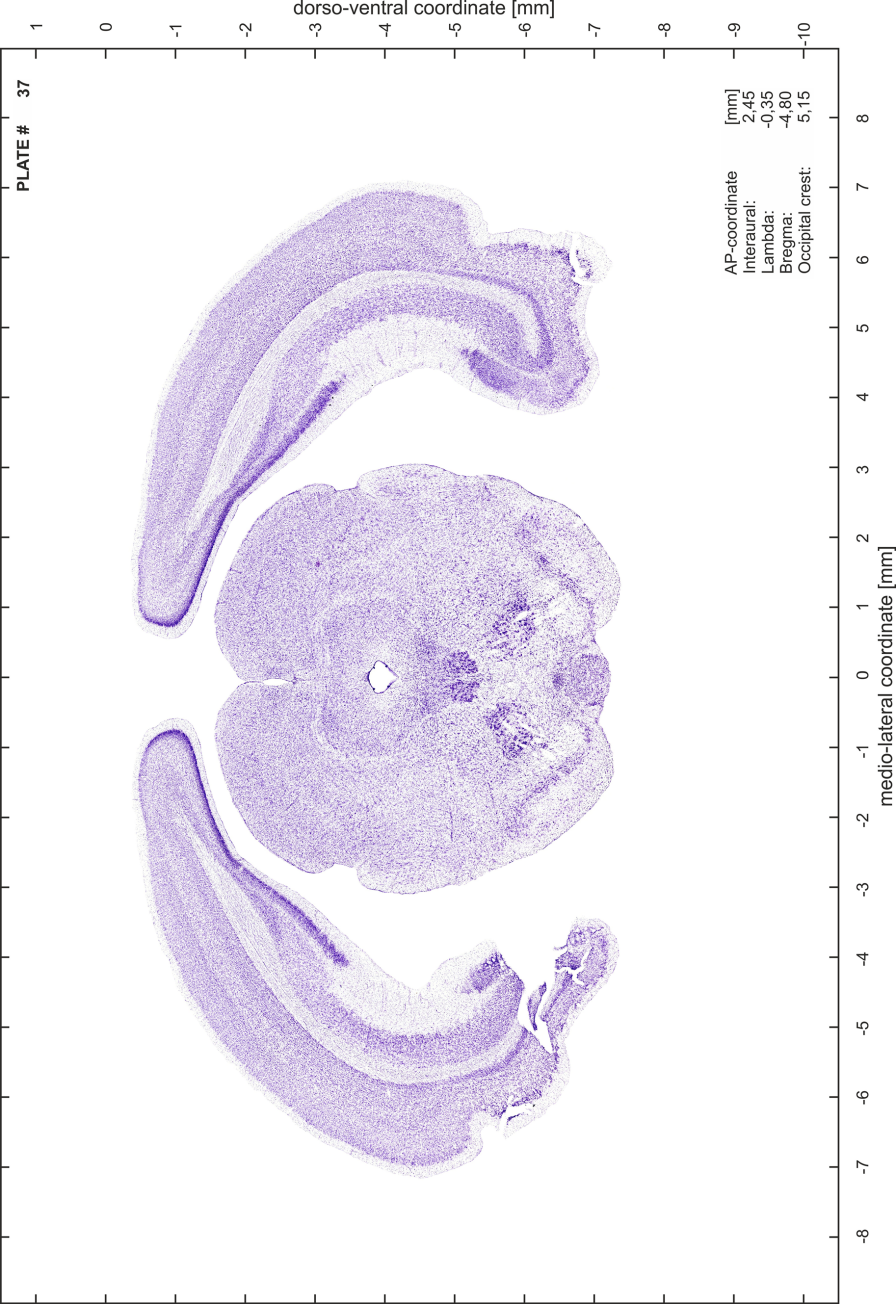

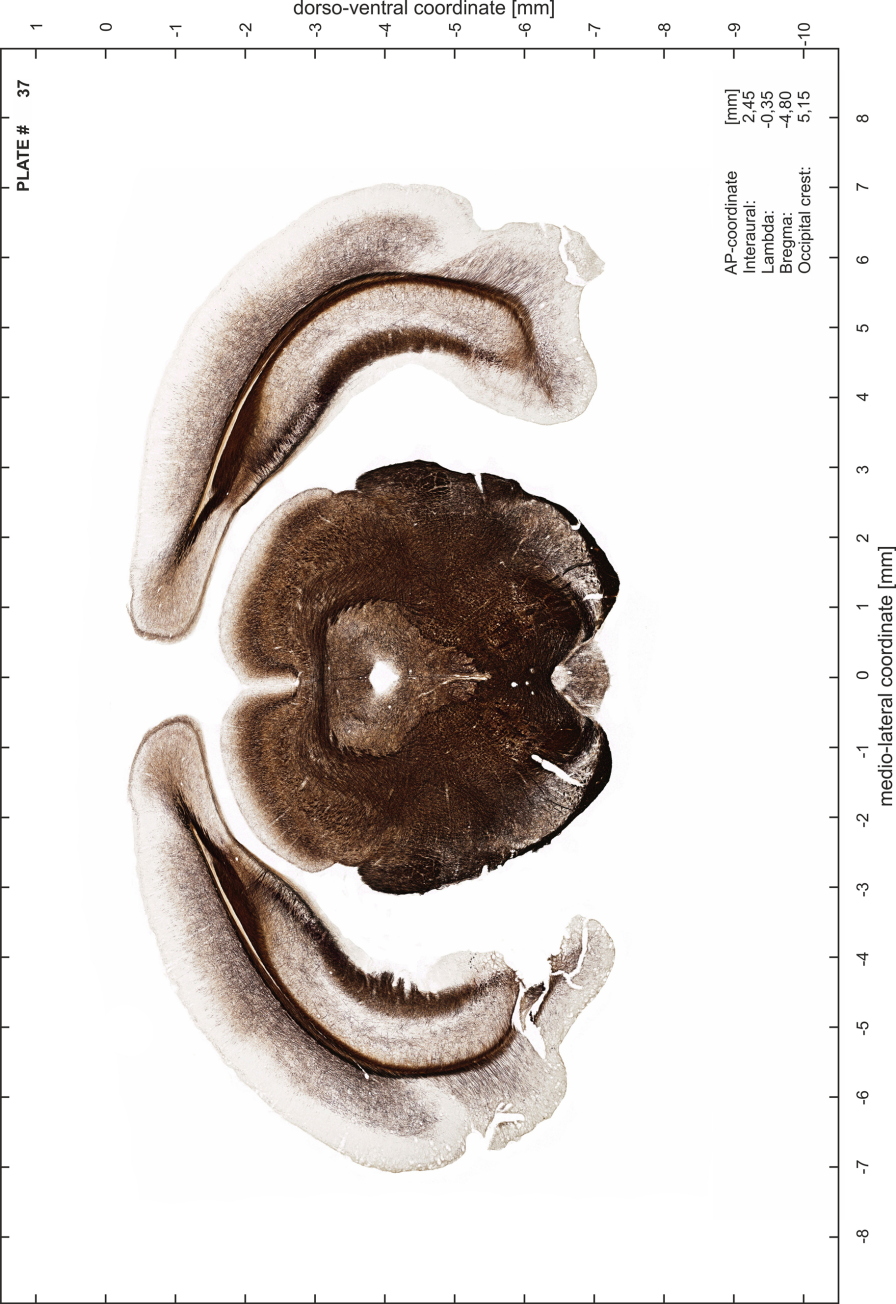

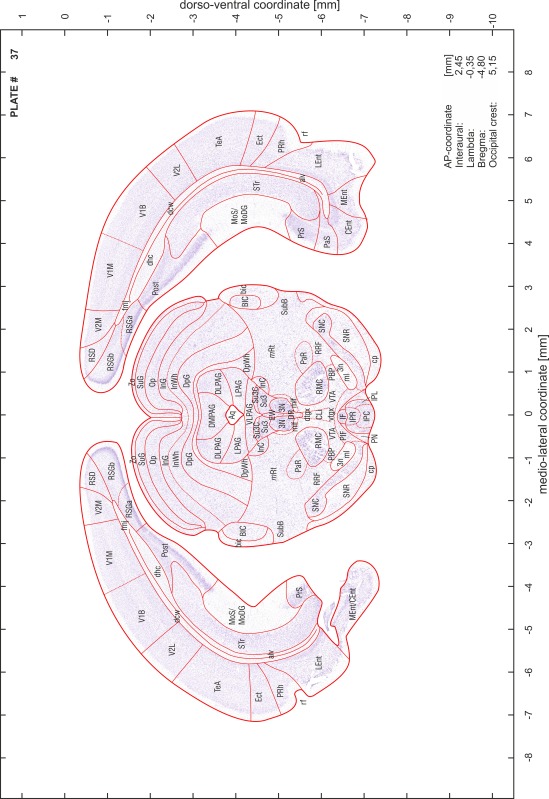

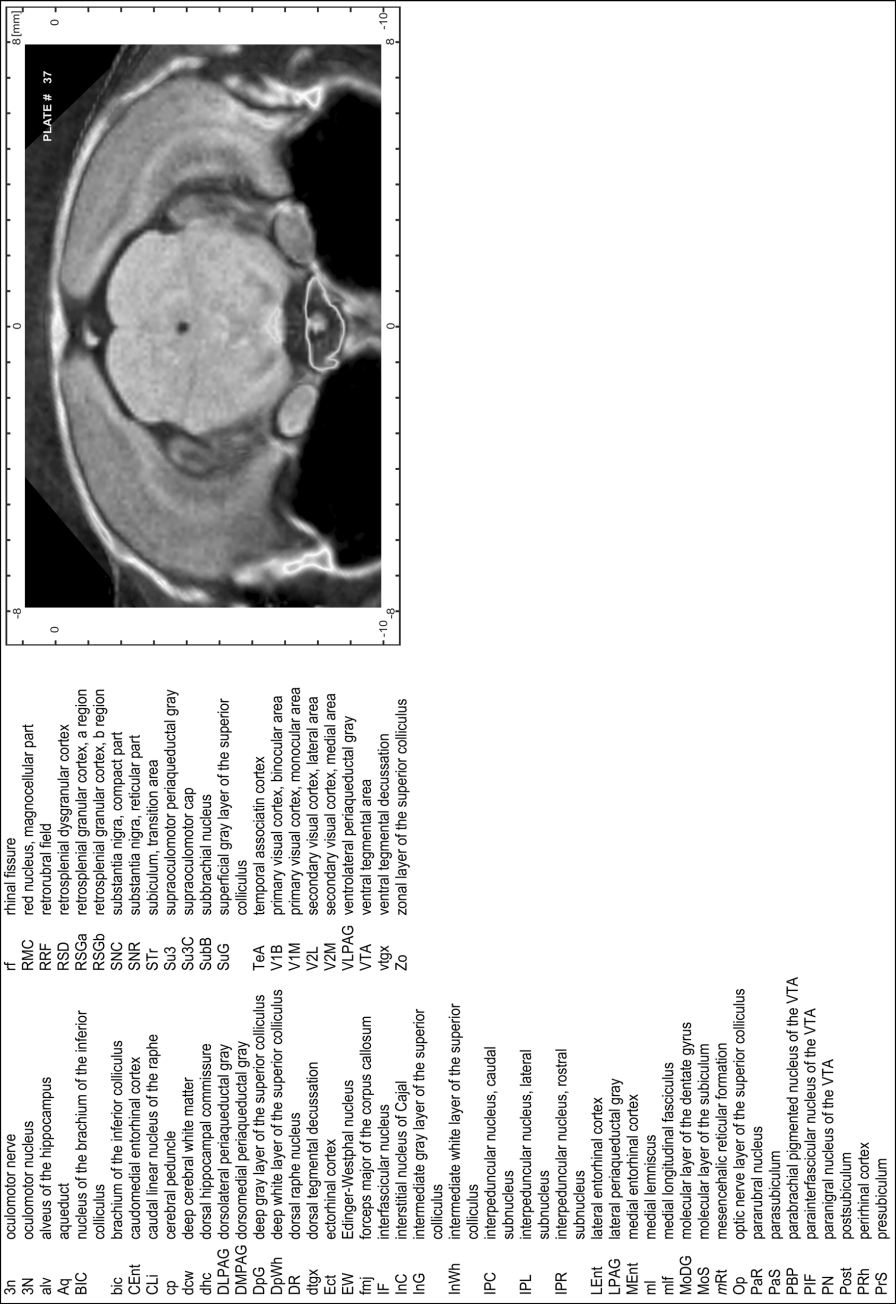

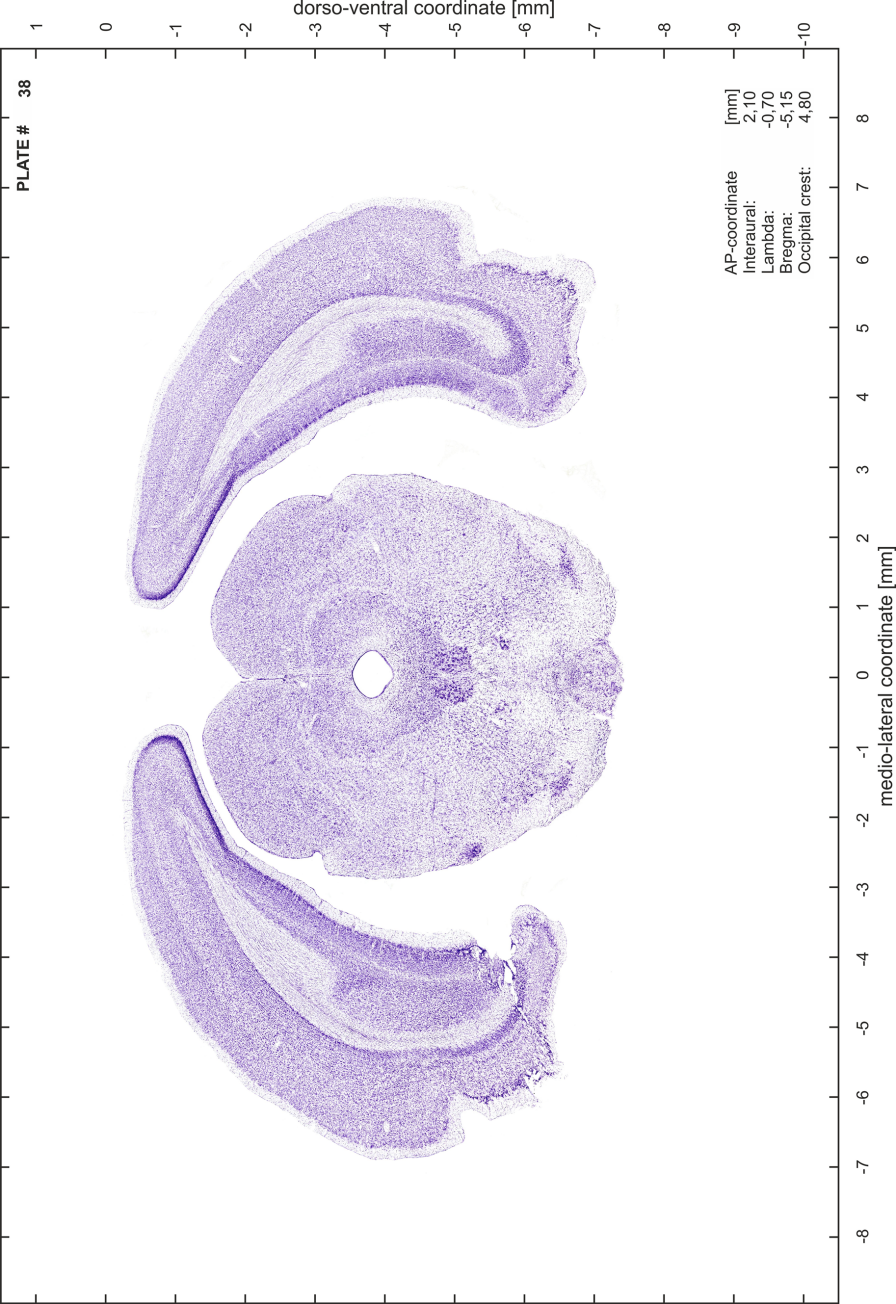

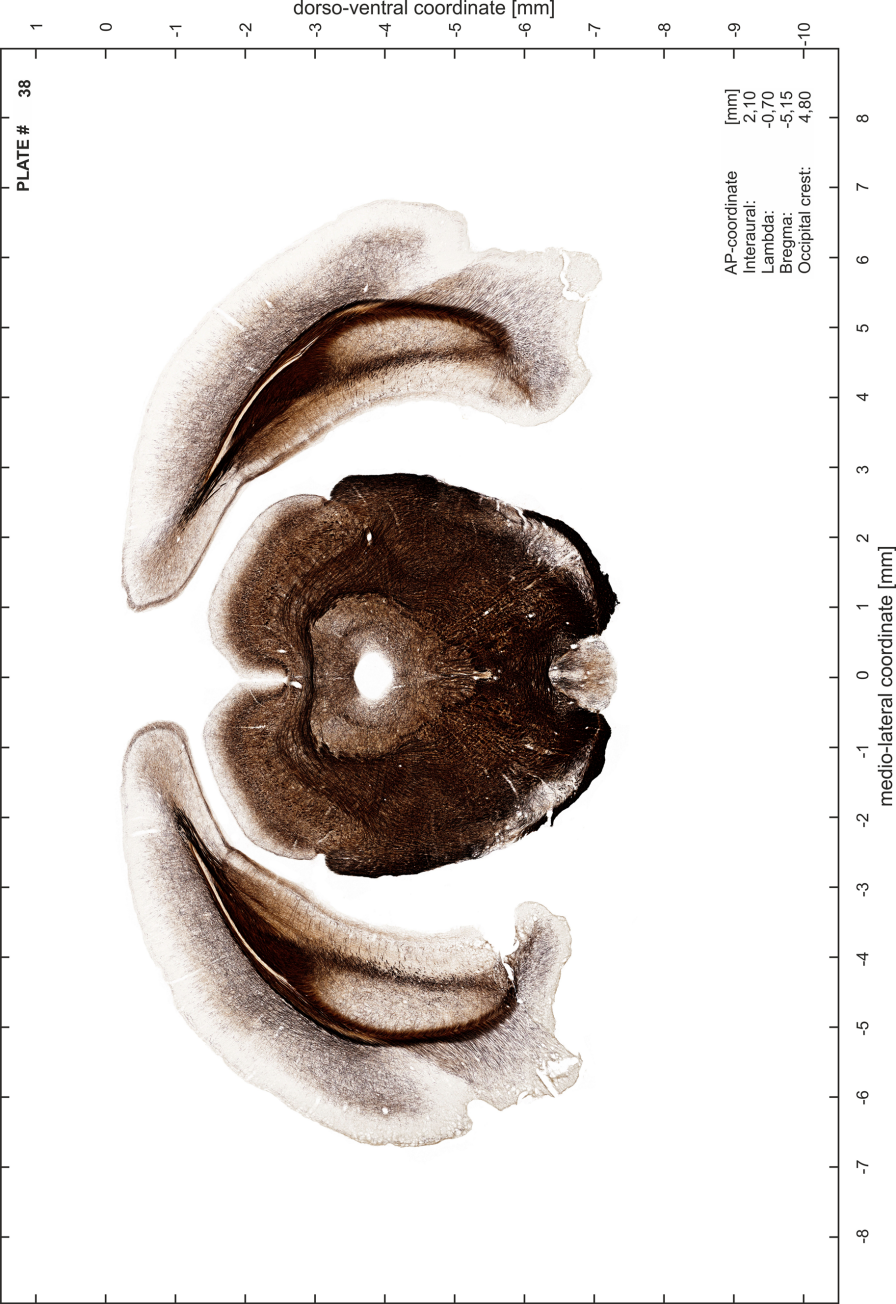

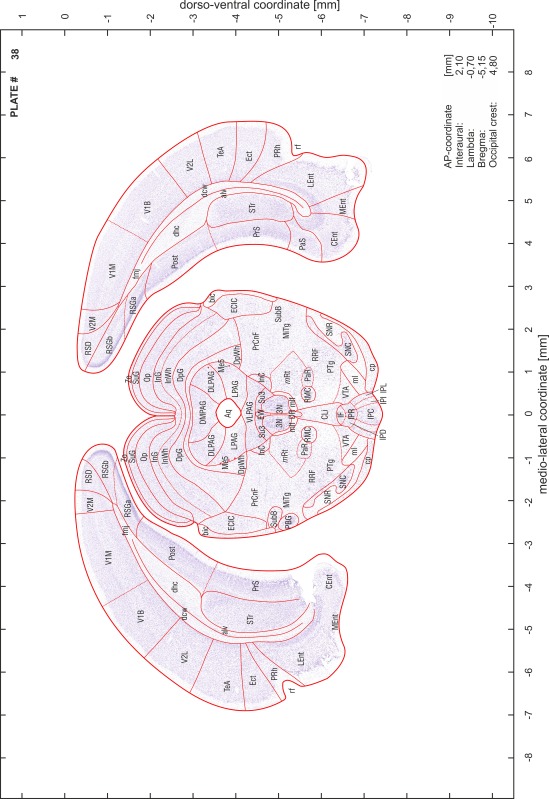

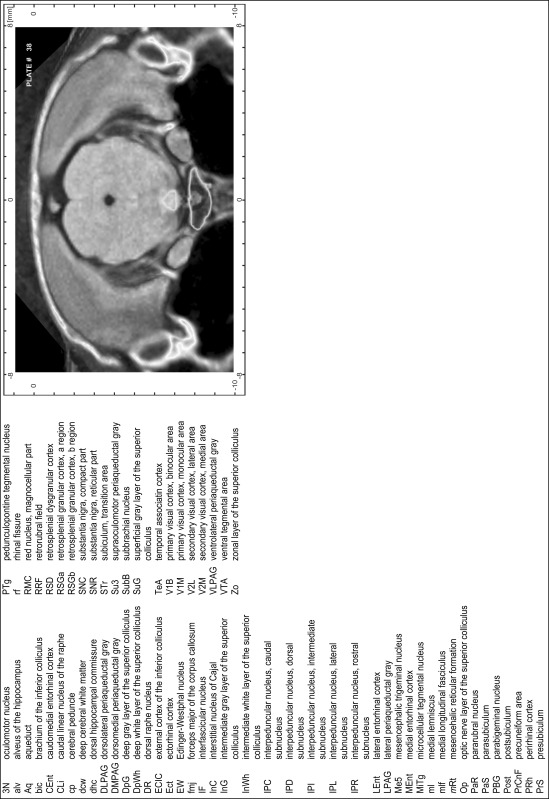

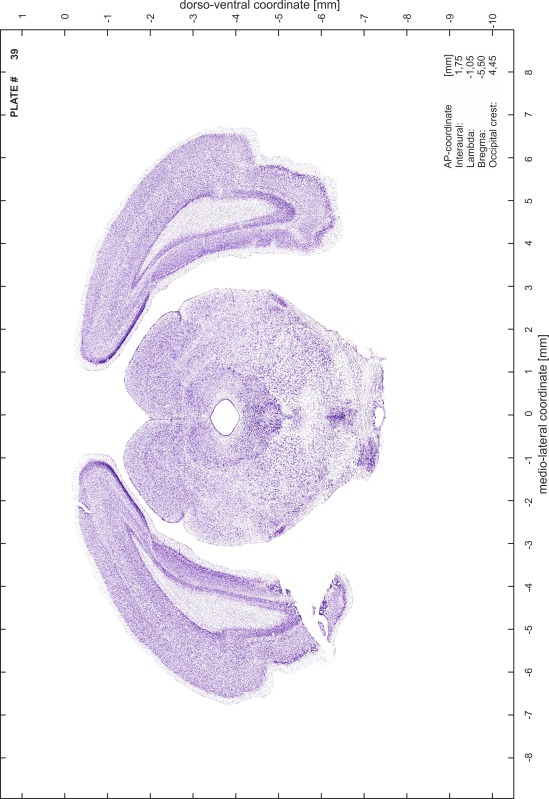

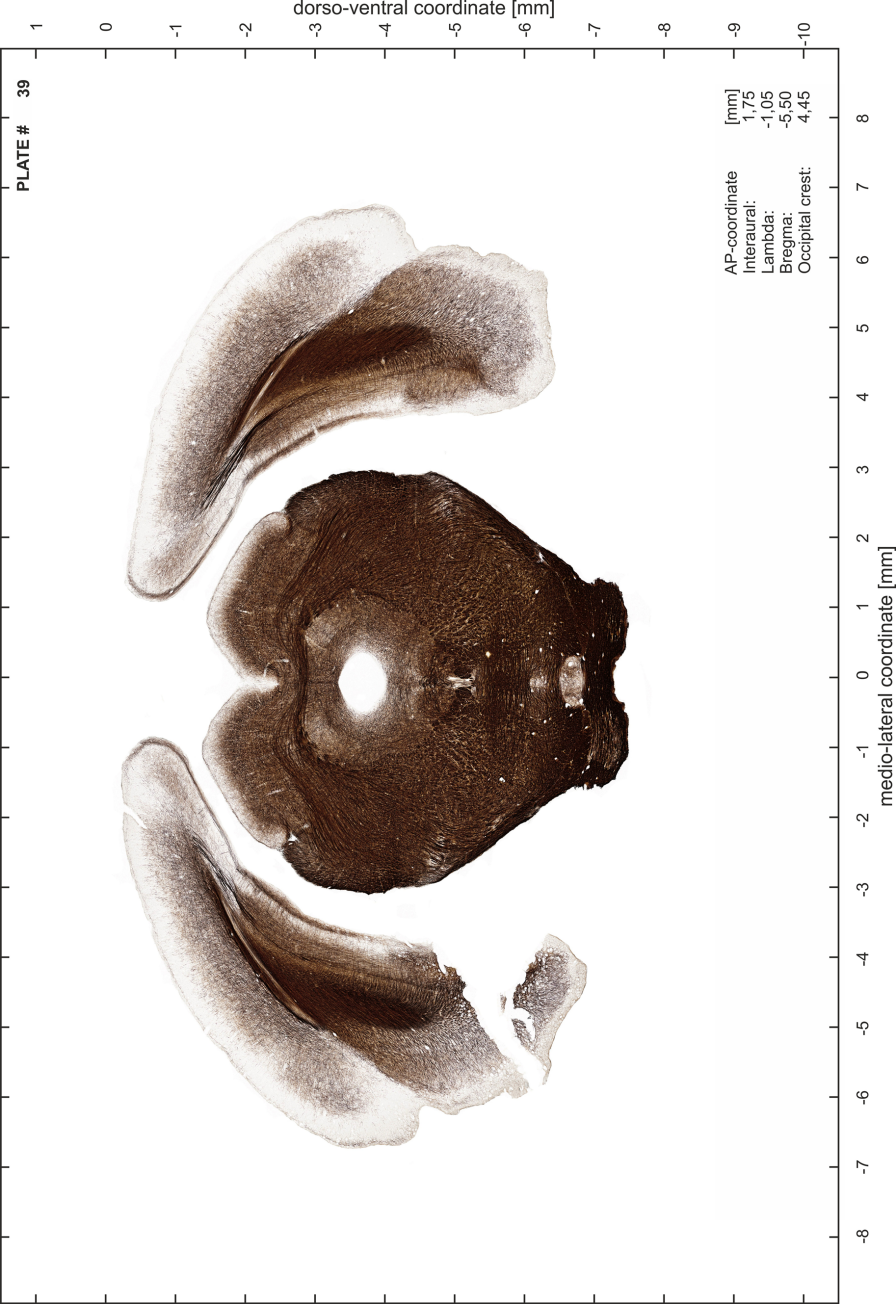

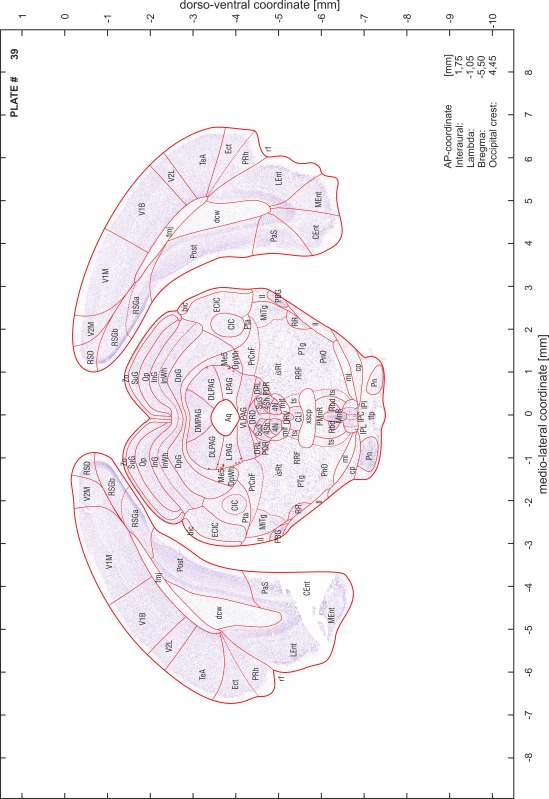

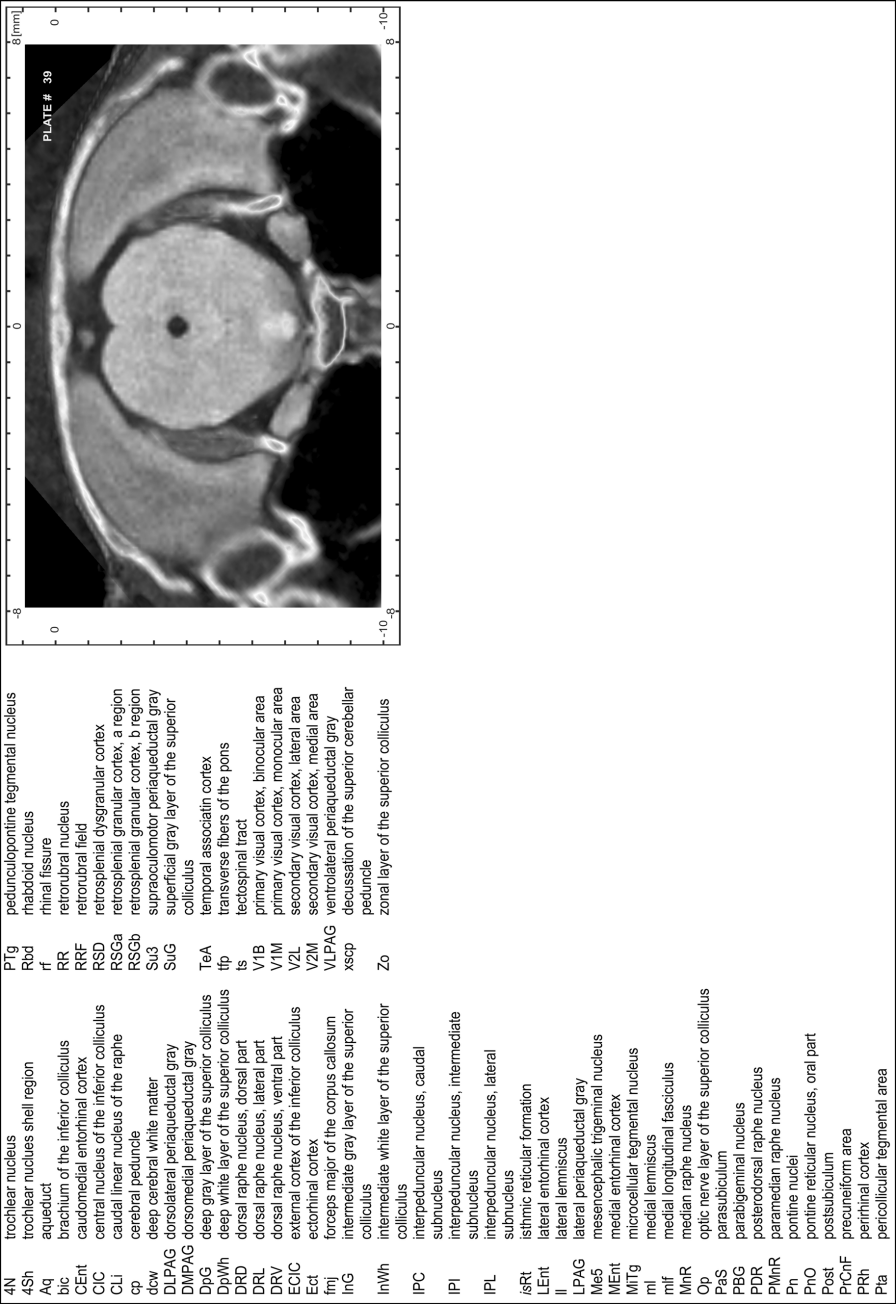

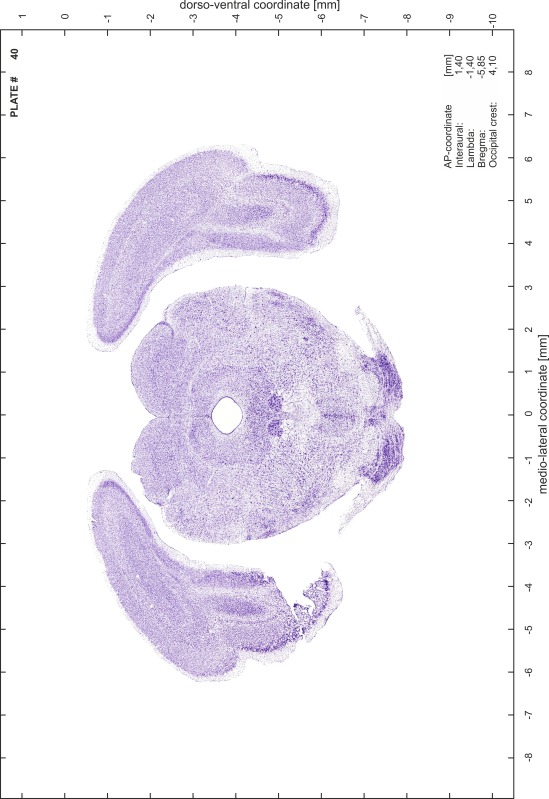

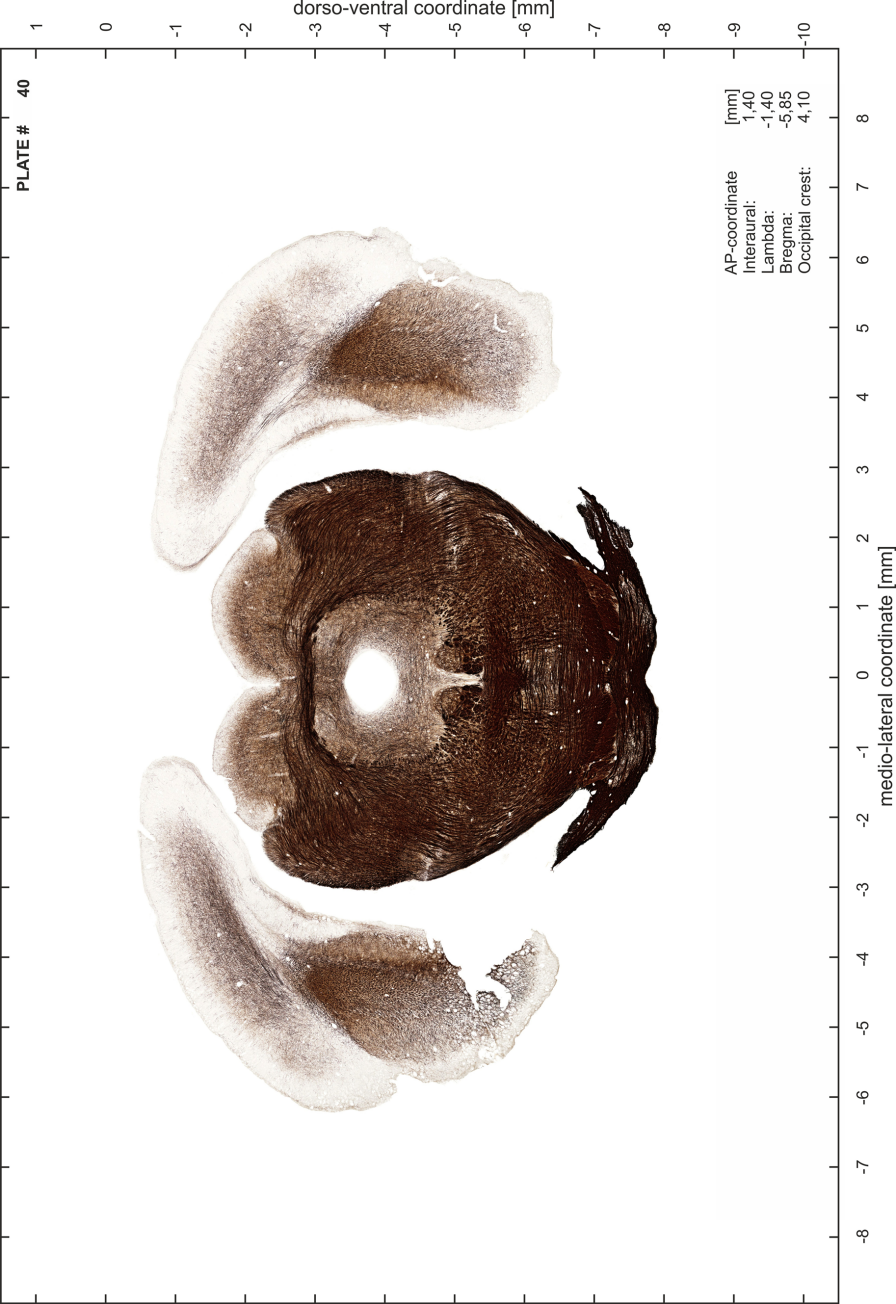

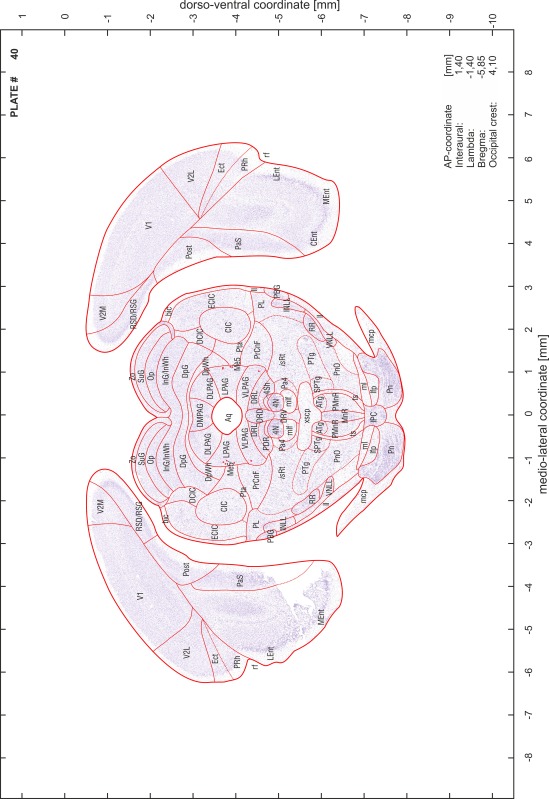

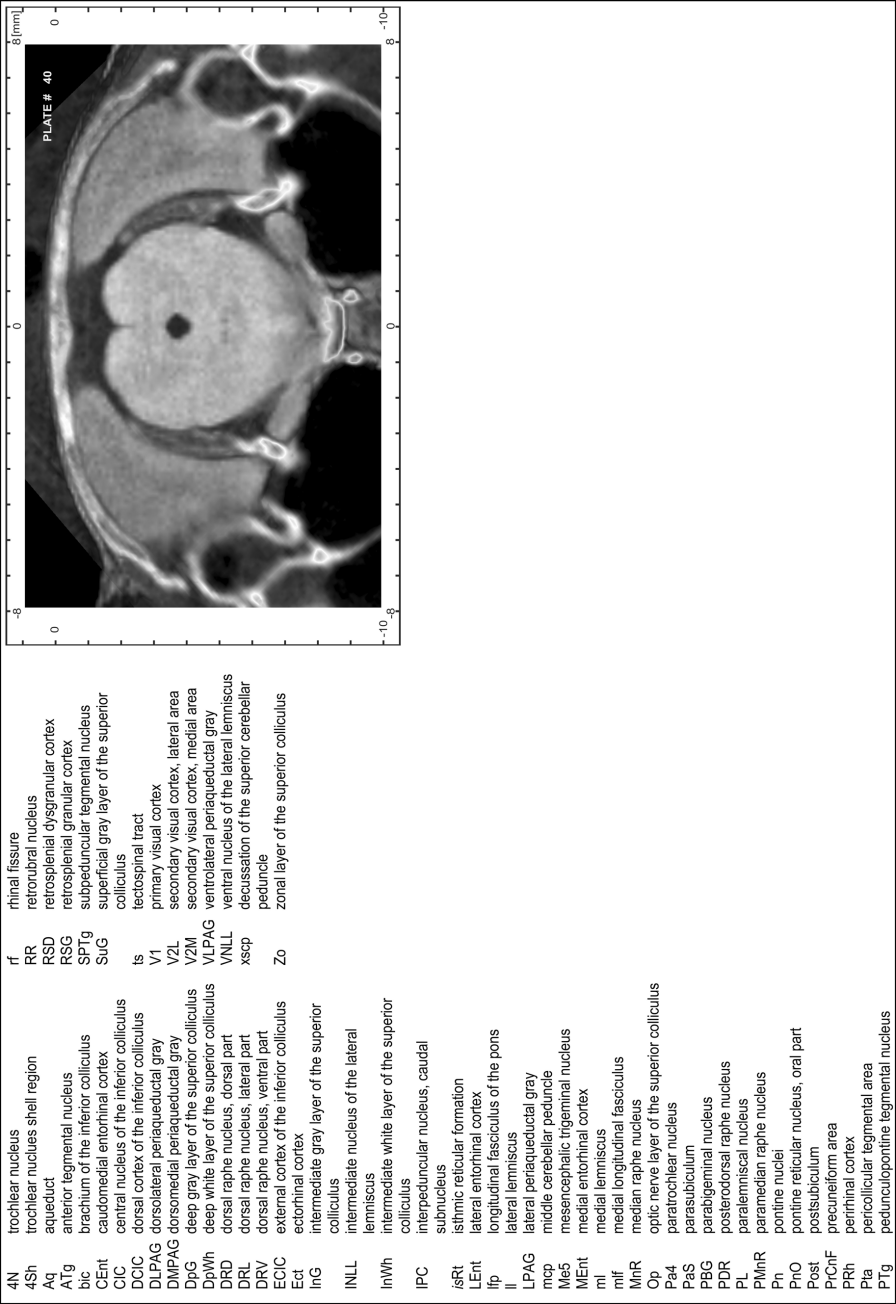

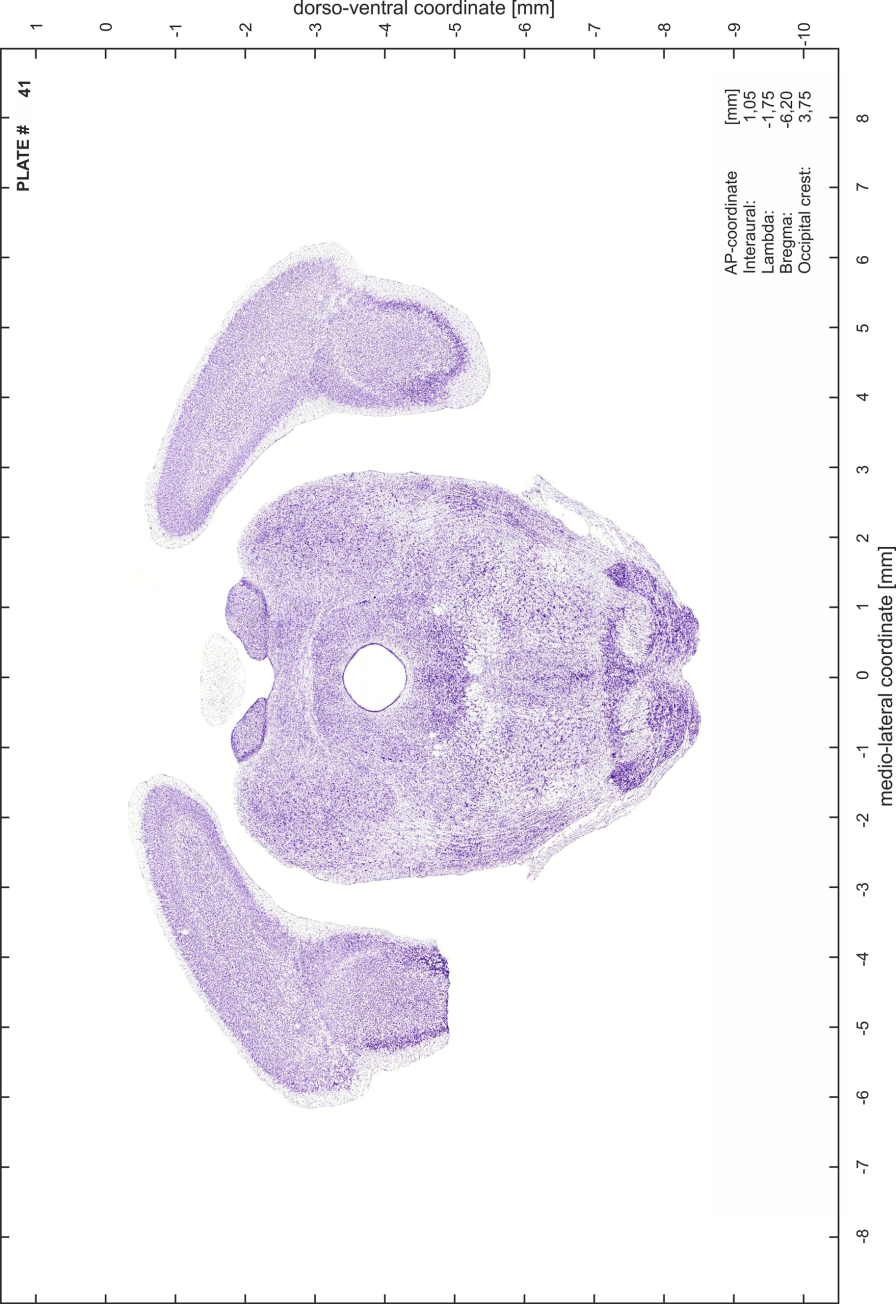

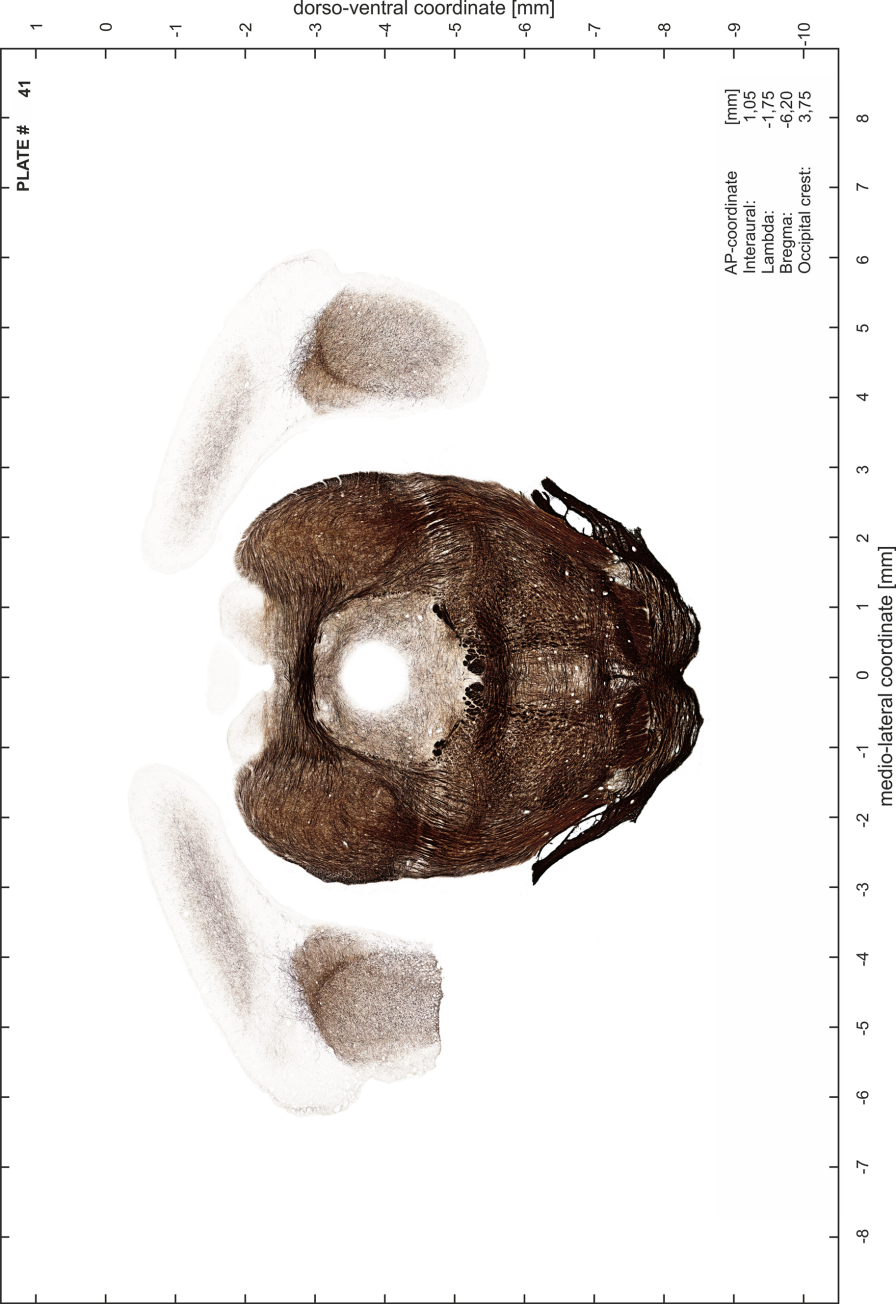

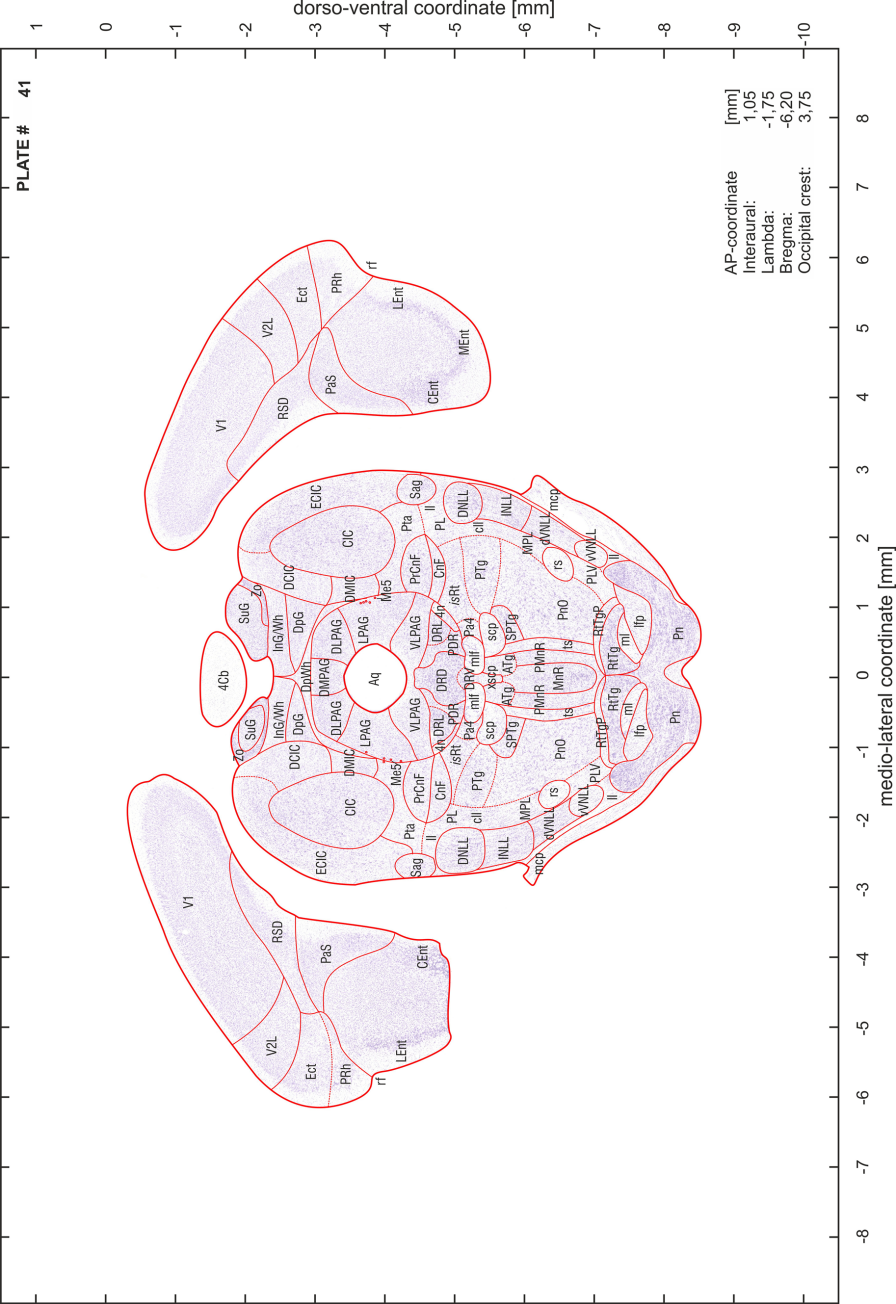

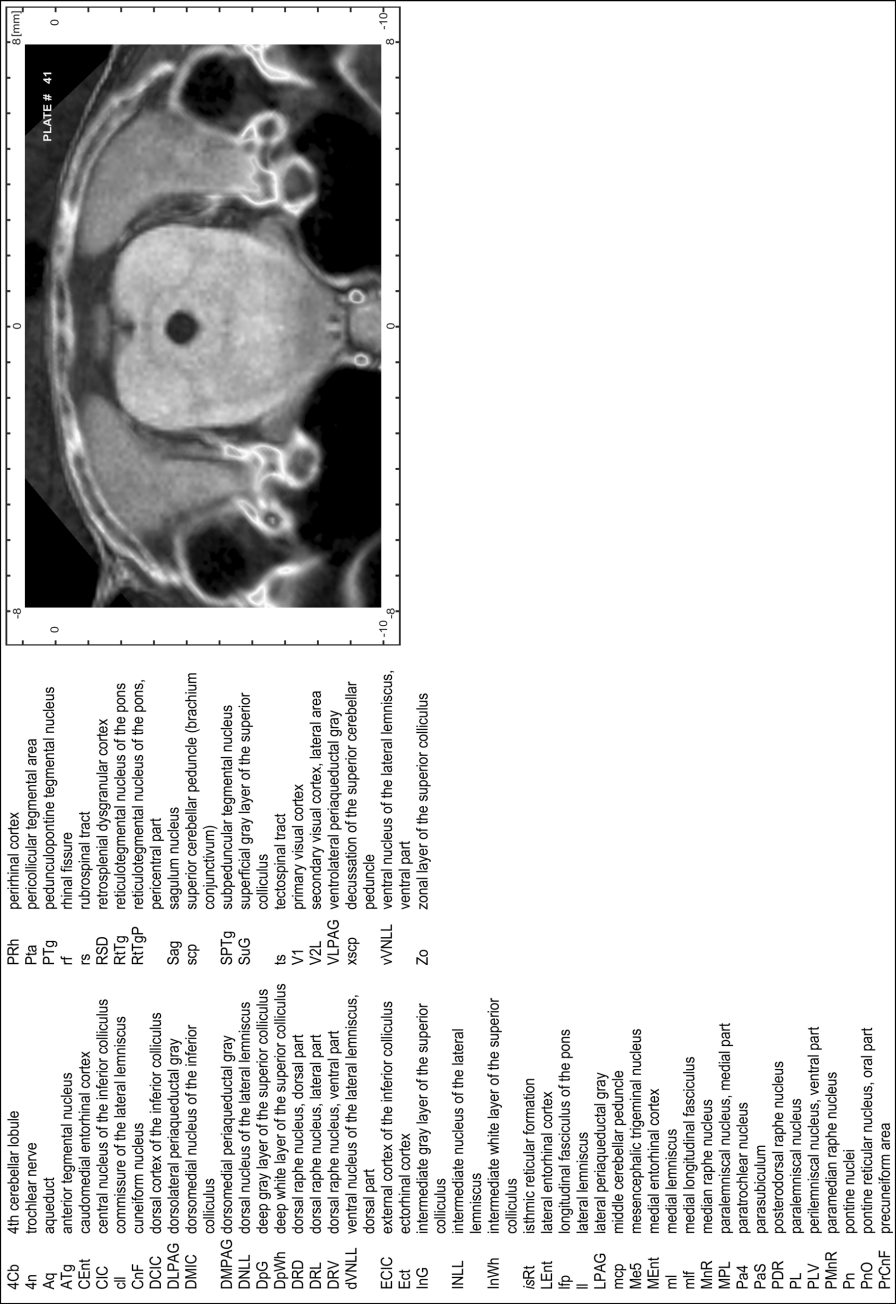

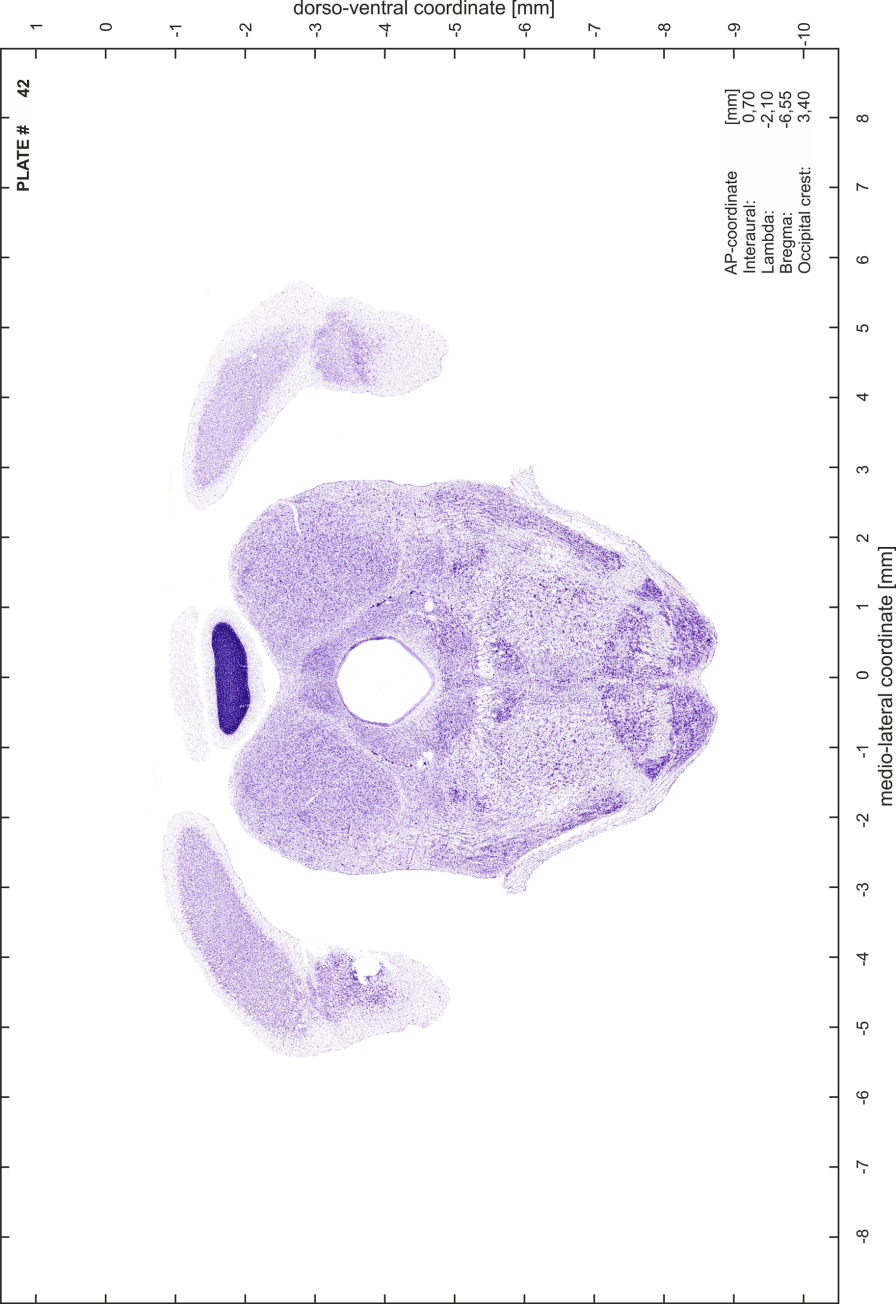

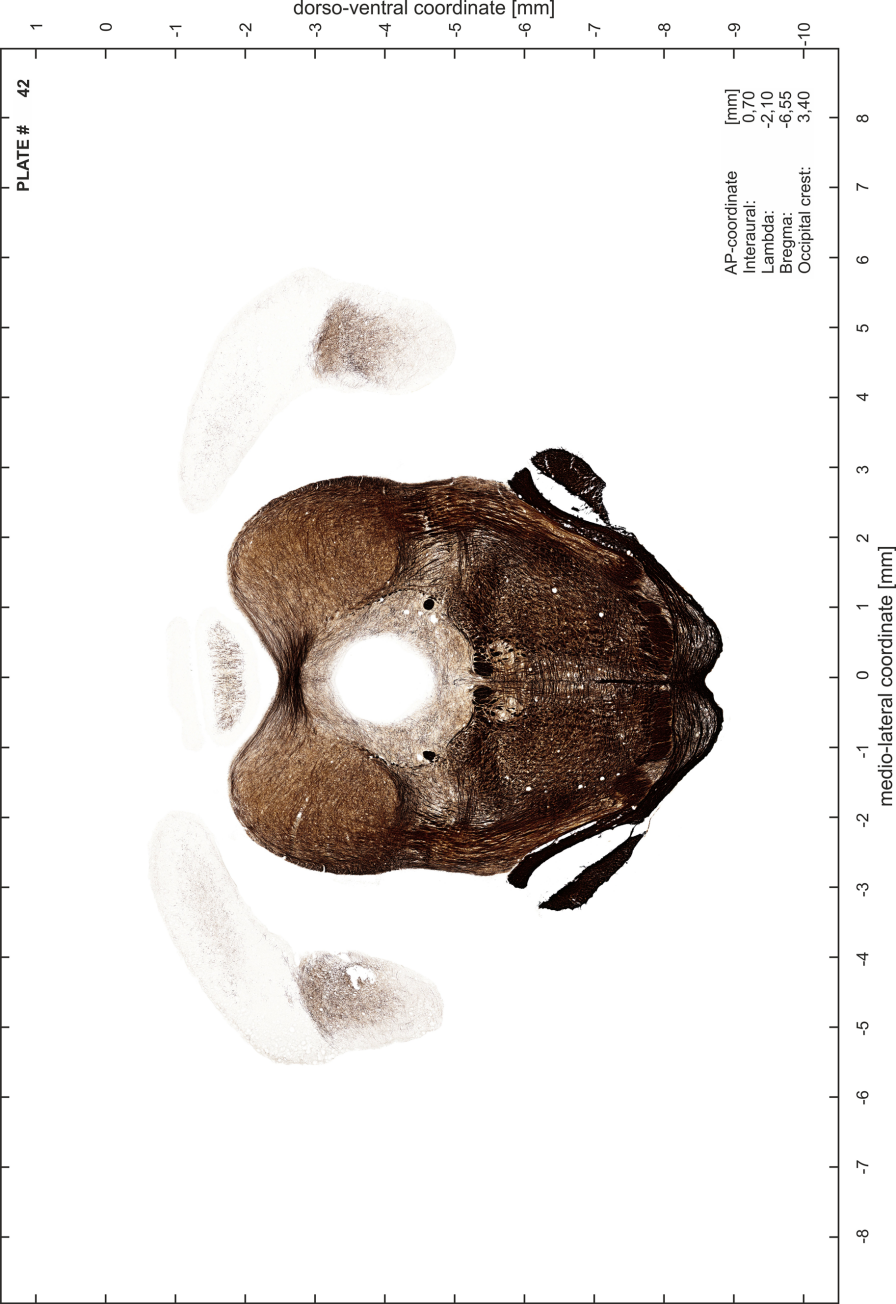

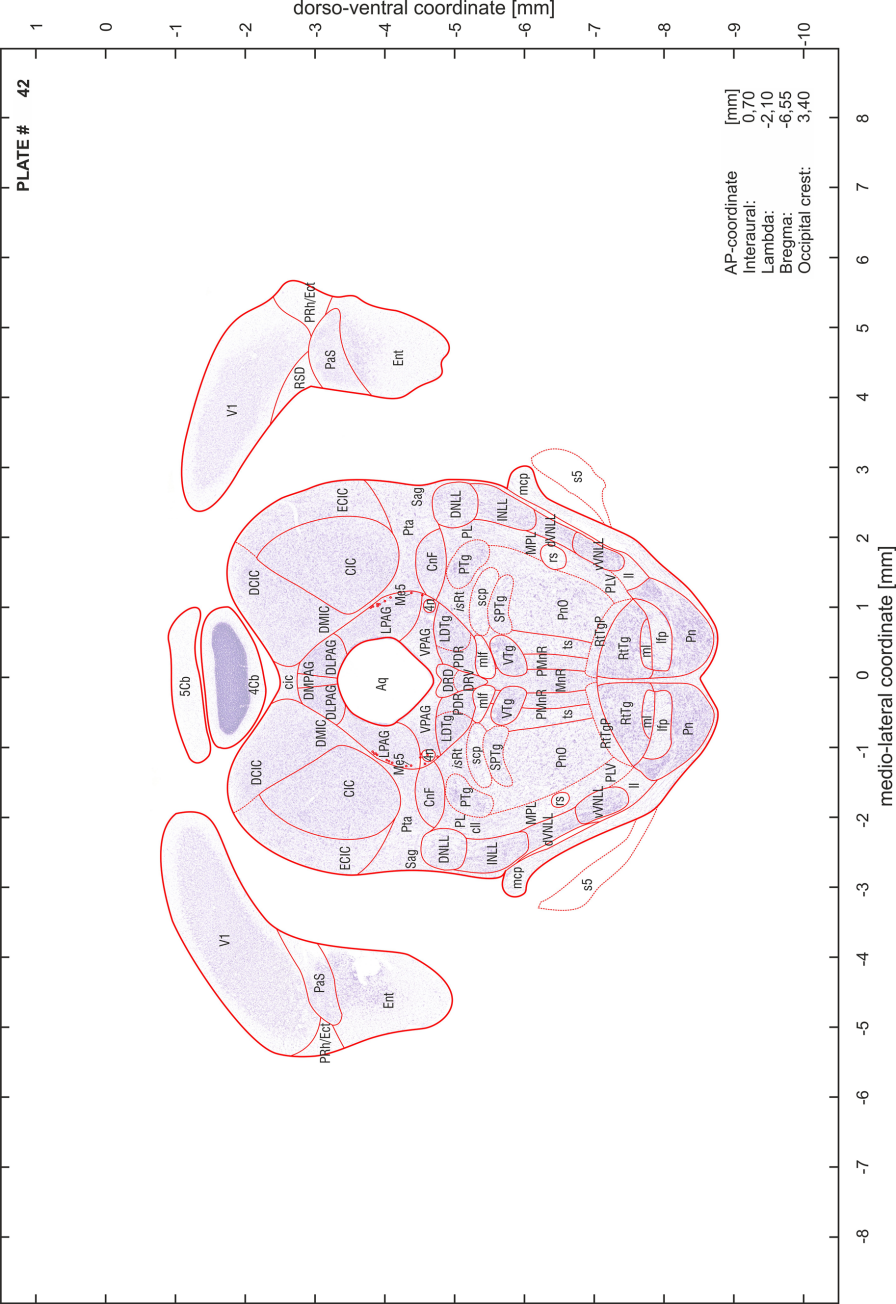

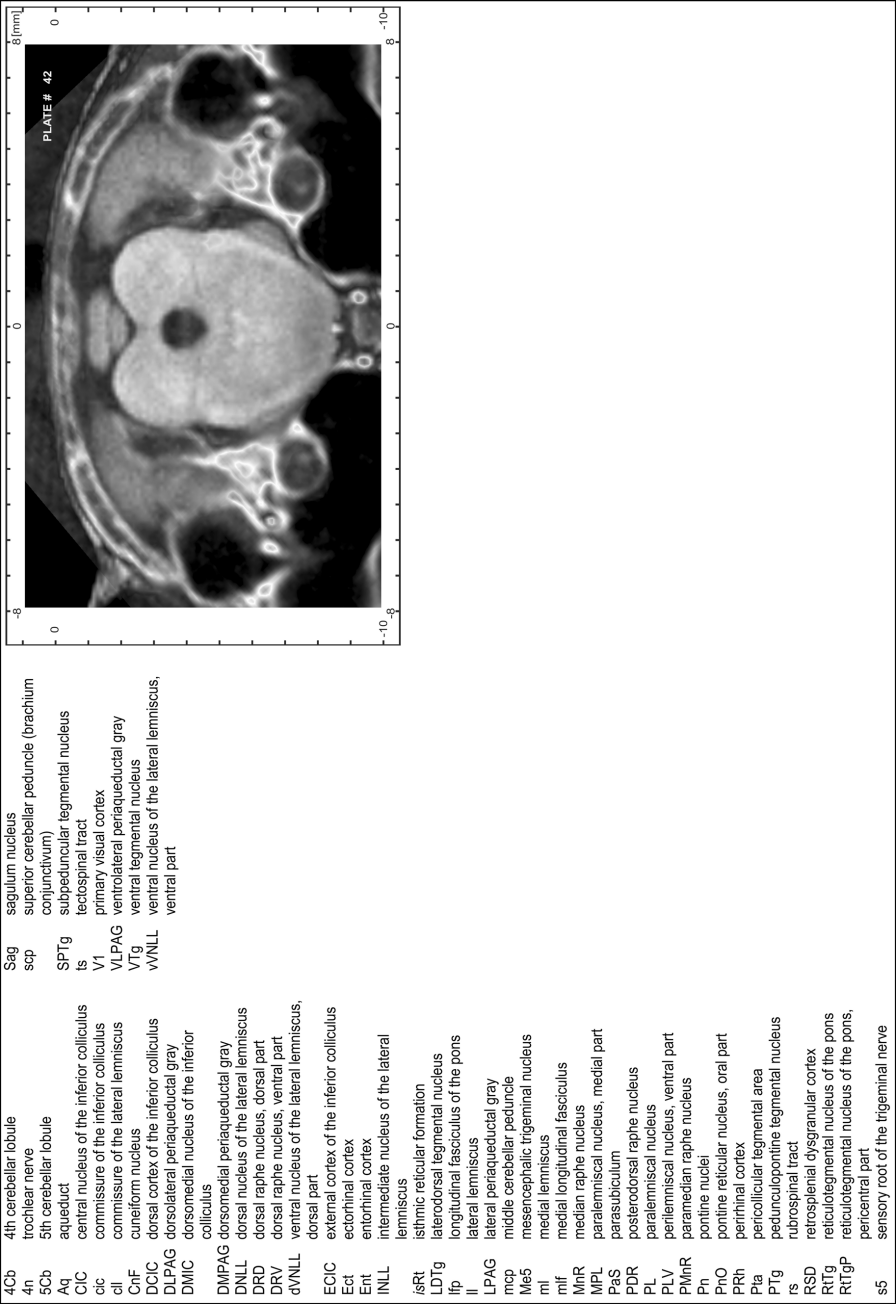

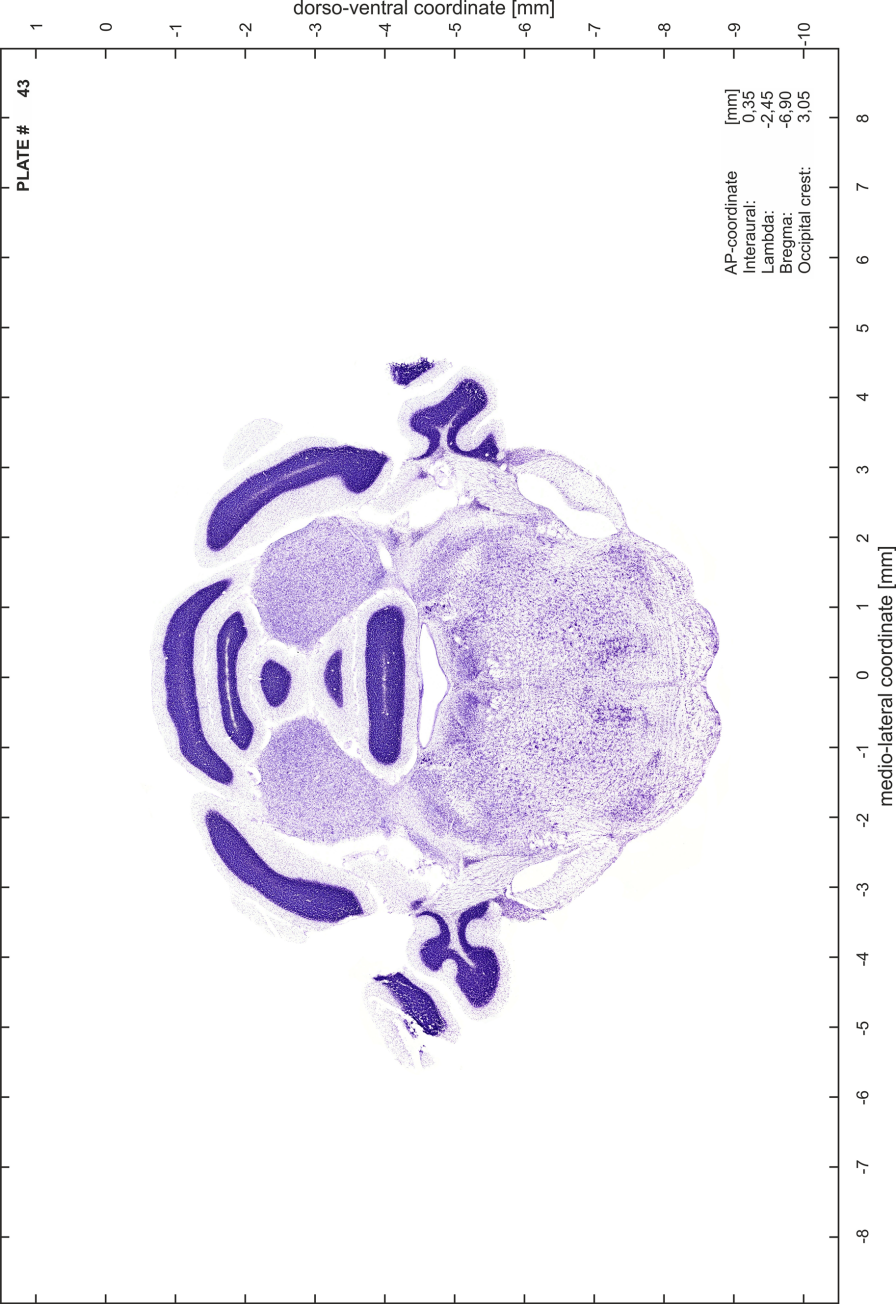

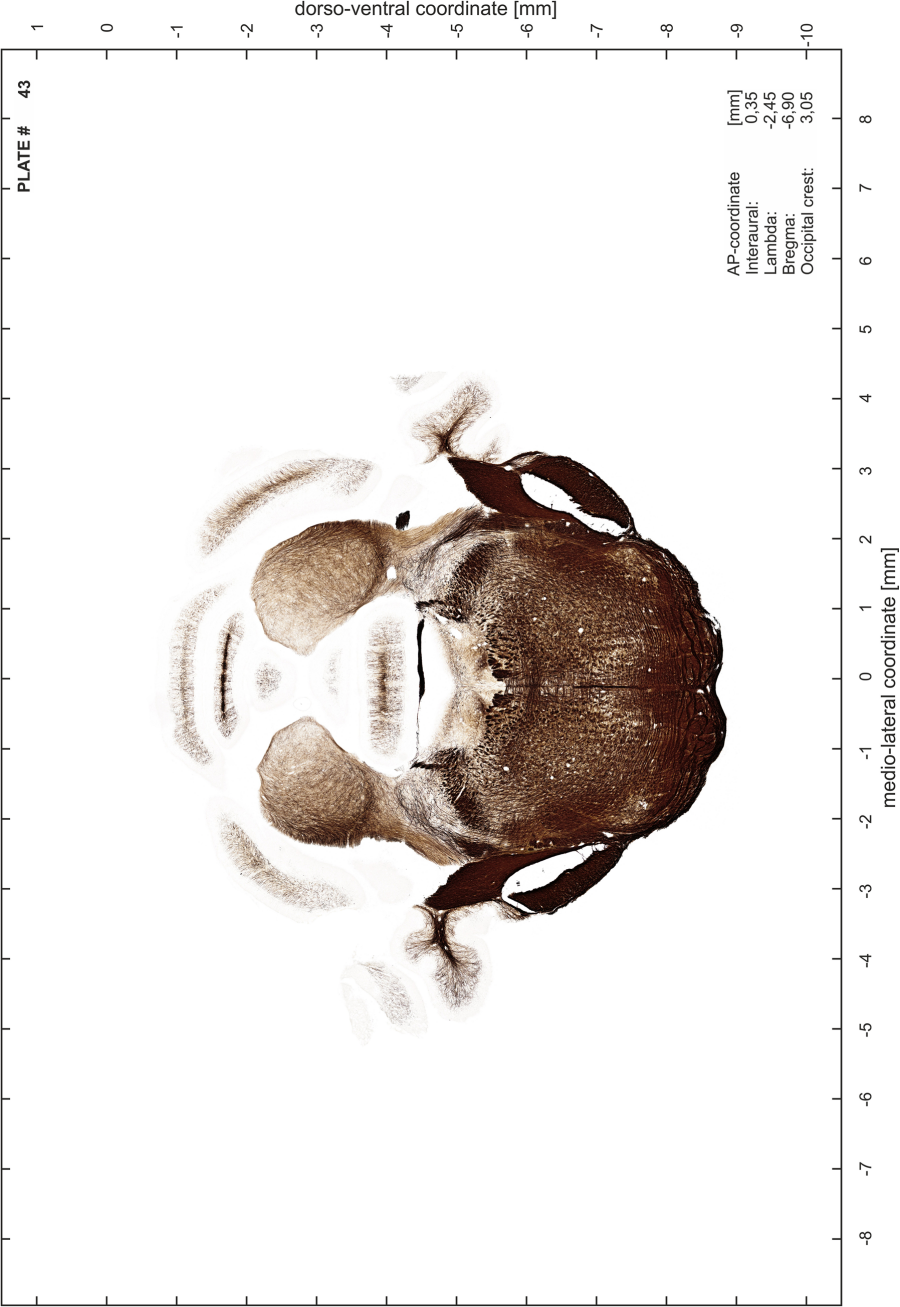

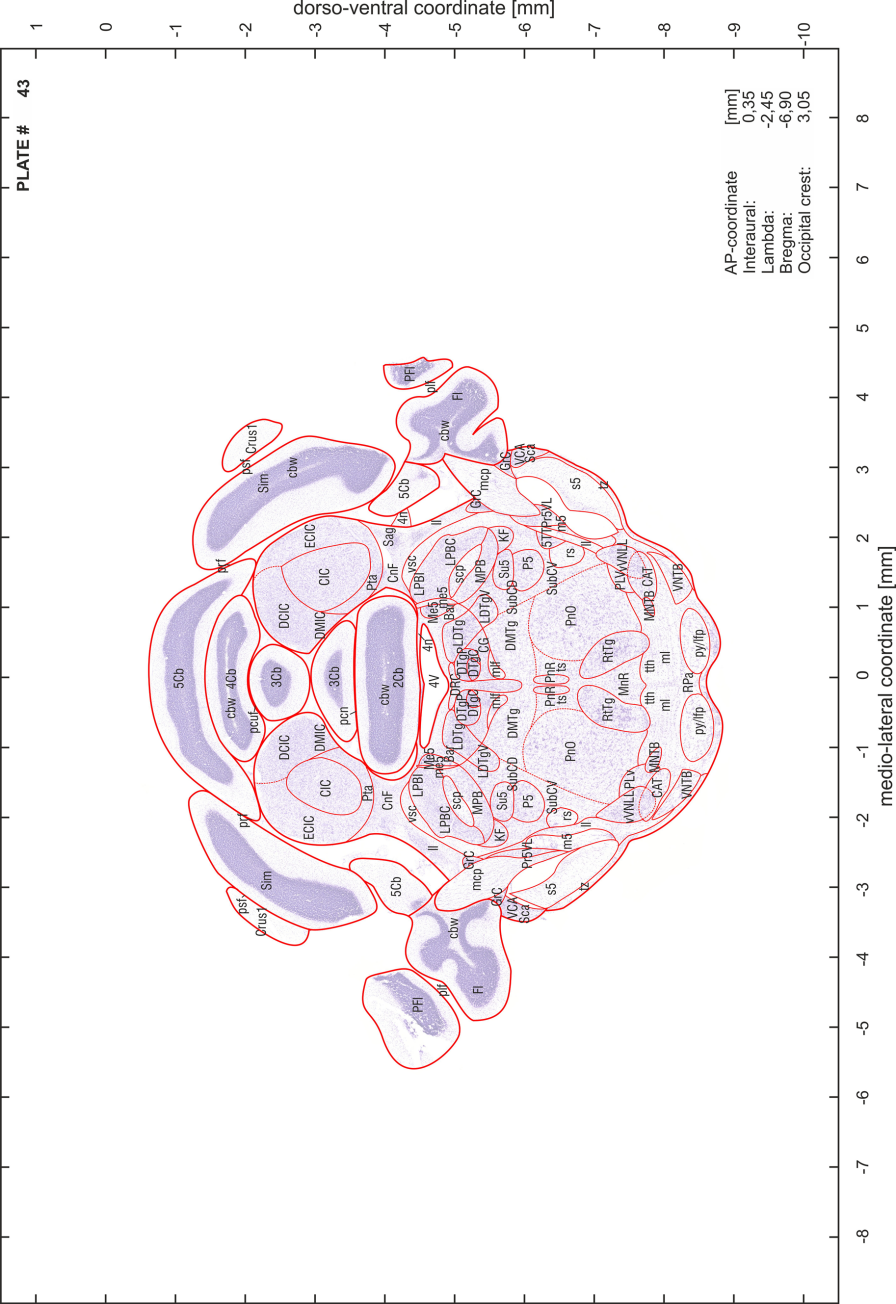

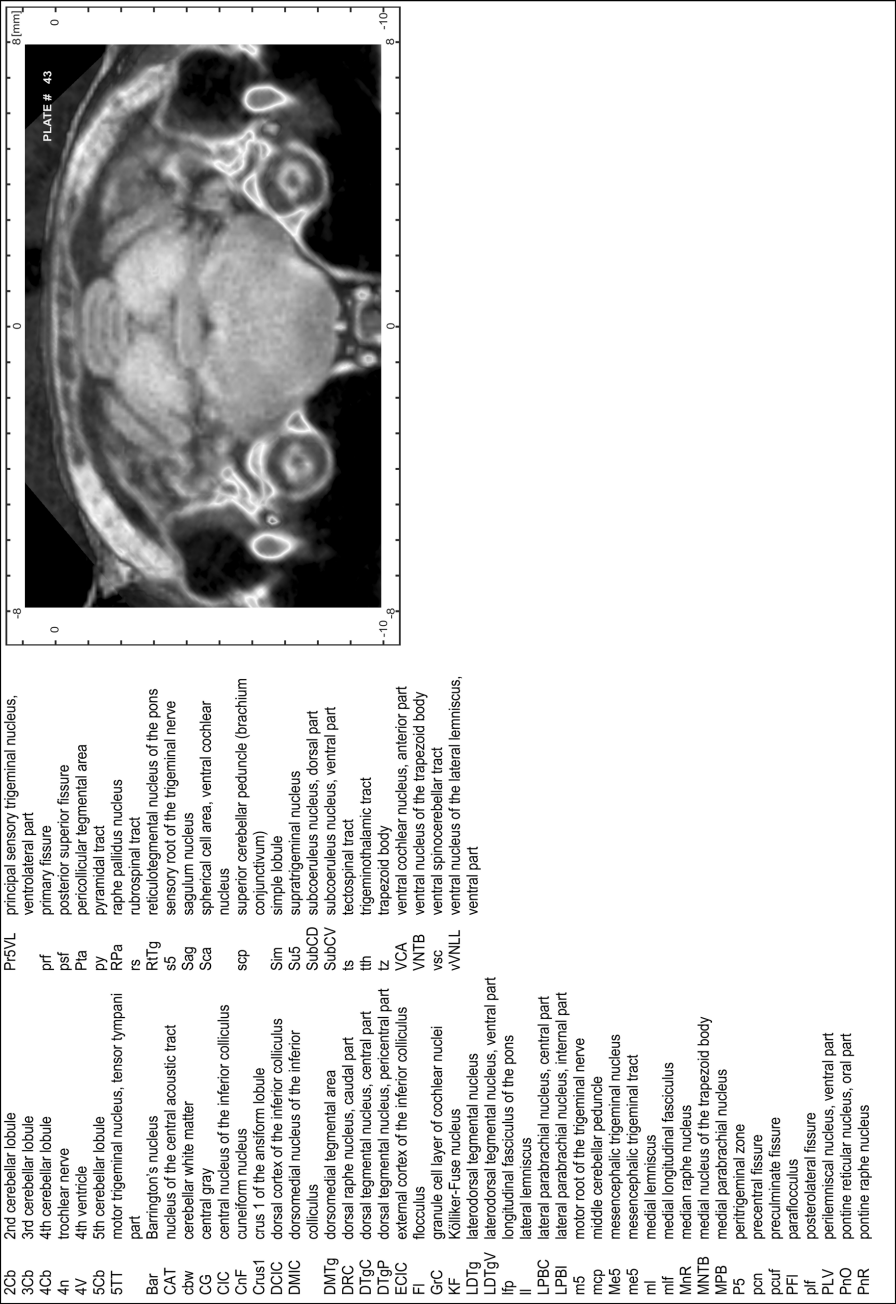

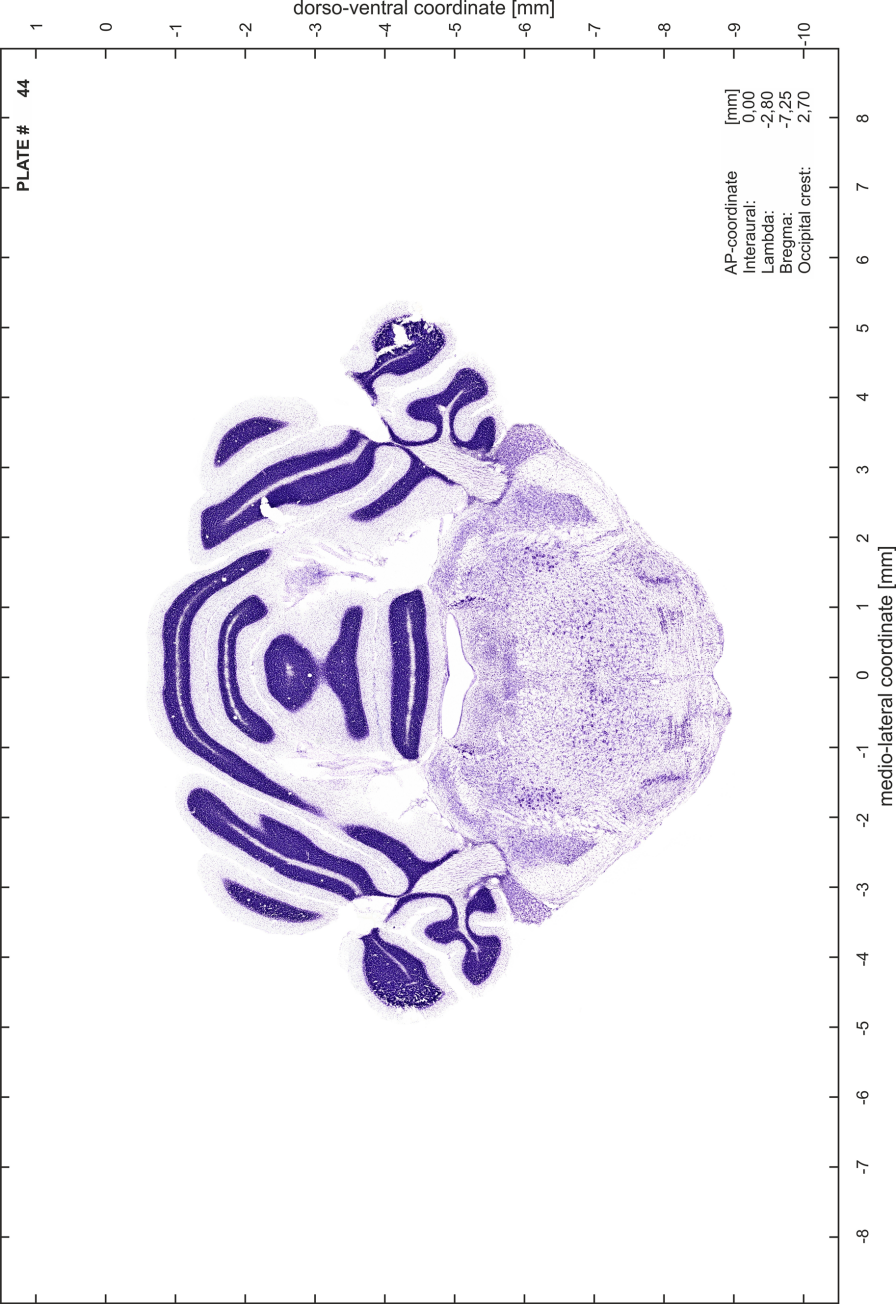

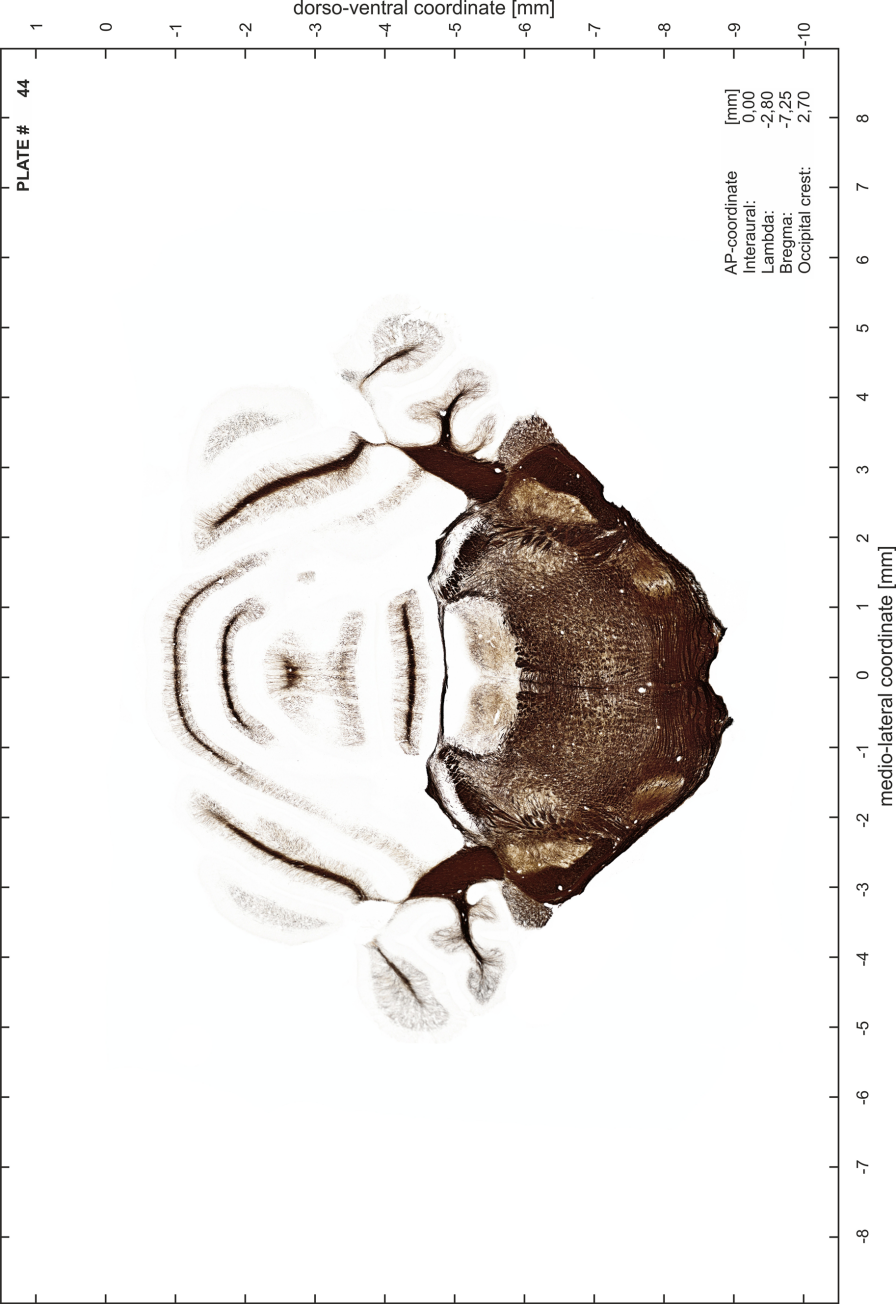

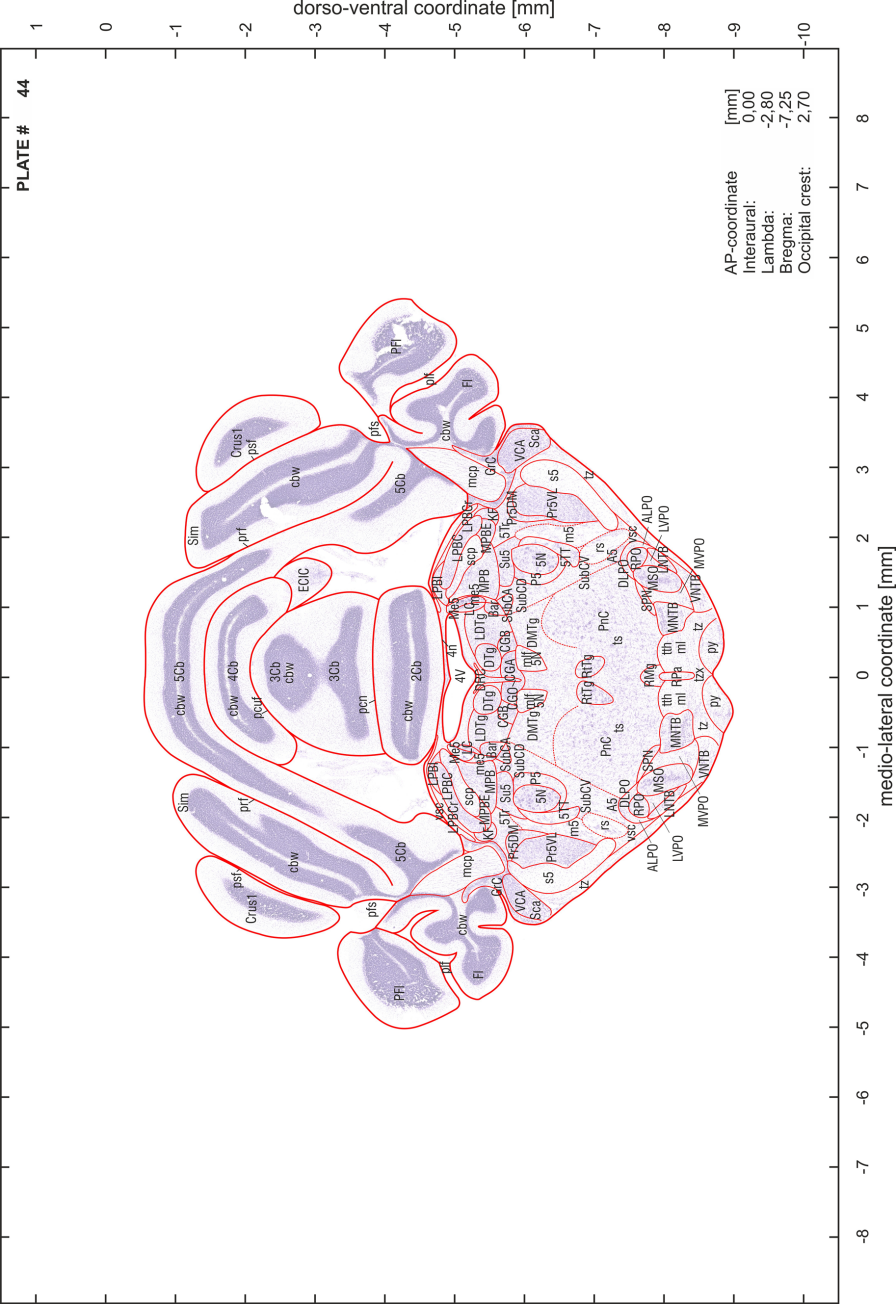

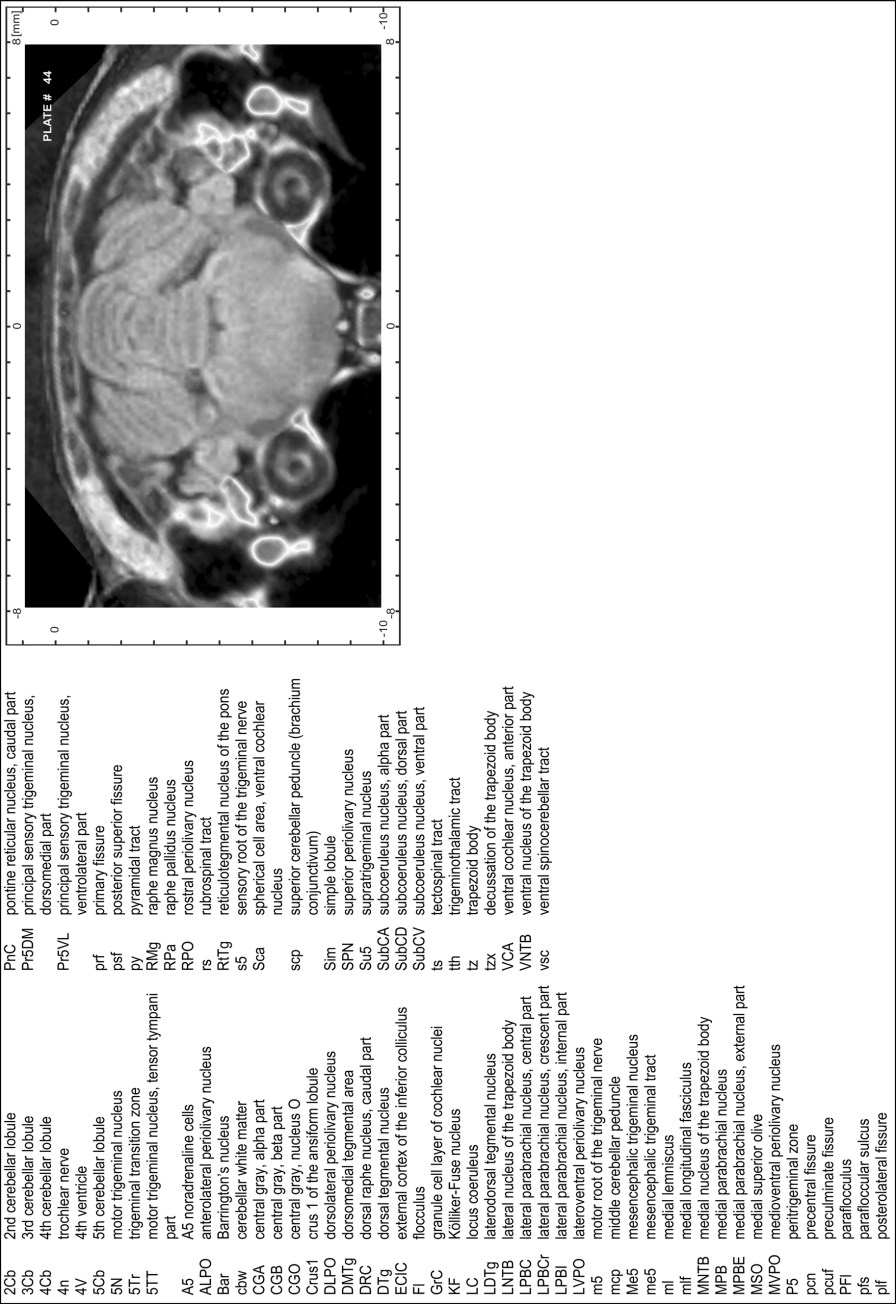

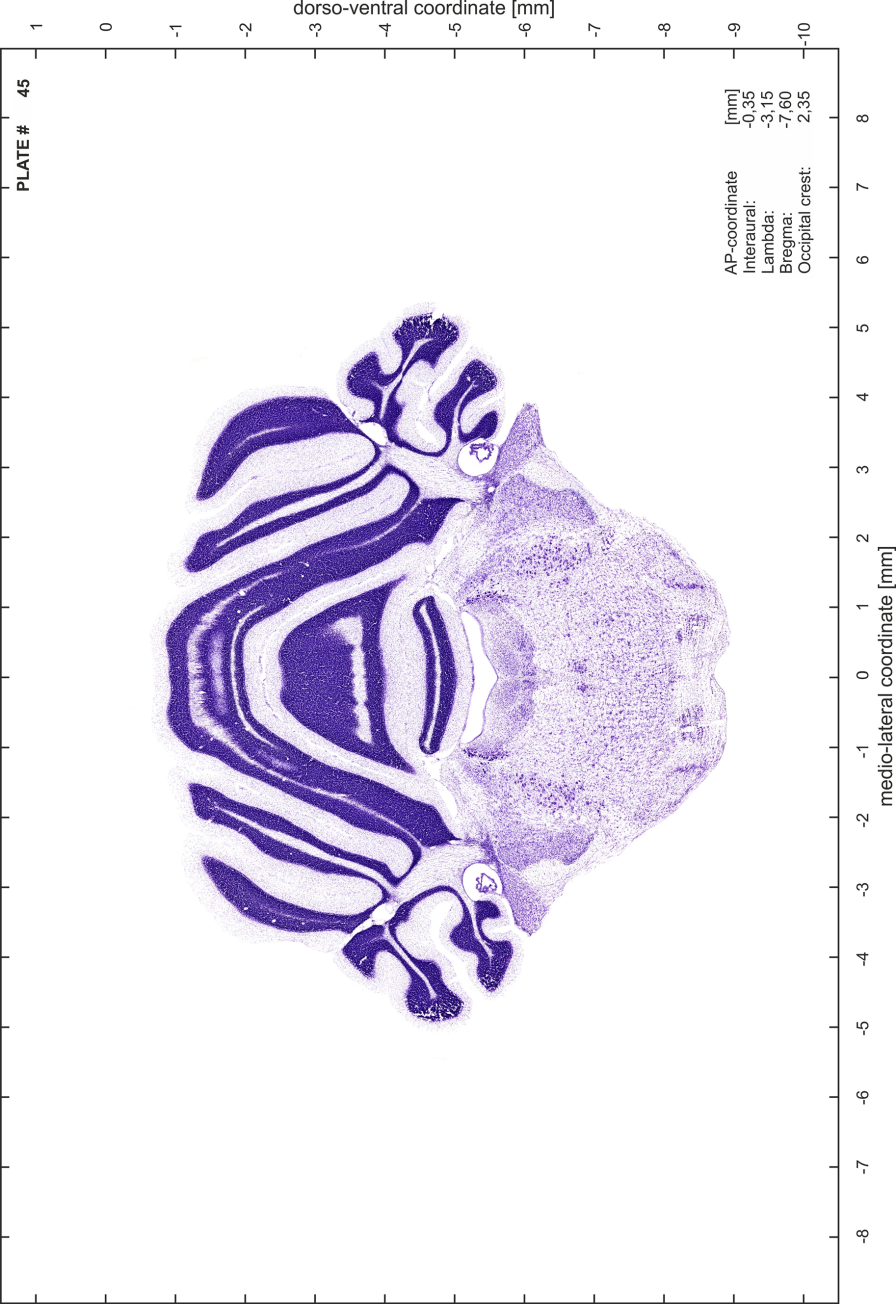

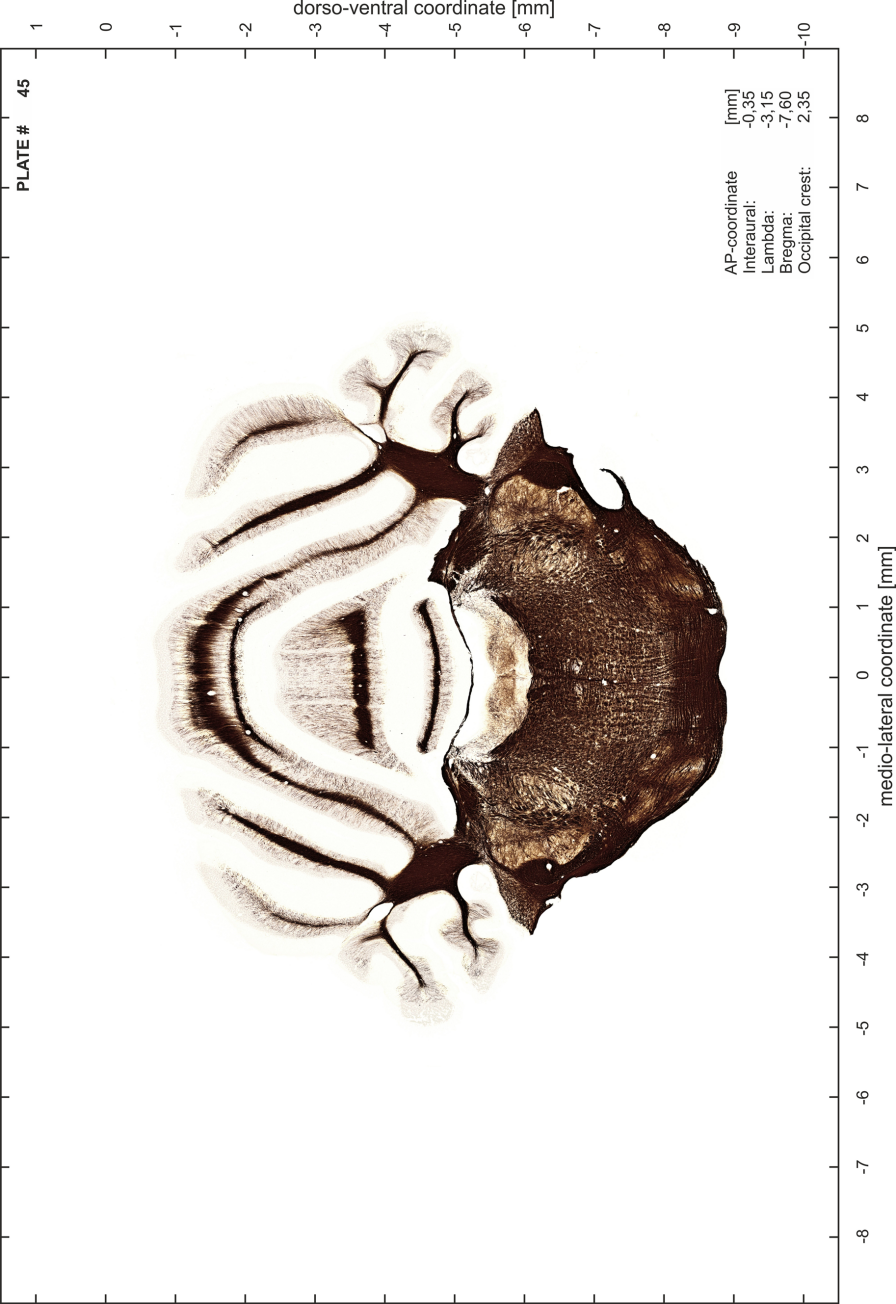

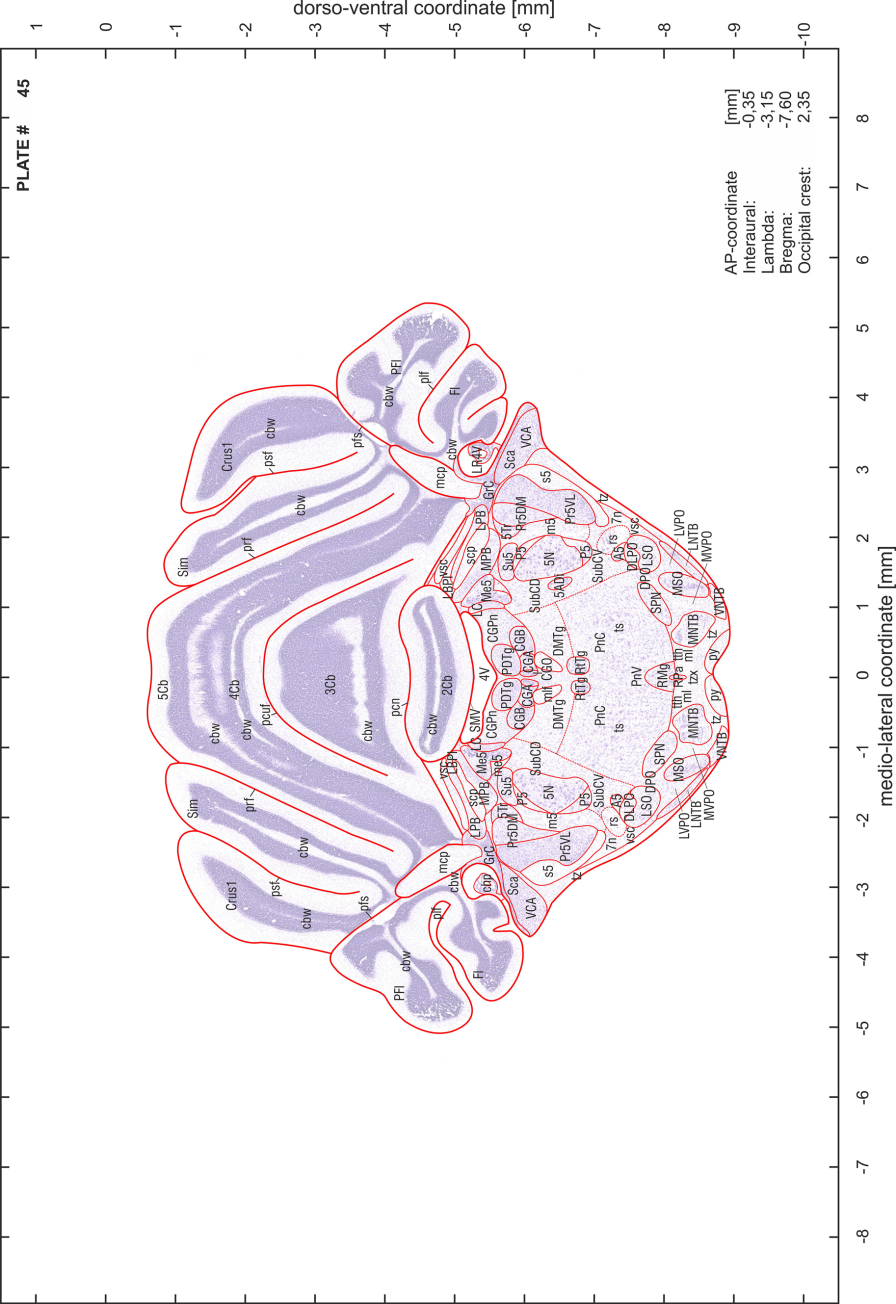

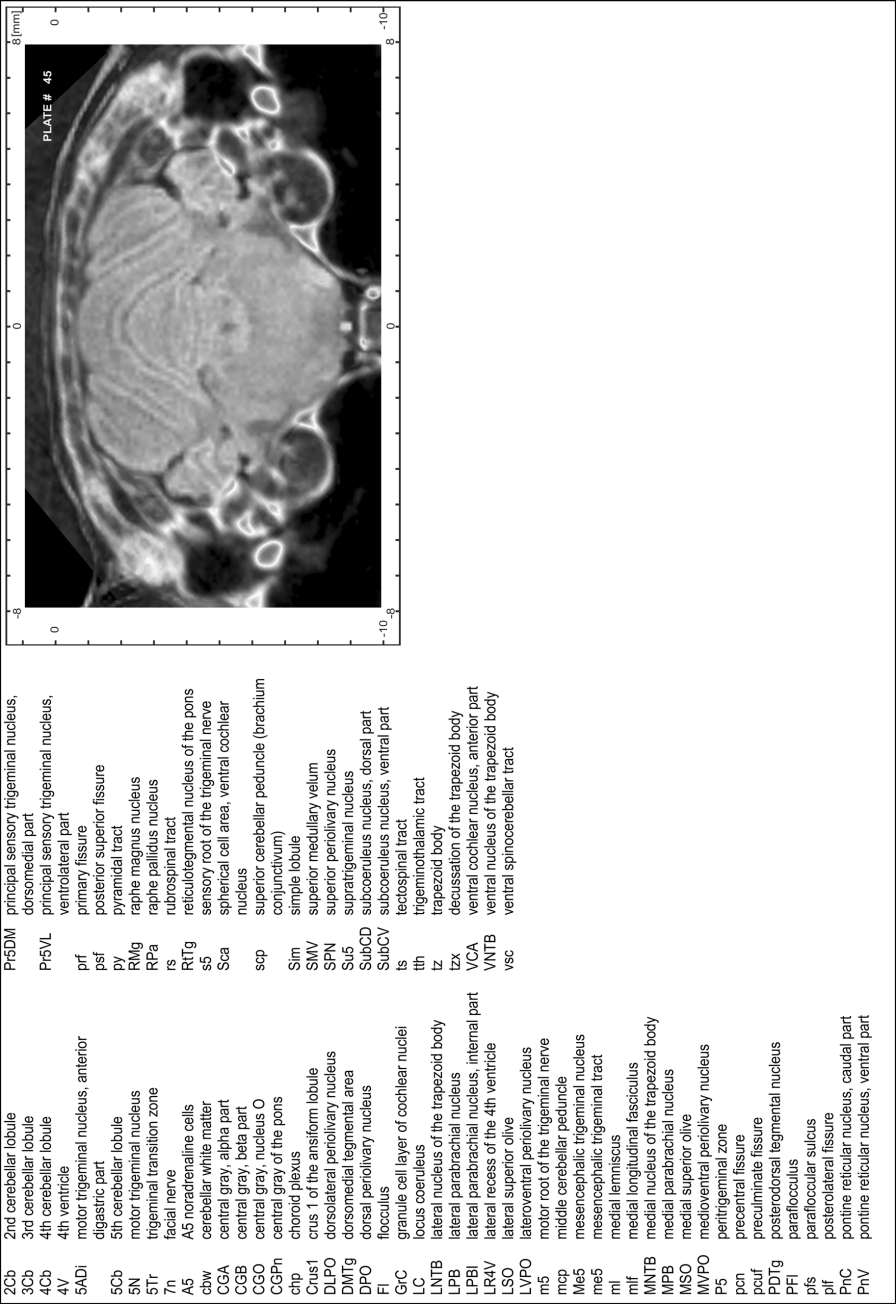

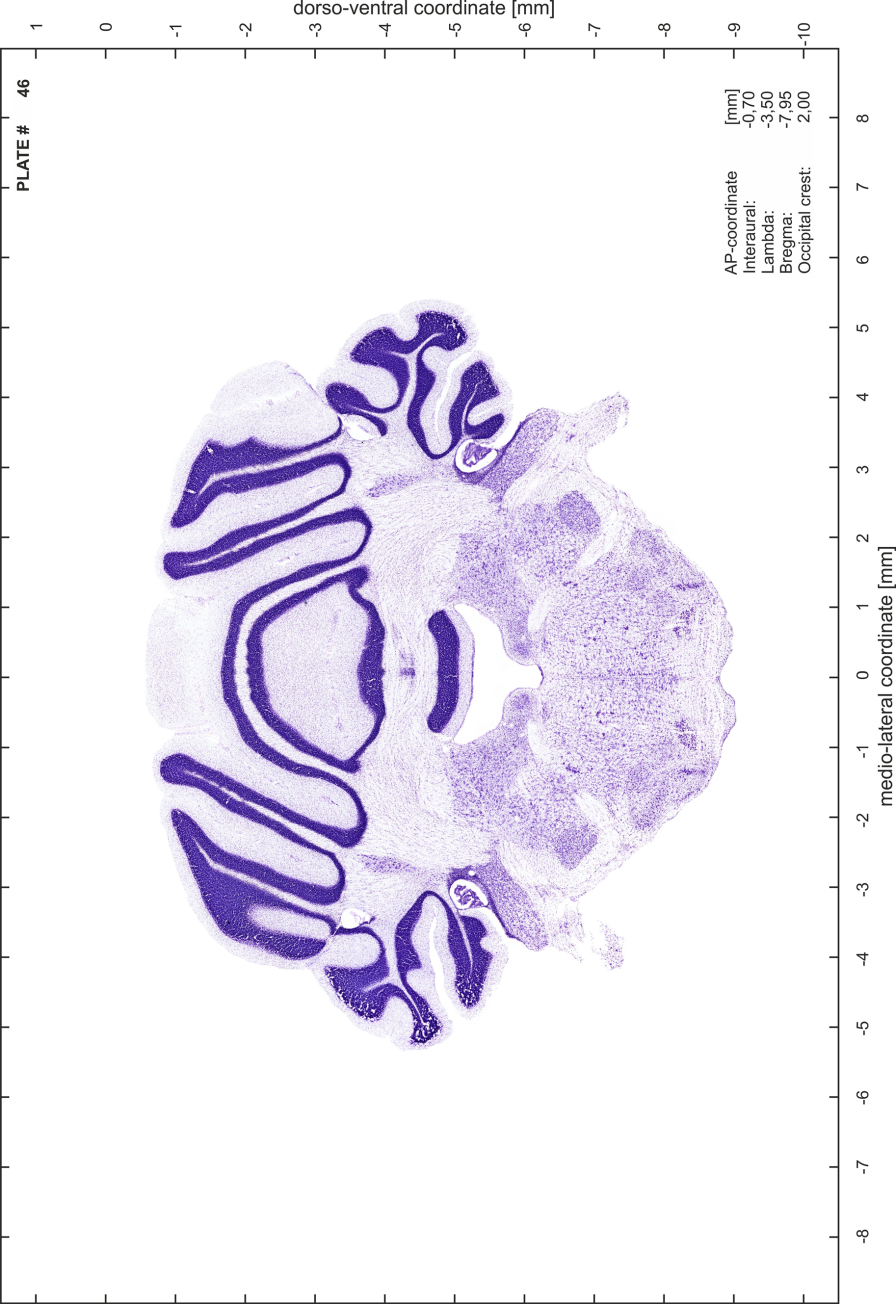

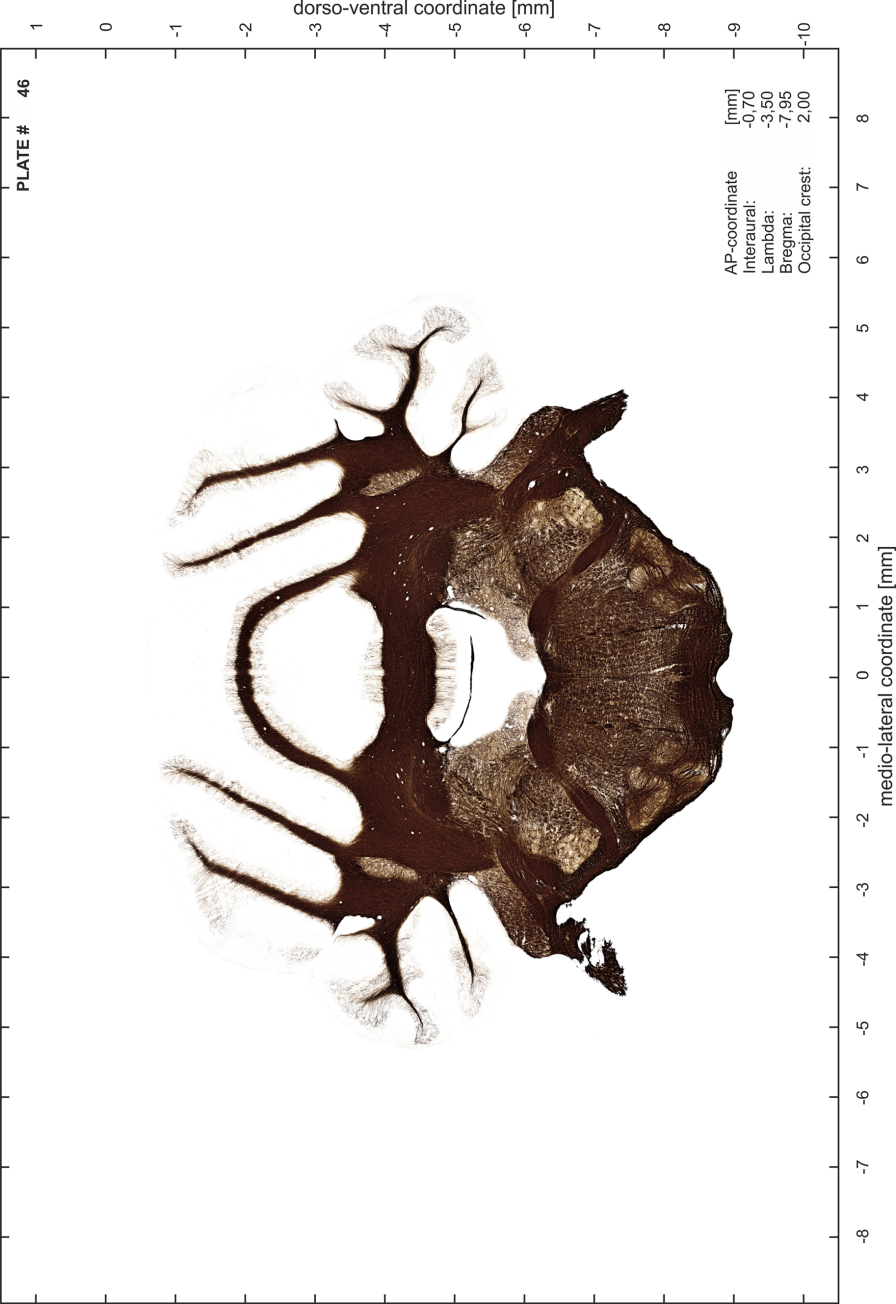

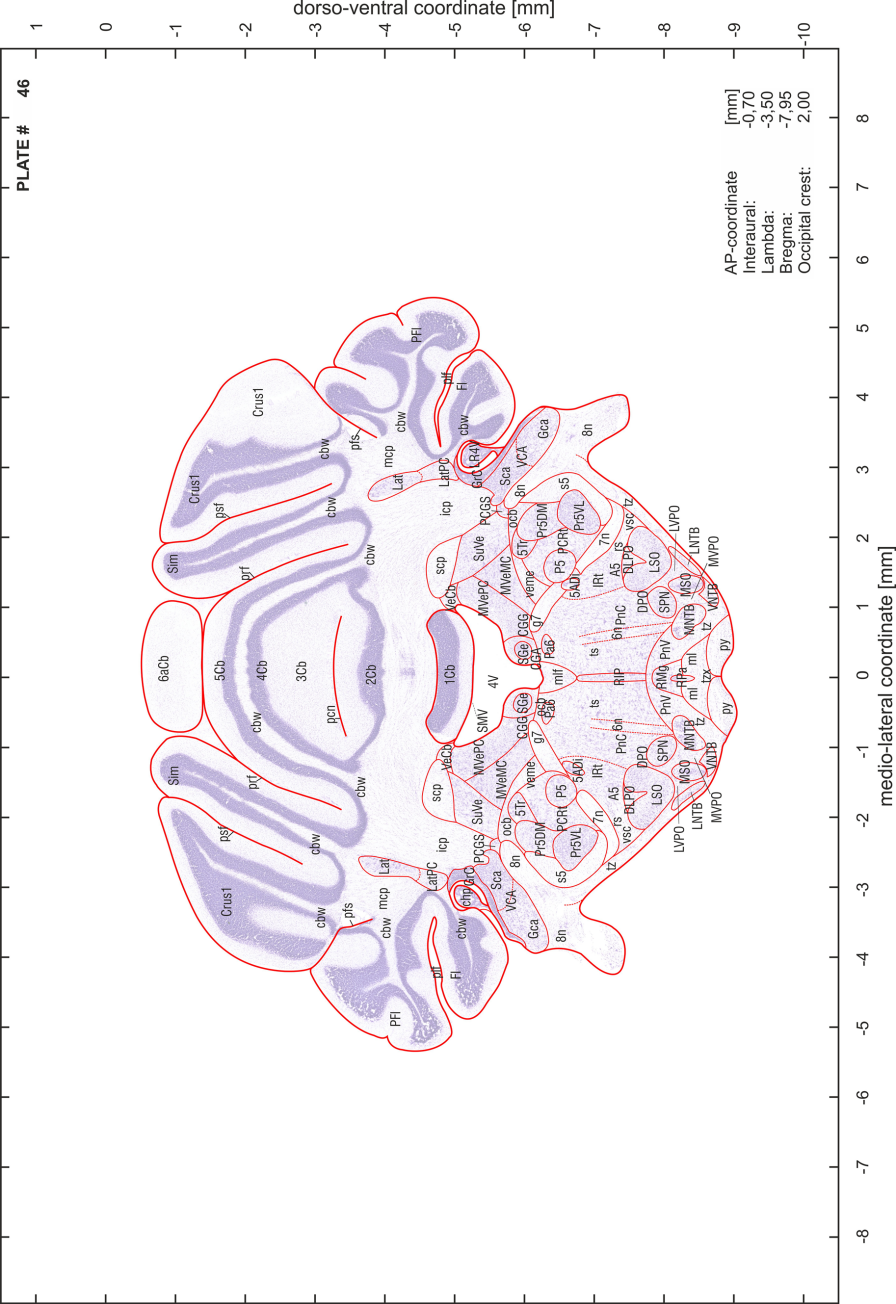

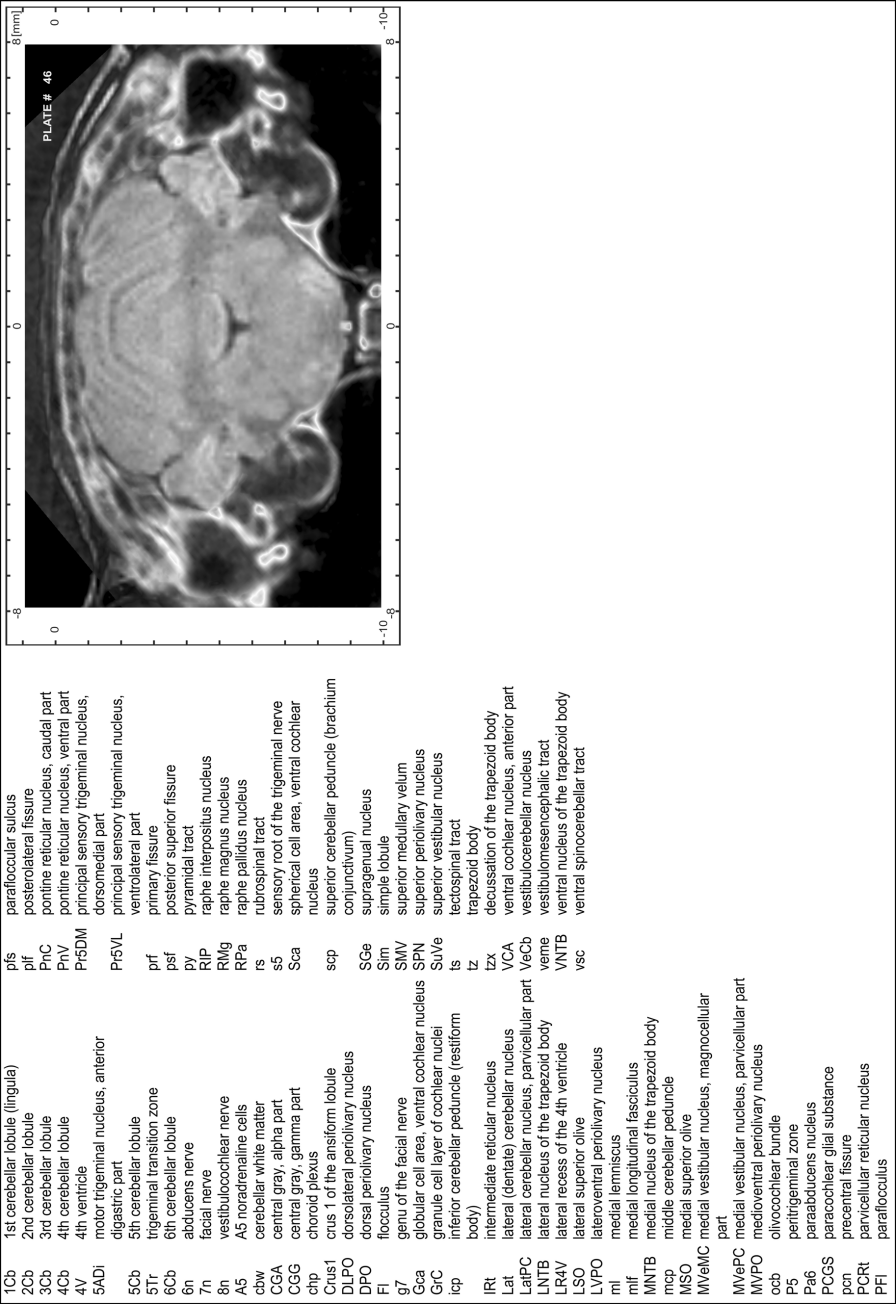

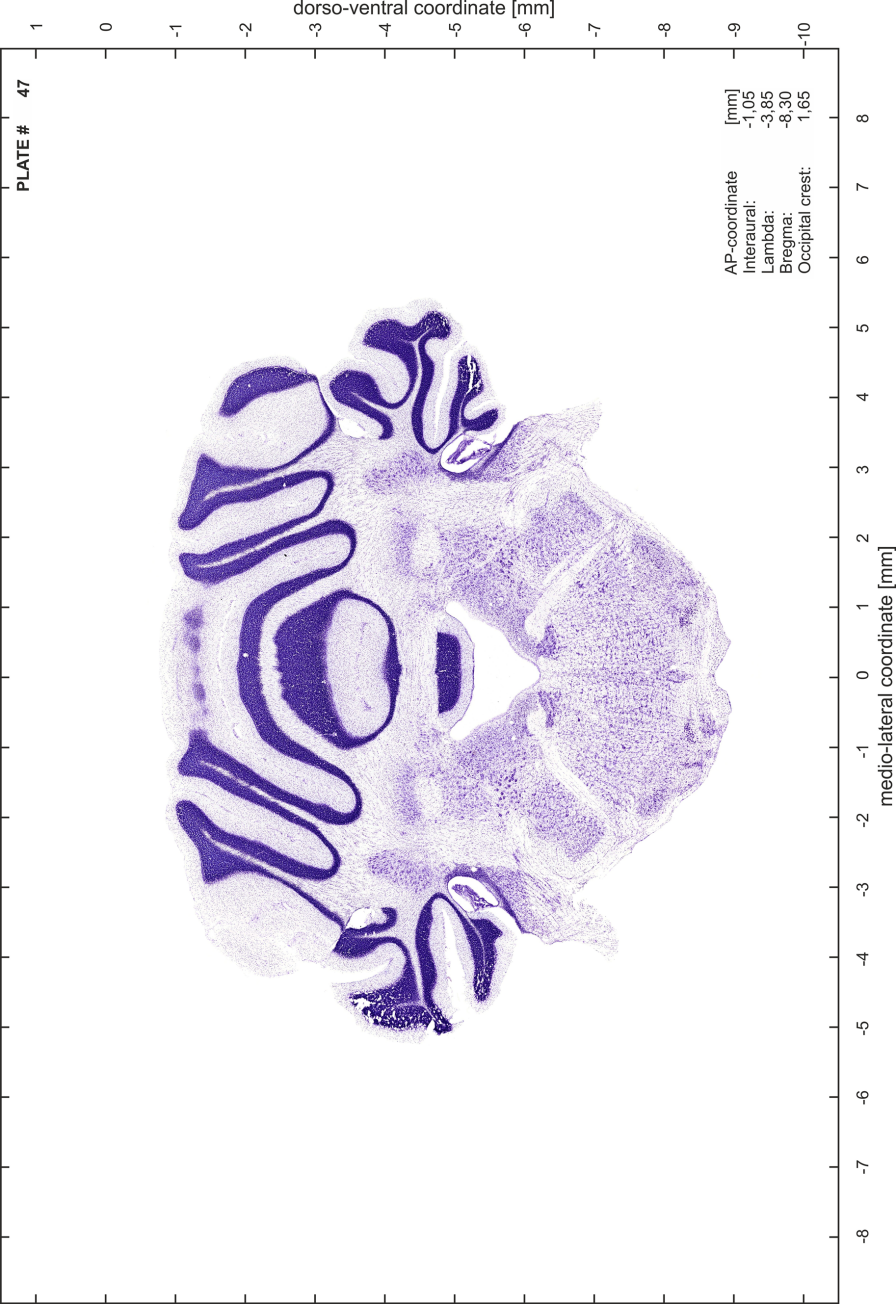

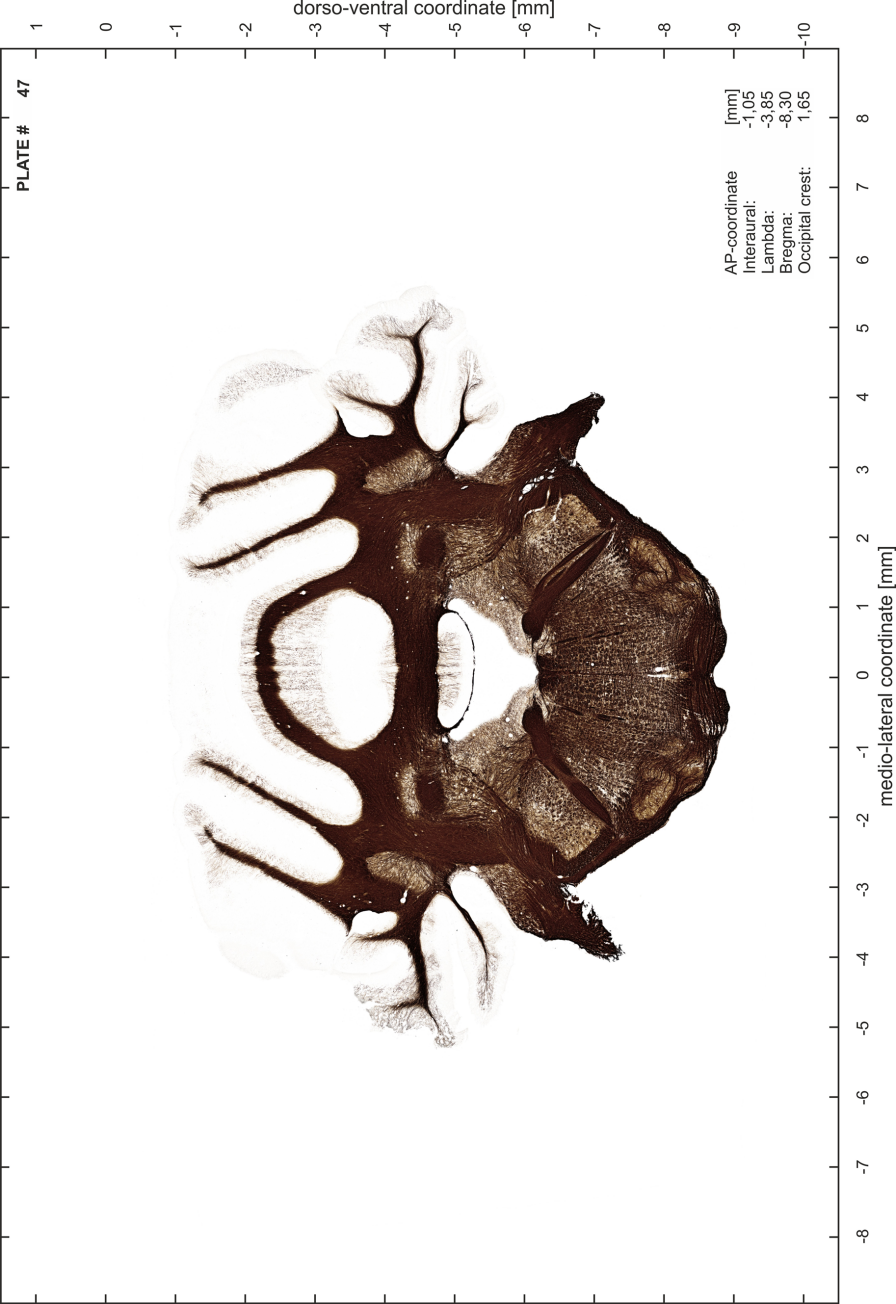

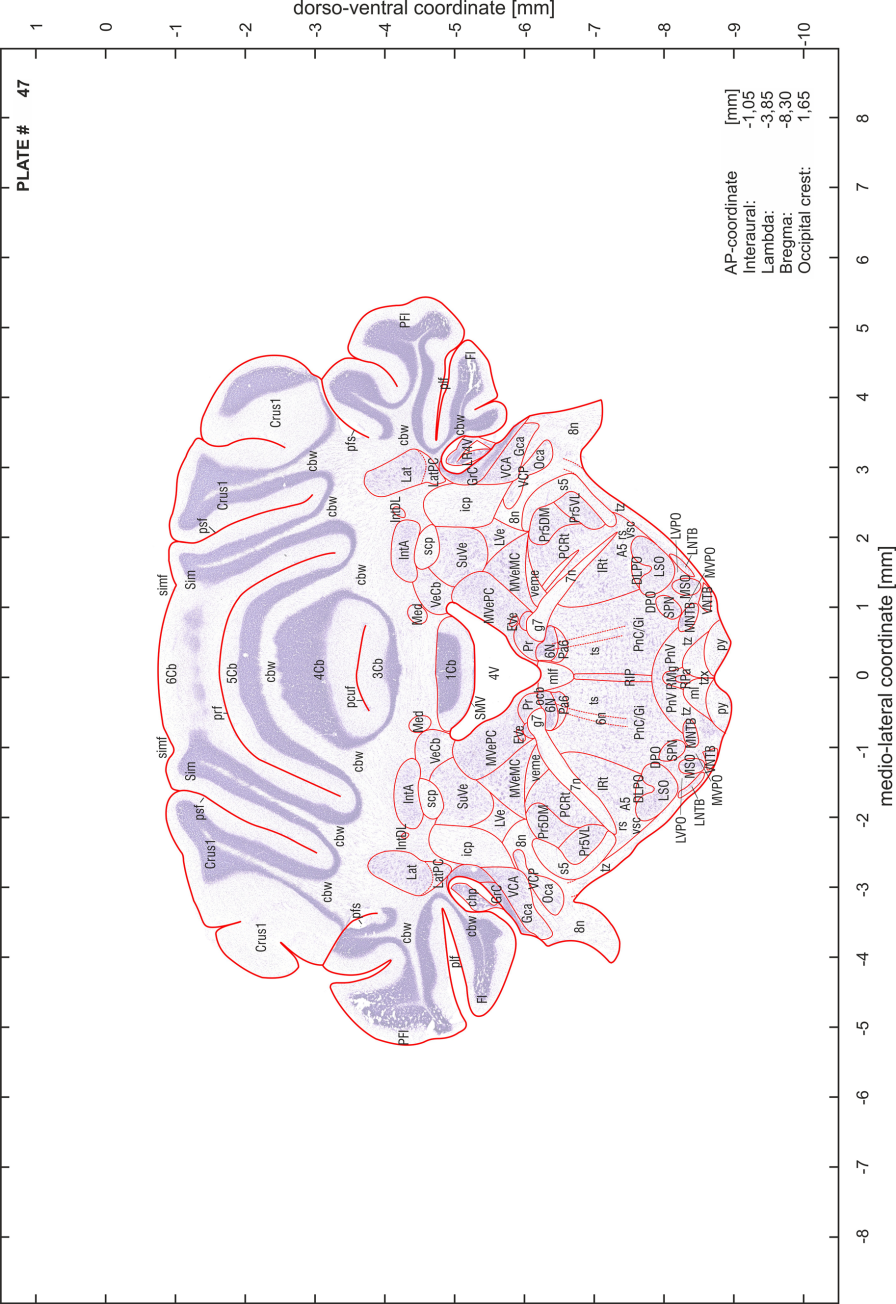

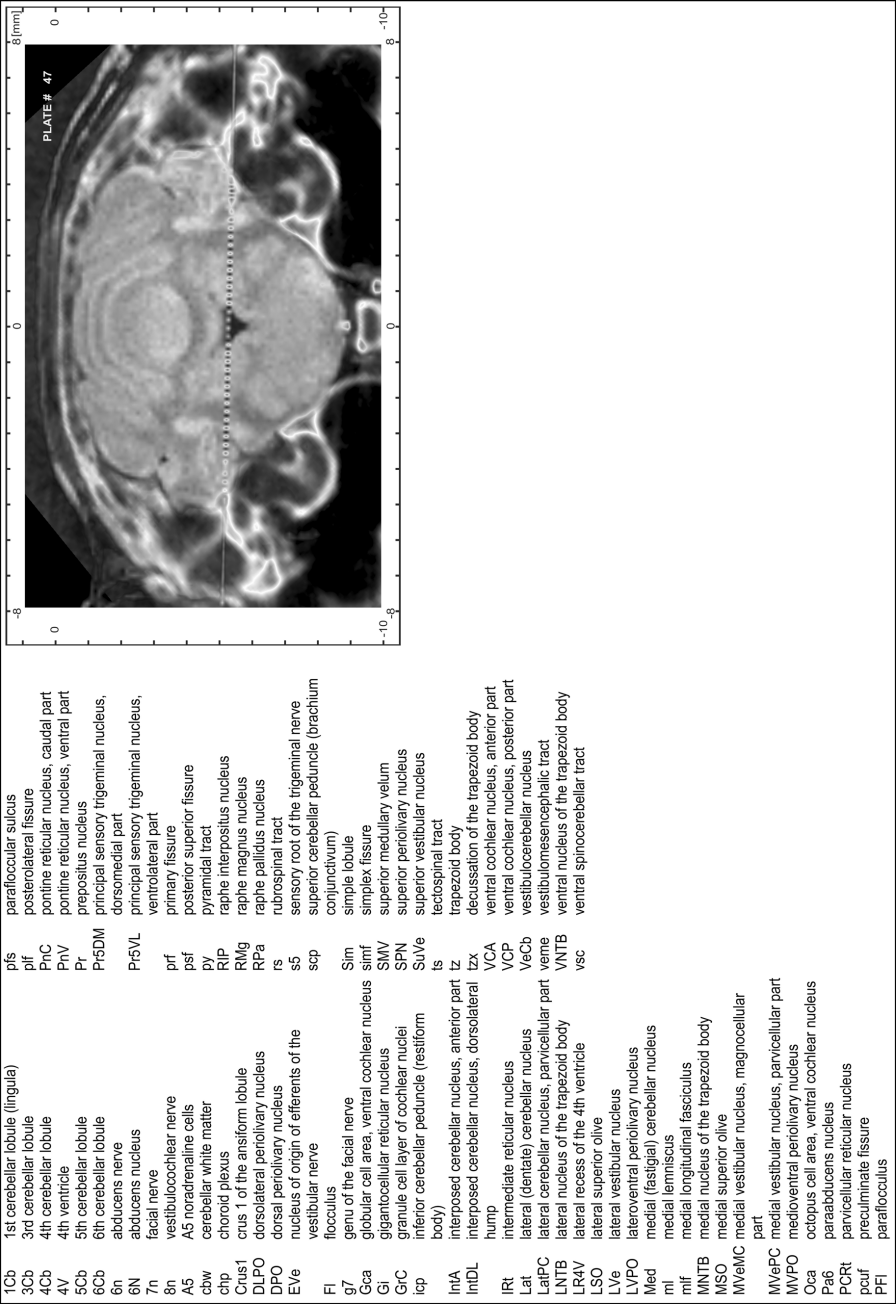

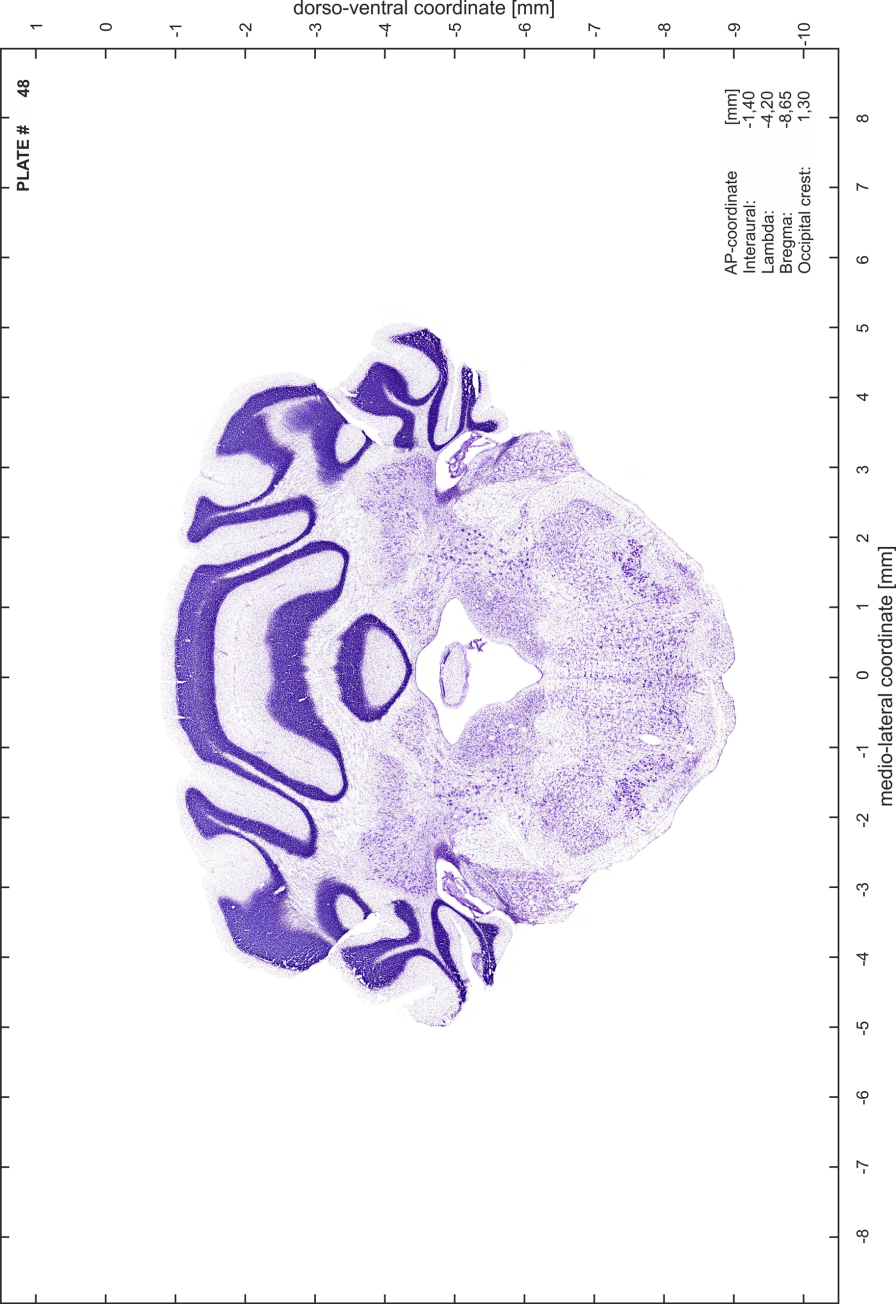

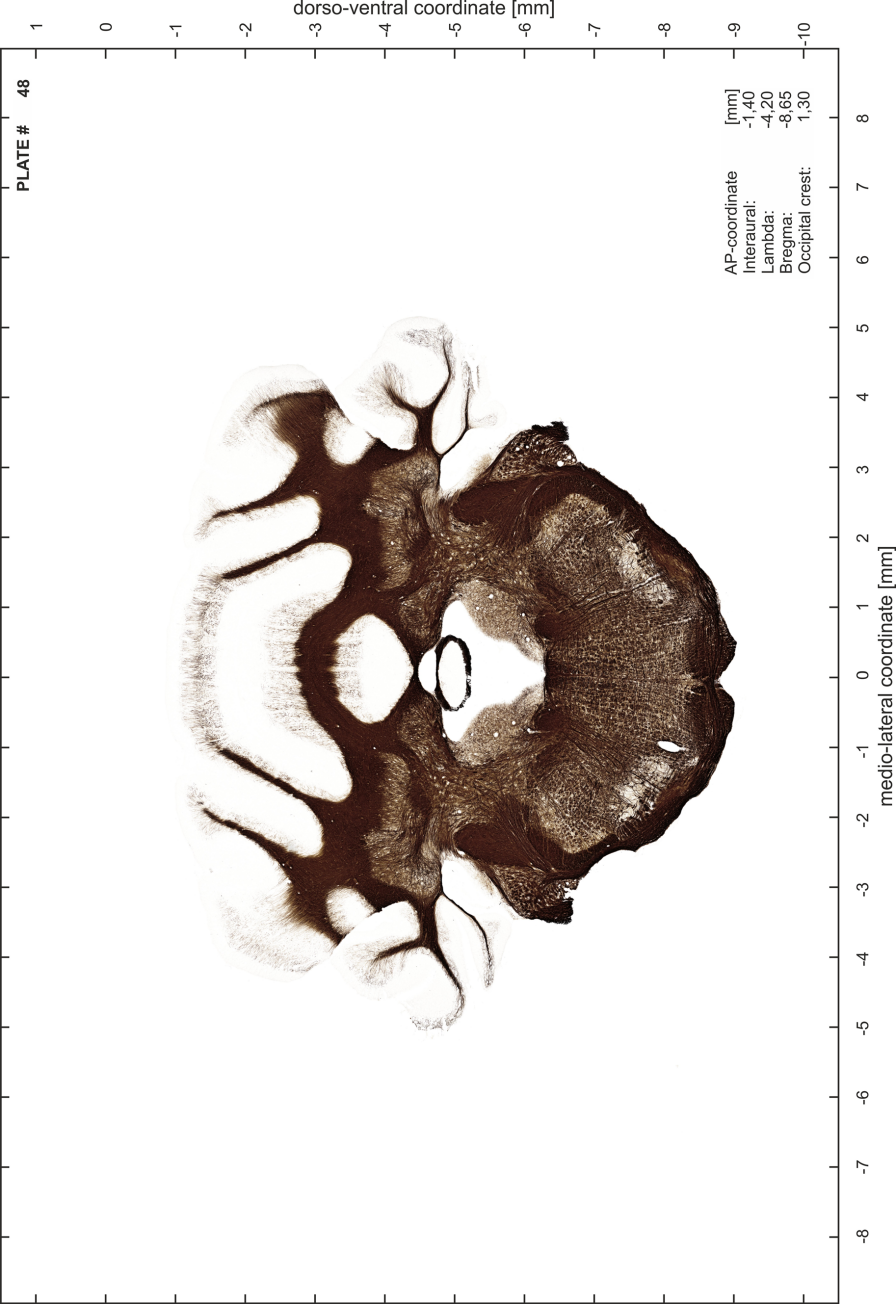

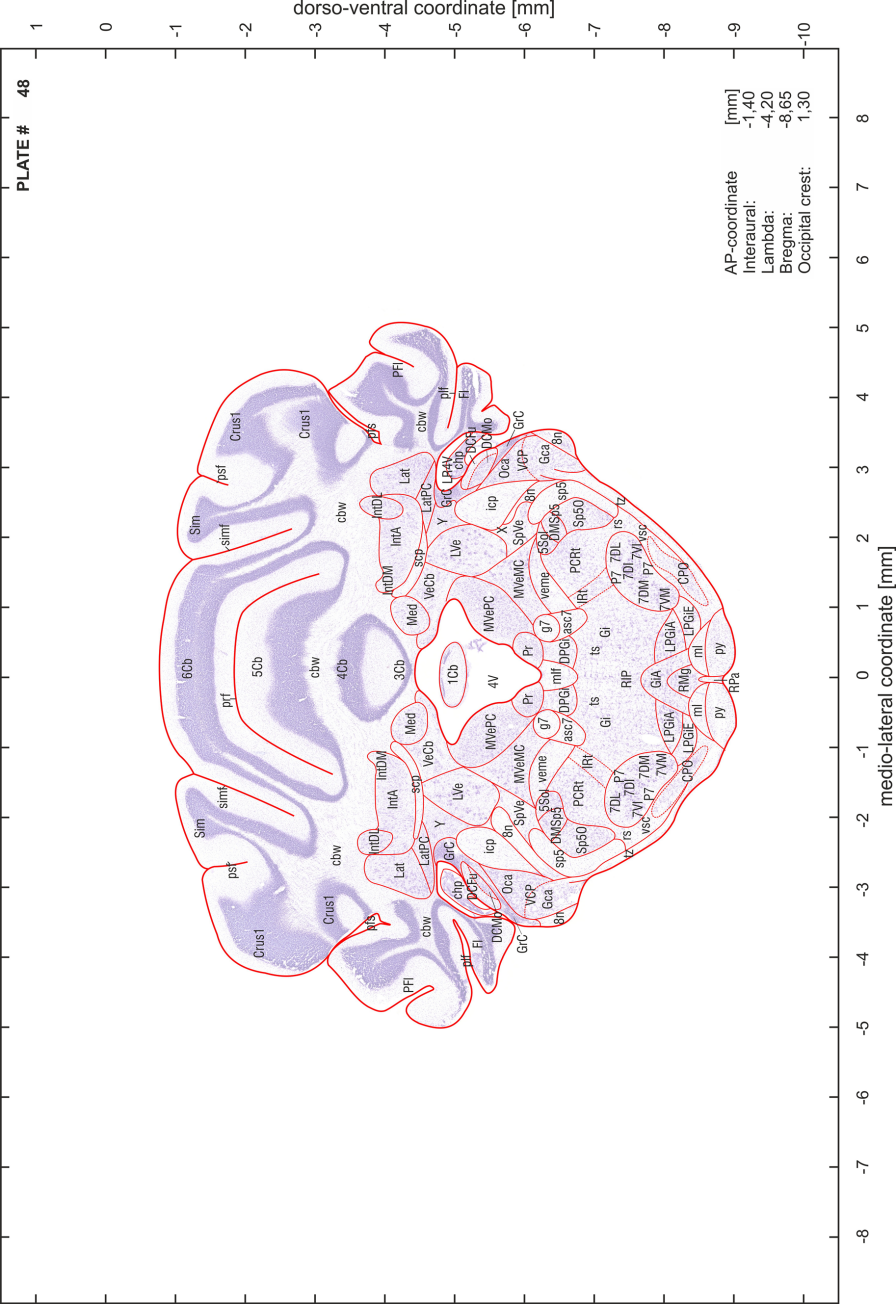

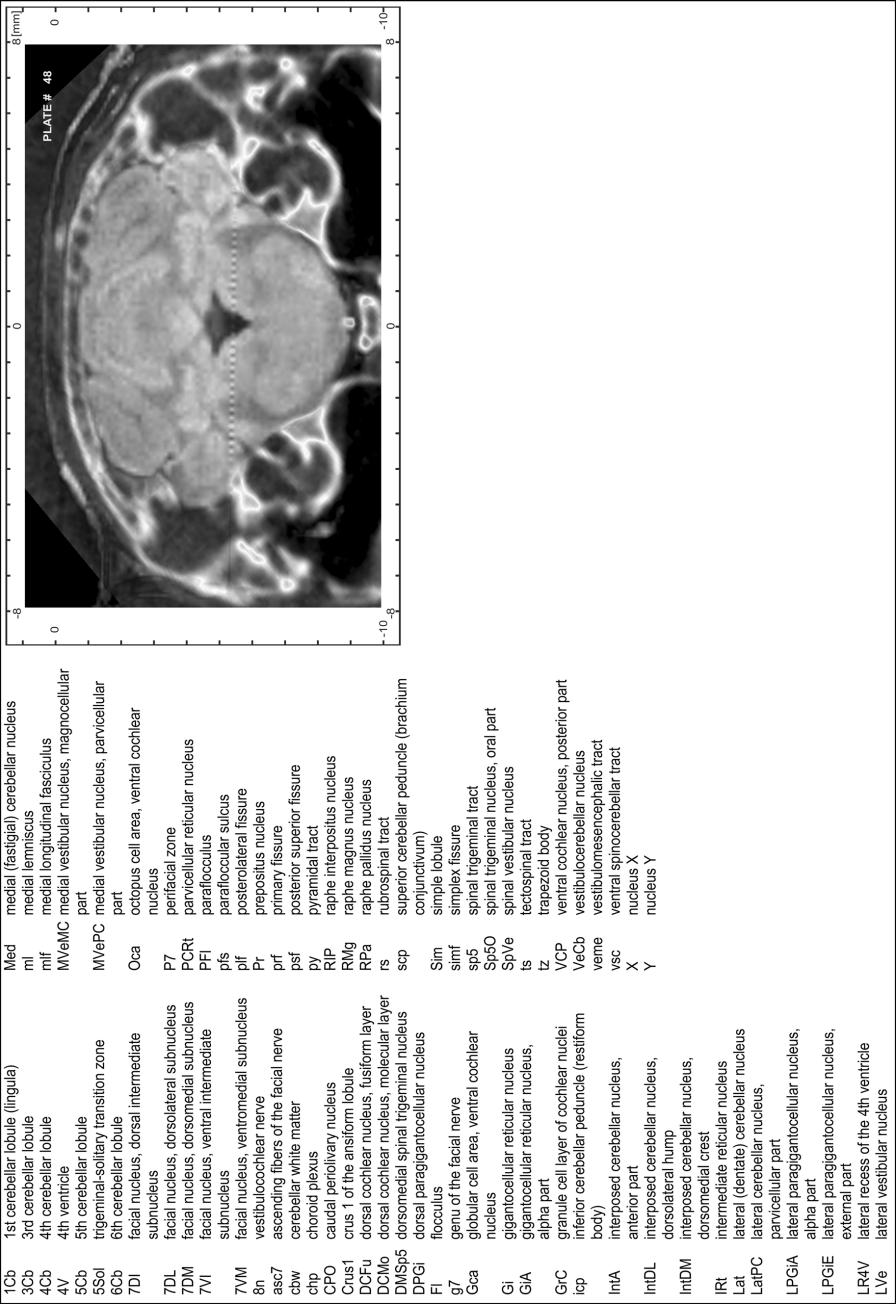

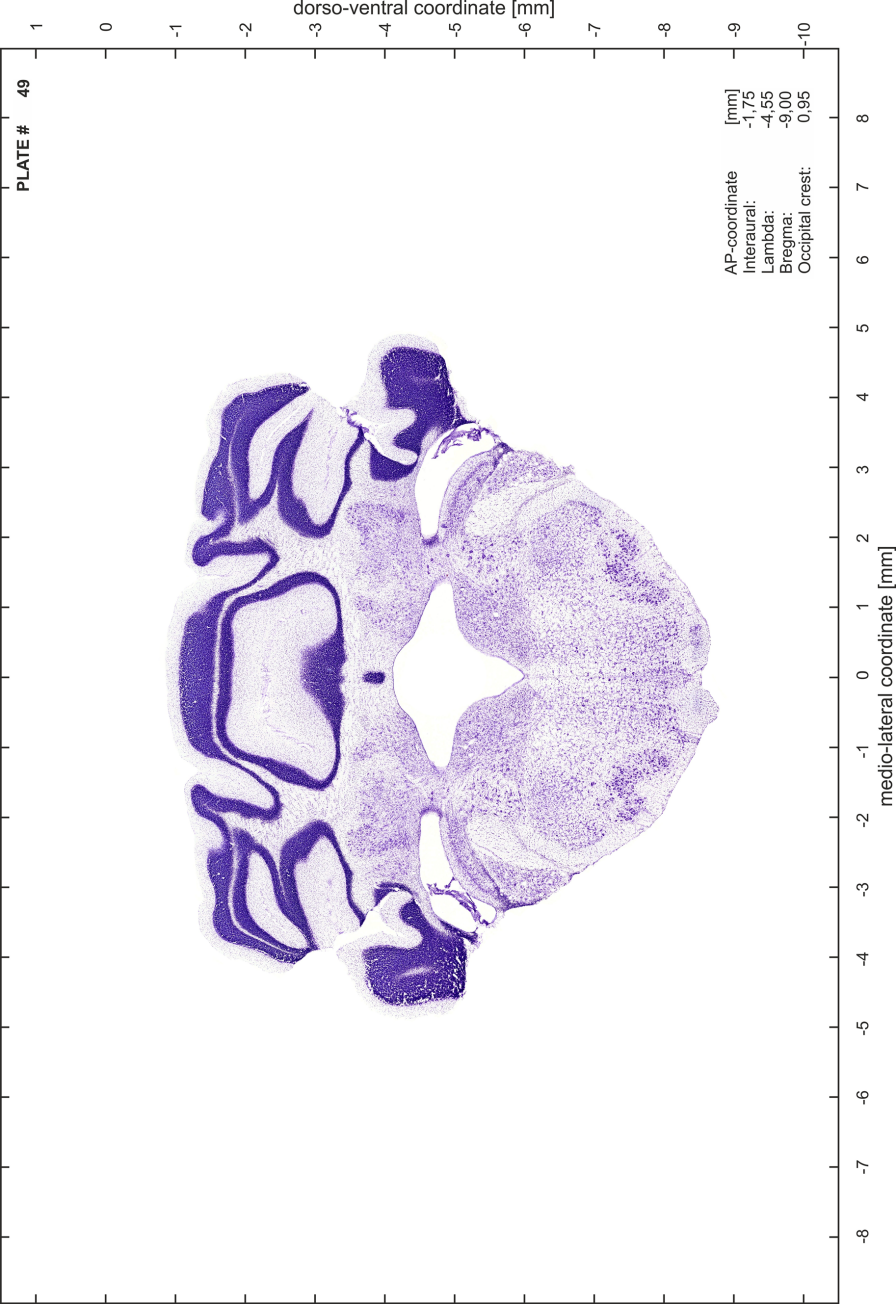

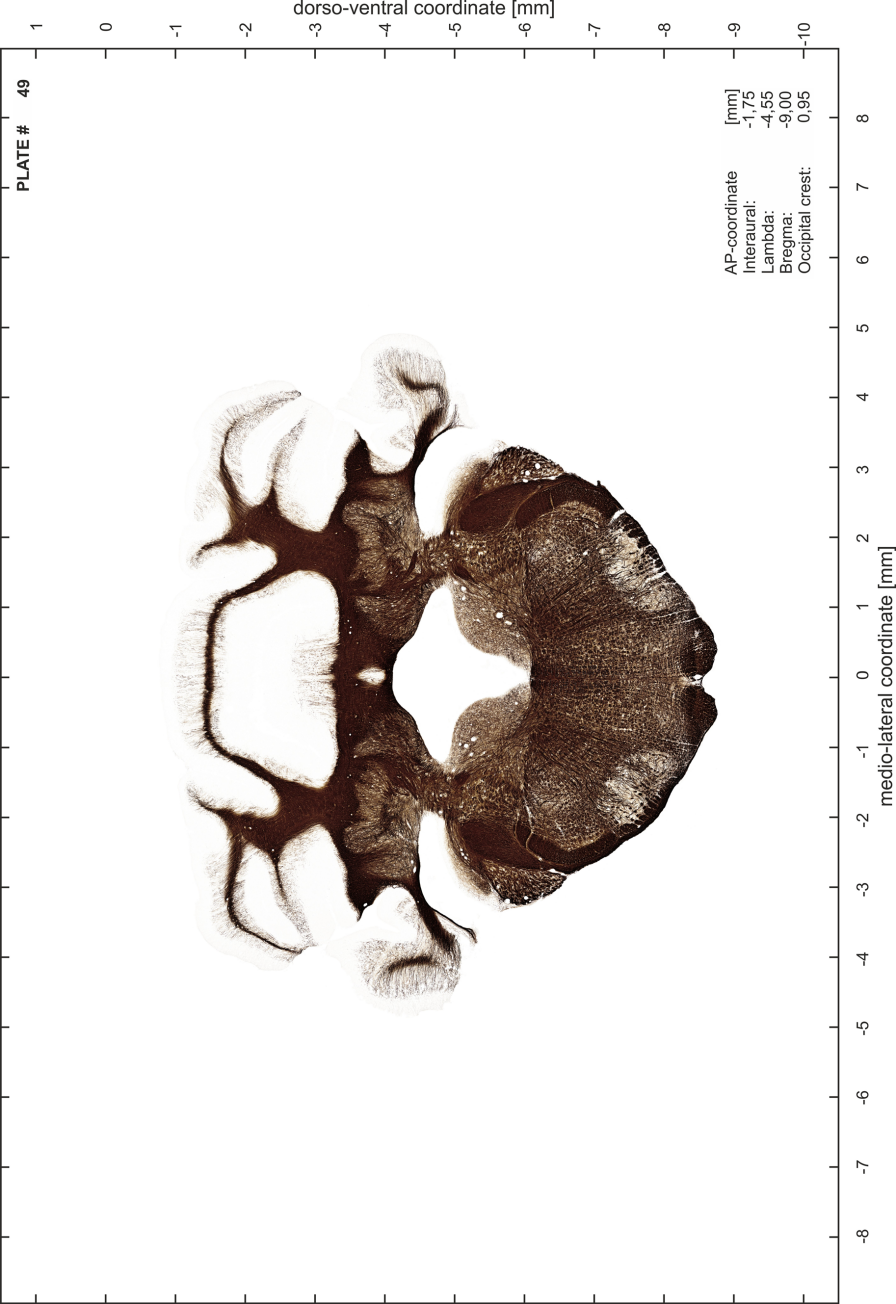

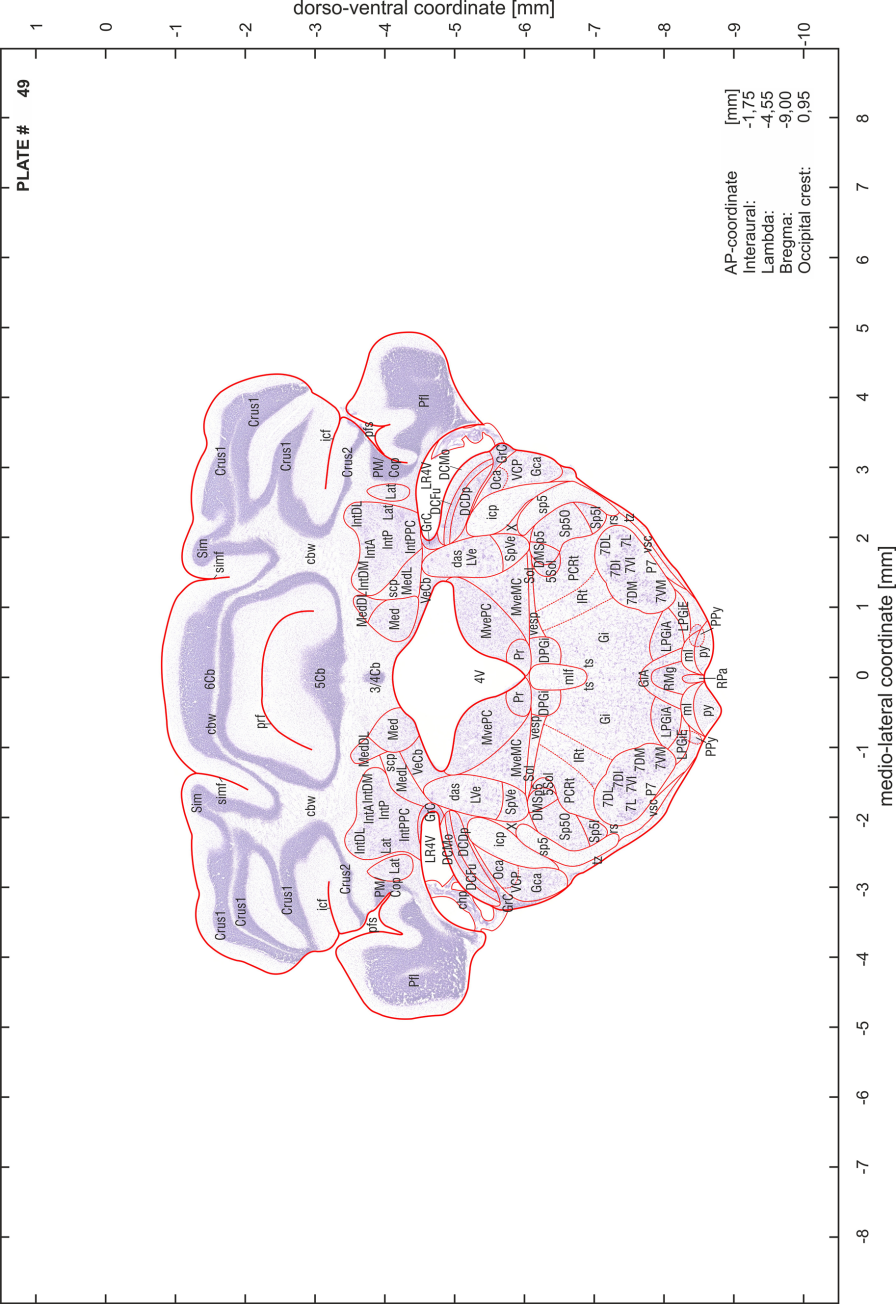

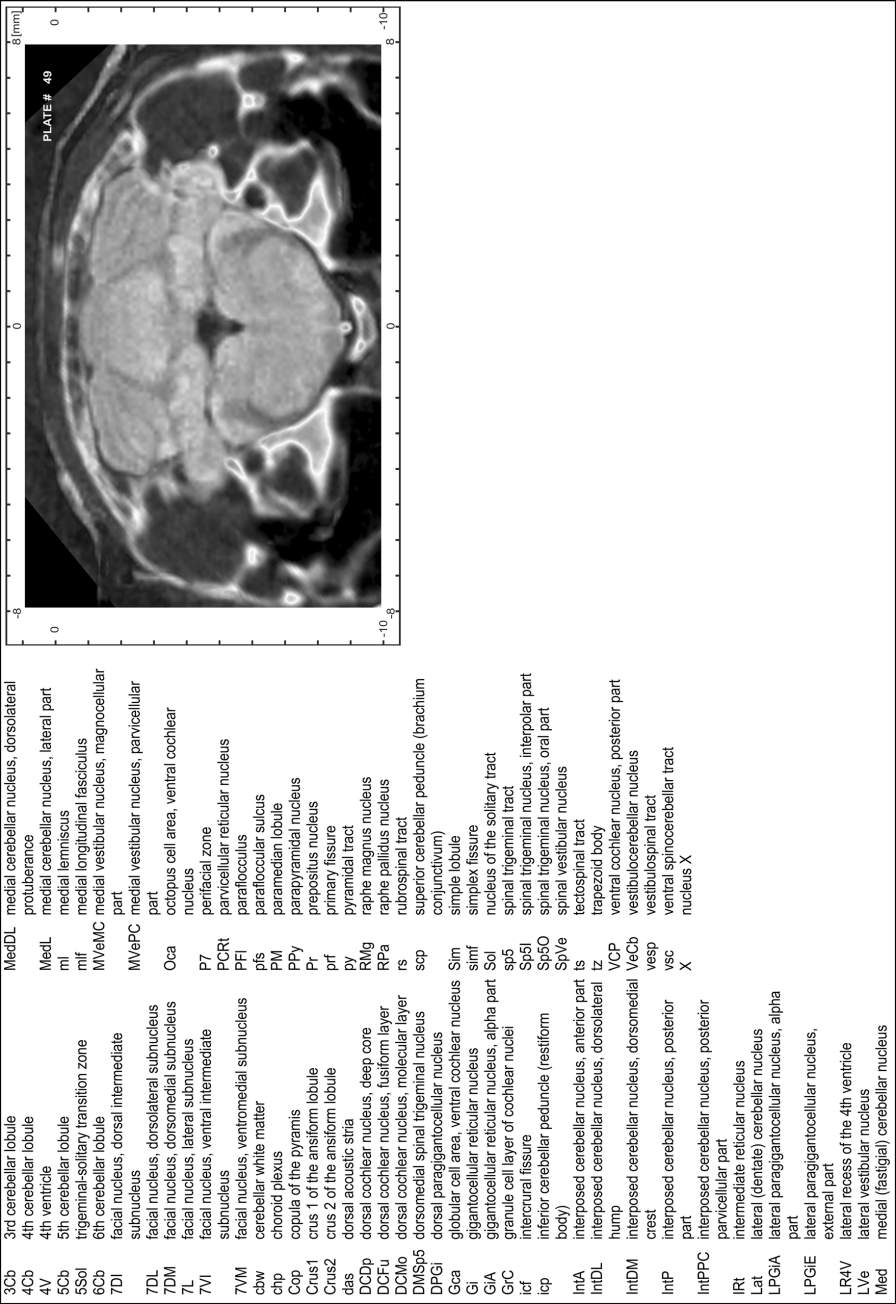

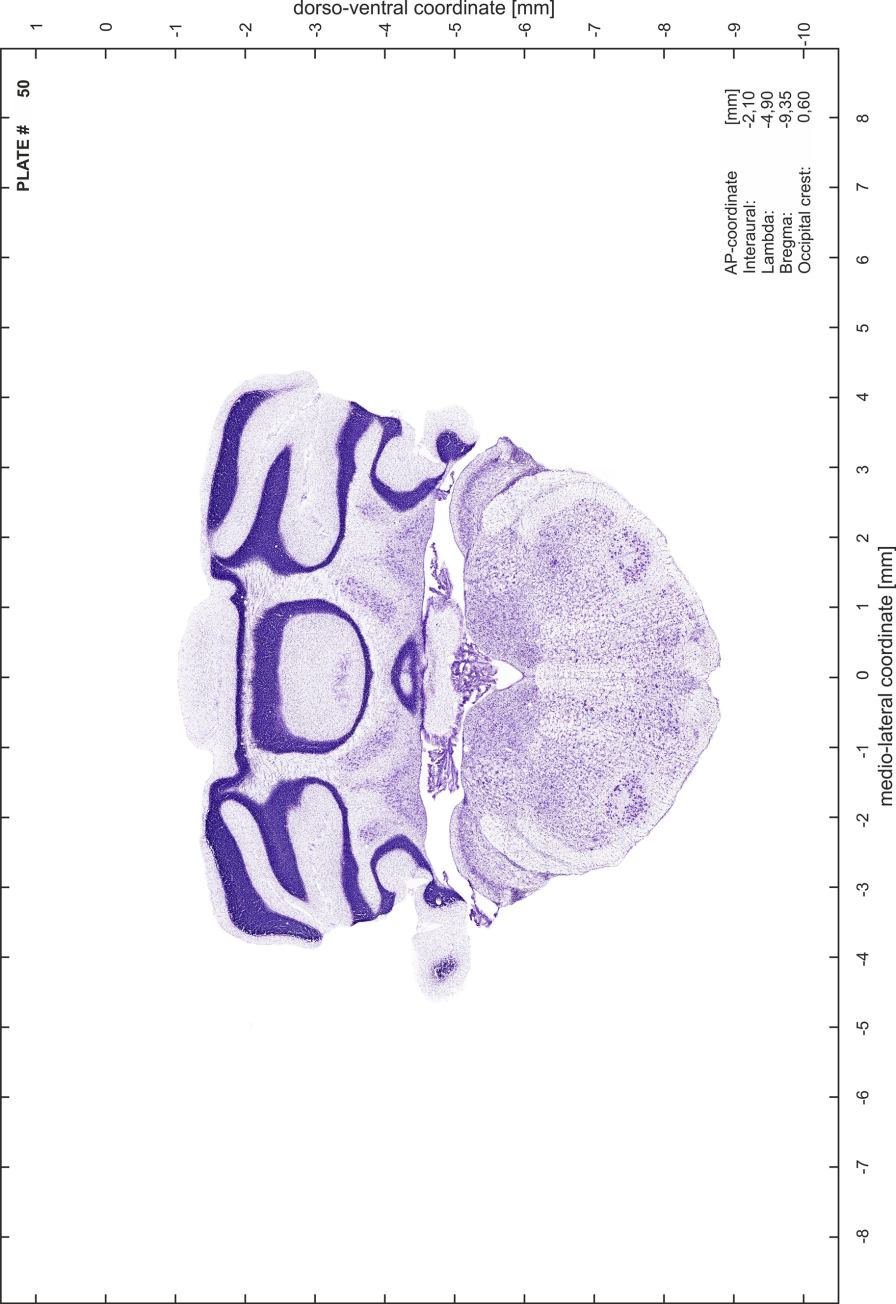

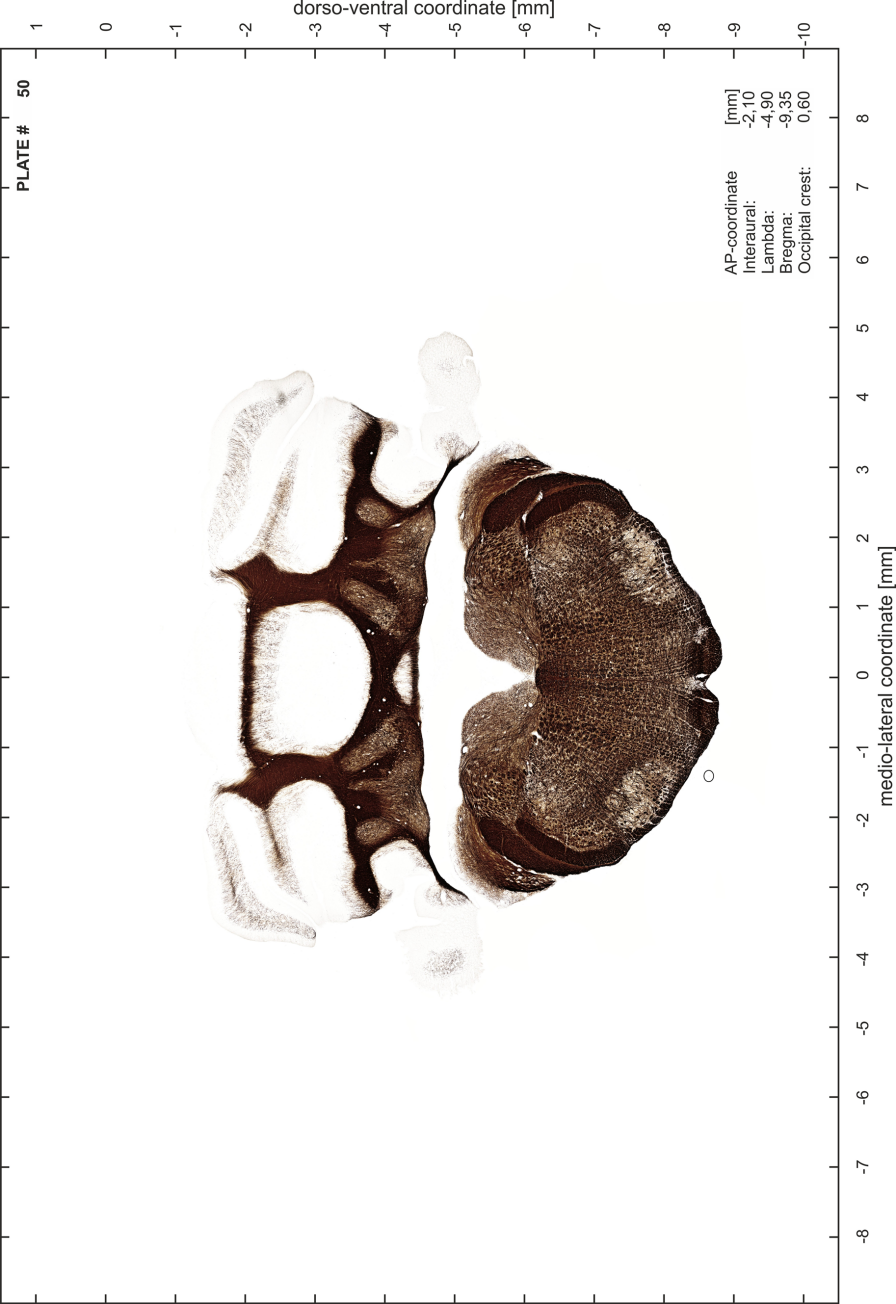

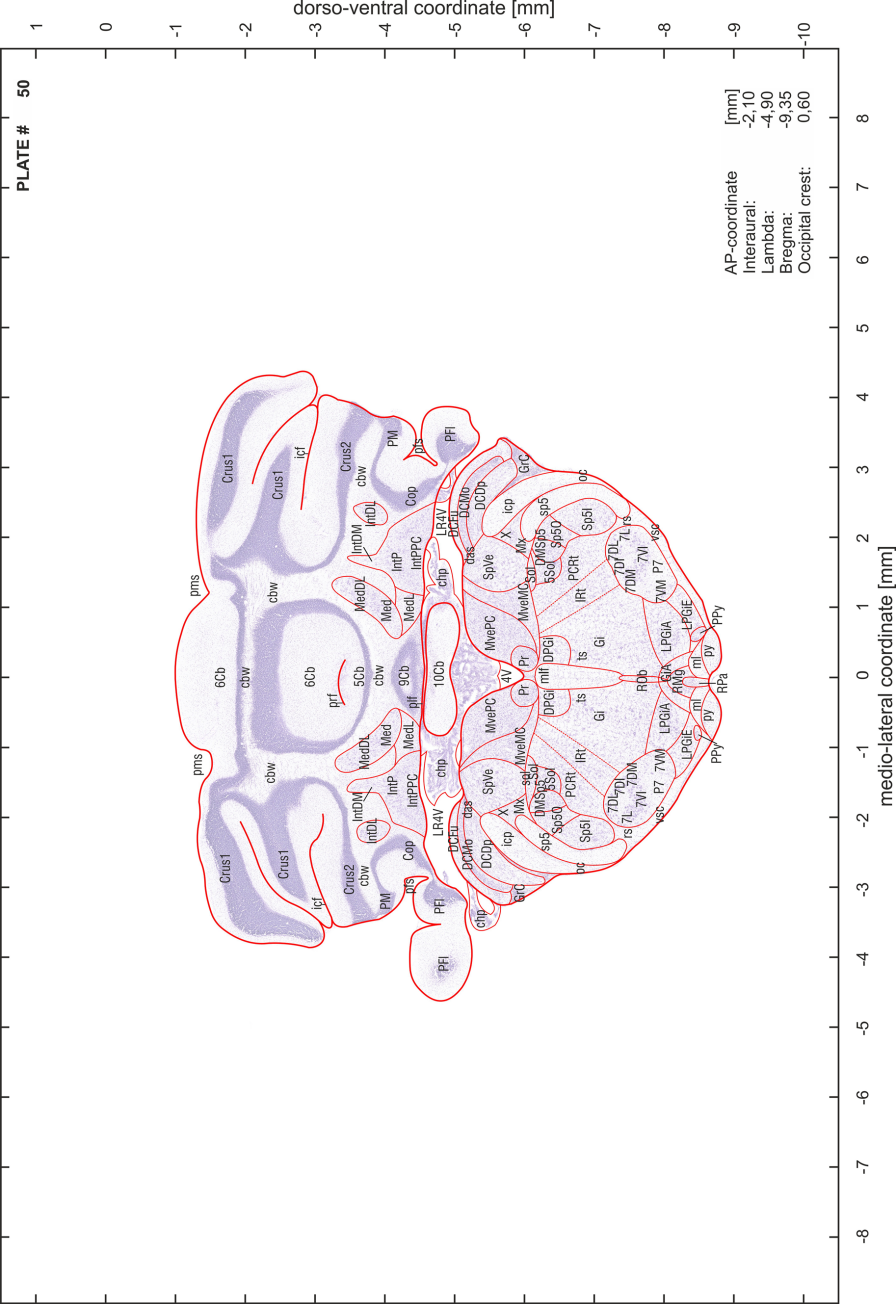

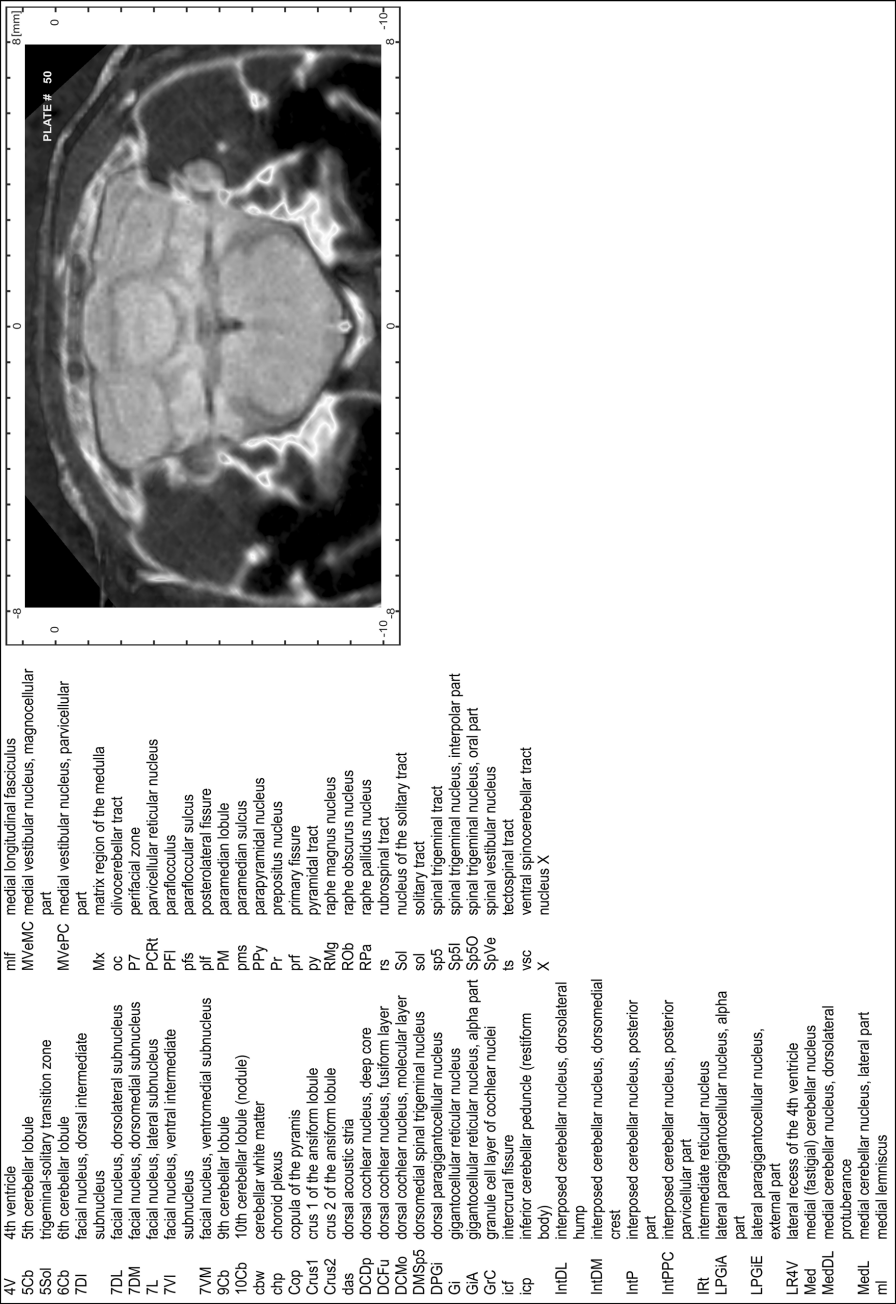

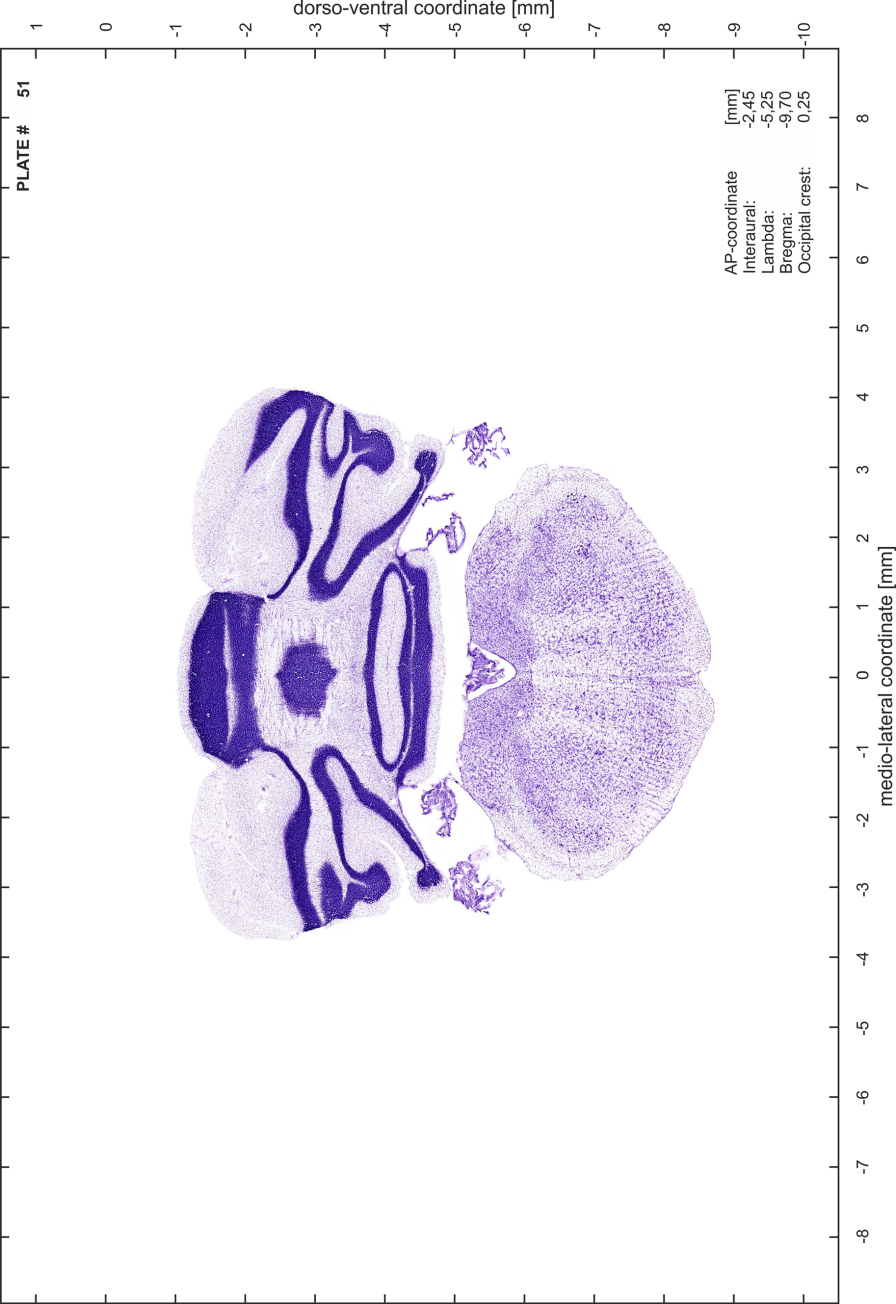

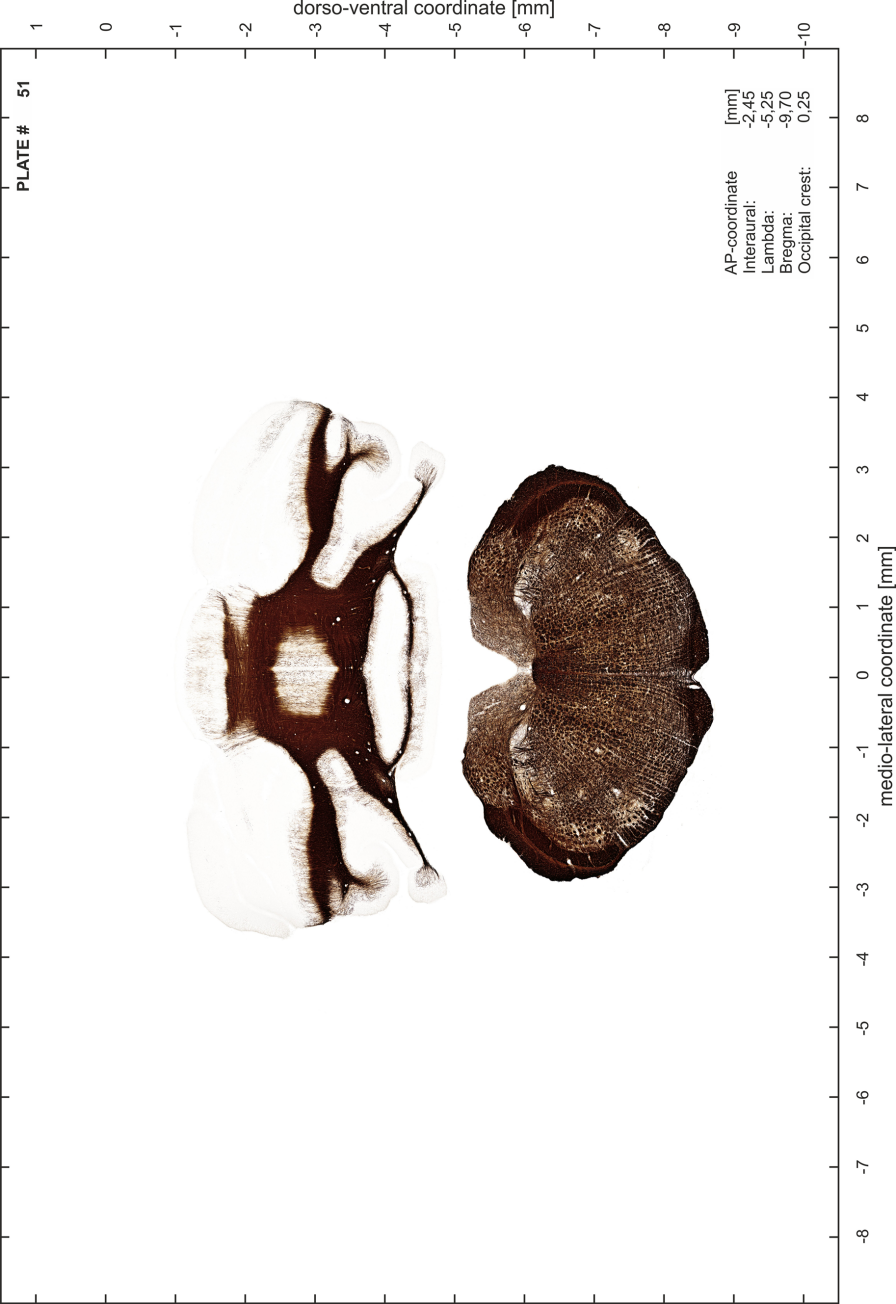

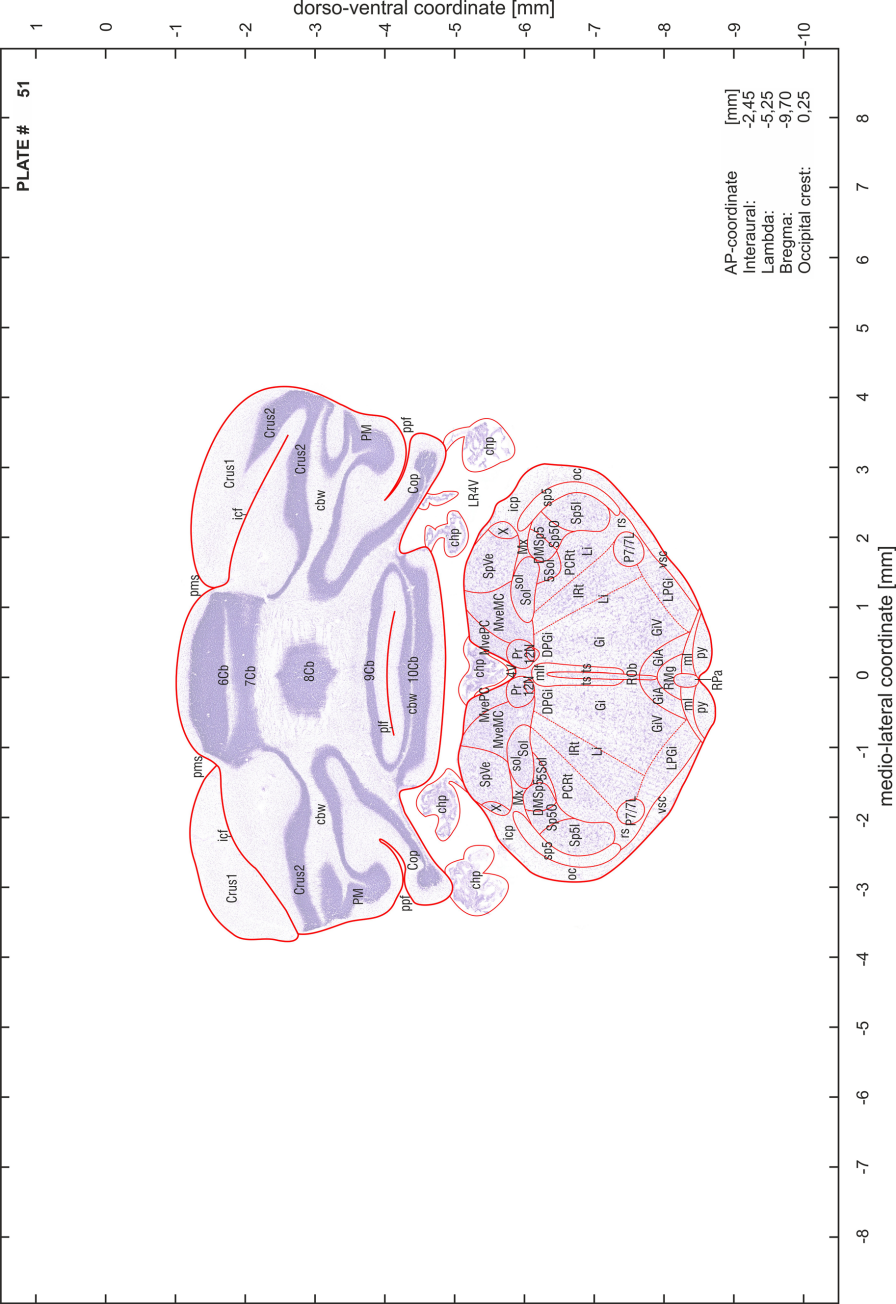

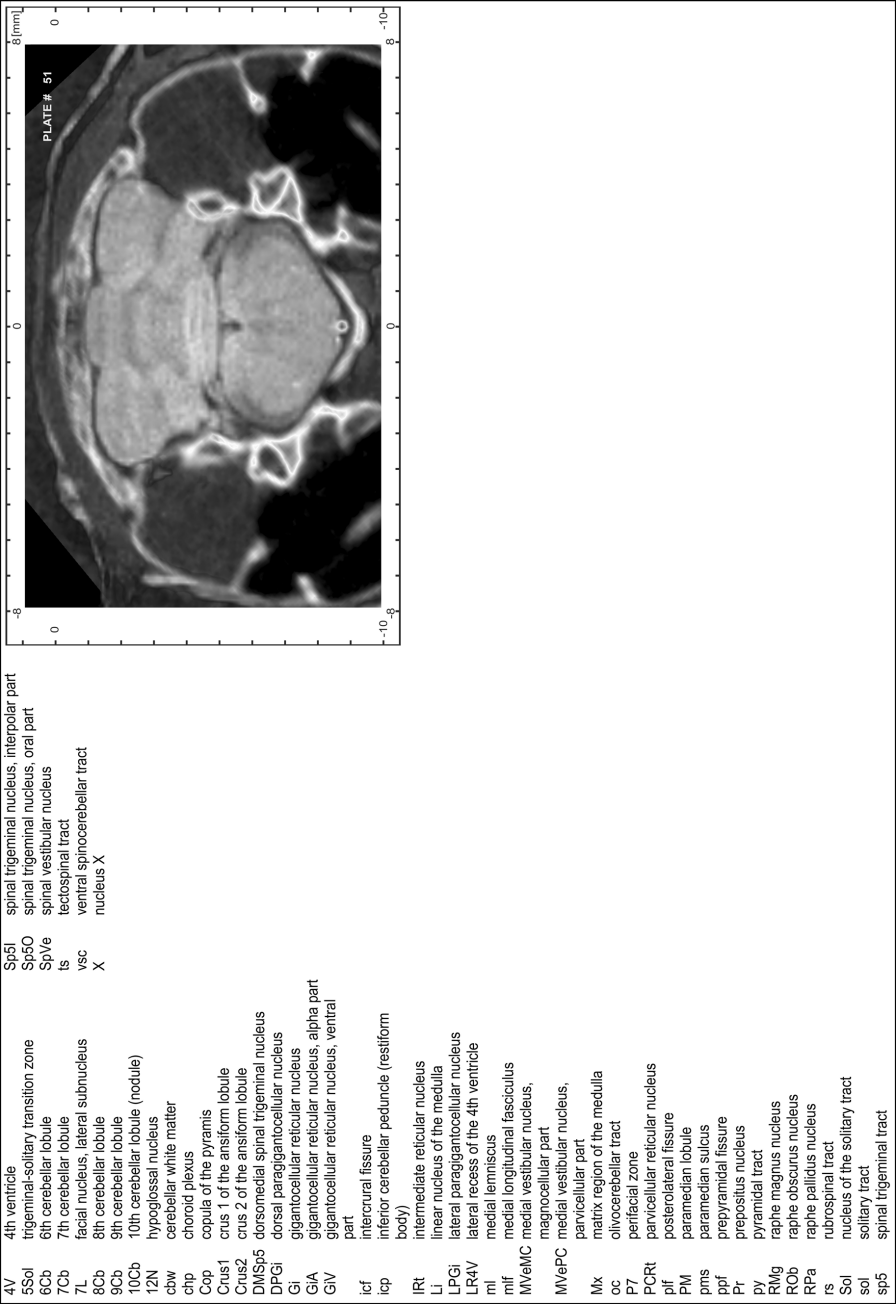

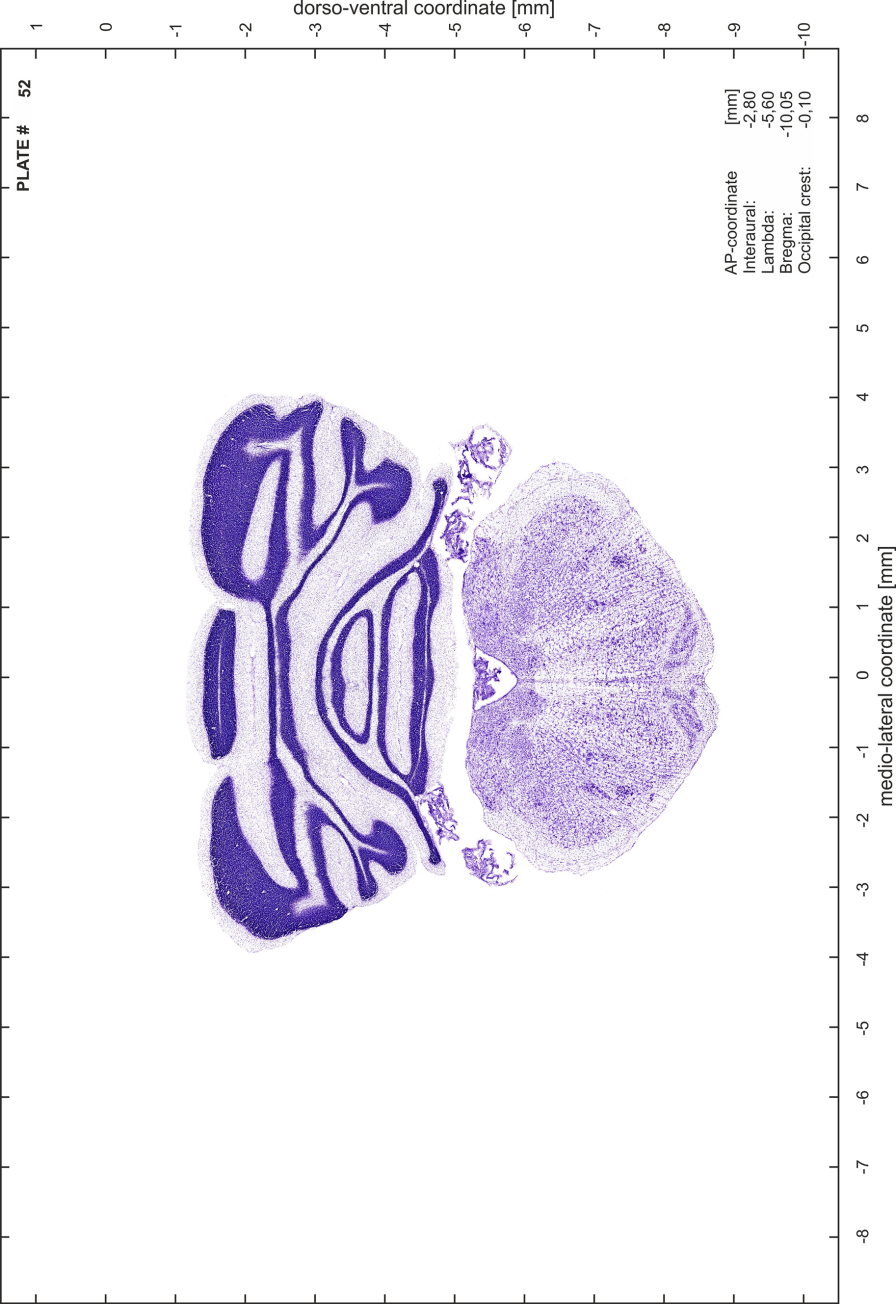

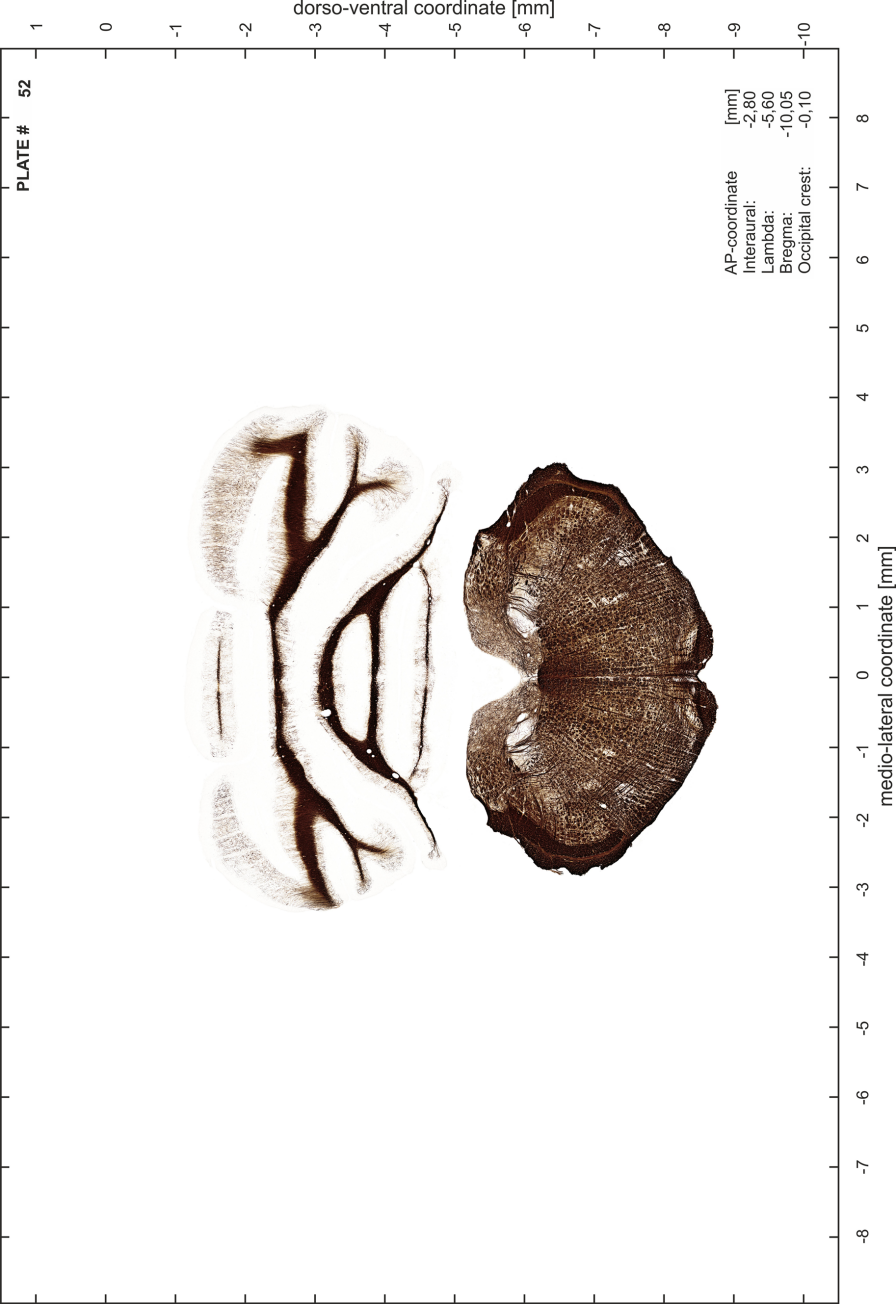

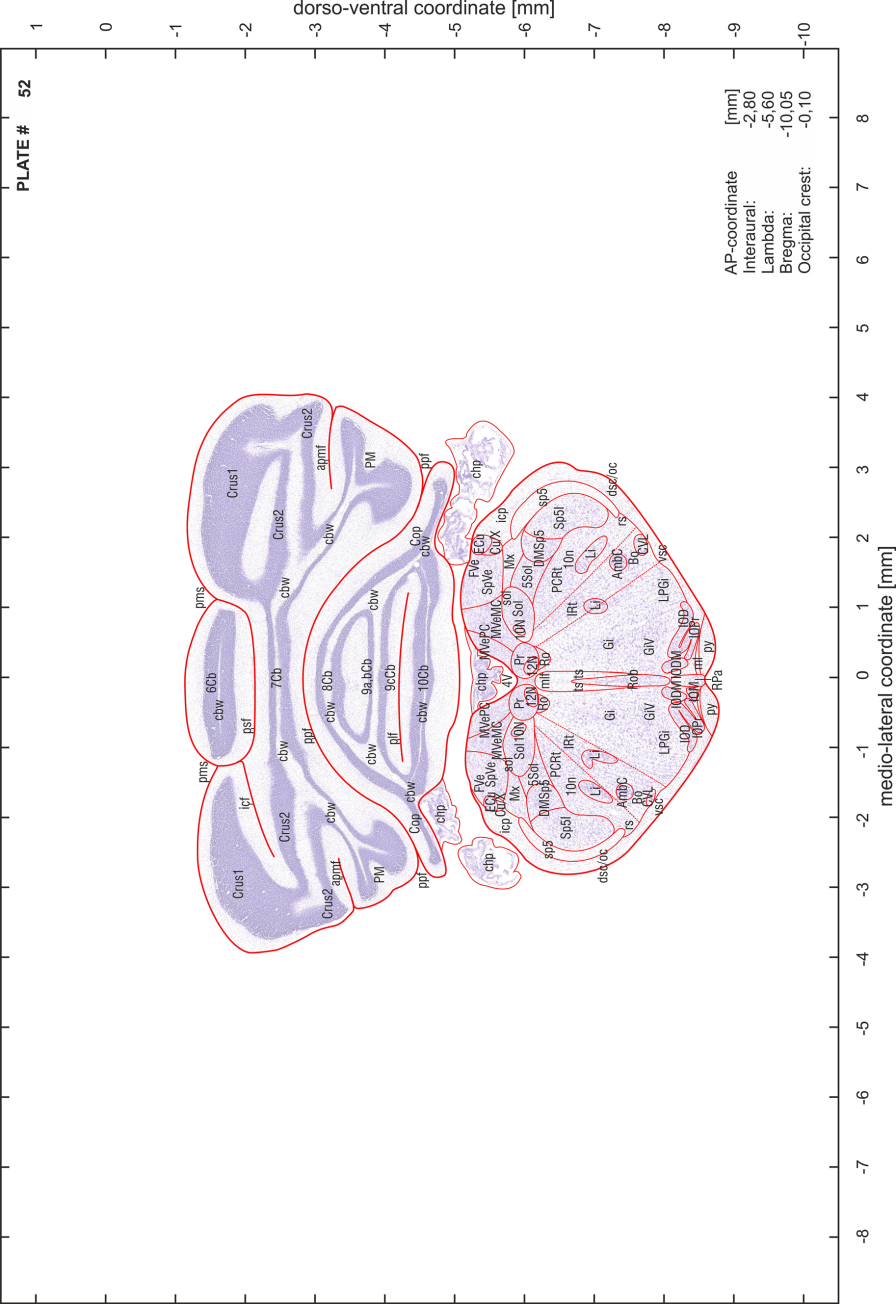

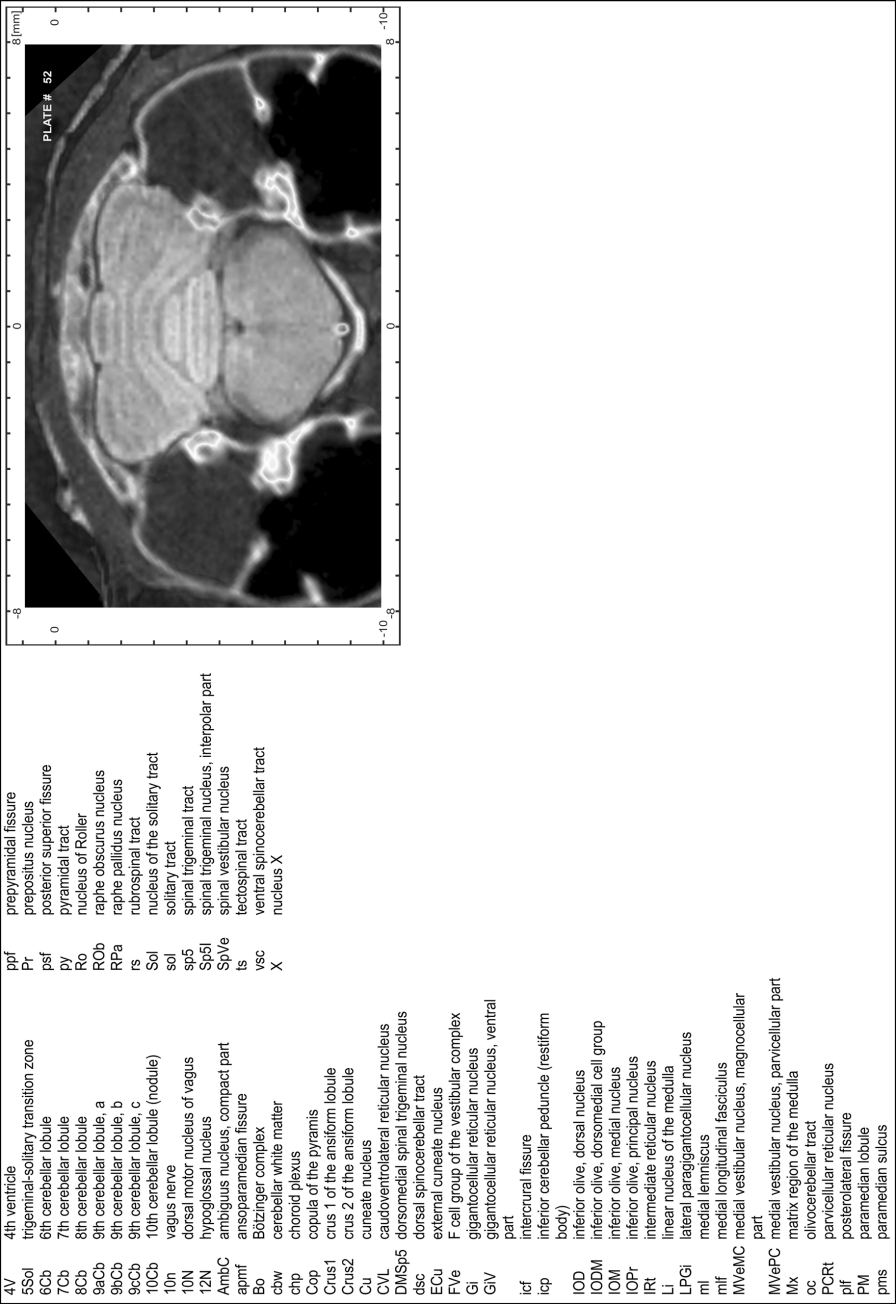

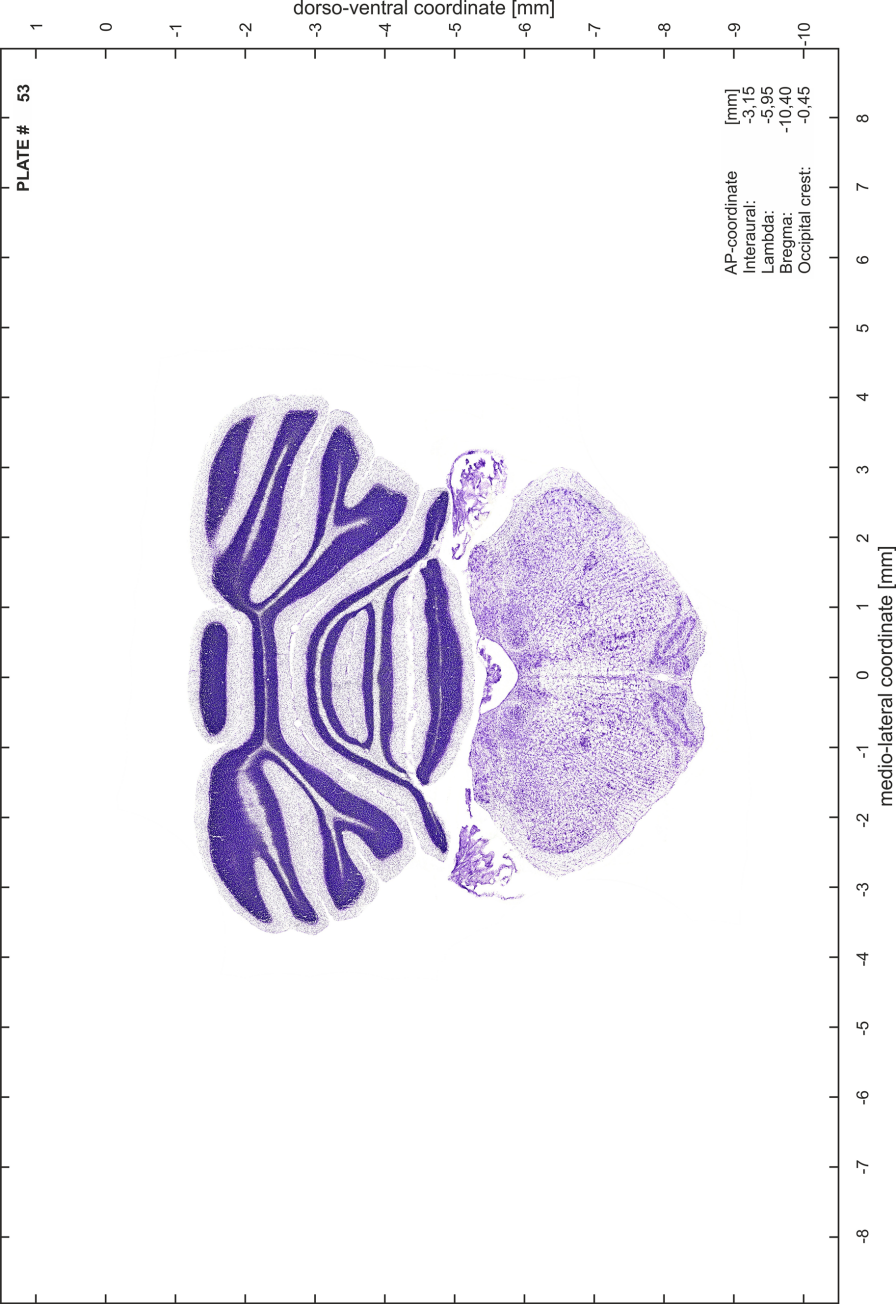

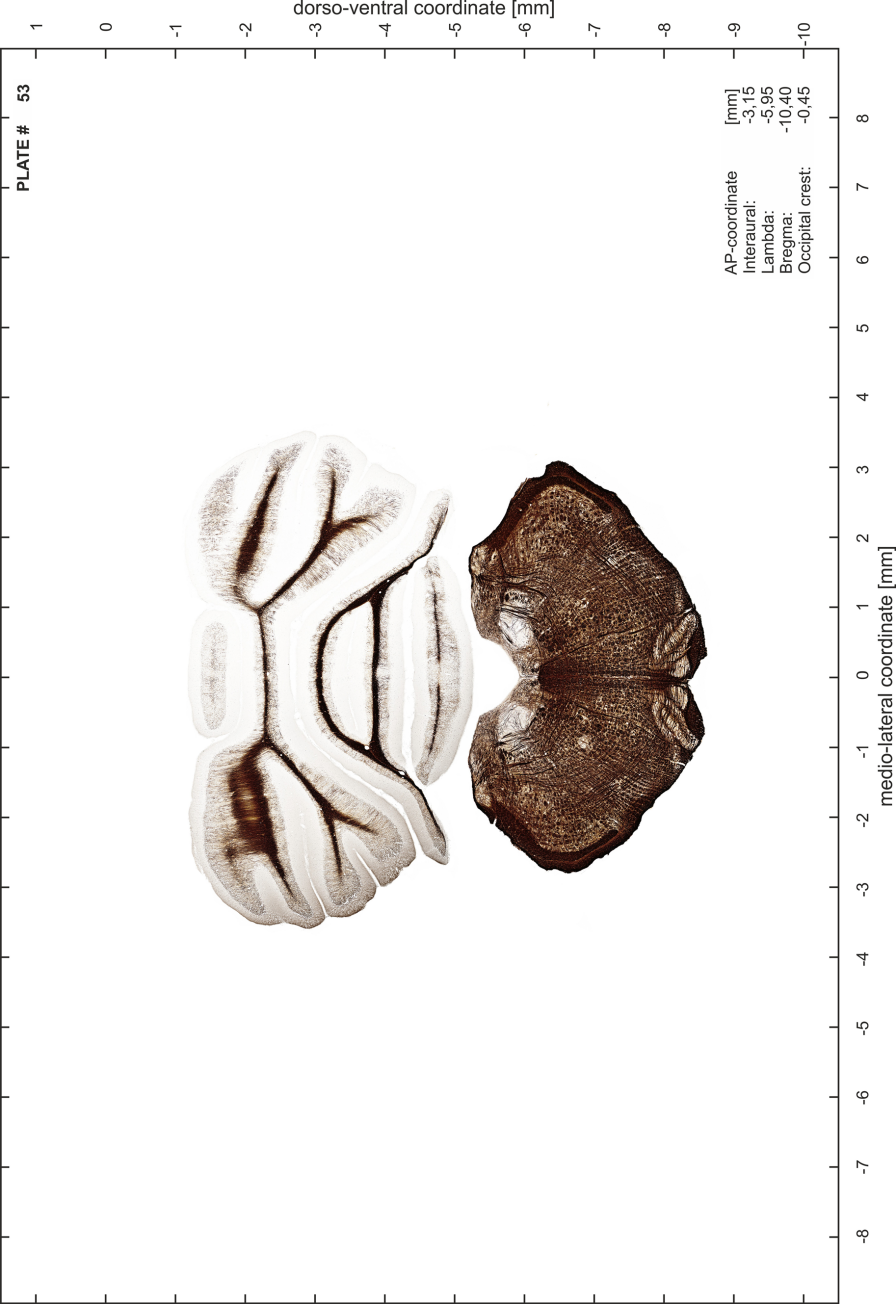

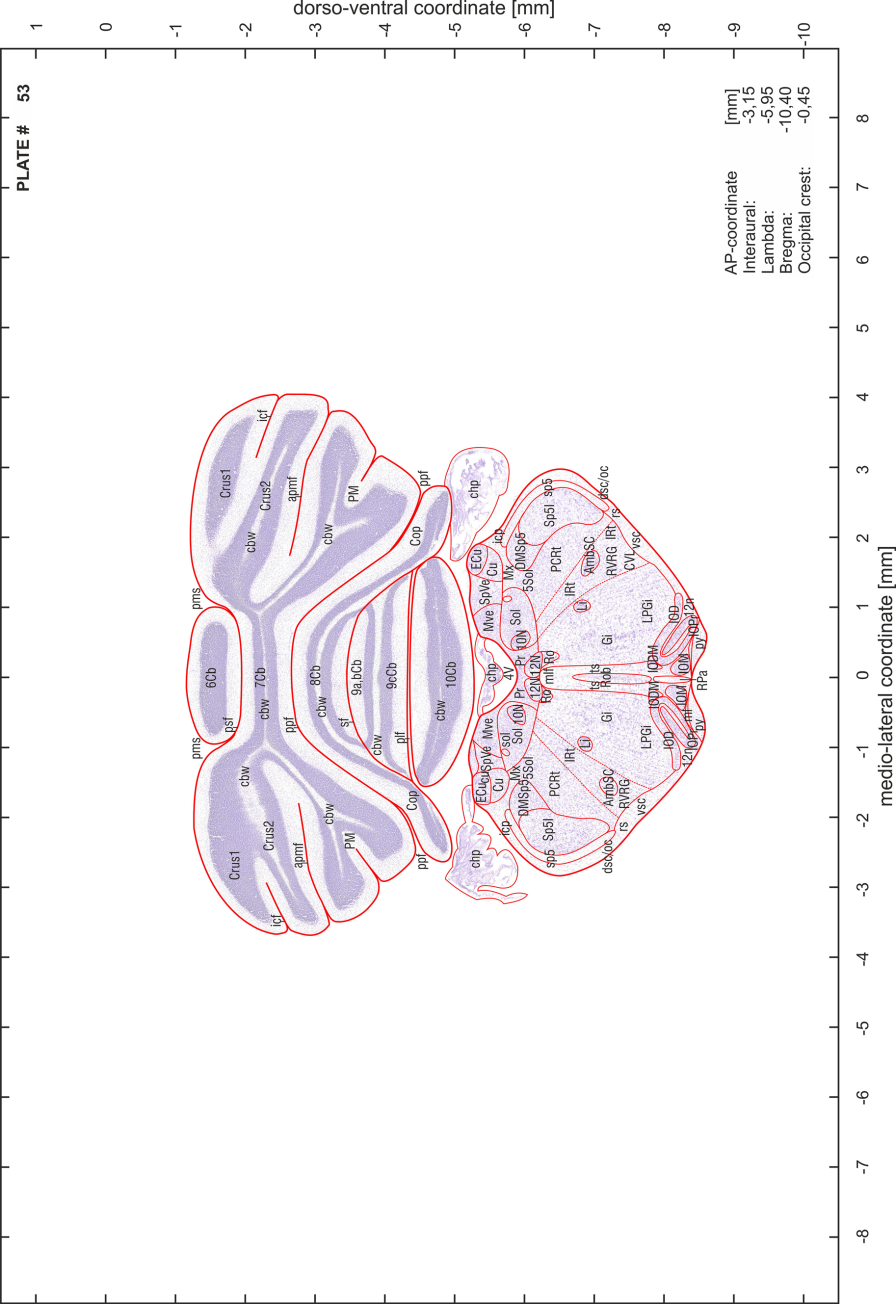

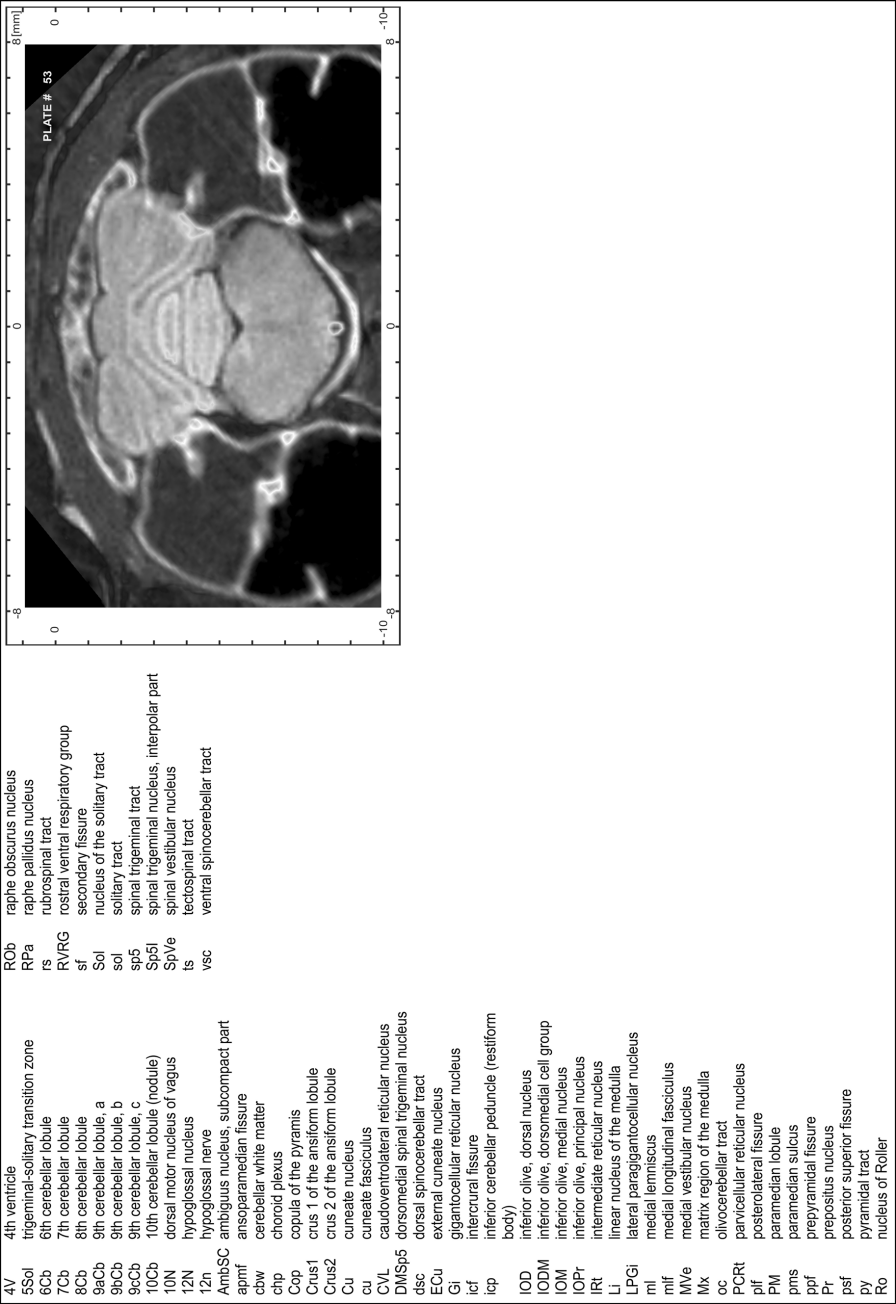

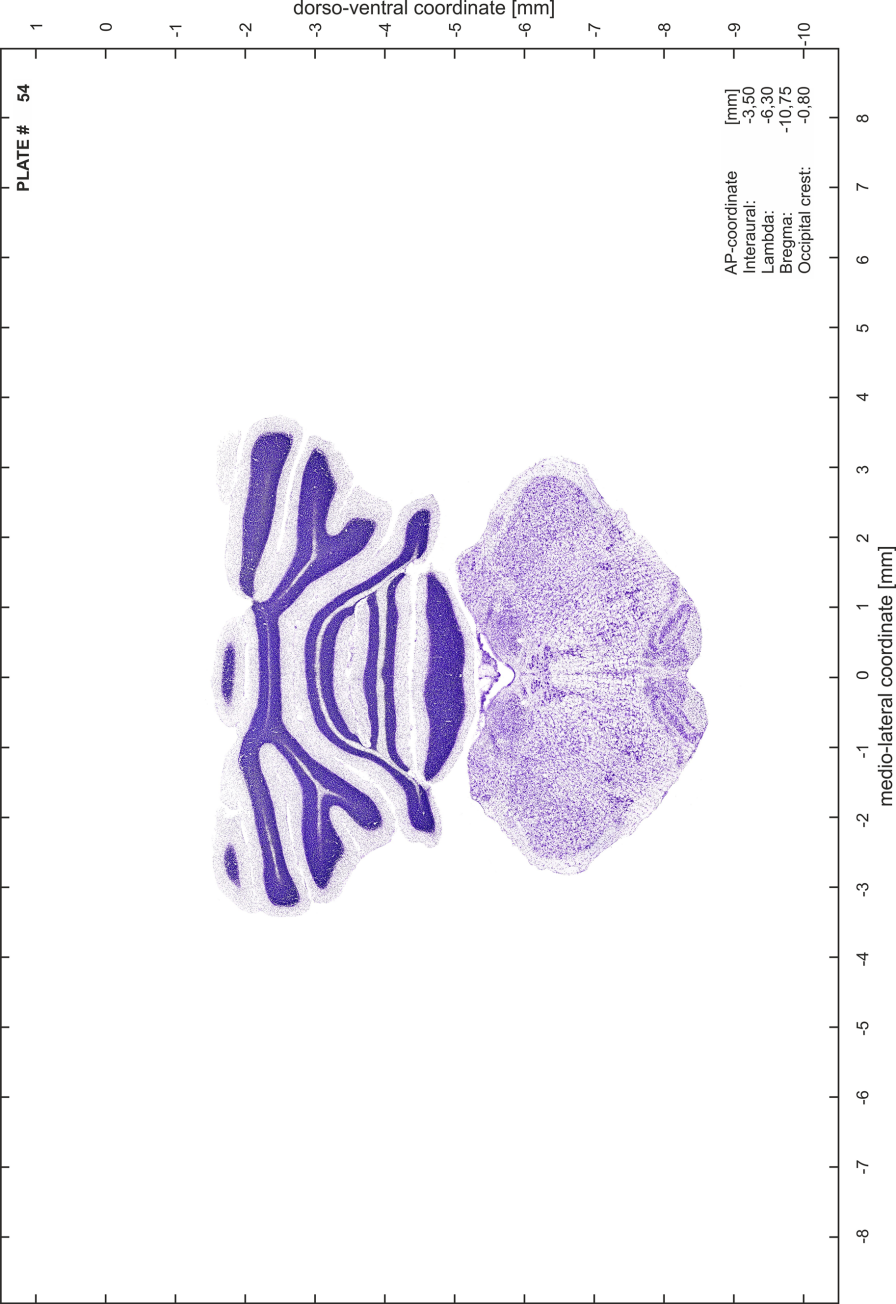

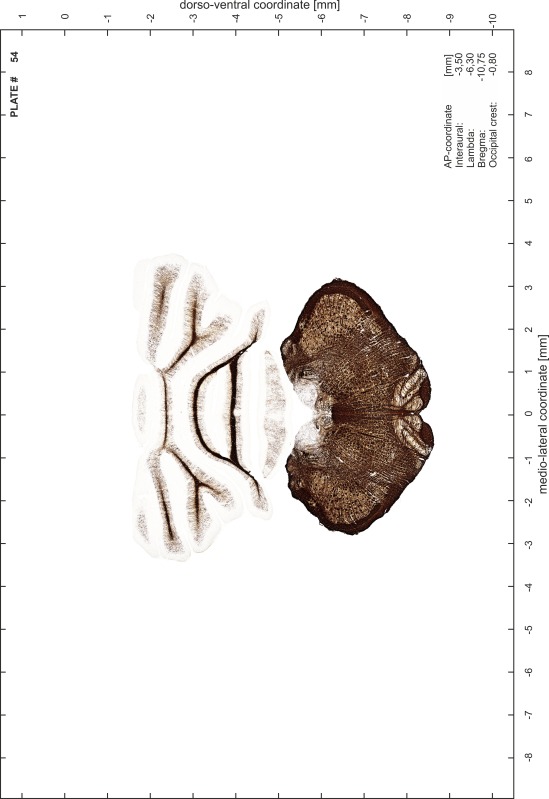

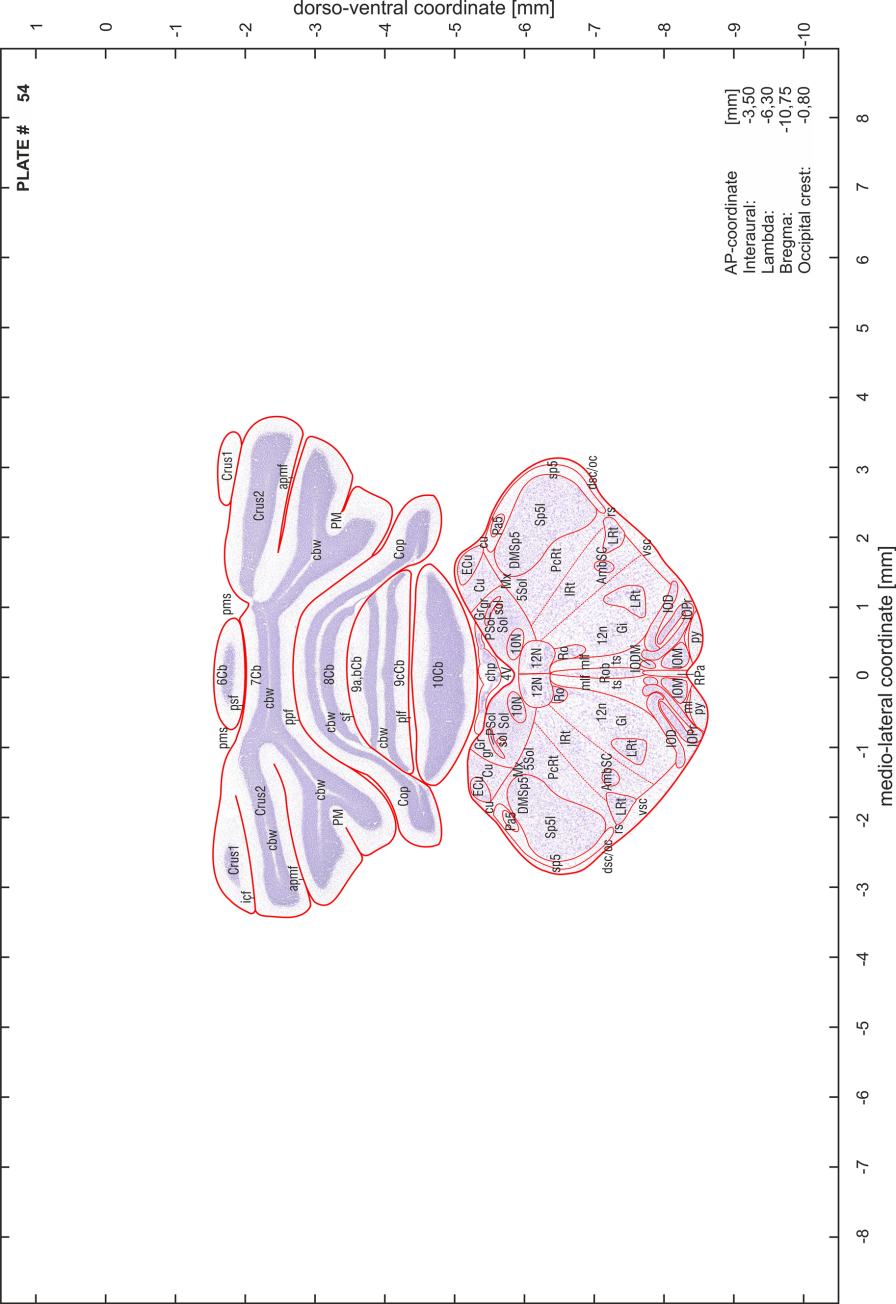

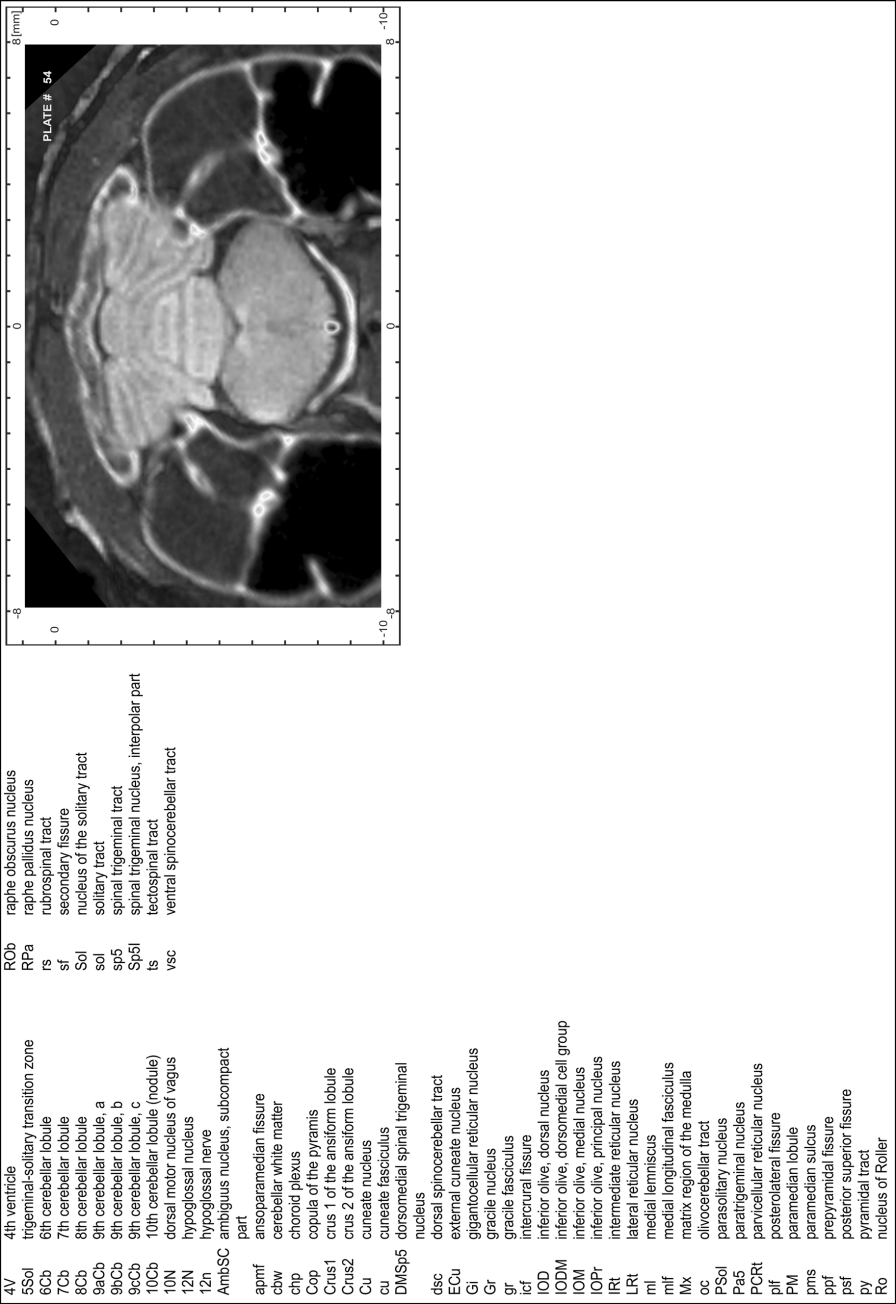

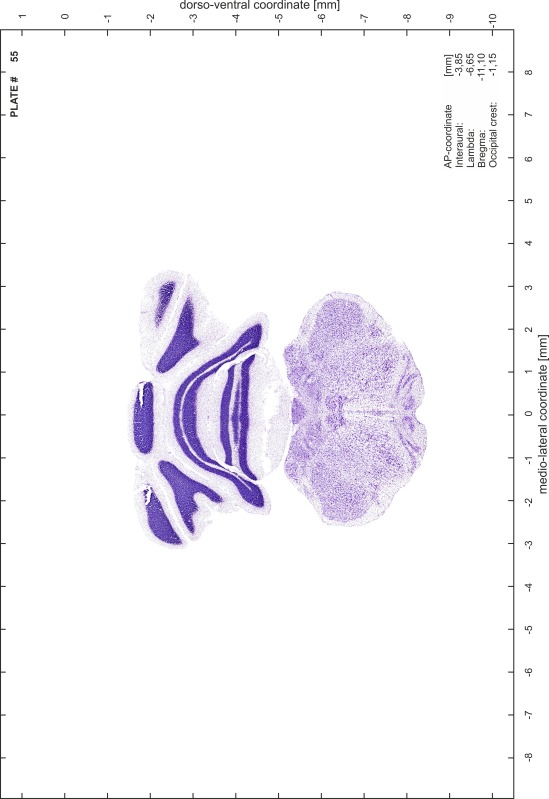

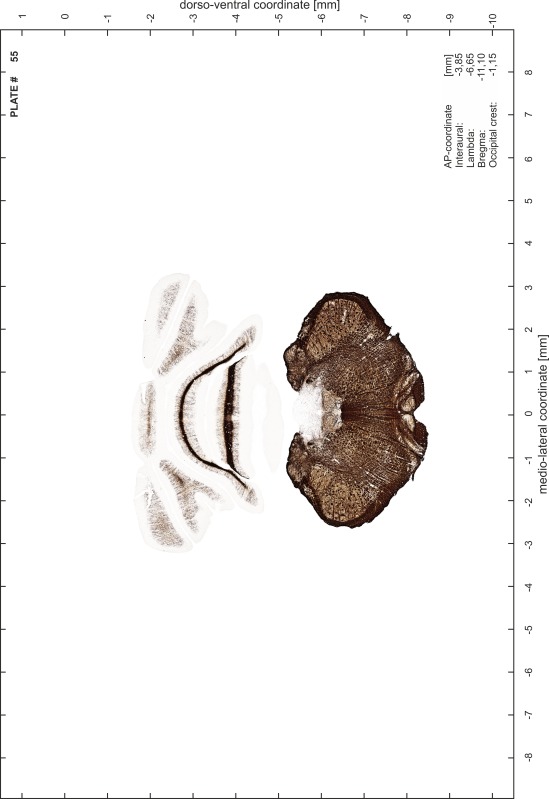

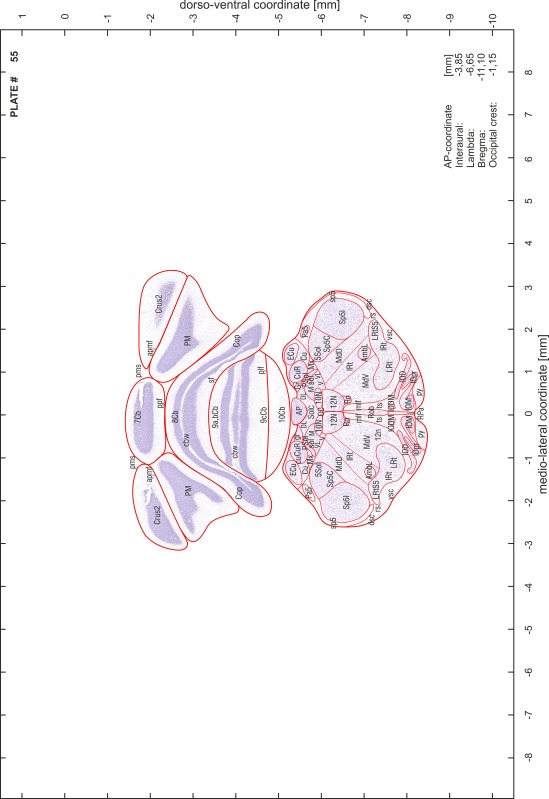

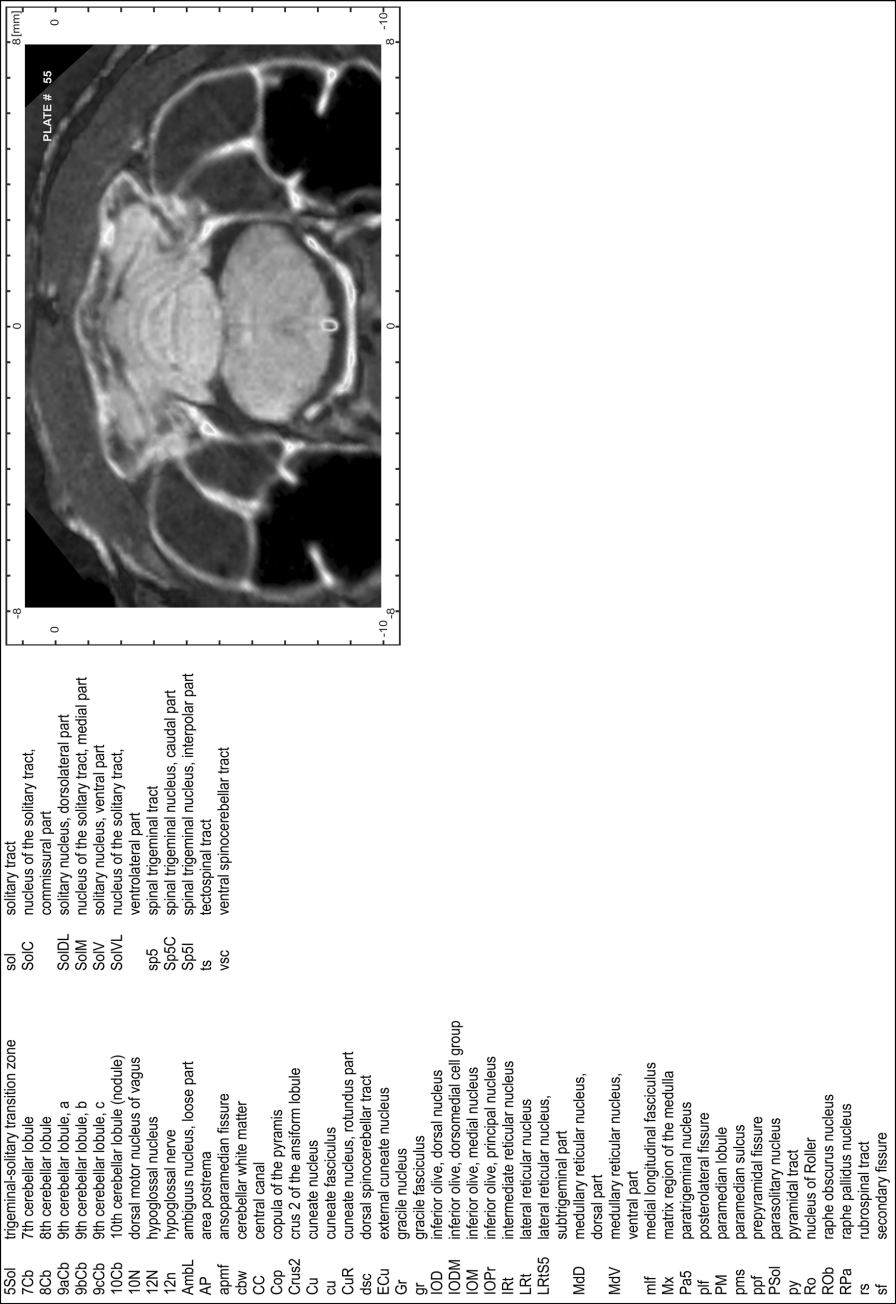

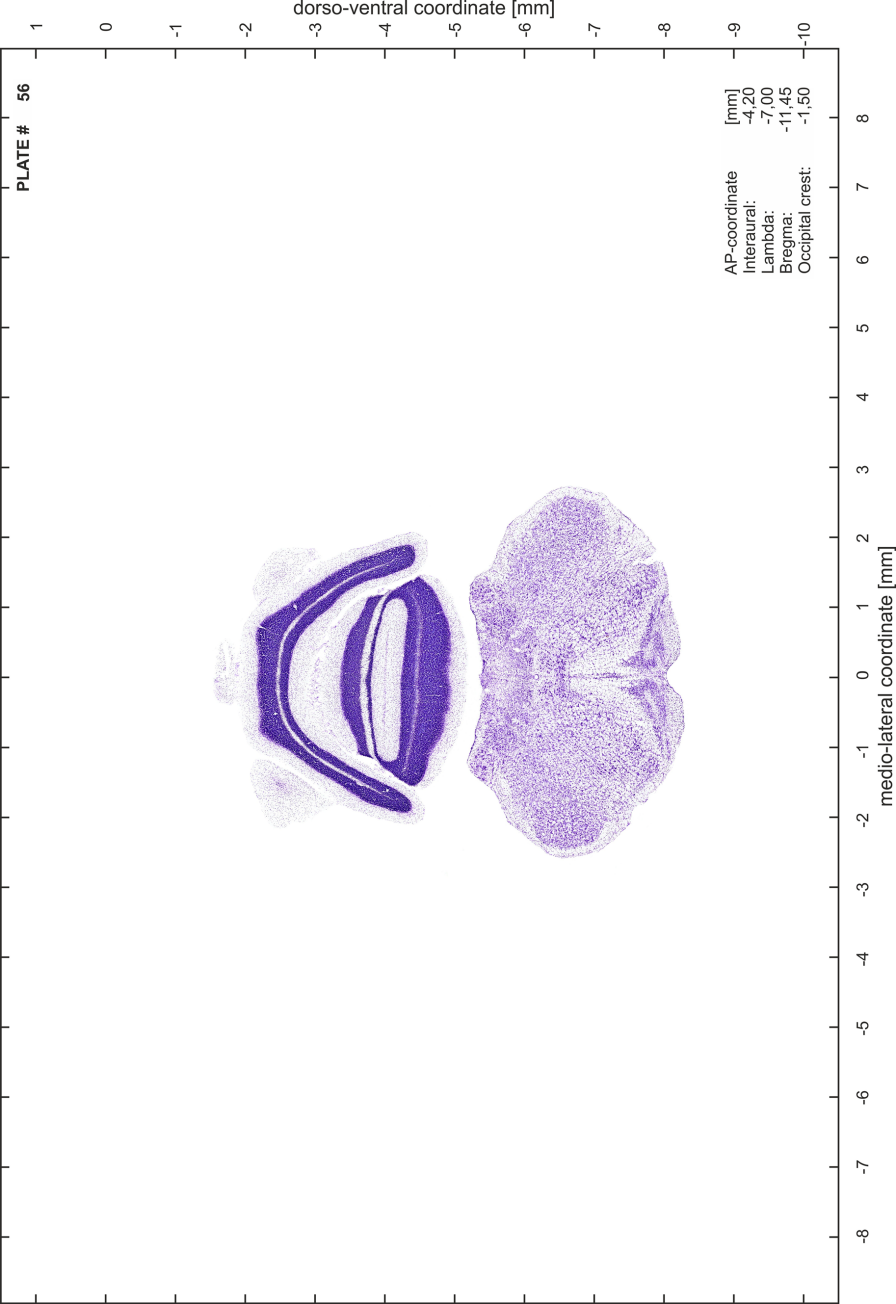

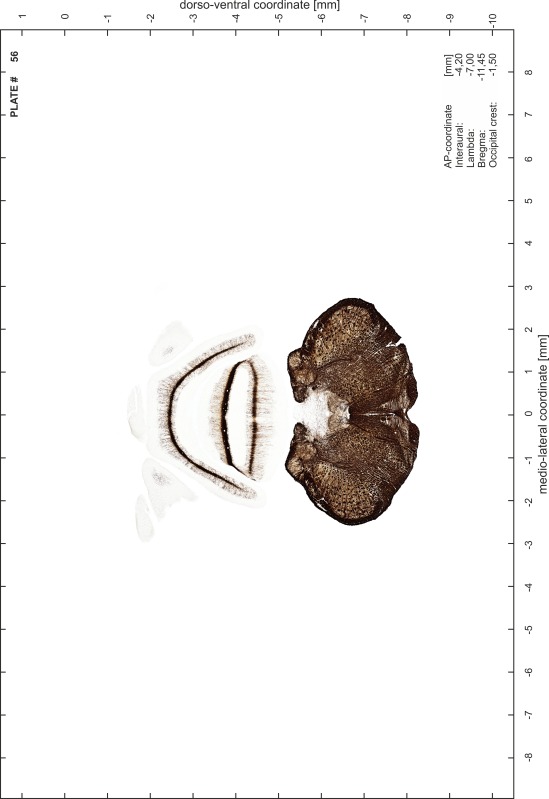

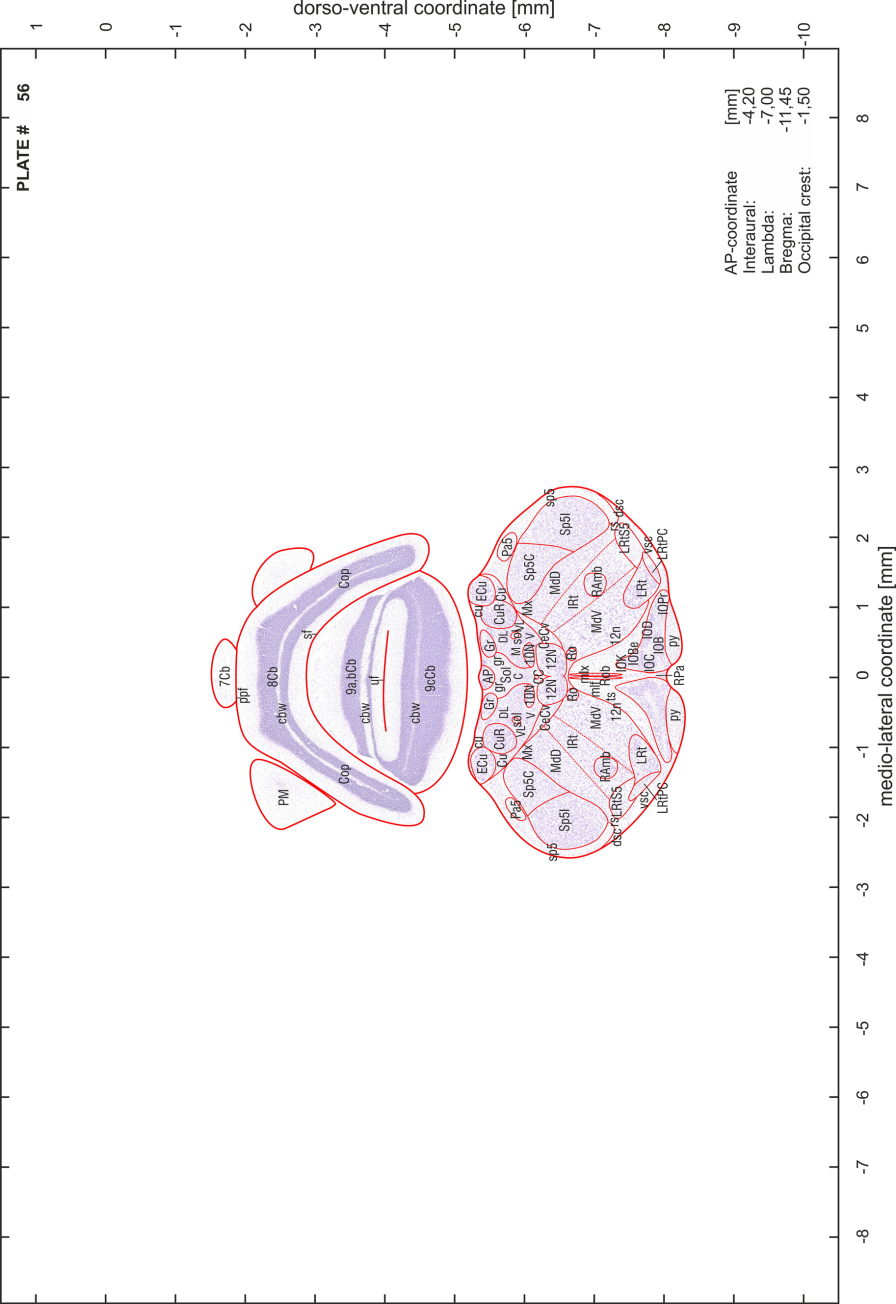

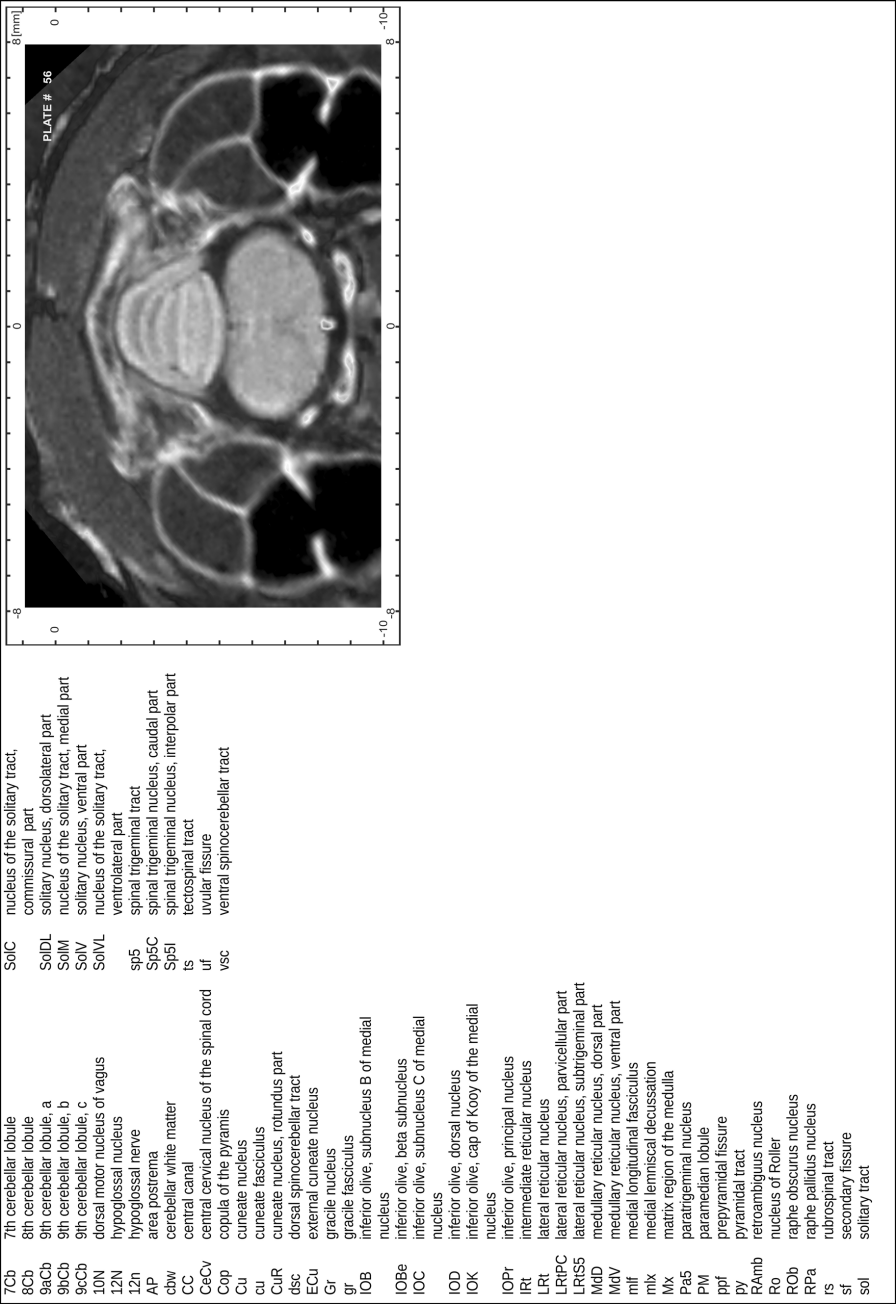

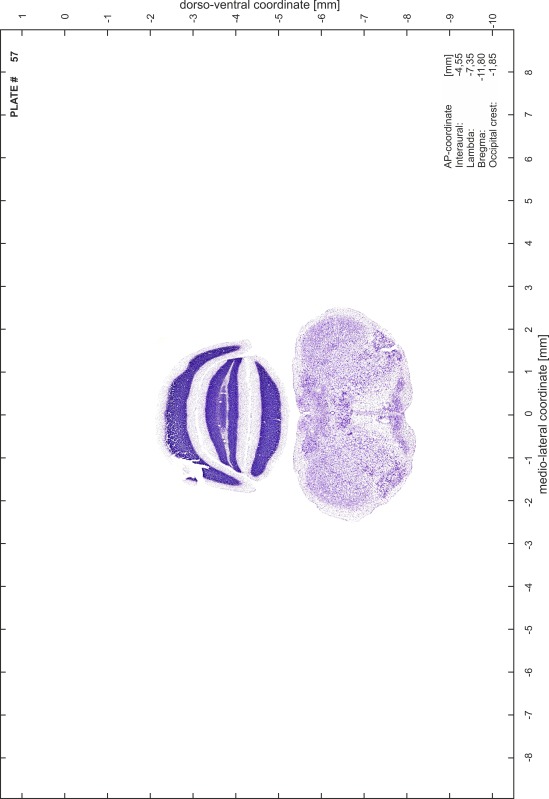

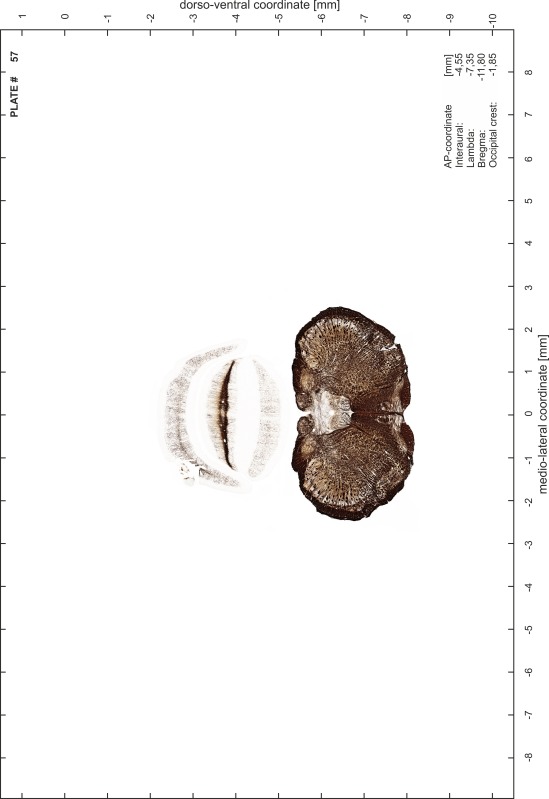

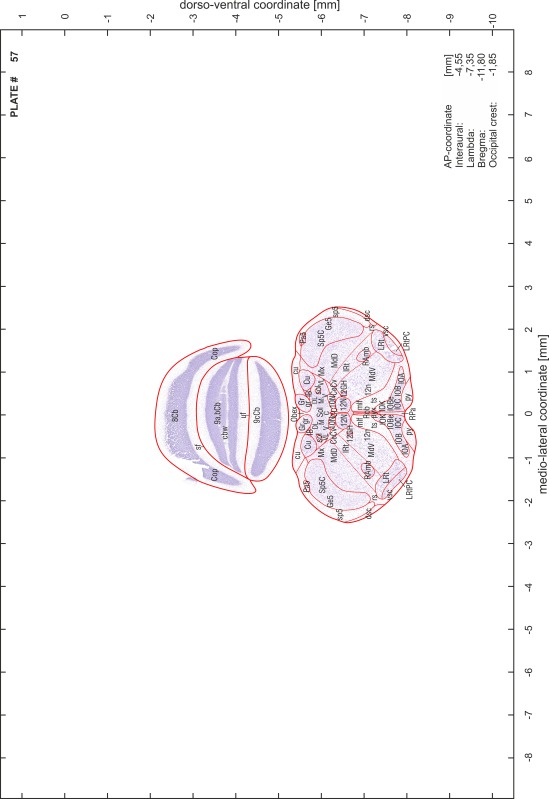

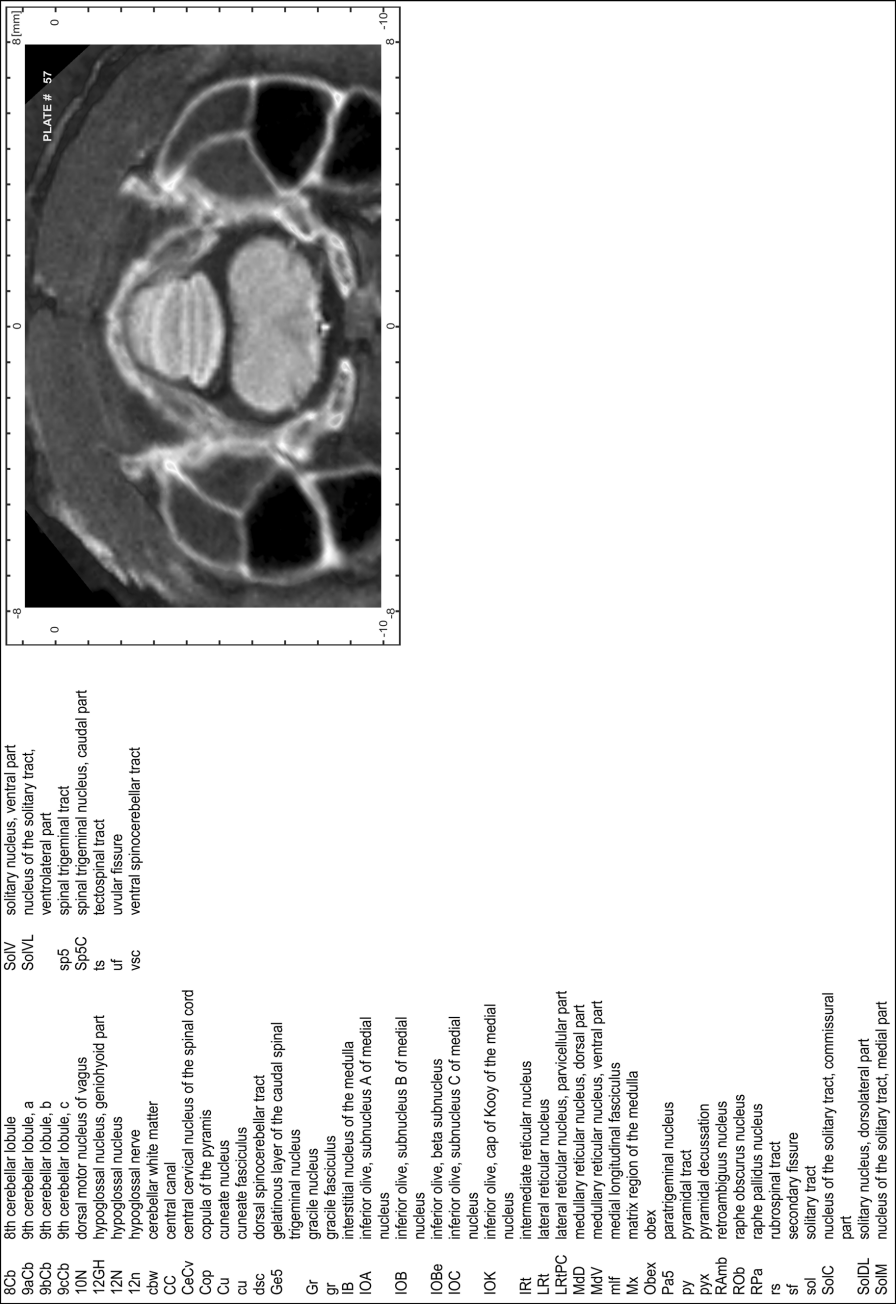

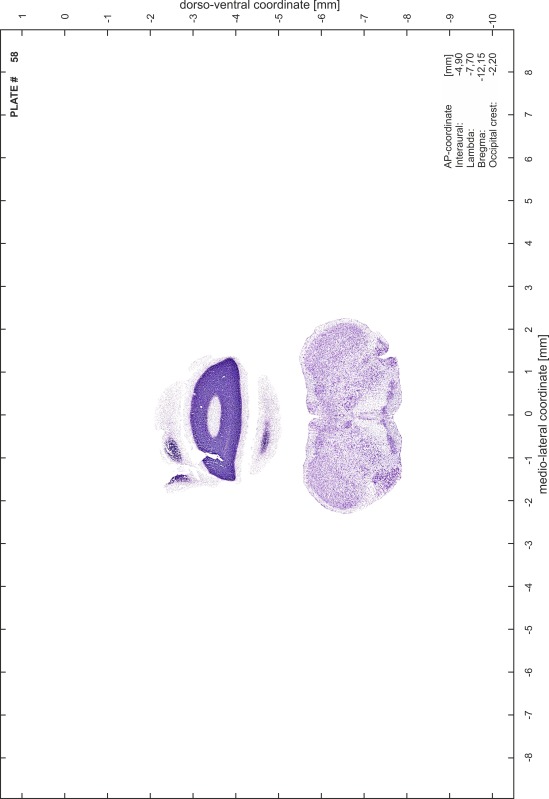

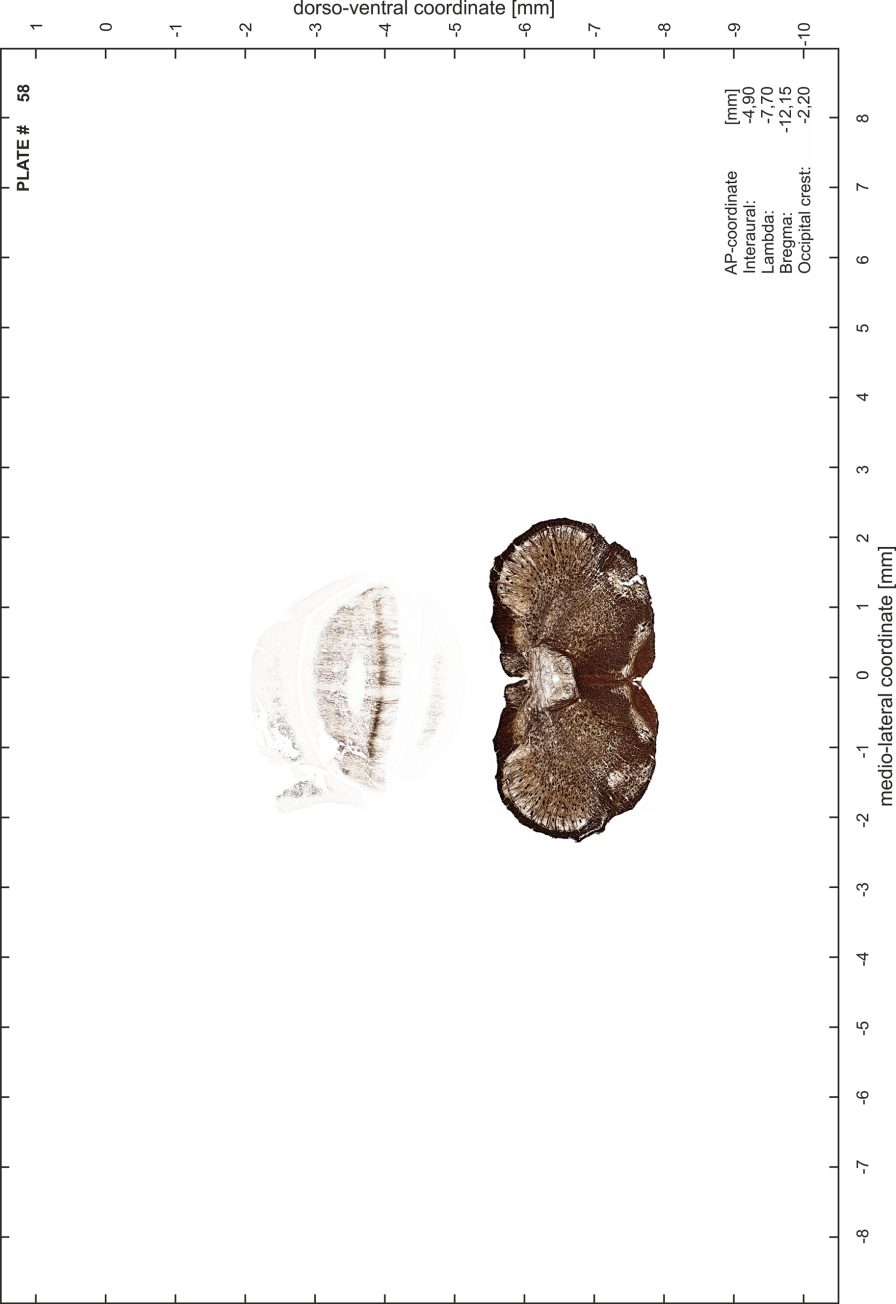

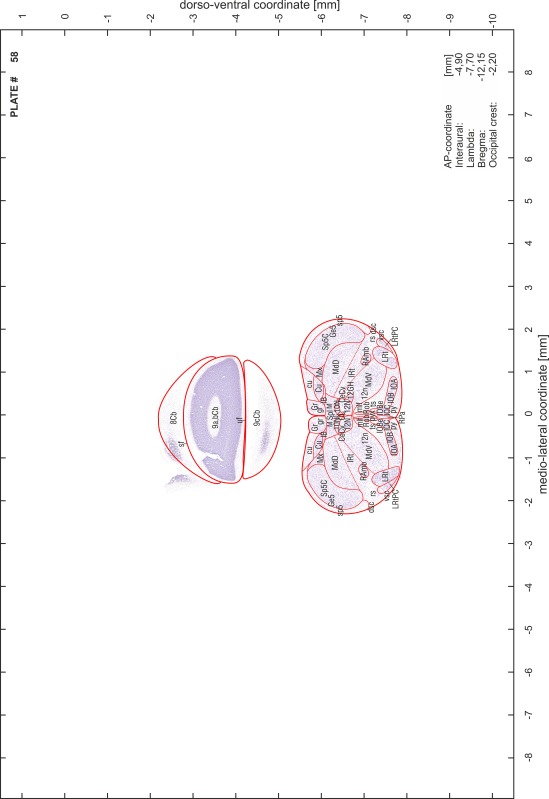

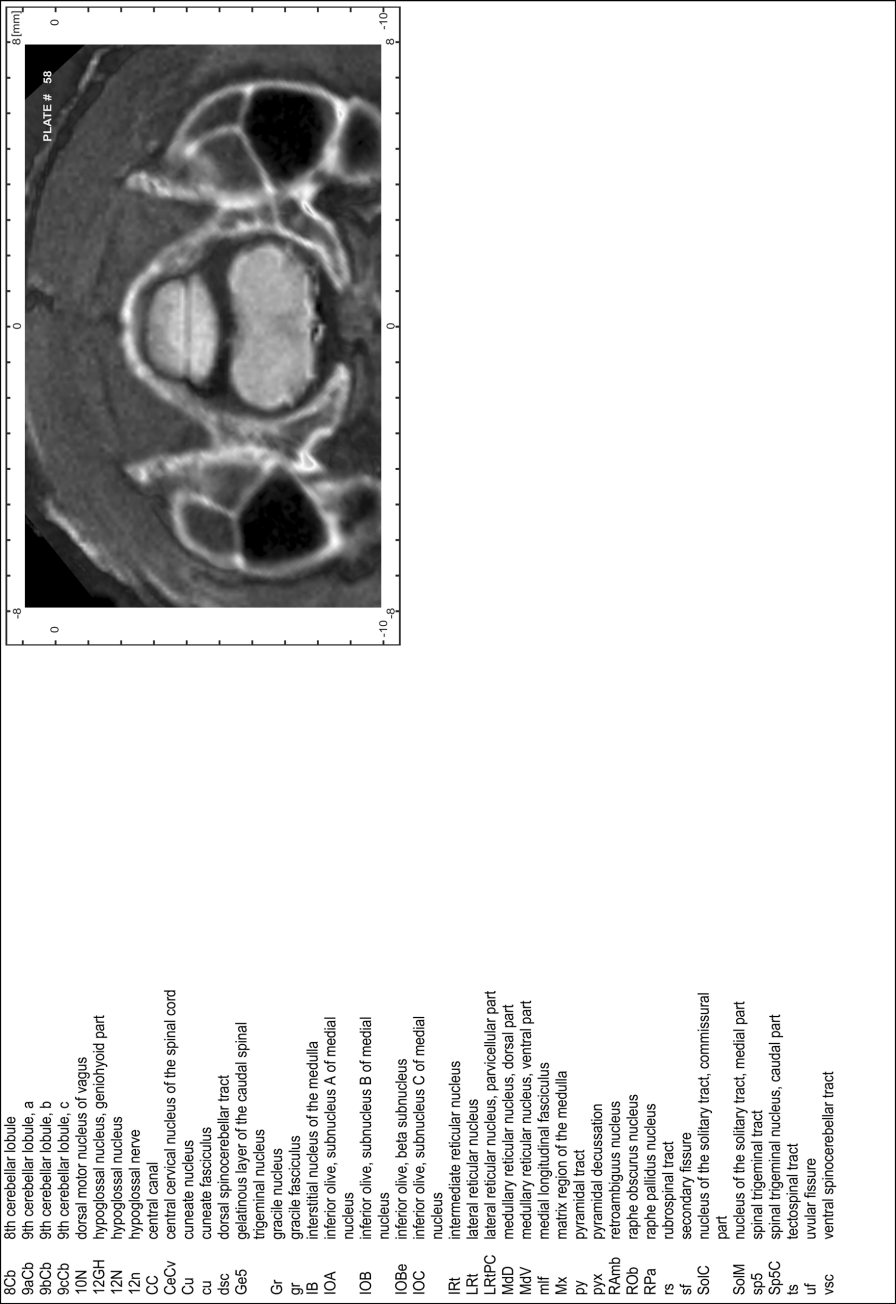

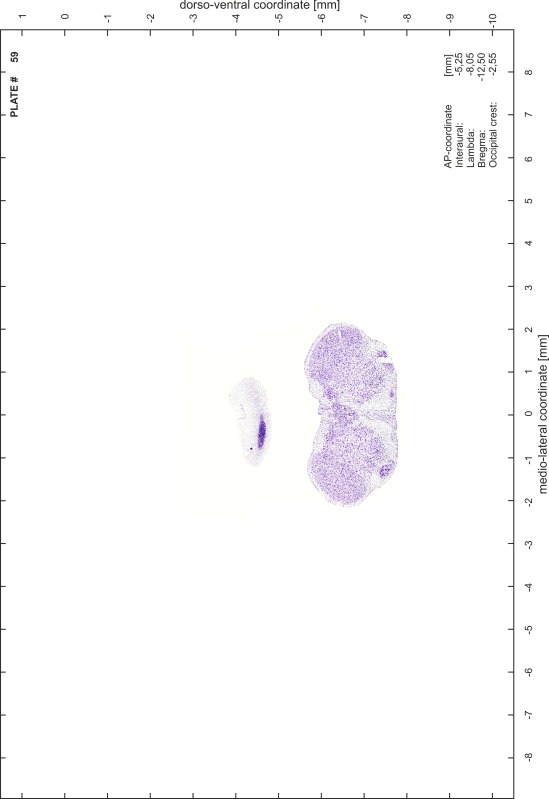

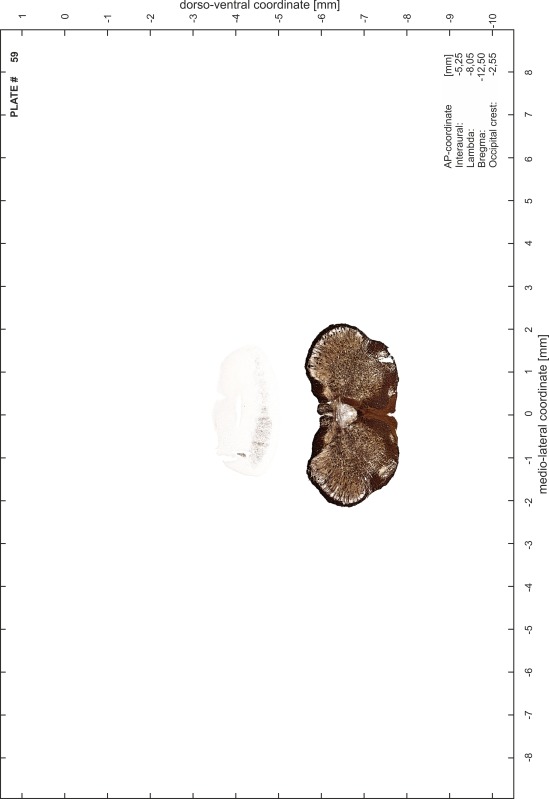

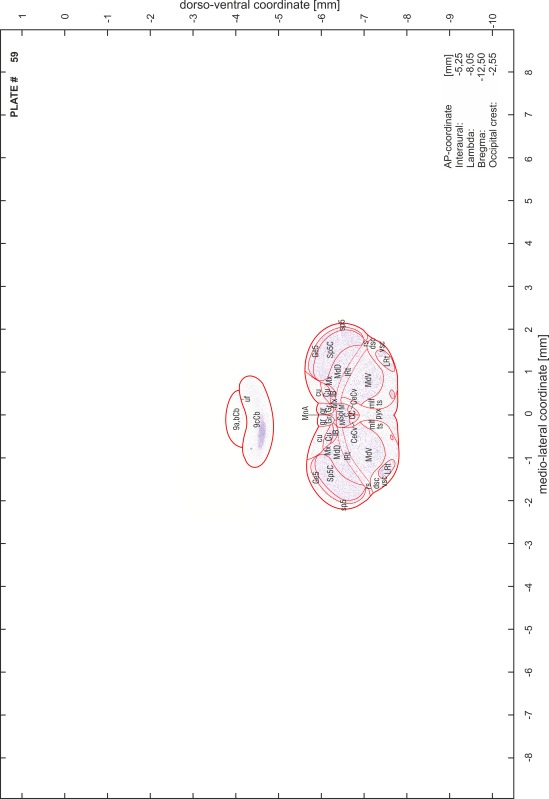

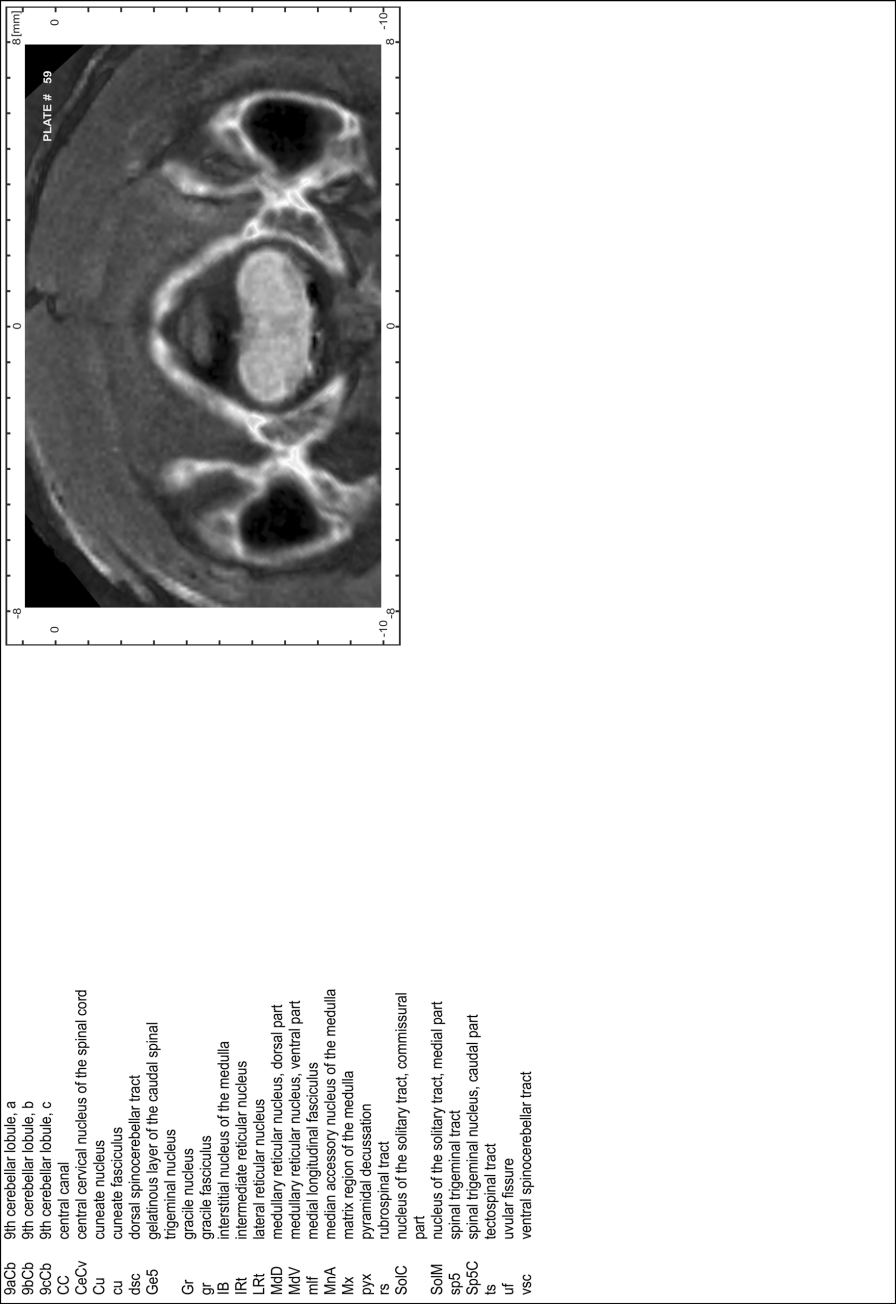

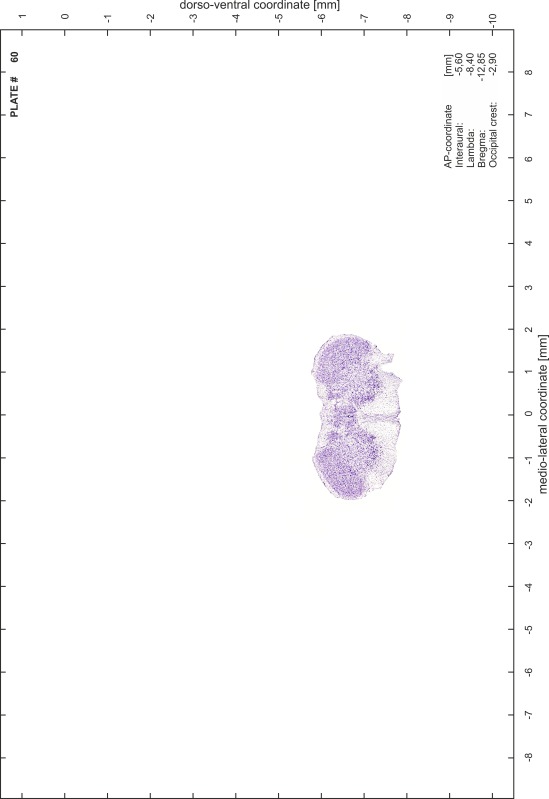

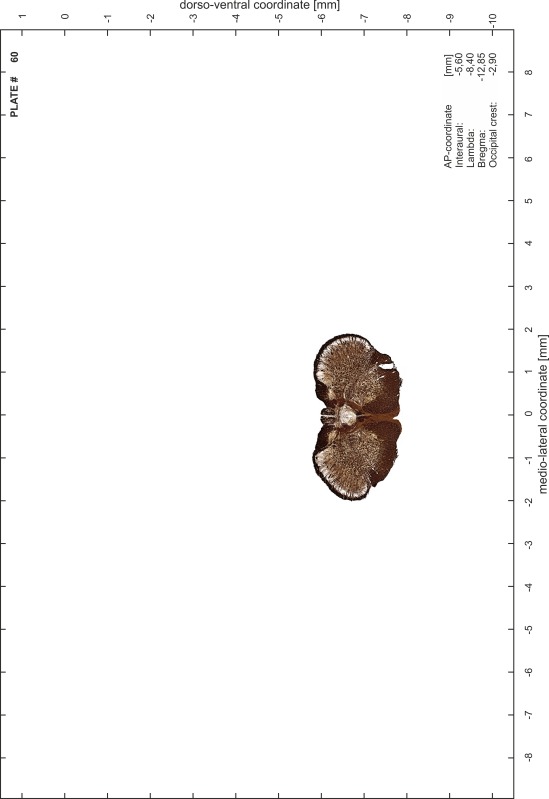

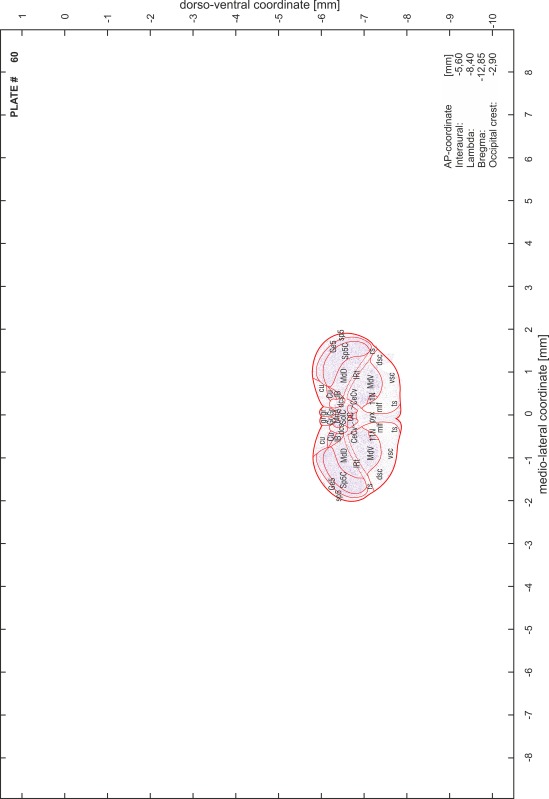

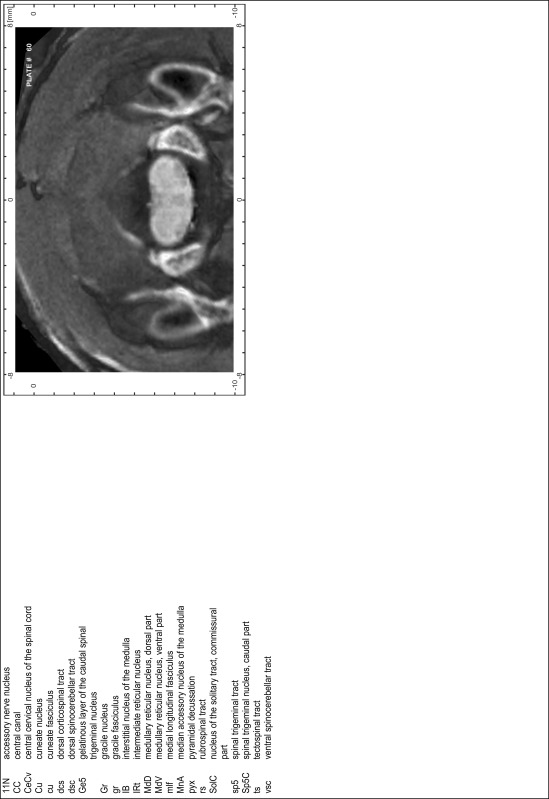

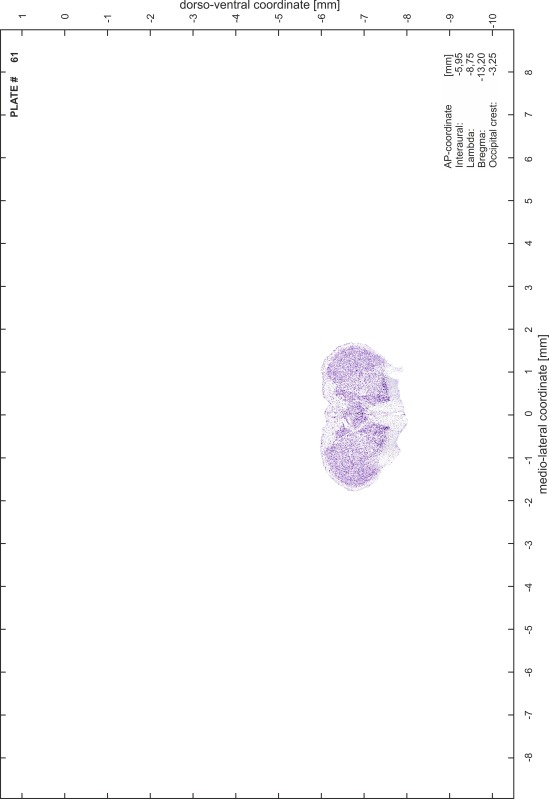

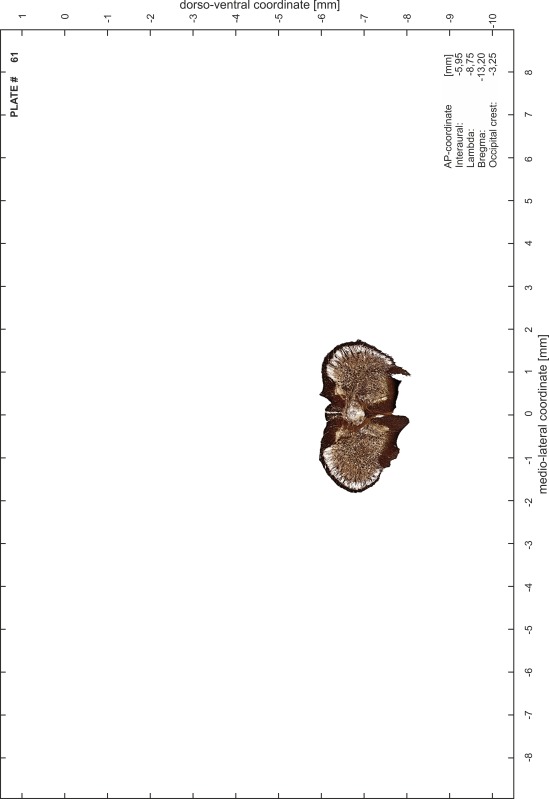

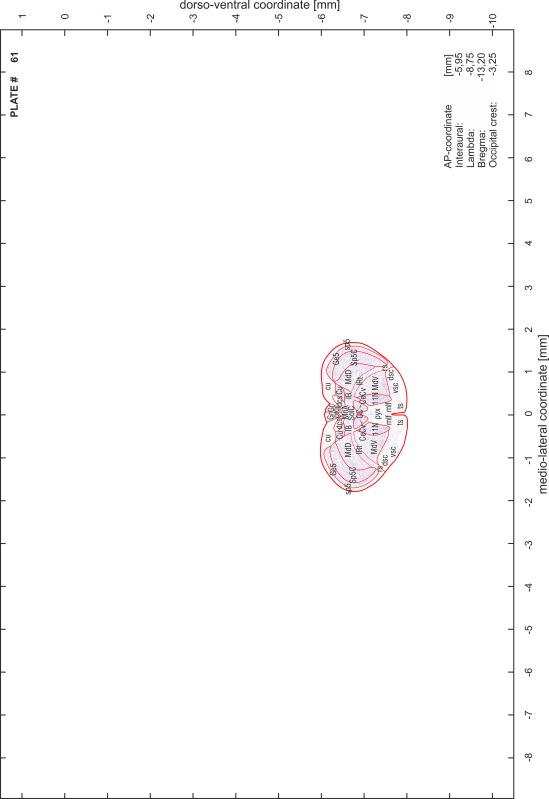

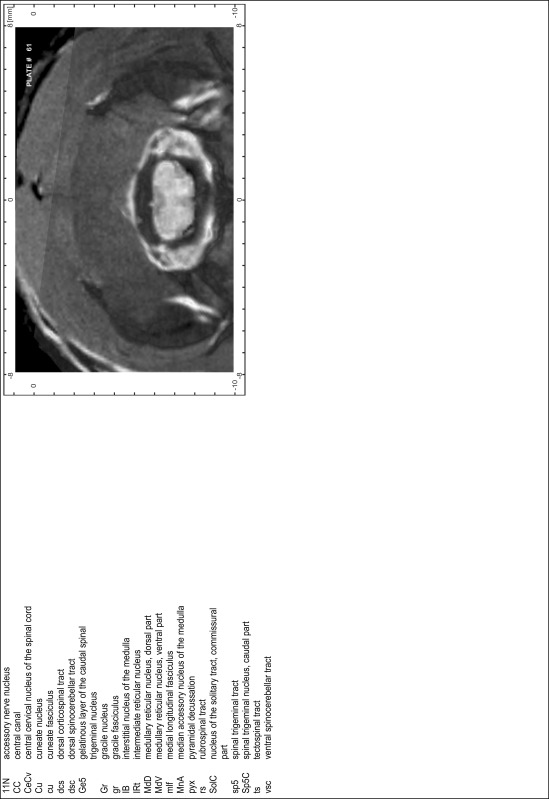

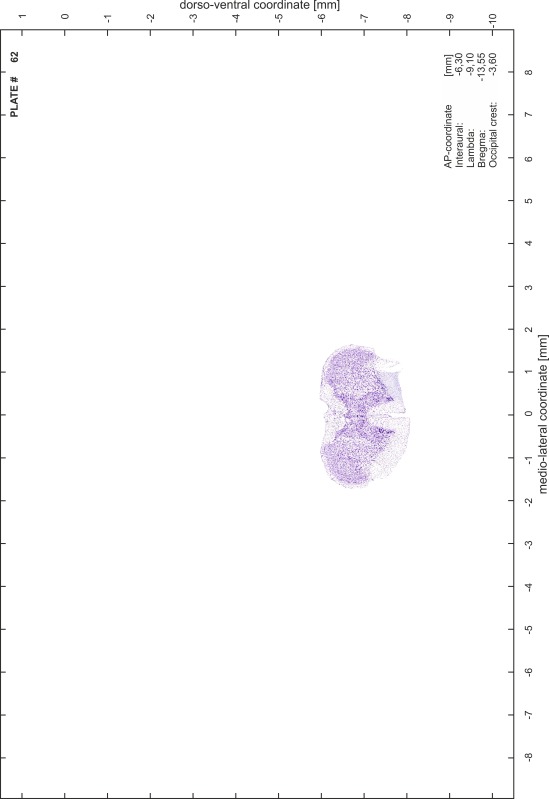

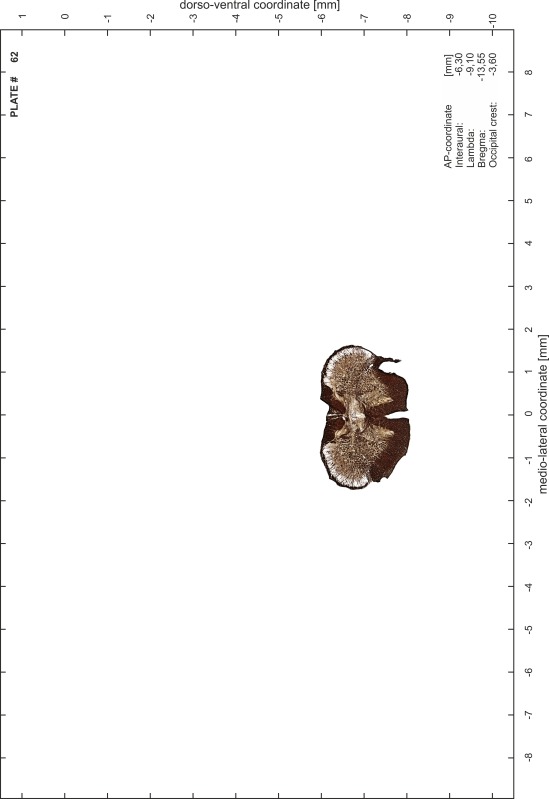

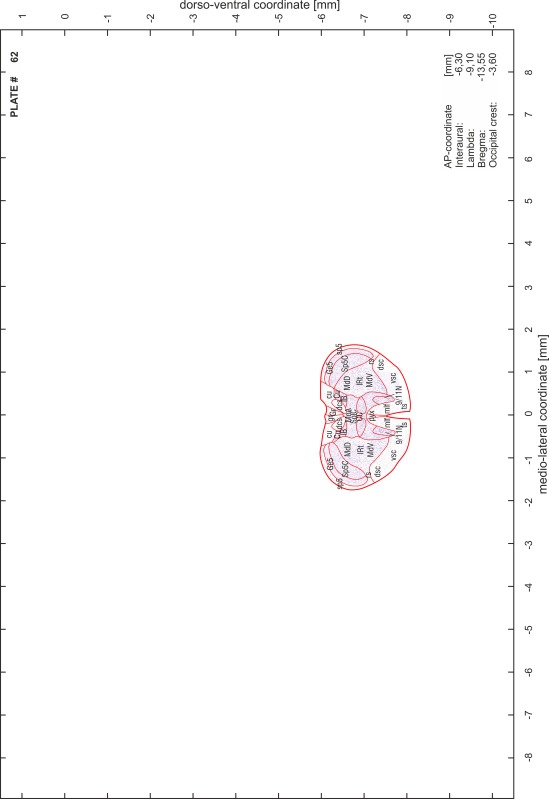

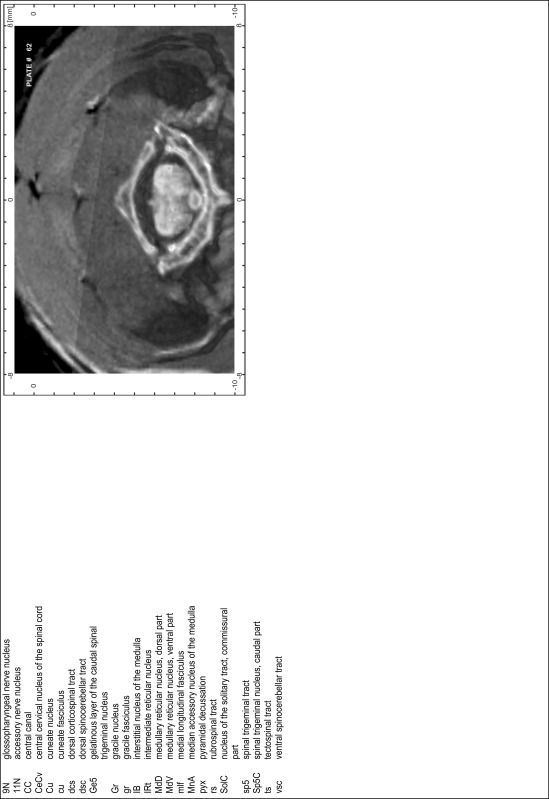
.
